# The Relationship between Cognitive and Emotional Factors and Healthcare and Medication Use in People Experiencing Pain: A Systematic Review

**DOI:** 10.3390/jcm9082486

**Published:** 2020-08-03

**Authors:** Eva Huysmans, Lynn Leemans, David Beckwée, Jo Nijs, Kelly Ickmans, Maarten Moens, Lisa Goudman, Ronald Buyl, Koen Putman, Iris Coppieters

**Affiliations:** 1 Research Foundation Flanders (FWO), Egmontstraat 5, 1000 Brussels, Belgium; 2 Pain in Motion Research Group (PAIN), Department of Physiotherapy, Human Physiology and Anatomy, Faculty of Physical Education & Physiotherapy (KIMA), Vrije Universiteit Brussel, Laarbeeklaan 103, 1090 Brussels, Belgium; 3 Department of Public Health (GEWE), Faculty of Medicine and Pharmacy, Vrije Universiteit Brussel, Laarbeeklaan 103, 1090 Brussels, Belgium; 4 Department of Physical Medicine and Physiotherapy, Universitair Ziekenhuis Brussel, Laarbeeklaan 101, 1090 Brussels, Belgium; 5 Interuniversity Center for Health Economics Research (I-CHER), Laarbeeklaan 103, 1090 Brussels, Belgium; 6 Rehabilitation Research (RERE) Research Group, Department of Physiotherapy, Human Physiology and Anatomy, Faculty of Physical Education & Physiotherapy (KIMA), Vrije Universiteit Brussel, Laarbeeklaan 103, 1090 Brussels, Belgium; 7 Department of Neurosurgery, Universitair Ziekenhuis Brussel, Laarbeeklaan 101, 1090 Brussels, Belgium; 8 Center for Neurosciences, Faculty of Medicine & Pharmacy, Vrije Universiteit Brussel, Laarbeeklaan 103, 1090 Brussels, Belgium; 9 Department of Radiology, Universitair Ziekenhuis Brussel, Laarbeeklaan 101, 1090 Brussels, Belgium; 10 Department of Biostatistics and Medical Informatics, Faculty of Medicine and Pharmacy, Vrije Universiteit Brussel, Laarbeeklaan 103, 1090 Jette, Belgium; 11 Department of Rehabilitation Sciences, Faculty of Medicine and Health Sciences, Ghent University, De Pintelaan 185, 9000 Ghent, Belgium

**Keywords:** healthcare use, pain, cognitions, emotions, health seeking behavior

## Abstract

Pain conditions are among the leading causes of global disability, impacting on global healthcare utilization (HCU). Health seeking behavior might be influenced by cognitive and emotional factors (CEF), which can be tackled by specific therapies. The purpose of this study was to systematically review the evidence concerning associations between CEF and HCU in people experiencing pain. Three databases were consulted: PubMed, Web of Science and EconLit. Risk of bias was assessed using the Downs and Black Checklist (modified). A total of 90 publications (total sample n = 59,719) was included after double-blind screening. In people experiencing pain, positive associations between general anxiety symptoms, depressive symptoms and catastrophizing and pain medication use were found. Additionally, there appears to be a relationship between general anxiety and depressive symptoms and opioid use. Symptom-related anxiety and psychological distress were found to be positively related with consulting behavior. Last, a positive association between use of complementary and alternative medicine and level of perceived symptom control was confirmed in people with pain. For other relationships no evidence or inconsistent findings were found, or they were insufficiently studied to draw firm conclusions, indicating that more research on this topic is needed.

## 1. Introduction

Pain is one of the most reported symptoms [1] and the second most common reason for consulting primary healthcare [2], implying a strong contribution to the global burden of disease [3,4]. Pain conditions are among the leading causes of global disability, in particular low back pain and headache disorders as these are the 2 leading causes of years lived with disability according to the Global Burden of Disease Project 1990–2017 [3,4,5,6]. This entails that pain is impacting on global healthcare utilization (HCU) and productivity loss [7], and especially for chronic pain, this is resulting in high socioeconomic burden due to excessive HCU [7,8].

The International Association for the Study of Pain (IASP) defines pain as “An unpleasant sensory and emotional experience associated with actual or potential tissue damage, or described in terms of such damage” [9]. Pain is not only a unique individual experience in terms of somatosensory characteristics (e.g., different intensity, spread and duration), but also the pain-related cognitive and emotional processes (e.g., pain catastrophizing, hypervigilance and fear) are unique and context-specific to each individual [10,11]. These differences in pain experience impact the, again unique, behavioral actions that people take in response to pain and the influence that pain has on daily life in general [10]. For example, not everyone suffering from pain will use healthcare services. Hence, HCU can be seen as a behavioral action [12], as proposed in the “Behavioral Model of Health Service Use” of Andersen [13].

Although it might seem logic that the propensity to seek care for pain is mainly determined by pain-specific characteristics, this is often not the case as other factors beyond the intensity or duration of the pain episode may be even stronger predictors [12]. According to Andersen’s model, health seeking behavior, which drives HCU, is mediated by predisposing (e.g., sex, age, cultural and social factors), enabling (e.g., access to care, financial factors) and need (e.g., patient and provider’s experience) factors [13]. People’s cognitions and beliefs toward their health status, including their pain symptoms, can be categorized under both predisposing and need factors and are therefore an important component of the model [14]. Additionally, other cognitive and emotional factors (CEF) possibly co-existing with pain symptoms, such as symptoms of depression and anxiety but also catastrophizing and kinesiophobia, may impact people’s need to seek healthcare. Several studies confirmed the presence of maladaptive CEF in at least subgroups of different populations experiencing pain, e.g., the presence of depressive symptoms in low back pain [15,16] and fibromyalgia [17]; the presence of kinesiophobia [18,19,20] and pain catastrophizing [18,20] in post-lumbar surgery patients and the presence of anxiety, depressive symptoms and pain catastrophizing in people suffering from osteoarthritis [21]. Moreover, the relationship between CEF and HCU in people experiencing pain has been suggested numerous times, for example, high levels of catastrophizing have been found to be associated with higher levels of HCU [7,22,23,24,25]. Moreover, Hirsch et al., (2014) [26] found that patients with low back pain and strong beliefs that activity causes pain (i.e., a kinesiophobic way of thinking) have higher HCU and costs, compared to patients with more positive cognitions and attitudes toward physical activity. Furthermore, associations between illness perceptions and HCU were found in a variety of populations, including people with pain [27,28,29,30].

Cognitive and emotional factors are often modifiable by targeted therapies, such as pain neuroscience education and/or cognitive-behavioral therapy [31,32,33]. Given a potential relationship between maladaptive CEF and excessive HCU in patients experiencing pain, interventions specifically targeting CEF could possibly lead to decreases in the need of seeking healthcare, which could imply a reduction of the socioeconomic burden related to pain. A first step toward accomplishing such a socioeconomic benefit would be to identify the cognitive and/or emotional factors that are most likely to affect HCU in this population. Subsequently, specific interventions which are targeting those particular factors can be developed and/or implemented. To the best of our knowledge, the current literature is lacking an extensive overview of those CEF that are associated with HCU in patients experiencing pain.

To address the above outlined knowledge gap, the objective of this systematic review is to answer the question whether a relationship can be confirmed between CEF and HCU, in terms of both amount of HCU and use of different types of healthcare, in people experiencing pain, by systematically reviewing and synthetizing the available literature. It was hypothesized that maladaptive CEF would be positively related with higher amounts of healthcare services and medication use and the use of different types of healthcare, whereas positive CEF were expected to be inversely related with HCU outcomes.

## 2. Methods

### 2.1. Protocol and Registration

This systematic review is reported in accordance with the PRISMA statement (Preferred Reporting Items for Systematic reviews and Meta-Analysis) [34]. The protocol was registered a priori in PROSPERO under the following registration number: CRD42018104980.

### 2.2. Search Strategy

All authors contributed to the development of the search strategy based on their own expertise. The final search (6 August 2019) was conducted by EH in 3 electronic databases: PubMed, Web of Science and EconLit. No limits were applied to the search.

The research question was composed according to the PICO (Population-Intervention-Comparison-Outcome) model [35,36]: “Is there a relationship between CEF (Outcome 1) and HCU (Outcome 2) in people experiencing pain (Population)?” The components “Intervention” and “Comparison” were not relevant for our research question and therefore not defined. The final search strategy was built by combining both free and MeSH terms. Within each separate part of the PICO model, i.e., “Population”, “Outcome 1” and “Outcome 2”, search terms were combined using the Boolean term OR. Between the complete search terms for “Population”, “Outcome 1” and “Outcome 2”, the Boolean term AND was used. The complete search strategy for PubMed can be found in Table A1 (Appendix A). After determining the search string for PubMed, it was adapted for each individual database.

### 2.3. Eligibility Criteria

Full-text observational studies and arms of (quasi-)experimental studies investigating the relationship between CEF and HCU in patients experiencing pain were considered for inclusion in this systematic review.

Cognitive and emotional constructs eligible for inclusion included, but were not limited to, anger, general anxiety symptoms (i.e., general emotion characterized by apprehension and somatic symptoms of tension in which impending danger, catastrophe or misfortune is anticipated [37], not specifically due to the experience of somatic symptoms), symptom-related anxiety symptoms (i.e., anxiety symptoms due to or concerning somatic symptoms, e.g., pain), catastrophizing, depressive symptoms, fear-avoidance beliefs, illness beliefs, psychological distress, stress, self-compassion, symptom vigilance, pain acceptance, perceived symptom control and self-efficacy beliefs. To be suitable for inclusion, these constructs had to be measured using patient-reported instruments. Studies using instruments specifically designed for the diagnosis of psychiatric conditions (e.g., PRIME-MD, Anxiety Disorders Interview Schedule for DSM-IV) were excluded.

Healthcare utilization had to be measured in terms of amount of healthcare used (either for a particular type of HCU or for HCU in general) or presence/absence of a certain type of HCU. Studies only reporting healthcare costs were excluded.

Participants had to be adults (≥18 years old) experiencing some form of pain (acute, subacute or chronic). If (part of) the sample was not experiencing pain, and no subgroup analysis in people with pain was executed, the study was considered not suitable for inclusion. Moreover, papers studying children, patients with dementia, pain during labor or during surgical procedures were excluded.

Full eligibility criteria can be found in Table 1.

### 2.4. Study Selection

Following de-duplication, all retrieved articles were screened for title and abstract by 2 reviewers independently (EH and LL) using Rayyan online software [38,39]. Subsequently, the same 2 reviewers performed the full text screening independently from each other. Percentage agreement was calculated to assess inter-rater reliability. Discrepancies were discussed after each stage of the screening in a consensus meeting with both reviewers and a third independent reviewer (IC).

### 2.5. Data Extraction

The a priori determined data extraction form included the following items: author, year of publication, country, study design, type of population (including pain duration), sample size (including sex distribution and age), outcome measures for CEF and HCU (including the moment of assessment if relevant), objective relevant for the present systematic review, statistics used to investigate the relationship between both outcomes and main findings (including numerical data, e.g., effect sizes, if reported).

Data extraction was performed by the first reviewer (EH) and checked for correctness by the second (LL) and last author (IC). Any discrepancies were discussed in a consensus meeting with all 3 reviewers.

### 2.6. Risk of Bias Assessment

A modified version of the Downs and Black checklist [40] (see Supplementary Material Document S1) was used for risk of bias assessment. This instrument was specifically designed for the risk of bias appraisal of different study designs, including randomized and non-randomized trials and observational studies [40], making it an appropriate risk of bias checklist for the variety of study designs included in this systematic review.

To further increase its suitability for estimating risk of bias for the specific objective of this systematic review, the original checklist was modified. Such modifications have been done before in previous systematic reviews [41,42,43]. The answer option “Not applicable” was added to several questions, as for some study designs particular questions were not applicable, resulting in a different total score depending on the study design. Original question 8 (“Have all important adverse events that may be a consequence of the intervention been reported?”) was omitted from the instrument as our research question is not focusing on an intervention, and therefore, there is no specific interest in potential adverse events. One additional question focusing specifically on the assessment of HCU was added to the section “internal validity—bias”. The question reads as follows: “Was healthcare utilization primarily registered for scientific research?” with answer options (1) Yes, (2) No and (3) Unable to determine. This was deemed important to consider as subtracting HCU data from for example clinical patient files or secondary databases may imply a higher risk for (coding) errors. The last question of the original checklist concerning the power of the reported results was adapted because of the unclarity of the original question. The adjusted question now reads: “Was an a priori sample size calculation performed, and was the anticipated sample size reached, or was a post hoc power analysis performed, which suggested that the results were sufficiently powered?” with answer options (1) Yes; (2) No and (3) Unable to determine.

For further data synthesis (see Section 2.7), all included studies were categorized as presenting low, moderate or high risk of bias by evaluating 3 criteria based on the results of the modified Downs and Black checklist for each study. These criteria were selected during a consensus meeting with EH, IC, LL and DB and were deemed to be the most relevant for estimating risk of bias for the present review. The 3 selected criteria were (1) use of reliable and valid outcome measures, (2) clear reporting of the study results and (3) the study results were generalizable. Each criterium was scored using a color code with, green (the study met the criterium), orange (partly meeting the criterium) and red (the criterium was not met). The first criterium was scored based on item numbers 2 (i.e., the main outcomes were clearly described), 19 (i.e., the outcome measures used were valid and reliable) and 20 (i.e., HCU was primarily registered for scientific research) of the modified Downs and Black checklist. Criterium number 2 was scored based on items 6 (i.e., clear description of the study findings) and 7 (i.e., reporting of estimates of random variability for the main outcomes) of the modified Downs and Black checklist. For the appraisal of the third criterium on generalizability, both items of the Downs and Black checklist (10 and 11) on patient representativeness were taken into account, together with item number 3 (i.e., clear description of the study sample). Studies scored green on a criterium if the study met all respective Downs and Black items for that criterium, orange if 1 item was not met and red if 2 or more items were not met. If a study scored green on all 3 criteria, risk of bias was deemed to be low; if there was uncertainty or absence of 1 out of 3 criteria, risk of bias was scored moderate; all other scenarios were scored high risk of bias.

The appraisal was performed by 2 reviewers (EH and LL) independently. Percentage agreement between both reviewers was calculated to assess inter-rater reliability. Discrepancies were discussed in a consensus meeting with both reviewers (EH and LL) and a third independent reviewer (IC).

### 2.7. Data Synthesis

All extracted data were categorized and synthetized in summarizing evidence tables with their accompanying explanatory results tables. For the reporting of study characteristics and details on the methodology of the included studies, a separate study characteristics table was created, containing the following items: author, year of publication, country, study design, population and duration of pain, sample size, sex and age of the sample, outcome measure(s) for CEF and HCU, moment of assessment, objective of the study relevant for the present systematic review and statistical analysis used.

Categorization was executed on the level of the individual outcome measures reported in a study; therefore, one study could be mentioned under different categories for HCU and/or CEF. The categories were determined based on the expertise of all co-authors and by consultation of additional experts in the field of psychology.

Two main categories of HCU outcomes were identified: (1) amount of HCU and (2) type of HCU. Healthcare use outcome measures were categorized under the first category if they measured the number or frequency of visits, treatments received, hospitalizations or medications used, the length of stay in healthcare facilities, or if the presence of HCU in general was reported without mentioning any particular type of HCU. The second category, “type of HCU”, contains HCU outcome measures reporting on the presence or absence of use of specific healthcare providers, services or medication types, without reporting anything about the amount of healthcare used. A separate summarizing evidence and results table was created for both categories (vide infra).

Within the main categories for HCU, further subcategories were created using a systematic approach. First, categorization was executed by the primary reviewer (EH). Next, all authors were granted the opportunity to provide their feedback. Last, a consensus meeting was organized between EH, LL and IC to discuss the remaining discrepancies and agree upon the final categorization. “Amount of HCU” contained the following subcategories (n = 7): amount of pain medication use (i.e., over-the-counter (OTC) and prescription pain medication use), consultations (i.e., all types of consultations with healthcare providers, excluding complementary and alternative medicine (CAM) visits and emergency visits), emergency HCU (i.e., visits to the emergency department and other unscheduled emergency consultations), hospitalizations (i.e., length of stay and number of individual hospitalizations), CAM use, invasive procedures (i.e., surgeries and other invasive interventions) and HCU in general (in case the study did not make any further specifications). For “Type of HCU”, outcome measures were categorized into the following subcategories (n = 12): pain medication (in case no further specification was made in the original article concerning the type of medication (i.e., OTC/prescription/opioids)), OTC pain medication, prescription pain medication (excluding opioids), opioids, consultations (in case the study did not make any further specifications regarding the level of care (i.e., primary/secondary/tertiary care)), primary care consultations, secondary care consultations, tertiary care consultations, emergency HCU, invasive procedures, hospital admissions and CAM use. A complete overview of the clustering of all HCU outcome measures reported in the included studies can be found in Supplementary Material Table S2.

Cognitive and emotional factors were clustered into 19 different construct groups using the same approach as described for the subcategorization of HCU outcome measures (Supplementary Material Table S3). Additionally, 2 experts in the field of psychology were contacted to provide their feedback about the clustering. The clustering process finally resulted in 15 maladaptive CEF clusters (anger, general anxiety symptoms, symptom-related anxiety symptoms, catastrophizing, depressive symptoms, fear-avoidance beliefs, frustration, health worry, helplessness, negative consequences of symptoms beliefs, negative illness beliefs, psychological distress, stress, symptom vigilance and thanatophobia), 8 positive CEF clusters (illness coherence, pain acceptance, perceived benefits, perceived symptom control, positive mood, psychological flexibility, self-compassion and self-efficacy beliefs) and 3 were CEF for which it was impossible to classify them as being either maladaptive or positive (health attribution, locus of control and perceived cause of symptoms). Maladaptive constructs were expected to be positively related with amount of HCU and presence of different types of HCU, whereas positive constructs were expected to be inversely related with amount of HCU and presence of different types of HCU.

After clustering, a detailed results table was created for each category of HCU outcomes (“amount of HCU” and “type of HCU”) presenting the main results for each analysis of a potential association between a particular HCU outcome and CEF. The table was structured based on the different combinations of a particular HCU subcategory and CEF cluster between which an association was investigated in the literature. For each analysis, the following items were included in the results table: author and year of publication of the original publication, sample size, considered population, CEF and HCU outcome measures, description of how the association was analyzed (including potential other factors considered for multivariate analyses) and main findings (i.e., effect size and significance level, if reported). Subsequently, summarizing tables were created for both categories of HCU outcome measures presenting all investigated associations between a particular subcategory of HCU and CEF cluster and their outcome in terms of a positive, negative or no association, in a more consumable way.

Additionally, a summarizing level of association score was assigned to each investigated association, based on the proportion of analyses reporting a positive, negative or no association and accounting for the risk of bias evaluation. The methodology for this summarizing appraisal was adopted from the method used by Sallis et al., (2000) [44], Van der Horst et al., (2007) [45], Hinkley et al., (2008) [46] and Lubans et al., (2010) [47]. Specifically, if 0–33% of analyses reported a significant association, the result was classified as “no association” (0); if 34–59% of analyses reported a significant association, or if fewer than 4 studies investigated that particular relationship, the result was classified as “inconsistent” or “uncertain”, respectively (?); and if ≥60% of the analyses reported a significant positive or negative association, the result was classified as “positive” (+) or “negative” (−), respectively, based on the direction of association. To account for risk of bias, the method of Lubans et al., (2010) [47] was followed, if after exclusion of high risk of bias studies the association (+/−) or absence of an association (0) was still supported by, respectively, ≥60% or 0–33% of the analyses reporting a positive or negative association, the summary score was up-/downgraded to ++/−−/00.

## 3. Results

### 3.1. Study Selection

**Figure 1:**
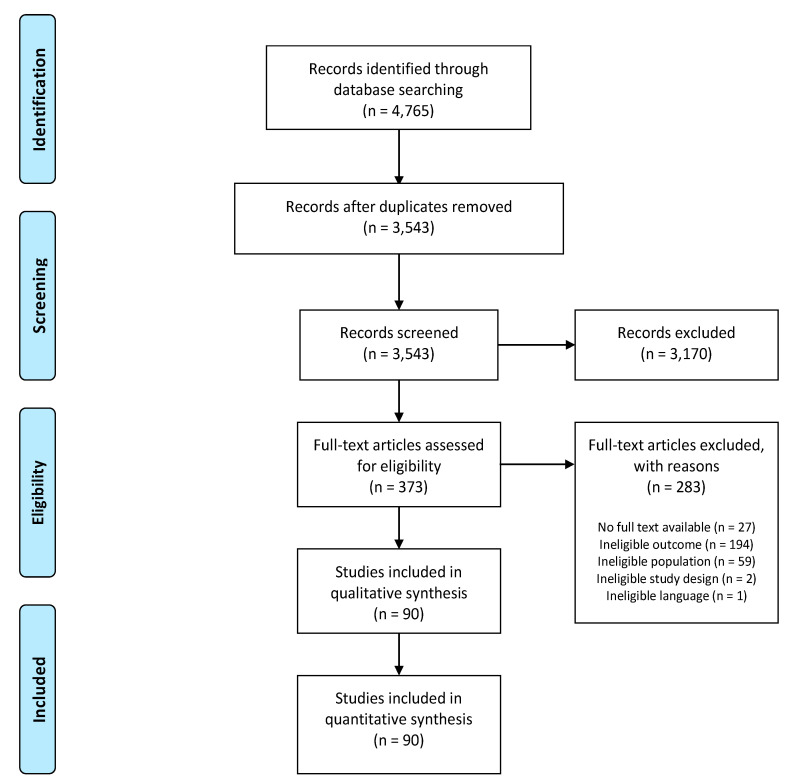
PRISMA flowchart.

The systematic search resulted in 3543 unique studies to be considered for screening, of which 90 were included in this systematic review (Figure 1). Percentage agreement between both reviewers for title and abstract screening and full text screening were 92% and 80%, respectively. Reasons for exclusion were ineligible outcome (n = 1661; e.g., studies using diagnostic tools instead of patient-reported CEF assessment tools and studies only reporting about healthcare costs), ineligible study design (n = 858; e.g., systematic/narrative reviews and letters to the editor), ineligible population (n = 855; e.g., pediatric populations and study samples where not all participants were experiencing pain), ineligible language (n = 49) and no full text available (n = 30).

### 3.2. Study Characteristics

In terms of study designs, 2 randomized controlled trials and 47 cross-sectional, 38 cohort and 3 case-control studies were included, comprising a total sample of 59,719 subjects. A complete overview of the characteristics of the included studies can be found in Table A2 (Appendix B).

### 3.3. Risk of Bias Assessment

Sum scores on the Downs and Black checklist ranged between 8/16 and 15/16 (mean: 12.3/16) for cross-sectional studies (n = 47) [7,48,49,50,51,52,53,54,55,56,57,58,59,60,61,62,63,64,65,66,67,68,69,70,71,72,73,74,75,76,77,78,79,80,81,82,83,84,85,86,87,88,89,90,91,92,93], 10/18 and 17/18 (mean: 12.5/18) for observational cohort studies (n = 26) [94,95,96,97,98,99,100,101,102,103,104,105,106,107,108,109,110,111,112,113,114,115,116,117,118,119], 7/21 and 16/21 (mean: 13.4/21) for single-group interventional cohort studies (n = 11) [25,120,121,122,123,124,125,126,127,128,129], 12/19 and 15/19 (mean: 13.3/19) for case-control studies (n = 3) [130,131,132] and 13/27 and 19/27 (mean: 16/27) for randomized controlled trials (n = 2) [133,134] and multiple-group cohort studies (n = 1) [135]. Percentage agreement between both reviewers was 93%. Most of the studies did not report on an a priori sample size calculation. Another main limitation was the unclarity about the representativeness of the study results for the target population. The complete risk of bias assessment can be found in Table A3 (Appendix C).

Based on the 3 most important criteria to estimate risk of bias for this systematic review, 18 studies showed low risk of bias, 46 moderate risk of bias and 26 high risk of bias. All but 30 studies appeared to have used valid and reliable outcome measures, and all but 7 studies reported their results in a clear and accurate way. The generalizability of the results was not entirely clear in 49 studies, while results did not appear to be generalizable in 16 studies. The remaining 25 studies were deemed to have generalizable results for their target population. The results of the summarizing risk of bias assessment for each study can be found in Table 2.

### 3.4. Evidence for Associations between CEF and HCU in People Experiencing Pain

Results will be discussed for each relationship between a certain CEF and HCU outcome for which a conclusive result could be formulated (i.e., relationship was investigated 4 times or more in the literature). First, the results for relationships between CEF and amount of HCU in people experiencing pain will be reported, and next, the investigated associations with different types of healthcare used will be outlined. Within each of these paragraphs, relationships with maladaptive CEF were discussed first, followed by the positive CEF and the unclassified CEF.

#### 3.4.1. Associations between CEF and Amount of HCU in People Experiencing Pain

The level of *general anxiety symptoms* was found to be consistently positively related with the amount of pain medication use in people experiencing pain, based on univariate analyses (4 analyses reporting a positive association [55,75,108,118] and 1 reporting no association [55]—80% agreement for a relationship), whereas it appeared to be unrelated with the number of consultations with healthcare providers (univariate: 1 analysis reporting a positive association [103], 2 a negative [125] and 5 no association [64,98,103,108]—13%; multivariate: 1 analysis reporting a positive association [103], 1 a negative [125] and 5 no association—14%) and the amount of emergency HCU (univariate: 1 analysis reporting a positive association [110] and 3 no association [85,125]—25%).

In people having pain symptoms, analyses for *symptom-related anxiety symptoms* showed a consistent positive relationship with the amount of consultations with healthcare providers based on univariate analyses (10 analyses reporting a positive association [60,72,92,103,113] and 1 no association [95]—91% agreement), while in multivariate analyses inconsistent results for this association were found (8 analyses reporting a positive association [60,71,72,95,103,113,120] and 8 no association [50,60,71,72,120]—50%).

Univariate analyses showed inconsistent findings for the association between *catastrophizing* and pain medication use (3 reporting a positive association [55,118,126] and 3 no association [54,55]—50%) and consultations with healthcare providers (4 reporting a positive association [74,123,126], 2 a negative [61,128] and 3 no association [54,98]—44%) in people with pain. For the latter, multivariate analyses showed absence of a direct relationship (7 analyses showing no association [25,61,74,123,126]—0%). Furthermore, no relationship was found between level of catastrophizing and the amount of emergency care used by people experiencing pain (both univariate and multivariate: 4 analyses reporting no association [97]—0%).

Level of *depressive symptoms* was found to be consistently positively related with the amount of pain medication use in univariate analyses (6 analyses reporting a positive association [55,75,100,108,118,126] and 1 no association [55]—86%) in people with pain; however, multivariate analyses were not able to confirm this relationship (2 analyses reporting a positive association [100,102] and 5 no association [62,102,108,126]—29%). Analyses investigating the relationship between depressive symptoms and the number of healthcare consultations resulted in inconsistent findings (univariate: 13 reporting a positive association [64,67,72,74,83,98,100,108,113,123,126,132], 1 a negative [61] and 11 no association [48,98,123,125]—52%; multivariate: 13 reporting a positive association [61,74,78,83,96,102,114,120,123] and 19 no association [25,48,50,64,67,71,72,100,102,108,113,117,125,126]—41%). Based on univariate analyses, it was unclear whether depressive symptoms were associated with emergency HCU (3 reporting a positive association [67,83,85], 1 a negative [125] and 3 no association [48,108]—43%) and HCU in general (2 reporting a positive association [48,135] and 3 no association [48,64,135]—40%) in people with pain; on the contrary, multivariate analyses showed absence of evidence for a relationship with both emergency HCU (1 reporting a positive association [83], 1 a negative [125] and 5 no association [67,102,117]—14%) and HCU in general (2 a positive [119,120] and 5 no association [48,57,58,135]—29%). Moreover, no evidence was found based on multivariate analyses for a relationship between depressive symptoms and amount and/or duration of hospitalizations in people having pain (1 analysis reported a positive association [83] and 8 no association [52,102,114,117,125]—11%).

There is absence of evidence for a multivariate relationship between *negative illness beliefs* and *fear-avoidance beliefs* and the amount of consultations with healthcare providers in people with pain (negative illness beliefs: 1 analyses reporting a positive association [50] and 3 no association [50,120,128]—20%, fear-avoidance beliefs: 1 analysis reporting a positive association [105] and 3 no association [105,120,126]—25%).

Level of *psychological distress* appeared to be unrelated with the amount of pain medication use based on univariate analyses in people experiencing pain (4 analyses reporting no association [82,91]—0%). With number of healthcare consultations, on the other hand, 100% agreement for a positive relationship was found based on 8 univariate analyses [63,87,88,111]; however, this relationship could not be confirmed by multivariate analyses (1 reporting a positive association [63], 2 a negative [50] and 10 no association [50,87,88,105,106]—8%).

Analyses investigating the relationship between *stress* (multivariate: 2 reporting a positive association [102,105] and 3 no association [102,105]—40%) and *symptom vigilance* (univariate: 2 reporting a positive association [69,72] and 2 reporting no association [98]—50%) and the amount of healthcare consultations showed inconsistent findings in people with pain.

Concerning the relationship between HCU and positive CEF in people experiencing pain inconsistent findings for a potential association between *pain acceptance* and the amount of pain medication use were found (univariate: 3 analyses reporting a negative association [55,109] and 5 no association [55,109,124]—38%; multivariate: 2 a positive [62,109] and 3 no association [62,109]—40%).

Additionally, inconsistent results were found in univariate analyses for a potential association between self-efficacy beliefs and the amount of healthcare consultations (3 reporting a negative association [55,126] and 5 no association [64,98]—38%). Based on multivariate analyses, no evidence for a relationship between the latter was found (1 reporting a negative association [67] and 4 no association [64,126,129]—20%).

There was absence of evidence for a relationship between *locus of control* and the amount of healthcare consultations in people having pain, based on multivariate analyses (1 reporting a positive association [106], 1 a negative [106] and 4 no association [106]—17%).

All remaining analyses investigating the association between CEF and the amount of healthcare used in people experiencing pain were investigated less than 4 times. All results for associations between CEF and amount of HCU were summarized in Table A4. More comprehensive details on the analyses reported in the literature that investigated this relationship and their results can be found in Supplementary Material Table S4.

#### 3.4.2. Associations between CEF and Type of HCU in People Experiencing Pain

In people experiencing pain, a positive association was found between level of *general anxiety symptoms* (4 analyses showing a positive association [94,121] and 2 no association [122,130]—67% agreement for a relationship) and *depressive symptoms* (8 analyses showing a positive association [86,94,96,121,122] and 3 no association [61,74,130]—73%) and using opioids based on univariate analyses. However, for depressive symptoms, multivariate analyses indicated the absence of an association with opioid use (2 analyses showing a positive association [86,101] and 4 no association [62,86,101,121]—33%). Moreover, no evidence was found for a relationship between depressive symptoms and prescription pain medication use (univariate: 2 analyses showing a positive association [48,76] and 16 no association [48,49]—11%), OTC pain medication use (univariate: 1 analyses showing a positive association [48] and 4 no association [48,49]—20%), having primary care consultation (univariate: 2 analyses showing a positive association [112] and 5 no association [48,104]—29%), undergoing invasive procedures (univariate: 1 analysis showing a positive association [48] and 4 no association [48,131]—20%) and using CAM services (univariate: 6 analyses showing a positive association [48,73,115] and 23 no association [48,73,77,112]—21%; multivariate: 5 analyses showing no association [73,115]—0%) in people with pain.

*Symptom-related anxiety symptoms* were found to be consistently positively related with having primary care consultations in patients with pain (univariate: 3 analyses reporting a positive association [60] and 1 no association [65]—75%).

Multivariate analyses found a consistently positive association between the level of *catastrophizing* and using pain medication in people experiencing pain (4 analyses found a positive relationship [53,84,89] and 2 no association—67%).

*Psychological distress* was found to be unrelated with using opioids (multivariate: 4 analyses showing no association [107,111]—0%), having consultations (univariate: 12 analyses showing no association [66,68,79,87,90,91]—0%; multivariate: 1 analysis showing a negative association [87] and 7 no association [68,80,87]—13%), having emergency consultations (multivariate: 1 analysis showing a positive association [88] and 3 no association [88,107]—25%), undergoing invasive procedures (multivariate: 2 analyses showing a positive association [107,111] and 5 no association [107]—29%) and using CAM (univariate: 4 analyses showing no association [82]—0%) in people having pain. Inconsistent evidence was found for the potential relationship between psychological distress and having primary care consultations in people experiencing pain (multivariate: 2 analyses showing a positive association [59,65] and 2 no association [65,80]—50%).

Both univariate and multivariate analyses indicated a positive relationship between perceived symptom control and using CAM services in people with pain (univariate: 3 analyses showing a positive association [73] and 1 no association [73]—75%; multivariate: 3 analyses showing a positive association [73] and 1 no association [73]—75%).

Based on univariate analyses there appears to be no association between *self-efficacy beliefs* and having secondary care consultations (1 analysis showing a positive association [51] and 3 no association [51]—25%)

All remaining analyses investigating the association between CEF and type of healthcare services used by people experiencing pain were investigated less than 4 times. Summarized results for associations between CEF and type of HCU can be found in Table A5. More comprehensive details on the analyses investigating the relationship between CEF and type of HCU retrieved from the literature and their results can be found in Supplementary Material Table S5.

## 4. Discussion

### 4.1. Discussion of the Results

#### 4.1.1. Summary of the Results

The present systematic review investigated whether a relationship between CEF and HCU, in terms of amount of HCU and type of healthcare services used, in people experiencing pain could be identified by synthesizing the existing literature. Based on univariate analyses on amount of HCU outcomes, a positive association between general anxiety symptoms and depressive symptoms and amount of pain medication use and between symptom-related anxiety symptoms and psychological distress and the number of consultations in people with pain could be confirmed. However, based on the results of multivariate analyses, it could be assumed that there is no direct relationship between depressive symptoms and pain medication use and between psychological distress and number of consultations. The level of general anxiety symptoms seems to have neither direct nor indirect relationship with the number of consultations with healthcare providers that people with pain are having. Additionally, no direct or indirect relationship could be confirmed between catastrophizing and the amount of emergency care use. In terms of type of healthcare services used, a univariate positive association was shown between general anxiety symptoms and depressive symptoms and the use of opioids in people with pain. However, for the latter, the relationship might be only indirect as multivariate analyses were not able to confirm the association. Overall, it can be stated that, apart from the univariate relationship with opioid use, depressive symptoms are not firmly related with the use of particular types of HCU. Moreover, psychological distress was found to be unrelated with the use of several healthcare services in people experiencing pain. For catastrophizing, on the other hand, the existing literature confirmed a multivariate positive relationship with using pain medication. Furthermore, a univariate positive association between symptom-related anxiety symptoms and having primary care consultations was found. Last, the literature indicated a positive association between perceived symptom control and the use of CAM services, based on both univariate and multivariate analyses.

Nevertheless, there is clearly an interest in this subject in the available literature, and many associations between particular combinations of CEF constructs and HCU categories were only scarcely studied, making it impossible to draw firm conclusions about the existence of these relationships. Furthermore, when associations were sufficiently studied, the findings were often inconsistent across the studies.

#### 4.1.2. Discussion of Confirmed Associations

Although it can be stated that the literature on the relationship between many of the reported CEF and HCU outcomes is still inconclusive, it is possible to confirm the presence or absence of some associations.

Concerning pain medication use, the literature confirms an at least indirect relationship between the level of general anxiety and/or depressive symptoms and the amount of pain medication used [55,75,100,102,108,118,126,133] and the odds of using opioids [86,94,96,121,122]. Furthermore, the level of catastrophizing was directly related with the odds for using pain medication in general [53,84,89]. The latter are important findings in the light of the current opioid epidemic [136] in Northern America, Canada and Australia [137] and to a lesser extent in European countries, such as Germany and the UK [138]. Although all patients with pain should receive an appropriate treatment, and opioids can be useful in the treatment of short-term acute pain episodes [136], the opioid epidemic is an example of how HCU for pain can become excessive with major negative impact on the individual and society. This is characterized by, for instance, abusive prescription practices of medical practitioners, illicit opioid overdose-related deaths and high socio-economic burden with an estimated economic liability of $78 billion a year in the USA [139], not even including the costs of decreased quality of life, psychological distress and social dysfunction [136,140]. The relationship between the odds for opioid use, and by extension pain medication use in general, and the level of symptoms of depression, general anxiety and catastrophizing suggests that it might be possible to decrease excessive opioid use by managing depressive symptoms better [48]. Moreover, it has been suggested that there might be a positive relationship between symptoms of depression [55,141,142,143,144] and/or anxiety [55,141,142,144] and non-adherence to medication recommendations (e.g., pain medication dependence, overuse or taking someone else’s prescription). Such non-adherence to medication recommendations is one of the causes of the opioid epidemic, indicating that it might be useful to target these CEF in clinical practice in an attempt to decrease medication misuse, withholding great potential for impacting upon the opioid epidemic. Although more research is needed to confirm these assumptions.

Maladaptive CEF were expected to be positively related with consultation behavior. Based on the available literature, we cannot confirm this hypothesis for many CEF, but there is potential for a positive association between symptom-related anxiety symptoms and both the number of healthcare consultations [60,71,72,92,95,103,113,120] and the odds for having primary care consultations [60]. However, it should be stated that this might only be an indirect relationship as only univariate analyses showed a consistent positive association. This can possibly be explained by the notion that patients who are experiencing pain-related anxiety and/or fear often deem their condition to be threatening, which drives them into safety-seeking behavior, such as avoiding symptom-provoking activities [145] and frequent consultations with healthcare providers [71,72,146]. Additionally, there is potential for a positive association between the propensity to have healthcare consultations and catastrophizing [7,89] and psychological distress [59,65,82], although these relationships were only scarcely studied, making it hard to draw firm conclusions about them. The presence of symptoms of anxiety and/or fear and catastrophizing might go hand in hand in some patients experiencing pain, high anxiety and/or fear levels and could lead to more catastrophizing about a painful episode, and catastrophizing could in turn result in more anxiety and/or fear, suggesting a bidirectional relationship [145]. Moreover, it is known that anxiety [147], catastrophizing [147,148,149,150] and psychological distress [151,152,153] can enhance pain intensity and related disability. This augmented pain experience combined with the fact that catastrophizers often view their condition as threatening might lead to a faster decision to consult a healthcare provider.

Remarkably, most of the maladaptive CEF for which positive associations with HCU were found are part of the fear-avoidance model (i.e., depressive symptoms, general anxiety symptoms, symptom-related anxiety/fear symptoms and catastrophizing) [145,154]. This leads to the consideration that the influence of these CEF on pain intensity and disability might also play a role in their relationship with HCU, which has been suggested in the literature before [154]. Although for some factors consistent independent relationships were shown, based on multivariate analyses. Additionally, Alschuler et al., (2012) [48] could not confirm that presence of depressive symptoms had a moderator effect on the relationship between pain intensity and HCU. It should also be considered that the fear-avoidance model was designed based on chronic pain conditions, whereas different types of pain conditions were included in this systematic review. However, when looking at the results for chronic and acute pain conditions separately, we did not find any outstanding differences. Furthermore, it should be taken into account that due to their involvement in the fear-avoidance model, these CEF are possibly more popular in research, making them more extensively studied compared to some of the other CEF included in this systematic review.

Some additional aspects that might have led to the presence or absence of a confirmation of certain associations in particular studies should be considered. In the introduction, it was already mentioned that the modifiable CEF considered in this systematic review can be categorized under the need and/or predisposing factors of Andersen’s model of health services use, next to many other demographic and clinical patient-related characteristics [13,14,155]. Although it would go beyond the scope of this systematic review to go into too much in detail, it is worth considering that the results of the included studies might have been influenced by factors from the third component in van Andersen’s model: enabling factors [12,13,14] [WU1]. These enabling factors can be individual-specific (e.g., income), but many of these factors apply to an entire community, healthcare system or patient population. Therefore, the amount of and whether people seek care can be dependent on the healthcare system in which they are residing, based on for instance, differences in the accessibility [13,14,50,51,68,155,156] and cost of care [155]. This can explain why certain associations between CEF and HCU outcomes might only be present in specific countries with their particular healthcare and/or health security systems.

Another factor that should be considered when interpreting HCU among different conditions and pathologies is the fact that for some health conditions, the healthcare trajectories are more predetermined than for others. Therefore HCU of some people experiencing pain will be more selective and therefore to a greater extent determined by the free will of the patient, while for others care seeking behavior will be highly influenced by the fact that a standard care trajectory is available for their condition [157]. It might be assumed that for the latter CEF will play a less important role in the decision to have care.

### 4.2. Directions for Future Research

Several potential associations between particular CEF and HCU outcomes were investigated less than 4 times; wherefore, it was deemed impossible to draw firm conclusions about the effective existence of these relationships. However, the limited results for some of them point towards a confirmation of our hypotheses (i.e., ≥60% of the analyses confirm hypothesis). More specifically, concerning maladaptive CEF, there is potential for an, at least indirect, positive relationship between (1) general anxiety symptoms and the propensity to seek emergency care [110] and to use CAM services [115], (2) catastrophizing and the odds of using prescription pain medication [84], using opioids [84,122], having consultations with healthcare providers in general [7,89] and having tertiary care consultations in particular [56], (3) depressive symptoms and having hospitalizations [52], (4) fear-avoidance beliefs and the amount of pain medication use [126] and the chance of having a healthcare consultation [68], (5) frustration and using pain medication [59], (6) health worry and number of consultations with healthcare providers [132], (7) helplessness and the amount of healthcare consultations [128] and the odds for having secondary care consultations [116], (8) the level of beliefs of negative consequences of health condition and the propensity to use pain medication [59] and to have primary [59] and secondary care consultation [116], (9) negative illness beliefs and the chance of using pain medication [59], having healthcare consultations in general [68] and primary care consultations in particular [59] and the amount of HCU in general [120], (10) psychological distress and the number of emergency room visits [111] and hospital admissions [111] and the propensity of using pain medication in general [59] and prescription pain medication in particular [81,111], and of having primary care consultations [59,65,82] and (11) symptom vigilance and the amount of healthcare consultations [69].

Moreover, for some relationships between positive CEF clusters and HCU outcomes that were investigated less than 4 times, the limited results met our expectations of showing a negative association. This was the case for the association between: (1) illness coherence and the odds for pain medication use [59], (2) pain acceptance and the propensity of using opioids [62], (3) perceived symptom control and the chance of using pain medication [59] and having consultations [66], (4) positive mood and the amount of emergency HCU [102] and hospitalizations [102], (5) psychological flexibility and the amount of pain medication use [70] and consultations with healthcare providers [70] and (6) self-efficacy beliefs and the amount of pain medication use [55,75,126], emergency HCU [67,93] and HCU in general [135] and the odds for using prescription pain medication [81].

It would be erroneous to assume that these results provide us with conclusive evidence for a relationship between these variables, but also considering the impact that revealing these associations could have on socioeconomic burden, it is clear that further research on this topic is needed.

Next to some associations that can be assumed to be non-existent, there is evidence for the presence of several relationships between CEF and HCU. Although for many it is not clear yet what the mechanism behind these associations is and whether they are independent relationships. This should be further investigated before conclusions can be drawn about potential causal interactions between CEF and HCU. In case the suggested causal interactions can be confirmed, further research could focus on the development and/or implementation of interventions that address CEF in an attempt to keep HCU to an optimal level and avoid excessive use.

### 4.3. Implications for Clinical Practice

The findings of this systematic review indicate that there is evidence for a relationship between several maladaptive CEF (in particular for general and symptom-related anxiety symptoms, catastrophizing and depressive symptoms) and HCU measures in patients experiencing pain, whereas for others more research is needed to confirm a potential relationship. Although it is impossible to conclude anything about causal interactions, it can carefully be suggested that interventions specifically targeting the former CEF could lead to decreases in HCU towards an optimal level, which potentially implies a socioeconomic benefit. An example of such a therapy option is pain neuroscience education, which has been found to effectively address maladaptive CEF and enhance positive CEF in several patient populations experiencing pain [158,159], including patients with chronic spinal pain [32,160,161,162], knee osteoarthritis [163,164,165], fibromyalgia [166] and chronic fatigue syndrome [167]. Moreover, Louw and colleagues found that one preoperative session of pain neuroscience education in patients undergoing surgery for lumbar radiculopathy resulted in large long-term decreases of postoperative healthcare costs [168,169], which are inseparably linked to HCU. Based on the findings of this systematic review, it could be suggested that these results on healthcare costs might be mediated by the direct effect of pain neuroscience education on CEF.

### 4.4. Strengths and Limitations

To our knowledge, this is the first systematic review specifically focusing on the relationship between CEF and HCU in patients experiencing pain. Although many different constructs of CEF and different ways of approaching HCU were reported in the included studies, making it a complex matter, the authors aimed at giving a digestible overview of the evidence so far. This was achieved by clustering outcomes and creating summarizing tables as has been done before in previous systematic reviews investigating associations between certain variables in non-pain populations [44,45,46,47]. The results of this systematic review indicate that some modifiable CEF are associated with HCU in patients experiencing pain, which might imply that targeted interventions could eventually lead to decreased HCU.

Furthermore, this review has several methodological strengths that ensure minimization of potential bias: the double-blind screening of the literature and risk of bias appraisal, including consensus meetings when necessary; the fact that several databases where consulted aiming at a complete representation of the literature and the final inclusion of a large number of studies (n = 90) comprising a large sample of participants with pain (n = 59,719).

When interpreting the results of this systematic review, a number of limitations related to the included studies should be taken into account. First, most of the studies showed moderate risk of bias (51%), with some studies even showing high risk of bias (29%), and the minority were rated as having low risk of bias (20%). In light of this, readers should keep in mind that the generalizability of some of the results might be questionable. Second, the research question of the present systematic review was often only a secondary objective of the included studies, meaning that results were not always described in detail and the included studies might have been underpowered for this particular research question, which increases the risk for type II errors, or false negatives.

Next, some limitations related to the methodology of this systematic review should be considered. In an attempt to give a comprehensive overview of the literature about the relationship of CEF and HCU in patients experiencing pain, it was necessary to apply broad in- and exclusion criteria. Due to the amount of included studies, their analyses and the variety in outcome measures, it was necessary to cluster outcomes, making the results more consumable. Inevitably, this led to a loss of information; however, thanks to this clustering, it became possible to draw firm conclusions about particular relationships. Moreover, to our knowledge there are no standardized or validated criteria available yet to rate the level of associations in systematic reviews especially focusing on relationships between variables. Therefore, we aimed at using a methodology that was suitable for our research question and had been used before in comparable systematic reviews. This was the case for the chosen methodology, which had been used 4 times before [44,45,46,47] and was deemed to be reliable to make an estimation of the level of association for the pooled results. Furthermore, this review focused solely on associations between CEF and HCU, wherefore, no conclusions about causal relationships could be made based on the results.

## 5. Conclusions

Based on the available evidence, an at least indirect positive association between general anxiety symptoms, depressive symptoms and catastrophizing and the amount of pain medication use can be confirmed in people with pain. Additionally, general anxiety and depressive symptoms appear to be univariately related with the propensity to use opioids. In terms of consultation behavior in people experiencing pain, an at least indirect relationship with, symptom-related anxiety and psychological distress was found. Use of CAM services appeared to be positively related with the level of perceived symptom control. For other relationships, no evidence was found, inconsistent findings were reported, or they were insufficiently studied to draw firm conclusions. However, in the latter case, the limited results for some relationships pointed towards a confirmation of our hypothesis that maladaptive CEF were related to more HCU, and the other way around for positive CEF, indicating that more research on this topic is needed. Although it is impossible to draw conclusions about causal interactions, the results of this systematic review carefully suggest that it could be important to address maladaptive CEF, such as anxiety symptoms, catastrophizing and psychological distress, in clinical practice to potentially decrease excessive healthcare seeking behavior in people experiencing pain and the socio-economic burden related to pain.

## Figures and Tables

**Table 1:** Eligibility criteria.

Inclusion	Exclusion
Full text (arms of) (quasi-)experimental studies or observational studies	Case reports, systematic reviews and meta-analyses, narrative reviews, letters to the editor, expert opinions, conference abstracts, studies without available full-text version
English, French or Dutch written	Other languages
Evaluation of the relationship between CEF, including but not limited to, anger, anxiety symptoms, catastrophizing, depressive symptoms, fear-avoidance beliefs, illness beliefs, psychological distress, stress, self-compassion, symptom vigilance, pain acceptance, perceived symptom control and self-efficacy beliefs, and HCU	No investigation of the relationship between CEF and HCU
CEF assessed by means of patient-reported instruments	Instruments specifically designed for physicians to diagnose psychiatric conditions (e.g., PRIME-MD, Anxiety Disorders Interview Schedule for DSM-IV)
HCU reported in terms of amount of HCU (of a particular type of HCU or of HCU in general) or in terms of type of healthcare services used (absence/presence of certain types of HCU)	Studies only reporting healthcare costs, without mentioning utilization, those only investigating adherence to recommendations, medication misuse or substance abuse for non-medical purposes and studies concerning the use of assistive or ergonomic devices (e.g., prosthesis, orthosis and canes)
Participants had to be adults (≥18 years old) experiencing either acute, subacute or chronic pain.	Complete or part of the sample was not experiencing pain and no separate analysis for people with pain was executed. Studies on children, women experiencing labor pain, people suffering from dementia, intraoperative subjects and palliative patients.
Studies reporting a quantified association or relationship analyzed by using statistics.	Studies only reporting observations without quantitative analysis or studies only including qualitative analyses.

CEF: cognitive and emotional factors; HCU: healthcare use.

**Table 2:** Summarizing risk of bias assessment.

	Reliable and Valid Outcome Measures ^1^	Reporting of Results ^1^	Generalizability of Results ^1^	Risk of Bias (High/Moderate/Low) ^2^
Alschuler (2012) [48]				Moderate
Asmundson (2001) [49]				High
Biggs (2003) [50]				Moderate
Boyer (2009) [51]				Moderate
Buse (2012) [94]				Moderate
Carroll (2016) [96]				High
Carroll (2018) [95]				High
Ciechanowski (2003) [25]				Low
Citero (2007) [97]				Moderate
Cronan (2002) [135]				High
Cronin (2018) [93]				High
Cronin (2019) [52]				Moderate
Daltroy (1998) [133]				High
De Boer (2012) [53]				Moderate
Demmelmaier (2010) [98]				Low
Dobkin (2006) [99]				Moderate
Durá-Ferrandis (2017) [134]				Low
Elander (2003) [54]				Moderate
Elander (2014) [55]				Moderate
Engel (1996) [100]				High
Fink-Miller (2014) [56]				High
Gebauer (2019) [101]				High
Gil (2004) [102]				Moderate
Görge (2017) [120]				Moderate
Grant (2000) [57]				Moderate
Hadlandsmyth (2013) [103]				Moderate
Harden (1997) [130]				High
Harding (2019) [58]				Moderate
Hill (2007) [59]				Low
Howell (1999) [60]				Moderate
Huffman (2017) [121]				High
Jensen (1994) [128]				Moderate
Jensen (2006) [122]				High
Jordan (2006) [104]				High
Jöud (2017) [7]				Moderate
Kapoor (2012) [123]				High
Kapoor (2014) [61]				Moderate
Keeley (2008) [105]				Moderate
Kratz (2018) [62]				Moderate
Kuijper (2014) [106]				Moderate
Lee (2008) [63]				Moderate
Lentz (2018) [107]				Low
Levenson (2008) [108]				Moderate
Lozano-Calderon (2008) [131]				Low
Lozier (2018) [64]				Moderate
Macfarlane (1999) [65]				Moderate
Macfarlane (2003) [66]				Moderate
Mann (2017) [67]				Moderate
Mannion (2013) [68]				Moderate
McCracken (1997) [69]				Moderate
McCracken (2005; Pain) [109]				Low
McCracken (2005; Beh Res Ther) [124]				Low
McCracken (2007) [70]				Moderate
Mourad (2016) [72]				Moderate
Mourad (2018) [71]				Moderate
Musey (2018) [110]				High
Navabi (2018) [111]				High
Ndao-Brumblay (2010) [73]				High
Newman (2018) [74]				High
Nielsen (2015) [75]				Moderate
Osborne (2007) [129]				Moderate
Pagé (2019) [112]				Low
Philpot (2018) [125]				High
Pierce (2019) [76]				High
Primavera (1994) [127]				High
Rosenberg (2008) [77]				Moderate
Shmagel (2016) [78]				Moderate
Talley (1998) [79]				Low
Thorstensson (2009) [80]				Low
Torrance (2013) [81]				Low
Trask (2001) [82]				Moderate
Tremblay (2018) [113]				Moderate
Tsuji (2019) [83]				Low
Ullrich (2013) [114]				Moderate
Valdes (2015) [84]				Moderate
van Tilburg (2008) [115]				Low
Vervoort (2019) [116]				Moderate
Villani (2010) [85]				High
Vina (2019) [86]				Low
Von Korff (1991) [87]				High
Von Korff (2007) [132]				High
Walker (2016) [88]				Moderate
Wideman (2011) [126]				Moderate
Wijnhoven (2007) [89]				Moderate
Williams (2006) [90]				Low
Williams (2018) [117]				High
Wong (2019) [118]				High
Woodhouse (2016) [119]				Moderate
Zebenholzer (2016) [91]				Low
Zondervan (2001) [92]				Low

^1^ Each criterium was scored using a color code: green (the study met the criterium), orange (uncertainty about the criterium) and red (the criterium was not met). ^2^ Overall risk of bias score: (1) green on all 3 criteria: low risk of bias; (2) orange or red on 1 criterium: moderate risk of bias; (3) >1 orange or red criterium: high risk of bias.

## Supplementary Materials

The following are available online at https://www.mdpi.com/2077-0383/9/8/2486/s1. Document S1: Modified Downs and Black Checklist for risk of bias assessment. Table S2: Clustering of HCU outcome measures. Table S3: Clustering of outcome measures for CEF. Table S4: Comprehensive overview of the results of analyses investigating associations between CEF and amount of HCU. Table S5: Comprehensive overview of the results of analyses investigating associations between CEF and HCU.

## Appendix A

**Table A1:** Complete systematic search strategy for PubMed.

Search Terms		#Hits in PubMed (Date of Search)
**Population**		
*	Pain	726,663 (27 April 2018)
**Outcome 1: CEF**		
	“anxiety” [MeSH terms]	
	“catastrophization” [MeSH terms]	
	“pain perception” [MeSH terms]	
	“acceptance”	
	“anger”	
	“anxiety”	
	“attention to pain”	
	“attitude”	
	“attribution”	
	“attributions”	
	“catastrophic thinking”	
	“catastrophisation”	
	“catastrophising”	
	“catastrophization”	
	“catastrophizing”	
	“depressive symptoms”	
	“depressive thoughts”	
	“emotional stress”	
	“fear of movement”	
	“fear of pain”	
	“hypervigilance”	
	“illness belief”	
	“illness beliefs”	
	“illness cognition”	
	“illness cognitions”	
	“illness perception”	
	“illness perceptions”	
	“kinesiophobia”	
	“mental stress”	
	“mind set”	
	“mindset”	
	“pain attention”	
	“pain awareness”	
	“pain belief”	
	“pain beliefs”	
	“pain catastrophisation”	
	“pain catastrophising”	
	“pain catastrophization”	
	“pain catastrophizing”	
	“pain cognition”	
	“pain cognitions”	
	“pain perception”	
	“pain perceptions”	
	“pain-related stress”	
	“pain thoughts”	
	“pain vigilance”	
	“pain vigilant”	
	“perceived injustice”	
	“psychological stress”	
	“psychosocial”	
	“resilience”	
	“rumination”	
	“self-compassion”	
	“self-efficacy”	
	“somatisation”	
	“somatization”	
	“Tampa scale”	
	“vigilance to pain”	
*	((((((((((((((((((((((((((((((((((((((((((((((((((((((((((“anxiety” [MeSH terms]) OR “pain perception” [MeSH terms]) OR “catastrophization” [MeSH terms]) OR “pain attention”) OR “attention to pain”) OR “pain awareness”) OR “vigilance to pain”) OR “pain vigilant”) OR “hypervigilance”) OR “pain vigilance”) OR “catastrophisation”) OR “catastrophization”) OR “catastrophising”) OR “catastrophizing”) OR “catastrophic thinking”) OR “pain catastrophisation”) OR “pain catastrophization”) OR “pain catastrophising”) OR “pain catastrophizing”) OR “fear of movement”) OR “kinesiophobia”) OR “Tampa scale”) OR “illness cognitions”) OR “illness cognition”) OR “illness belief”) OR “illness beliefs”) OR “illness perception”) OR “illness perceptions”) OR “anxiety”) OR “fear of pain”) OR “psychosocial”) OR “attitude”) OR “pain belief”) OR “pain beliefs”) OR “pain perception”) OR “pain perceptions”) OR “pain cognition”) OR “pain cognitions”) OR “pain thoughts”) OR “self-efficacy”) OR “attribution”) OR “attributions”) OR “resilience”) OR “mindset”) OR “mind set”) OR “acceptance”) OR “self-compassion”) OR “anger”) OR “rumination”) OR “perceived injustice”) OR “depressive thoughts”) OR “mental stress”) OR “psychological stress”) OR “emotional stress”) OR “pain-related stress”) OR “somatization”) OR “somatisation”) OR “depressive symptoms”)	783,679 (27 April 2018)
**Outcome 2: HCU**		
	“delivery of health care/utilization” [MeSH terms]	
	“health care costs” [MeSH terms]	
	“ambulatory care cost”	
	“ambulatory care costs”	
	“ambulatory care delivery”	
	“ambulatory care expenditure”	
	“ambulatory care use”	
	“ambulatory care utilization”	
	“care trajectories”	
	“care trajectory”	
	“continuity of care”	
	“cost of drugs”	
	“cost of health care”	
	“cost of healthcare”	
	“delivery of drugs”	
	“delivery of health care”	
	“delivery of health services”	
	“delivery of healthcare”	
	“doctor shopping”	
	“drug cost”	
	“drug costs”	
	“drug delivery”	
	“drug expenditure”	
	“drug spending”	
	“drug use”	
	“drug utilisation”	
	“drug utilization”	
	“health care cost”	
	“health care costs”	
	“health care delivery”	
	“health care expenditure”	
	“health care savings”	
	“health care seeking behavior”	
	“health care seeking behaviour”	
	“health care service costs”	
	“health care service delivery”	
	“health care service seeking behavior”	
	“health care service use”	
	“health care service utilisation”	
	“health care service utilization”	
	“health care services delivery”	
	“health care services utilisation”	
	“health care services utilization”	
	“health care spending”	
	“health care use”	
	“health care utilisation”	
	“health care utilization”	
	“health seeking behavior”	
	“health seeking behaviour”	
	“health service delivery”	
	“health service expenditure”	
	“health service cost”	
	“health service costs”	
	“health service savings”	
	“health service spending”	
	“health service use”	
	“health service utilisation”	
	“health service utilization”	
	“health services cost”	
	“health services delivery”	
	“health services expenditure”	
	“health services use”	
	“health services utilisation”	
	“health services utilization”	
	“healthcare cost”	
	“healthcare costs”	
	“healthcare delivery”	
	“healthcare expenditure”	
	“healthcare savings”	
	“healthcare seeking behavior”	
	“healthcare seeking behaviour”	
	“healthcare service costs”	
	“healthcare service delivery”	
	“healthcare service use”	
	“healthcare service utilisation”	
	“healthcare service utilization”	
	“healthcare services delivery”	
	“healthcare services utilisation”	
	“healthcare services utilization”	
	“healthcare spending”	
	“healthcare use”	
	“healthcare utilisation”	
	“healthcare utilization”	
	“inpatient care”	
	“medical care delivery”	
	“medical care seeking behavior”	
	“medical care seeking behaviour”	
	“medical care use”	
	“medical care utilisation”	
	“medical care utilization”	
	“medicine delivery”	
	“medicine use”	
	“medicine utilisation”	
	“medicine utilization”	
	“medical care cost”	
	“medical care costs”	
	“medical care expenditure”	
	“medical care savings”	
	“medical care spending”	
	“medication cost”	
	“medication costs”	
	“medication delivery”	
	“medication expenditure”	
	“medication savings”	
	“medication seeking behavior”	
	“medication spending”	
	“medication use”	
	“medication utilisation”	
	“medication utilization”	
	“medicine cost”	
	“medicine costs”	
	“medicine expenditure”	
	“outpatient care”	
	“resource cost”	
	“resource costs”	
	“resource delivery”	
	“resource expenditure”	
	“resource saving”	
	“resource savings”	
	“resource spending”	
	“resource use”	
	“resource utilisation”	
	“resource utilization”	
	“resources costs”	
	“resources expenditure”	
	“resources saving”	
	“resources savings”	
	“resources use”	
	“resources utilisation”	
	“resources utilization”	
	“self-medication”	
	“shopping behavior”	
	“shopping behaviour”	
	“use of drugs”	
	“use of health care”	
	“use of health care services”	
	“use of health service”	
	“use of health services”	
	“use of healthcare”	
	“use of healthcare services”	
	“use of medicine”	
	“use of resources”	
	“utilisation of health services”	
	“utilization of health care”	
	“utilization of health service”	
	“utilization of health services”	
	“utilization of healthcare”	
	“utilization of healthcare services”	
	“utilization of resources”	
*	((((((((((((((((((((((((((((((((((((((((((((((((((((((((((((((((((((((((((((((((((((((((((((((((((((((((((((((((((((((((((((((((((((((((((((((((((((“health care costs” [MeSH terms]) OR “delivery of health care/utilization” [MeSH terms]) OR “health care seeking behaviour”) OR “health care seeking behavior”) OR “delivery of health care”) OR “use of health care”) OR “utilization of health care”) OR “health care delivery”) OR “health care use”) OR “health care utilisation”) OR “health care utilization”) OR “healthcare seeking behaviour”) OR “healthcare seeking behavior”) OR “delivery of healthcare”) OR “use of healthcare”) OR “utilization of healthcare”) OR “healthcare delivery”) OR “healthcare use”) OR “healthcare utilisation”) OR “healthcare utilization”) OR “use of health service”) OR “utilization of health service”) OR “health service delivery”) OR “health service use”) OR “health service utilisation”) OR “health service utilization”) OR “medical care seeking behaviour”) OR “medical care seeking behavior”) OR “medical care delivery”) OR “medical care use”) OR “medical care utilisation”) OR “medical care utilization”) OR “use of healthcare services”) OR “utilization of healthcare services”) OR “healthcare services delivery”) OR “healthcare services utilisation”) OR “healthcare services utilization”) OR “healthcare service delivery”) OR “healthcare service use”) OR “healthcare service utilisation”) OR “healthcare service utilization”) OR “delivery of health services”) OR “use of health services”) OR “utilisation of health services”) OR “utilization of health services”) OR “health services delivery”) OR “health services use”) OR “health services utilisation”) OR “health services utilization”) OR “use of medicine”) OR “medicine delivery”) OR “medicine use”) OR “medicine utilisation”) OR “medicine utilization”) OR “health care service seeking behavior”) OR “health care service delivery”) OR “health care service use”) OR “health care service utilisation”) OR “health care service utilization”) OR “use of health care services”) OR “health care services delivery”) OR “health care services utilisation”) OR “health care services utilization”) OR “resource delivery”) OR “resource use”) OR “resource utilisation”) OR “resource utilization”) OR “medication seeking behavior”) OR “medication delivery”) OR “medication use”) OR “medication utilisation”) OR “medication utilization”) OR “ambulatory care delivery”) OR “ambulatory care use”) OR “ambulatory care utilization”) OR “use of resources”) OR “utilization of resources”) OR “resources use”) OR “resources utilisation”) OR “resources utilization”) OR “health services cost”) OR “health services expenditure”) OR “health service savings”) OR “health service costs”) OR “health service cost”) OR “health service expenditure”) OR “health service spending”) OR “medical care savings”) OR “medical care costs”) OR “medical care cost”) OR “medical care expenditure”) OR “medical care spending”) OR “cost of health care”) OR “cost of healthcare”) OR “health care savings”) OR “health care costs”) OR “health care cost”) OR “health care expenditure”) OR “health care spending”) OR “healthcare savings”) OR “healthcare costs”) OR “healthcare cost”) OR “healthcare expenditure”) OR “healthcare spending”) OR “health care service costs”) OR “healthcare service costs”) OR “self-medication”) OR “health seeking behaviour”) OR “health seeking behavior”) OR “ambulatory care costs”) OR “ambulatory care cost”) OR “ambulatory care expenditure”) OR “resources savings”) OR “resources saving”) OR “resources costs”) OR “resources expenditure”) OR “resource savings”) OR “resource saving”) OR “resource costs”) OR “resource cost”) OR “resource expenditure”) OR “resource spending”) OR “medication savings”) OR “medication costs”) OR “medication cost”) OR “medication expenditure”) OR “medication spending”) OR “medicine costs”) OR “medicine cost”) OR “medicine expenditure”) OR “drug use”) OR “drug utilization”) OR “drug utilisation”) OR “use of drugs”) OR “drug delivery”) OR “delivery of drugs”) OR “drug cost”) OR “drug costs”) OR “cost of drugs”) OR “drug spending”) OR “drug expenditure”) OR “inpatient care”) OR “outpatient care”) OR “continuity of care”) OR “care trajectory”) OR “care trajectories”) OR “doctor shopping”) OR “shopping behavior”) OR “shopping behaviour”	407,551 (27 April 2018)
**Outcome 1 AND Outcome 2**		
*	((((((((((((((((((((((((((((((((((((((((((((((((((((((((((((“anxiety” [MeSH terms]) OR “pain perception” [MeSH terms]) OR “catastrophization” [MeSH terms]) OR “pain attention”) OR “attention to pain”) OR “pain awareness”) OR “vigilance to pain”) OR “pain vigilant”) OR “hypervigilance”) OR “pain vigilance”) OR “catastrophisation”) OR “catastrophization”) OR “catastrophising”) OR “catastrophizing”) OR “catastrophic thinking”) OR “pain catastrophisation”) OR “pain catastrophization”) OR “pain catastrophising”) OR “pain catastrophizing”) OR “fear of movement”) OR “kinesiophobia”) OR “Tampa scale”) OR “illness cognitions”) OR “illness cognition”) OR “illness belief”) OR “illness beliefs”) OR “illness perception”) OR “illness perceptions”) OR “anxiety”) OR “fear of pain”) OR “psychosocial”) OR “attitude”) OR “pain belief”) OR “pain beliefs”) OR “pain perception”) OR “pain perceptions”) OR “pain cognition”) OR “pain cognitions”) OR “pain thoughts”) OR “self-efficacy”) OR “attribution”) OR “attributions”) OR “resilience”) OR “mindset”) OR “mind set”) OR “acceptance”) OR “self-compassion”) OR “anger”) OR “rumination”) OR “perceived injustice”) OR “depressive thoughts”) OR “mental stress”) OR “psychological stress”) OR “emotional stress”) OR “pain-related stress”) OR “somatization”) OR “somatisation”) OR “depressive symptoms”))) AND (((((((((((((((((((((((((((((((((((((((((((((((((((((((((((((((((((((((((((((((((((((((((((((((((((((((((((((((((((((((((((((((((((((((((((((((((((((“health care costs” [MeSH terms]) OR “delivery of health care/utilization” [MeSH terms]) OR “health care seeking behaviour”) OR “health care seeking behavior”) OR “delivery of health care”) OR “use of health care”) OR “utilization of health care”) OR “health care delivery”) OR “health care use”) OR “health care utilisation”) OR “health care utilization”) OR “healthcare seeking behaviour”) OR “healthcare seeking behavior”) OR “delivery of healthcare”) OR “use of healthcare”) OR “utilization of healthcare”) OR “healthcare delivery”) OR “healthcare use”) OR “healthcare utilisation”) OR “healthcare utilization”) OR “use of health service”) OR “utilization of health service”) OR “health service delivery”) OR “health service use”) OR “health service utilisation”) OR “health service utilization”) OR “medical care seeking behaviour”) OR “medical care seeking behavior”) OR “medical care delivery”) OR “medical care use”) OR “medical care utilisation”) OR “medical care utilization”) OR “use of healthcare services”) OR “utilization of healthcare services”) OR “healthcare services delivery”) OR “healthcare services utilisation”) OR “healthcare services utilization”) OR “healthcare service delivery”) OR “healthcare service use”) OR “healthcare service utilisation”) OR “healthcare service utilization”) OR “delivery of health services”) OR “use of health services”) OR “utilisation of health services”) OR “utilization of health services”) OR “health services delivery”) OR “health services use”) OR “health services utilisation”) OR “health services utilization”) OR “use of medicine”) OR “medicine delivery”) OR “medicine use”) OR “medicine utilisation”) OR “medicine utilization”) OR “health care service seeking behavior”) OR “health care service delivery”) OR “health care service use”) OR “health care service utilisation”) OR “health care service utilization”) OR “use of health care services”) OR “health care services delivery”) OR “health care services utilisation”) OR “health care services utilization”) OR “resource delivery”) OR “resource use”) OR “resource utilisation”) OR “resource utilization”) OR “medication seeking behavior”) OR “medication delivery”) OR “medication use”) OR “medication utilisation”) OR “medication utilization”) OR “ambulatory care delivery”) OR “ambulatory care use”) OR “ambulatory care utilization”) OR “use of resources”) OR “utilization of resources”) OR “resources use”) OR “resources utilisation”) OR “resources utilization”) OR “health services cost”) OR “health services expenditure”) OR “health service savings”) OR “health service costs”) OR “health service cost”) OR “health service expenditure”) OR “health service spending”) OR “medical care savings”) OR “medical care costs”) OR “medical care cost”) OR “medical care expenditure”) OR “medical care spending”) OR “cost of health care”) OR “cost of healthcare”) OR “health care savings”) OR “health care costs”) OR “health care cost”) OR “health care expenditure”) OR “health care spending”) OR “healthcare savings”) OR “healthcare costs”) OR “healthcare cost”) OR “healthcare expenditure”) OR “healthcare spending”) OR “health care service costs”) OR “healthcare service costs”) OR “self medication”) OR “health seeking behaviour”) OR “health seeking behavior”) OR “ambulatory care costs”) OR “ambulatory care cost”) OR “ambulatory care expenditure”) OR “resources savings”) OR “resources saving”) OR “resources costs”) OR “resources expenditure”) OR “resource savings”) OR “resource saving”) OR “resource costs”) OR “resource cost”) OR “resource expenditure”) OR “resource spending”) OR “medication savings”) OR “medication costs”) OR “medication cost”) OR “medication expenditure”) OR “medication spending”) OR “medicine costs”) OR “medicine cost”) OR “medicine expenditure”) OR “drug use”) OR “drug utilization”) OR “drug utilisation”) OR “use of drugs”) OR “drug delivery”) OR “delivery of drugs”) OR “drug cost”) OR “drug costs”) OR “cost of drugs”) OR “drug spending”) OR “drug expenditure”) OR “inpatient care”) OR “outpatient care”) OR “continuity of care”) OR “care trajectory”) OR “care trajectories”) OR “doctor shopping”) OR “shopping behavior”) OR “shopping behaviour”)	35,152 (27 April 2018)
**Population AND Outcome 1 AND Outcome 2**		
*	((((((((((((((((((((((((((((((((((((((((((((((((((((((((((((((“anxiety” [MeSH terms]) OR “pain perception” [MeSH terms]) OR “catastrophization” [MeSH terms]) OR “pain attention”) OR “attention to pain”) OR “pain awareness”) OR “vigilance to pain”) OR “pain vigilant”) OR “hypervigilance”) OR “pain vigilance”) OR “catastrophisation”) OR “catastrophization”) OR “catastrophising”) OR “catastrophizing”) OR “catastrophic thinking”) OR “pain catastrophisation”) OR “pain catastrophization”) OR “pain catastrophising”) OR “pain catastrophizing”) OR “fear of movement”) OR “kinesiophobia”) OR “Tampa scale”) OR “illness cognitions”) OR “illness cognition”) OR “illness belief”) OR “illness beliefs”) OR “illness perception”) OR “illness perceptions”) OR “anxiety”) OR “fear of pain”) OR “psychosocial”) OR “attitude”) OR “pain belief”) OR “pain beliefs”) OR “pain perception”) OR “pain perceptions”) OR “pain cognition”) OR “pain cognitions”) OR “pain thoughts”) OR “self-efficacy”) OR “attribution”) OR “attributions”) OR “resilience”) OR “mindset”) OR “mind set”) OR “acceptance”) OR “self-compassion”) OR “anger”) OR “rumination”) OR “perceived injustice”) OR “depressive thoughts”) OR “mental stress”) OR “psychological stress”) OR “emotional stress”) OR “pain-related stress”) OR “somatization”) OR “somatisation”) OR “depressive symptoms”))) AND (((((((((((((((((((((((((((((((((((((((((((((((((((((((((((((((((((((((((((((((((((((((((((((((((((((((((((((((((((((((((((((((((((((((((((((((((((((“health care costs” [MeSH terms]) OR “delivery of health care/utilization” [MeSH terms]) OR “health care seeking behaviour”) OR “health care seeking behavior”) OR “delivery of health care”) OR “use of health care”) OR “utilization of health care”) OR “health care delivery”) OR “health care use”) OR “health care utilisation”) OR “health care utilization”) OR “healthcare seeking behaviour”) OR “healthcare seeking behavior”) OR “delivery of healthcare”) OR “use of healthcare”) OR “utilization of healthcare”) OR “healthcare delivery”) OR “healthcare use”) OR “healthcare utilisation”) OR “healthcare utilization”) OR “use of health service”) OR “utilization of health service”) OR “health service delivery”) OR “health service use”) OR “health service utilisation”) OR “health service utilization”) OR “medical care seeking behaviour”) OR “medical care seeking behavior”) OR “medical care delivery”) OR “medical care use”) OR “medical care utilisation”) OR “medical care utilization”) OR “use of healthcare services”) OR “utilization of healthcare services”) OR “healthcare services delivery”) OR “healthcare services utilisation”) OR “healthcare services utilization”) OR “healthcare service delivery”) OR “healthcare service use”) OR “healthcare service utilisation”) OR “healthcare service utilization”) OR “delivery of health services”) OR “use of health services”) OR “utilisation of health services”) OR “utilization of health services”) OR “health services delivery”) OR “health services use”) OR “health services utilisation”) OR “health services utilization”) OR “use of medicine”) OR “medicine delivery”) OR “medicine use”) OR “medicine utilisation”) OR “medicine utilization”) OR “health care service seeking behavior”) OR “health care service delivery”) OR “health care service use”) OR “health care service utilisation”) OR “health care service utilization”) OR “use of health care services”) OR “health care services delivery”) OR “health care services utilisation”) OR “health care services utilization”) OR “resource delivery”) OR “resource use”) OR “resource utilisation”) OR “resource utilization”) OR “medication seeking behavior”) OR “medication delivery”) OR “medication use”) OR “medication utilisation”) OR “medication utilization”) OR “ambulatory care delivery”) OR “ambulatory care use”) OR “ambulatory care utilization”) OR “use of resources”) OR “utilization of resources”) OR “resources use”) OR “resources utilisation”) OR “resources utilization”) OR “health services cost”) OR “health services expenditure”) OR “health service savings”) OR “health service costs”) OR “health service cost”) OR “health service expenditure”) OR “health service spending”) OR “medical care savings”) OR “medical care costs”) OR “medical care cost”) OR “medical care expenditure”) OR “medical care spending”) OR “cost of health care”) OR “cost of healthcare”) OR “health care savings”) OR “health care costs”) OR “health care cost”) OR “health care expenditure”) OR “health care spending”) OR “healthcare savings”) OR “healthcare costs”) OR “healthcare cost”) OR “healthcare expenditure”) OR “healthcare spending”) OR “health care service costs”) OR “healthcare service costs”) OR “self medication”) OR “health seeking behaviour”) OR “health seeking behavior”) OR “ambulatory care costs”) OR “ambulatory care cost”) OR “ambulatory care expenditure”) OR “resources savings”) OR “resources saving”) OR “resources costs”) OR “resources expenditure”) OR “resource savings”) OR “resource saving”) OR “resource costs”) OR “resource cost”) OR “resource expenditure”) OR “resource spending”) OR “medication savings”) OR “medication costs”) OR “medication cost”) OR “medication expenditure”) OR “medication spending”) OR “medicine costs”) OR “medicine cost”) OR “medicine expenditure”) OR “drug use”) OR “drug utilization”) OR “drug utilisation”) OR “use of drugs”) OR “drug delivery”) OR “delivery of drugs”) OR “drug cost”) OR “drug costs”) OR “cost of drugs”) OR “drug spending”) OR “drug expenditure”) OR “inpatient care”) OR “outpatient care”) OR “continuity of care”) OR “care trajectory”) OR “care trajectories”) OR “doctor shopping”) OR “shopping behavior”) OR “shopping behaviour”))) AND pain	2561 (27 April 2018) 2828 (6 August 2019)

* Terms used in the final systematic search with corresponding number of search results. CEF: cognitive and emotional factors; HCU: healthcare use.

## Appendix B

**Table A2:** Characteristics of the included studies.

Author (Year)	C	D	Sample		Outcome Measures		Investigated Associations and Statistics ^4^
			Condition Duration of Pain	n Sex (%♂/%♀) Age (Mean ± SD) ^1^	CEF Time of Assessment ^2^ *CEF Cluster* Outcome Measure	HCU Type of Data Collection ^3^ (Considered Period ^2^) Content *(HCU Category)*	
Alschuler (2012) [48]	US	CS	Multiple sclerosis with pain Mean pain duration: 137.68 m	161 17/83 54.02 ± 11.86 y	*Depressive symptoms* → Patient Health Questionnaire-9 (PHQ-9) → Dichotomized for comparative analyses: - PHQ-9 ≥ 10: clinically relevant depressive symptoms - PHQ-9 < 10: no depressive symptoms	Retrospective (past 6 m + current) Types of pain treatments used → yes/no for each: - PT *(type; primary care consultations)* - Nerve blocks *(type; invasive procedures)* - Biofeedback/relaxation *(type; CAM use)* - Acupuncture *(type; CAM use)* - Magnets *(type; CAM use)* - Massage *(type; CAM use)* - Hypnosis *(type; CAM use)* - Counseling/psychotherapy *(type; primary care consultations)* - Mexiletine *(type; prescription pain medication)* - Neurontin *(type; prescription pain medication)* - TCA *(type; prescription pain medication)* - Narcotics *(type; prescription pain medication)* - Acetaminophen *(type; OTC pain medication)* - Advil/Aspirin/Aleve *(type; OTC pain medication)* - Diazepam/Alprazolam *(type; prescription pain medication)* - Tegretol *(type; prescription pain medication)* - Baclofen *(type; prescription pain medication)* - TENS unit *(type; CAM use)* - Dilantin or other anticonvulsant *(type; prescription pain medication)* - Chiropractic adjustment *(type; CAM use)* - Heat *(type; CAM use)* - Ice *(type; CAM use)* - Marijuana *(type; prescription pain medication)* - Strengthening exercises *(type; CAM use)* - Mobility exercises or ROM *(type; CAM use)* - Implanted nerve stimulator *(type; invasive procedures)* - Implanted medication pump *(type; invasive procedures)* → Frequency of use of the former pain treatments was also assessed to calculate the total amount of pain treatments used. *(amount; HCU in general)* Number of visits w/ healthcare providers for pain: - Primary care providers - MS specialists - Other physicians - PT/OT - Other providers *(all above: amount; consultations)* - Chiropractors *(amount; CAM use)* - ER *(amount; emergency HCU)* Total number of visits and total number of visits w/o PT/OT visits was also calculated. *(amount; consultations)*	To compare current and past use of the listed pain treatments between patients w/ and w/o depressive symptoms. → Chi^2^
							To investigate whether depressive symptoms were influencing the total number of pain treatments currently used and used in the past. → Regression
							Idem, but controlling for pain intensity. → Regression
							To comparw/ and w/o cle the number of visits w/ the following healthcare providers between patients inically relevant depressive symptoms: - PT/OT - Primary care providers - MS specialists - Other MDs - Chiropractors - ER - Other providers - Total amount of healthcare visits w/o PT/OT visits → *t*-test
							To investigate whether depressive symptoms were influencing the total number of healthcare visits. → Regression
							Idem, but controlling for pain intensity. → Multivariate regression
Asmundson (2001) ^5^ [49]	US	CS	Chronic recurring headache Mean duration of pain: 205.6 ± 156.7 m Range: 1–600 m	108 12/88 42.3 ± 12.0 y	*Anger* → State-Trait Anger Expression Inventory - Trait form for anger *General anxiety symptoms* → State-Trait Anxiety Inventory - Trait form *Symptom-related anxiety symptoms* → Pain Anxiety Symptom Scale - Pain-specific cognitive anxiety - physiological anxiety - fearful appraisals of pain subscales *Depressive symptoms* → Beck Depression Inventory	Patient-reported Current use Headache Questionnaire: - Current OTC headache medication use *(type; OTC pain medication use)* - Current prescription headache medication use *(type; prescription pain medication use)* → Both yes/no	To investigate associations between the listed CEF and the use of prescription and OTC pain medication use for headache. → Correlations
							To investigate whether the listed CEF ** were significantly influencing the likelihood of using prescription and OTC pain medication use for headache while also accounting for pain severity * and anxiety sensitivity **. → Hierarchical multiple regression * Fixed factor in model ** Omitted from final model if not significant
Biggs (2003) [50]	UK	CS	Upper abdominal or chest pain: (1) functional dyspepsia; (2) noncardiac chest pain; (3) GERD; (4) IHD; or (5) a combination of these. Median duration of symptoms: 16 m (IQR: 7–36 m)	151 47/53 Range: 18–75 y	*General anxiety symptoms* → Hospital Anxiety and Depression Scale - Anxiety subscale *Symptom-related anxiety symptoms* → Health Anxiety Questionnaire *Depressive symptoms* → Hospital Anxiety and Depression Scale - Depression subscale *Negative consequences beliefs* → Illness Perception Questionnaire - Consequences subscale *Negative illness beliefs* → Illness Perception Questionnaire - Timeline subscale *Psychological distress* → Short Form Health Survey (SF-36) - Mental health subscale *Perceived symptom control* → Illness Perception Questionnaire - Cure subscale	Database extraction Retrospective (12 m before and 6 m after index visit) Number of consultations w/ - Healthcare providers in general in the 18 m period - GP - Other providers than GP *(all: amount; consultations)*	To investigate whether the listed CEF were influencing the total number of consultations w/ healthcare providers, number of GP visits and number of consultations w/ other providers than GP while also accounting for sex, marital status, education, access to confidant, diagnosis, pain score, remaining 7 SF-36 scores, recent social stress, exposure to death of a family member (father, mother or sibling) during childhood and reported childhood adversity (antipathy from father or mother, neglect and physical, psychological or sexual abuse). → Hierarchical stepwise multiple regression Independent variables were omitted from the final model if not significant.
Boyer (2009) [51]	US	C	Fibromyalgia Mean pain duration: Primary care: 9.77 ± 10.22 y Rheumatology setting: 12.93 ± 1.10 y	315 0/100 Primary care (n = 101): 49.8 ± 10.39 y Rheumatology setting (n = 214): 52.7 ± 9.01 y	*General anxiety symptoms* → Hospital Anxiety and Depression Scale - Anxiety subscale *Depressive symptoms* → Hospital Anxiety and Depression Scale - Depression subscale *Self-efficacy beliefs* → Chronic Pain Self-Efficacy Scale - Pain management - symptoms management - physical functioning subscales *Locus of control* → Multidimensional Pain Locus of Control Scale - Internal, fate and chance subscales	Database extraction Patients were recruited from a rheumatology setting or a primary care setting. → Binary variable *(type; secondary care consultations)*	To compare the listed CEF between patients attending either a rheumatology setting or primary care. → *t*-tests
Buse (2012) [94]	US	C	Migraine	5796 19/81 Nonusers (n = 4076): 50.7 ± 12.5 y Previous users (n = 798): 53.0 ± 12.5 y Current nondependent users (n = 769): 53.6 ± 11.3 y Current probable dependence (n = 153): 53.1 ± 12.4 y	*General anxiety symptoms* → General Anxiety Disorder-7 → Dichotomized for comparative analyses: - Clinically relevant anxiety symptoms - No anxiety symptoms → DSM-IV clinical algorithm *Depressive symptoms* → Patient Health Questionnaire-9 (PHQ-9) → Dichotomized for comparative analyses: - PHQ-9 ≥ 10: clinically relevant depressive symptoms - PHQ-9 < 10: no depressive symptoms	Patient-reported Retrospective (yearly survey over a period of 3 y) Frequency of opioid use + risk of dependency questionnaire based on DSM-IV criteria → sample divided in 4 groups based on type of opioid user: - Non-users (reference) - Previous users - Current non-dependent users - Current probable dependent users *(type; opioid use)*	To investigate whether presence of depressive and anxiety symptoms (reference: no symptoms) is influencing the likelihood of being a previous, current non-dependent or current dependent opioid user (reference: non-user). → Logistic regressions
Carroll (2016) ^5^ [96]	US	C	Sickle cell disease	83 31/69 Chronic opioid therapy (n = 29): 40.6 ± 11.7 y No chronic opioids (n = 54): 38.0 ± 12.4 y	*Depressive symptoms* → Center for Epidemiologic Studies Depression Scale	Database review Current situation Being on chronic opioid therapy → yes/no *(type; opioid use)*	To compare level of depressive symptoms between patients on chronic opioid therapy and those who are not. → ANOVA
						Patient-reported Daily diary - Days w/ calls to healthcare providers - Days w/ medical visits *(Both: amount; consultations)*	To investigate whether level of depressive symptoms is a significant covariate in the relationship between being on chronic opioid therapy (reference: not on chronic opioid therapy) and days w/ calls to providers and w/ medical visits. → Regression
Carroll (2018) ^5^ [95]	US	C	Sickle cell disease (SCD)	73 39.36/61.64 34.43 ± 9.70 y	Assessed at baseline *Symptom-related anxiety symptoms* → Pain Anxiety Symptoms Scale	Database review Prospective (1 y) - Use of the Sickle Cell Infusion Center → sample divided into: - Non-utilizers - Typical utilizers (median or less) - High utilizers (above median) *(amount; consultations)* - Opioid dose (converted to oral morphine equivalents) *(amount; pain medication use)*	To compare pain anxiety scores between the 3 listed sickle cell infusion center use groups. → ANOVA
							To investigate whether baseline pain anxiety score was influencing frequency of SCD Infusion Center use while also accounting for demographics (age and sex), disease-related variables (genotype, hemoglobin, acute chest, avascular necrosis, prior hydroxyurea, chronic transfusion and total daily opioid), socioeconomic status and psychiatric variables (family history, psychiatric treatment and substance use family). → Negative binomial generalized linear model
							To investigate whether baseline pain anxiety score was influencing within-visit acute opioid dose while also accounting for demographics (age and sex), disease-related variables (genotype, hemoglobin, acute chest, avascular necrosis, prior hydroxyurea, chronic transfusion, total daily opioid and utilization), socioeconomic status and psychiatric variables (family history, psychiatric treatment and substance use family). → Linear mixed models
Ciechanowski (2003) [25]	US	C	Patients with chronic pain participating in a multidisciplinary pain program Mean pain duration: 6.3 ± 7.8 y	111 45/55 44.7 ± 10.7 y	Assessed at baseline *Catastrophizing* → Coping Strategies Questionnaire - Catastrophizing subscale *Depressive symptoms* → Center for Epidemiologic Studies Depression Scale	Patient-reported Retrospective (past 3 m; assessed at 12 m follow-up of multidisciplinary program) Frequency of pain-related visits → Subdivided in: - ≥monthly - ≥weekly - <weekly *(amount; consultations)*	To investigate whether the listed CEF were influencing the likelihood of greater than monthly (reference: greater than weekly) and greater than weekly (reference: less) pain-related visits while also accounting for age, gender, baseline pain-related HCU and attachment style. → Logistic regression
Citero (2007) [97]	US	C	Sickle cell disease	220 38.6/61.4 34 ± 11.4 y	Assessed at baseline *Catastrophizing* → Coping Strategy Questionnaire - Catastrophizing subscale	Patient-reported Prospective (daily diaries for up to 6 m) Number of pain-related: - Unscheduled doctor visits *(amount; emergency HCU)* - ER visits *(amount; emergency HCU)* - Hospitalizations *(amount; hospitalizations)* → analyzed both separately and combined all together *(amount; HCU in general)*	To investigate whether baseline catastrophizing was influencing the following HCU outcomes during the upcoming 6 m on crisis days and non-crisis days: - unscheduled doctor visits - ER visits - hospitalizations - all 3 above combined → Simple linear regression
							Idem, but controlling for depression. → Linear regression
Cronan (2002) ^5^ [135]	US	C	Fibromyalgia patients participating in an intervention study	600 4.7/95.3 53.92 ± 11.45 y	Assessed at baseline *Depressive symptoms* → Center for Epidemiologic Studies Depression Scale *Helplessness* → Arthritis Helplessness Index *Self-efficacy beliefs* → Arthritis Self-Efficacy Scale	Database extraction Retrospective (1 y before and 1 y after study initiation) Number and type of contacts and prescribed medical tests and medication → Combined into 1 HCU outcome for the year before and after study initiation *(amount; HCU in general)*	To investigate the association between the listed baseline CEF and the total amount of HCU 1 y before and after study initiation. → Correlations
							To investigate whether the listed baseline CEF were influencing total HCU 1 y after study initiation, while also accounting for baseline health status, ethnicity, comorbidity, education, income, age, employment, social support, baseline HCU and coping. → Hierarchical regression
Cronin (2018) ^5^ [93]	US	CS	Sickle cell disease	67 46.3/53.7 27.0 y (Range: 18–61 y)	*Self-efficacy beliefs* → Sickle Cell Self-Efficacy Scale	Database extraction Retrospective (1 y) Number of acute ER visits and hospitalizations for vaso-occlusive pain episodes → Combined in one variable for emergency HCU *(amount; emergency HCU)*	To investigate whether self-efficacy was significantly predicting amount of emergency HCU while also accounting for age, sex, SCD phenotypes, disease-modifying therapy and Patient Activation Measure. → Negative binomial regression
Cronin (2019) [52]	US	CS	Sickle cell disease	201 42.3/57.3 26.0 y (Range: 22.0–35.0 y)	*Depressive symptoms* → Patient Health Questionnaire-2	Patient-reported Retrospective (1 y) - Hospitalizations *(type; hospitalizations)* - Readmissions (being hospitalized twice in a 30-day period) *(amount; hospitalizations)* → Both yes/no	To investigate whether level of depressive symptoms was influencing the likelihood of having a hospital admission (reference: no hospital admission) and being readmitted to the hospital (reference: no readmission) while also accounting for age, sex, education, ability to pay bills, literacy, spirituality and social support. → Logistic regressions
Daltroy (1998) ^5^ [133]	US	RCT	Patients scheduled for total knee or hip arthroplasty participating in an intervention study delivering education and relaxation interventions.	222 34/66 64 ± 12 y 73% osteoarthritis 19% rheumatoid arthritis 8% other	Measured at baseline (preoperative) *General anxiety symptoms* State anxiety → State-Trait Anxiety Inventory - State form *Perceived symptom control* → self-designed question (a lot; moderate; a little; none)	Database extraction Retrospective (4 d post-surgery) - Length of stay *(amount; hospitalizations)* - Postoperative pain medication use *(amount; pain medication use)*	To investigate whether the listed preoperative CEF were influencing postoperative length of stay and pain medication use while also accounting for age, sex, reliance in God, surgeon, date of surgery, comorbidities, cemented joint, desire for information, passive range of motion, lack of a discharge plan, denial, perceived pain control and provision of information and relaxation training. → General linear models Independent variables were omitted from final model if not contributing significantly to the model.
de Boer (2012) [53]	NL	CS	Patients attending a pain center Pain duration: <3 m: 34.1% 3–6 m: 2.4% >6 m: 63.5% Community sample w/ pain Pain duration: <3 m: 4.7% 3–6 m: 4% >6 m: 91.3%	Pain center patients: 150 40.7/59.3 50.6 ± 15.4 y Community sample w/ pain: 137 65/35 53.2 ± 13.5 y	*Catastrophizing* → Pain Catastrophizing Scale	Patient-reported Retrospective (the past in general) - Specialist consultations *(type; secondary care consultations)* - Pain medication use *(type; pain medication use)* → Both yes/no	To investigate whether level of catastrophizing was influencing the likelihood of having specialist consultations (reference: no consultation) and using pain medication (reference: no use) while also accounting for age, sex and pain intensity in the pain center patients and community sample w/ pain separately. → Hierarchical logistic regression
Demmelmaier (2010) [98]	SE	C	Back pain Pain duration: <3 m: 42 >12 m: 271	First-episode group (pain < 3 m): 42 Long-duration group (pain >12 m): 271	Measured at baseline *General anxiety symptoms* → Hospital Anxiety and Depression Scale - Anxiety subscale *Catastrophizing* → Coping Strategy Questionnaire - Catastrophizing Subscale *Depressive symptoms* → Hospital Anxiety and Depression Scale – Depression subscale *Fear-avoidance beliefs* Fear of movement and/or (re)injury → Tampa Scale for Kinesiophobia-2 *Symptom vigilance* → Pain Vigilance and Awareness Questionnaire *Self-efficacy beliefs* - Functional self-efficacy → Self-Efficacy Scale - Self-efficacy for exercise → Self-Efficacy Scale for Exercise	Patient-reported Retrospective (past 3 m; measured at 12 m follow-up) Number of consultations w/6 different healthcare providers *(amount; consultations)*	To investigate associations between the listed CEF at baseline and number of healthcare visits 12 m later in patients of the first-episode and long-duration groups separately. → Correlations
							To investigate whether the listed baseline CEF are significant predictors of the number of healthcare visits 12 m later in patients of the first-episode and long-duration groups separately. → Simple linear regression Regression was only performed for variables showing a significant correlation. If simple linear regression was performed, then this was included in the review instead of the correlation analysis.
Dobkin (2006) [99]	CA	C	Fibromyalgia Median disease duration: 32 m (IQR: 8.8–72)	Tertiary care: 60 Community: 82 Total sample: 142 0/100 50.9 ± 10.2 y	Measured at baseline: *Psychological distress* → Symptom Checklist 90-R	Patient-reported Retrospective Attending tertiary care vs. community patients *(type; tertiary care consultations)*	To compare levels of psychological distress between patients from the tertiary care and community samples. → *t*-test
Durá-Ferrandis (2017) [134]	ES	RCT	TMD Participating in CBT intervention study	72 Experimental group: 41 13/87 39.57 ± 13.82 y Control group: 29 9/91 38.38 ± 16.57 y	Assessed pre- and post-treatment (3 m after baseline) *Catastrophizing* → Pain Catastrophizing Scale *Psychological distress* → Brief Symptoms Inventory-18 *Perceived symptom control* → Survey of Pain Attitudes-35 - Perceived control subscale	Patient-reported Retrospective (past 2 m; measured pre-treatment and post-treatment (3 m after baseline)) Frequency of self-medication: number of days on which the patient voluntarily took medication to manage pain symptoms. *(amount; pain medication use)*	To investigate whether the listed CEF were significant mediators of the treatment effect on frequency of self-medication, meaning that the relationship between changes in CEF and treatment outcome was investigated, while also accounting for pain intensity change and coping strategies. → Structural equation modelling
Elander (2003) [54]	UK	CS	Hemophilia	68 41 ± 14 y	*Catastrophizing* → Hemophilia-Adapted Coping Strategies Questionnaire - Negative thoughts subscale	Database extraction Comprehensive care center use vs. another hemophilia center *(type; secondary care consultations)*	To compare level of negative thoughts about pain between patients attending a comprehensive care center vs. another hemophilia center. → Fisher’s Exact test
						Patient-reported Retrospective (last month) Number of: - Days when OTC pain medication was used *(amount; pain medication use)* - Days when prescription pain medication was used *(amount; pain medication use)* - Healthcare visits *(amount; consultations)*	To investigate correlations between level of negative thoughts about pain and amount of: - OTC pain medication use - Prescription pain medication use - Healthcare visits → Correlations
Elander (2014) [55]	UK	CS	General adult population w/ pain and using OTC or prescription painkillers in the last month	112 18/82 44.5 ± 13.5 y	*General anxiety symptoms* → Depression, Anxiety and Stress Scale-21 - Anxiety subscale *Symptom-related anxiety symptoms* → Pain Anxiety Symptoms Scale *Catastrophizing* → Pain Catastrophizing Scale *Depressive symptoms* → Depression, Anxiety and Stress Scale-21 - Depression subscale *Self-compassion* → Self-Compassion Scale-Short Form *Stress* → Depression, Anxiety and Stress Scale-21 - Stress subscale *Pain acceptance* → Chronic Pain Acceptance Questionnaire *Self-efficacy beliefs* → Pain Self-Efficacy Questionnaire	Patient-reported Retrospective (last month) Frequency of OTC and prescription pain medication use → 5-point scales: once or twice; about once a week; more than once a week; almost every day; every day *(Both: amount; pain medication use)*	To investigate associations between the listed CEF and OTC and prescription pain medication use. → Pearson correlations
Engel (1996) ^5^ [100]	US	C	Patients w/ spinal pain having a primary care back pain visit	1059 47.2/52.8 18–44 y: 48.3% 45–64 y: 35.9% 65–74 y: 15.8%	Measured 1 m after index visit *Depressive symptoms* → Symptom Checklist-90 - Depression subscale → Categorized into: - ≤1.0 - 1.01–1.6 - >1.6	Database extraction Prospective (until 12 m after index visit) Amount of use of healthcare services for back pain (listed below), categorized into the following categories: - ≥2 primary care visits vs. <2 *(amount; consultations)* - ≥2 radiologic procedures vs. <2 *(amount; consultations)* - ≥1 specialist visit vs. <1 *(type; secondary care consultations)* - ≥1 admission vs. <1 *(type; hospitalizations)* - ≥8 pain medication fills vs. <8 *(amount; pain medication use)*	To investigate whether the presence of depressive symptoms was influencing use of the listed healthcare services. → Univariate logistic regressions
							Idem, but also accounting for age, gender, education, chronic pain grade, days in pain, disability pay and diagnosis. → Multivariate logistic regression
Fink-Miller (2014) ^5^ [56]	US	CS	Chronic non-cancer pain Pain duration: >6 m	233 49/51 49 ± 11.55 y	*Catastrophizing* → Pain Catastrophizing Scale *Depressive symptoms* → Beck Depression Inventory II	Database extraction Attending primary vs. tertiary care *(type; tertiary care consultations)*	To compare catastrophizing and depressive symptoms scores between primary and tertiary care patients. → Wilcoxon rank sum test
							To investigate the influence of attending tertiary care (reference: primary care) on level of catastrophizing and depressive symptoms while adjusting for age. → Linear regression Regression was only performed for significant outcomes in comparative analysis.
Gebauer (2019) ^5^ [101]	US	C	Chronic non-cancer low back pain Mean pain duration: 13.9 ± 13.6 y	327 26.6/73.4 18–45 y: 23.2% 46–59 y: 43.7% ≥60 y: 33.0%	Assessed at baseline, 12 m and 24 m follow-up *General anxiety symptoms* → Self-designed question: feeling anxious on several or more days in the past 30 d or having a panic attack in the past 2 w → yes/no *Depressive symptoms* → Patient Health Questionnaire-2 → Dichotomized: - PHQ-2 ≥ 3: clinically relevant depressive symptoms - PHQ-2 < 3: no depressive symptoms	Database extraction Retrospective at 12 m and 24 m follow-up for the past 12 m Opioid prescription: Morphine Equivalent Dose (MED) was calculated from the daily dose of 9 possible opioids: codeine, fentanyl, hydrocodone, hydromorphone, meperidine, methadone, morphine, oxycodone and propoxyphene. → Categorized as: - none - 1–50 mg/day MED - >50 mg/day MED *(type; opioid use)*	To investigate whether presence of depressive and anxiety symptoms (reference: no symptoms for both) were influencing the likelihood of using 1–50 mg/day MED opioids and >50 mg/day MED opioids (reference: no opioid use for both) while also accounting for moment of assessment, collecting disability, age, race, sex, education, pain severity, pain duration, health-related quality of life (pain interference, physical functioning, role physical and general health), overweight/obesity, other treatments, having a written pain contract and continuity of care. → Multinomial logistic regressions
Gil (2004) [102]	US	C	Sickle cell disease (SCD)	41 44/56 36.6 ± 13.2 y	Assessed daily *Depressive symptoms* Negative mood → Daily Mood Scale - Negative mood subscale *Stress* → VAS perceived level of overall stress of the day *Positive mood* → Daily Mood Scale - Positive mood subscale	Patient-reported Prospective (daily diaries) Amount of use of the following healthcare services on the same day, the next day and 2 d later: - doctor calls *(amount; consultations)* - hospitalizations *(amount; hospitalizations)* - ER visits *(amount; emergency HCU)* - prescription pain medication use *(amount; pain medication use)*	To investigate whether stress and negative and positive mood were influencing use of the listed healthcare services on the same day, the next day and 2 d later after controlling for level of SCD pain. → Multilevel model regression analyses
Görge (2017) [120]	DE	C	Patients with chronic low back pain who were undergoing multidisciplinary rehabilitation Pain duration: Acute event: 0.6% <1 y: 12.4% 1–2 y: 11.1% 3–5 y: 18.6% 6–10 y: 16.3% >10 y: 40.2%	688 42.8/57.2 51.0 ± 11.2 y	Measured at baseline and at the end of rehabilitation: *Anger* → Pain Coping Questionnaire - Anger subscale *Symptom-related anxiety symptoms* → Pain-Coping Questionnaire - Pain-related anxiety subscale *Depressive symptoms* → Pain Coping Questionnaire - Helplessness & depression subscale Measured at baseline only: *Fear-avoidance beliefs* → Fear-Avoidance Beliefs Questionnaire - Activity beliefs subscale *Negative illness beliefs* → Control Beliefs Concerning Illness and Health Questionnaire - Fatalistic external locus of control subscale	Patient-reported Retrospective (last 6 m; measured at baseline and 6 m after rehabilitation) Frequency of visits w/ - GP *(amount; consultations)* - Specialists *(amount; consultations)* - PT *(amount; consultations)* - Psychotherapy *(amount; consultations)* - Complementary therapist - Massage therapist - Hospital → For the baseline outcome total HCU was calculated. *(amount; HCU in general)* At follow-up visits w/ specific providers were analyzed separately (except for complementary and massage therapists and hospitalizations).	To investigate the influence of baseline helplessness and depression, activity beliefs and fatalistic external locus of control on baseline HCU while also accounting for gender, hours of work and days on sick leave. → Hierarchical regression analysis
							To investigate the influence of baseline anger and anxiety symptoms and change in anxiety symptoms from baseline to post-rehabilitation on the number of follow-up GP visits while also accounting for baseline GP visits hours of work, days on sick leave, state of health, SF-12 physical component score and chronicity and change in coping (experience of competencies) and sick leave. → Hierarchical regression analysis
							To investigate the influence of change in helplessness and depression and anxiety scores on the number of specialist visits post-rehabilitation while also accounting for baseline specialist visits, days on sick leave, state of health and change in sick leave and pain function and disability. → Hierarchical regression analysis
							To investigate the influence of baseline helplessness and depression, activity beliefs and fatalistic external locus of control on the number of PT visits post-rehabilitation while also accounting for baseline PT visits, gender, inability to work, hours of work, days on sick leave and coping (experience of competencies) and change in sick leave. → Hierarchical regression analysis
							To investigate the influence of baseline helplessness and depression and change in anger on the number of psychotherapy visits post-rehabilitation while also accounting for baseline psychotherapy visits, employment, hours of work, days on sick leave and disability. → Hierarchical regression analysis
Grant (2000) [57]	US	CS	Sickle cell disease	43 41.9/58.1 Depressed (n = 11): 34.8 ± 7.5 y Non-depressed (n = 32): 35.1 ± 10.9 y	*Depressive symptoms* → Center for Epidemiologic Studies Depression Scale	Patient-reported Retrospective (last 12 m) Frequency of HCU → Structured Pain Interview; including ER visits, hospitalizations and consultations with healthcare providers *(amount; HCU in general)*	To investigate the relationship between depressive symptoms and frequency of HCU while controlling for age, sex, phenotype and complications. → Hierarchical regression analysis
Hadlandsmyth (2013) [103]	US	C	Non-cardiac chest pain Pain duration: ≤7 d: 15% 7 d–<1 m: 4% 1–6 m: 26% 6 m–1 y: 15% >1 y: 40%	Baseline: 196 43/57 50 ± 11 y Follow-up: 70 47/53 53 ± 12 y	Measured at baseline *General anxiety symptoms* → Depression, Anxiety and Stress Scale - Anxiety subscale *Symptom-related anxiety symptoms* → Albany Panic and Phobia Questionnaire - Interoceptive fear subscale	Patient-reported Retrospective (past year; measured at baseline and 1 y follow-up) Number of caregivers seen and frequency of treatment → Kelner Illness Attitude Scale *(amount; consultations)*	To investigate the correlation between the listed baseline CEF and baseline and follow-up frequency of healthcare visits. → Correlations
							To investigate if the listed baseline CEF were influencing baseline and follow-up frequency of healthcare visits while also accounting for chest pain. → Linear regression Independent variables were only included in the multivariate analysis if significantly correlated w/ HCU in univariate correlation analyses.
Harden (1997) ^5^ [130]	US	CC	Chronic pain Mean pain duration: Opioid group: 60.9 ± 78.1 m Non-opioid group: 51.5 ± 76.1 m	Taking daily opioids: 100 39.4/60.6 45.8 ± 14.2 y Not taking opioids: 100 36/64 44.7 ± 14.1 y	*General anxiety symptoms* → State-Trait Anxiety Inventory - Trait form *Depressive symptoms* → Beck Depression Inventory *Psychological distress* → Multidimensional Pain Inventory - Affective distress subscale	Database extraction Retrospective (period not specified) Taking daily opioids → yes/no *(type; opioid use)*	To compare the listed CEF between patients taking and not taking opioids. → *t*-tests
Harding (2019) [58]	US	CS	Chronic pain Pain duration: ≥3 m	127 74.0/25.2/ 0.8% transgender 52.60 ± 12.07 y	*General anxiety symptoms* → PROMIS Emotional Distress - Anxiety subscale *Depressive symptoms* → PROMIS Emotional Distress - Depression subscale	Patient-reported Retrospective (past 3 m) - Use of provider management → yes/no for each of the following: massage, osteopathic manipulation, trigger point injection, spine/joint/facet injections, spinal cord stimulation, counseling/talk therapy and surgery *(amount; HCU general)* - Use of self-management → yes/no for each of the following: water therapy/swimming, another exercise, heat/cold application, TENS, ultrasound, brace/corset, pain education/self-help books and relaxation practice *(amount; CAM use)* → For each category the number of “yes” answers was added (higher number indicates the use of more different types of either provider or self-management)	To investigate whether anxiety and depressive symptoms are significantly related to the number of different provider management categories and self-management strategies used. → Correlations
							To investigate whether depressive and anxiety symptoms were influencing the number of different provider management categories and self-management strategies used while controlling for age, gender, pain intensity, pain interference, PTSD and sleep. → Linear regression
Hill (2007) [59]	UK	CS	Musculoskeletal hand problems	2113 37/63 65.4 ± 9.6 y	*Frustration* → Arthritis Impact Measurement Scale-2 - Frustration subscale → Dichotomized to no days (reference)/few or all days *Negative consequences beliefs* → Illness Perception Questionnaire-Revised - Consequences subscale *Negative illness beliefs* → Illness Perception Questionnaire-Revised - Timeline cyclical - timeline acute/chronic → dichotomized to low (reference)/high score *Psychological distress* → Illness Perception Questionnaire-Revised - Emotional representations subscale *Illness coherence* → Illness Perception Questionnaire-Revised - Illness coherence subscale *Perceived symptom control* → Illness Perception Questionnaire-Revised - Personal control and treatment control subscales *Perceived cause of symptoms* → Illness Perception Questionnaire-Revised - Psychological attributions	Patient-reported Retrospective (past 12 m) - Consultations with GP → Adjusted Knee Pain Screening Tool (dichotomized to yes/no) *(type; primary care consultations)* - Medication consumption → Arthritis Impact Measurement Scales 2 (dichotomized to no/some) *(type; pain medication use)*	To investigate whether the listed CEF were influencing the likelihood of having GP consultations (reference: no GP consultations) and using medication (reference: no medication use). → Univariate logistic regression. It appears that univariate results were only reported for those associations that were found to be significant in multivariate analyses. Because of this unclarity the univariate results were not included in this review for those relationships that were insignificant in multivariate analyses.
							To investigate whether the listed CEF were influencing the likelihood of having GP consultations (reference: no GP consultations) and using medication (reference: no medication use) while also accounting for age, sex and diagnosis. → Multivariate logistic regression
Howell (1999) [60]	AU	CS	Dyspepsia (upper gastrointestinal symptoms)	614 Previous HCU 73.5/84.1 46.97 ± 14.32 y Non-users 46.55 ± 15.24 y	*Symptom-related anxiety symptoms* - Symptom-related anxiety → self-designed question w/ answer options: none; a little; moderate; considerable; extreme - Fear of serious illness → yes/no - Fear that pain might be cancer → yes/no	Patient-reported Retrospective (past year) - Presence of prior GP visits for dyspepsia symptoms → yes/no *(type; primary care consultations)* - Frequent GP visits for dyspepsia symptoms: 6 or more in the past year → yes/no *(amount; consultations)*	To compare the listed CEF between patients who had prior GP visits and those who did not. → Chi^2^
							To investigate whether the listed CEF were influencing the likelihood of having had prior GP visits (reference: no visits) while also accounting for gender, alcohol consumption, marital status, ethnicity, smoking status, NSAID use, age, neuroticism, pain frequency, pain duration and pain severity. → Logistic regression Independent variables were omitted from the final model if not contributing significantly.
							To compare the listed CEF between patients having frequent GP visits and non-frequent visitors. → Chi^2^
							To investigate whether the listed CEF were influencing the likelihood of having ≥6 GP visits (reference: <6) while also accounting for gender, alcohol consumption, marital status, ethnicity, smoking status, NSAID use, age, neuroticism, pain frequency, pain duration and pain severity. → Logistic regression Independent variables were omitted from the final model if not contributing significantly.
Huffman (2017) ^5^ [121]	US	C	Patients w/ chronic non-cancer pain following an interdisciplinary outpatient program	1457 37.88/62.12 46.29 ± 13.72 y	Assessed at baseline and program discharge *General anxiety symptoms* → Depression, Anxiety and Stress Scale - Anxiety subscale *Depressive symptoms* → Depression, Anxiety and Stress Scale - Depression subscale	Database extraction Retrospective Chronic opioid use at program admission → no/low dose/high dose chronic opioid therapy *(type; opioid use)*	To compare the listed CEF between the different opioid use groups at baseline. → ANOVA
							To investigate whether level of baseline opioid use was influencing the listed post-discharge CEF while controlling for marital status, age, gender and baseline score of the respective CEF. → Linear mixed models
Jensen (1994) [128]	US	C	Chronic pain Participating in a 3 w multidisciplinary pain program Mean pain duration: 5.26 y (range: 3 m–32 y)	94 40/60 42 y	Assessed at baseline and follow-up → changes from baseline to follow-up *Catastrophizing* → Coping Strategies Questionnaire - Catastrophizing subscale *Helplessness* → Coping Strategy Questionnaire - Factor analysis of the changes in subscale scores from baseline to follow-up (3 to 6 m post-treatment) resulted in 1 factor of interest: “Helplessness” (loadings: Praying and hoping 0.61; Catastrophizing 0.45) *Negative consequences beliefs* → Survey of Pain Attitudes - Disability and harm subscales *Negative illness beliefs* → Survey of Pain Attitudes - Factor analysis on the changes in subscale scores from baseline to follow-up resulting in the factor “pain as illness belief” (3 to 6 m post-treatment) resulted in the factor “Pain as illness belief” (Loadings: disability 0.82; Harm 0.75; Pain control −0.70; Medication 0.51; Medical cure 0.44; Solicitude 0.38) and the subscales: medical cure, medication and solicitude *Perceived symptom control* → Survey of Pain Attitudes - Pain control subscale	Patient-reported Retrospective (last 3 m; measured at baseline and follow-up (3 to 6 m post-treatment) → changes from baseline to follow-up Number of pain-related visits to physicians *(amount; consultations)*	To investigate correlations between changes in the listed CEF and changes in the amount of physician visits. → Zero-order correlations
							To investigate the influence of changes in helplessness and pain as illness belief scores on post-treatment physician visits while also accounting for the baseline value of physician visits, cognitive coping attempts and coping ratings (exercise and relaxation, illness focus strategies and keeping busy). → Multiple regression Independent variables were omitted from the final model if not contributing significantly.
Jensen (2006) ^5^ [122]	DK	C	Patients w/chronic non-cancer pain who received a multidisciplinary pain treatment in the past Pain duration at baseline: <5 y: 54% 5–10 y: 21% >10 y: 25%	160 40/60 48 y	Measured 10 y after treatment discharge *General anxiety symptoms* → Hospital Anxiety and Depression Scale - Anxiety subscale *Catastrophizing* → Coping Strategies Questionnaire - Catastrophizing subscale *Depressive symptoms* → Hospital Anxiety and Depression Scale - Depression subscale *Psychological distress* → SF-36 - Mental health subscale	Patient-reported Prospective (current use; measured 10 y after treatment discharge) Opioid use → yes/no *(type; opioid use)*	To compare the listed CEF between users and non-users of opioids. → Chi^2^
Jordan (2006) ^5^ [104]	UK	C	Knee pain in older people w/o knee disorder consultation in the past 18 m Pain duration: <3 m: 870 ≥3 m: 862	1797 43/57 64.2 ± 9.46 y	Assessed at baseline *General anxiety symptoms* → Hospital Anxiety and Depression Scale - Anxiety subscale *Depressive symptoms* → Hospital Anxiety and Depression Scale - Depression subscale → Both dichotomized to most symptoms (being >top tertile of HADS scores) and less symptoms (≤top tertile)	Database extraction Retrospective (18 m after CEF survey) Recorded primary care visit for a knee disorder → yes/no *(type; primary care consultations)*	To investigate whether showing most depressive or anxiety symptoms (reference: less symptoms) were influencing the likelihood of having a future primary care consultation for a knee disorder (reference: no consultation). → Logistic regression
							Idem, while also accounting for BMI, widespread pain, favorable evaluation and frequency of consulting. → Logistic regression
Jöud (2017) [7]	SE	CS	People experiencing pain Pain duration: <3 m: 1019 ≥3 m: 6773	7792 39/61 56 y (median; Q1–Q3: 42–67 y)	*Catastrophizing* → Pain Catastrophizing Scale (PCS) → sample subdivided into PCS > 17; PCS 10–17; PCS < 10 (reference)	Patient-reported Retrospective (last 3 m) Pain-related healthcare consultation → yes/no *(type; consultations)*	To investigate whether level of PCS score (reference: PCS < 10) was significantly influencing the likelihood of having a pain-related healthcare consultation (reference: no consultation) while also accounting for age, education, sex, pain spread, pain intensity and pain duration. → Poisson regression
Kapoor (2012) ^5^ [123]	US	C	Patients w/ chronic non-cancer pain participating in an RCT comparing cognitive behavioral therapy to an education intervention	64 26.6/73.4 49.34 ± 12.48 y	Measured at baseline and completion of treatment *Catastrophizing* → Pain Catastrophizing Scale *Depressive symptoms* → Center for Epidemiological Studies Depression Scale	Database extraction Retrospective (3 m before and 12 m after treatment) Number of visits to rural healthcare center *(amount; consultations)*	To investigate the association between the listed CEF and number of healthcare visits pre- and post-treatment. → Correlations
							To investigate whether the listed baseline CEF were influencing the number of visits pre- and post-treatment initiation while also accounting for age, income, number of pain locations, duration of pain, sex, quality of life and self-reported disability. → Multivariate regression analysis Only independent variables showing a significant correlation w/ the respective HCU outcome were included in the multivariate model.
Kapoor (2014) [61]	US	CS	Chronic pain (rural, low-income population) Pain duration: 12.54 ± 16.28 y	64 26.6/73.4 49.34 ± 12.48 y	*Catastrophizing* → Pain Catastrophizing Scale *Depressive symptoms* → Center for Epidemiologic Studies Depression Scale	Database extraction Retrospective (past 3 m) - Total number of healthcare visits *(amount; consultations)* - Prescription of opioids → yes/no *(type; opioid use)*	To examine the association between the listed CEF and total number of healthcare visits. → Correlations
							To investigate whether the listed CEF ** were influencing the number of healthcare visits while also accounting for number of comorbidities *, pain intensity *, age ** and pain disability **. → Poisson regression * Fixed factor in model ** Only included in regression if significant in correlation analyses
							To examine the association between the listed CEF and receiving an opioid prescription (reference: no prescription). → Correlations
Keeley (2008) [105]	UK	C	Chronic low back pain Mean pain duration: 5.5 ± 5.7 y Median pain duration: 4.0 y	108 55.6/44.4 39.9 ± 12.2 y n = 86 for HCU data	Assessed at baseline *Fear-avoidance beliefs* → Fear Avoidance Beliefs Questionnaire - Work and activity beliefs subscales *Psychological distress* → Hospital Anxiety and Depression Scale - Total score *Stress* → Life Events and Difficulties Schedule Back pain-related and back pain-independent social stress subscales	Patient-reported Retrospective (6 m post-baseline) Total number of contacts with healthcare services → Client Socio-Demographic and Service Receipt Inventory *(amount; consultations)*	To investigate whether baseline CEF were influencing number of healthcare contacts at follow-up while controlling for age, education, cause of pain and duration of pain. → Negative binomial regression
Kratz (2018) [62]	US	CS	Spinal cord injury with chronic pain Time since injury: 14.57 ± 12.34 y	120 73/27 46.93 ± 46.93 y	*Depressive symptoms* → Patient Health Questionnaire-9 *Pain acceptance* → Chronic Pain Acceptance Questionnaire (CPAQ) - Total + Pain willingness and activities engagement subscales	Patient-reported Prospective (current use) - Total number of pain medications used *(amount; pain medication use)* - Use of opioids → yes/no *(type; opioid use)* - Use of Gabapentin → yes/no *(type; prescription pain medication use)*	To investigate if depressive symptoms and chronic pain acceptance (CPAQ total) were influencing the number of pain medications used while also accounting for pain intensity and number of painful body areas. → Poisson regression
							Idem but w/pain willingness and activities engagement subscales instead of the total CPAQ score. → Poisson regression
							To investigate if chronic pain acceptance was influencing the likelihood of using opioid and Gabapentin (reference: no use for both) while also accounting for pain intensity and number of painful body areas. → Logistic regression
							Idem but w/pain willingness and activities engagement subscales instead of the total CPAQ score. → Logistic regression
Kuijper (2014) [106]	NL	C	Patients presenting arthralgia w/o synovitis and rheumatoid arthritis patients Pain duration: Non-synovitis: Median: 136 d Range: 7–380 d Rheumatoid arthritis: Median: 103 d Range: 7–373 d	Non-synovitis: 330 15/85 45.0 ± 12.4 y Rheumatoid arthritis: 244 32/68 54.0 ± 13.7 y	Measured at baseline *Psychological distress* → SF-36 - Mental component subscale *Locus of control* → Multidimensional Health Locus of Control Questionnaire - Internal, external and chance subscales	Patient-reported Retrospective (past 6 m; measured at baseline, 6 and 12 m follow-up) Number of visits w/ healthcare providers for joint symptoms → Transformed into combined HCU outcome = visits to GP + medical specialist + PT divided by 5 + alternative healthcare providers *(amount; consultations)*	To investigate whether the listed baseline CEF ** were influencing the number of healthcare visits 6 m later in patients w/o synovitis while also accounting for month *, age *, sex *, ethnicity **, education **, household composition **, employment **, BMI **, duration of symptoms **, diagnosis **, comorbidities **, coping **, pain **, fatigue ** and SF-36 physical component **. → Poisson regression * Fixed factors in model ** If significant in univariate analysis (not reported)
							To investigate whether the listed baseline CEF ** were influencing the number of healthcare visits 6 m later in patients w/ rheumatoid arthritis while also accounting for month *, age *, sex *, ethnicity **, education **, household composition **, employment **, BMI **, duration of symptoms **, diagnosis **, comorbidities **, coping **, pain **, fatigue ** and SF-36 physical component **. → Poisson regression * Fixed factors in model ** If significant in univariate analysis (not reported)
Lee (2008) [63]	UK	CS	Functional bowel disease Abdominal pain for >12 w: 67%	420 11/89 40.2 ± 14.4 y	*Psychological distress* → General Health Questionnaire-28	Patient-reported Retrospective (past 12 m) Number of GP visits for bowel symptoms *(amount; consultations)*	To investigate the association between psychological distress and number of GP visits. → Correlations
							To investigate whether psychological distress * was influencing number of GP visits while also accounting for duration of IBS symptoms *, more severe IBS score *, symptom severity *, employment *, pain relief by opening bowels *, pain duration * and bowel passing *. → Negative binomial regression * These independent variables were selected based on their significance in univariate correlations (not reported)
Lentz (2018) [107]	US	C	Patients receiving out-patient PT for a primary complaint of musculoskeletal knee, shoulder, back or neck pain	246 34.6/65.0 46.59 ± 16.00 y	Measured at baseline (PT initiation) *Psychological distress* → OSPRO Yellow Flag (OSPRO-YF) tool → 2 variables: - 10-item OSPRO-YF score - 7 extra OSPRO-YF items Additionally, change in 10-item score from baseline to 4 w follow-up was calculated.	Patient-reported Retrospective (at 6 m follow-up for the previous 2 m; at 12 m follow-up for the previous 6 m → 8 m overview of HCU) Additional HCU after completion of initial PT program for primary musculoskeletal pain: - Opioid painkillers *(type; opioids)* - Injections *(type; invasive procedures)* - Surgeries *(type; invasive procedures)* - Diagnostic tests/imaging *(type; secondary care consultations)* - ER visits *(type; emergency HCU)* - Any HCU *(amount; HCU in general)* → yes/no for each	To investigate the influence of baseline psychological distress (OSPRO-YF 10-item + 7 items) and change in OSPRO-YF 10-item score on the likelihood of using the listed HCU outcomes (reference: no use) while also accounting for age, sex, race, anatomical region of pain, insurance, chronicity, surgery for current condition, comorbidity, baseline disability, baseline pain intensity and OSPRO Review of Systems score (10-item + 13 items), change in pain intensity and disability. → Logistic regression Independent variables were omitted from the final model if not contributing significantly.
Levenson (2008) [108]	US	C	Sickle cell disease (SCD)	232 38.4/61.6 Mean age: 34 y Range: 16–64 y 16–24 y: n = 51 25–34 y: n = 69 35–44 y: n = 66 45–54 y: n = 35 55–64 y: n = 11	Assessed at baseline *General anxiety symptoms* → Generalized Anxiety Disorder-7 *Depressive symptoms* → Patient Health Questionnaire-9 → Both dichotomized as: Clinically relevant symptoms yes/no	Patient-reported Retrospective (daily diary questioning the past 24 h; filled out for up to 6 m) Frequency of SCD-related: - Scheduled physician visits *(amount; consultations)* - Unscheduled physician visits *(amount; emergency HCU)* - ER visits *(amount; emergency HCU)* - Hospitalizations *(amount; hospitalizations)* - Opioids taken for sickle cell pain *(amount; pain medication use)*	To compare the percentage of days on which the listed healthcare services were used between patients w/ and w/o clinically relevant depressive symptoms. → Generalized estimating equations
							Idem, but controlling for age and income. → Generalized estimating equations Only executed for significant univariate associations.
							To compare the amount of scheduled physician visits and opioids used between patients w/ and w/o clinically relevant anxiety symptoms. → Generalized estimating equations
							Idem, but controlling for age and income. → Generalized estimating equations Only executed for significant univariate associations.
Lozano-Calderon (2008) [131]	US	CC	Trapezio- metacarpal joint arthrosis	72 19.4/80.6 65 ± 12.8 y Requested operative treatment: n = 31 Not opting for operative treatment: n = 41	*Symptom-related anxiety symptoms* → Pain Anxiety Symptoms Scale *Catastrophizing* → Pain Catastrophizing Scale *Depressive symptoms* → Center for the Epidemiological Study of Depression	Patient-reported Opting for surgery → yes/no *(type; invasive procedures)*	To compare the listed CEF between patients opting for surgery and those who do not. → *t*-test
Lozier (2018) [64]	US	CS	People w/chronic musculoskeletal pain prescribed long-term opioid therapy	517 Clinician- directed NPTs by level of engagement: No: 61.5 ± 10.9 y Low: 59.2 ± 11.5 y Moderate: 57.5 ± 10.1 y High: 52.4 ± 12.7 y Self-directed NPTs by level of engagement: No: 59.9 ± 11.5 y Low: 59.0 ± 10.0 y Moderate: 58.6 ± 12.3 y High: 58.3 ± 12.0 y	*General anxiety symptoms* → Generalized Anxiety Disorder-7 *Depressive symptoms* → Patient Health Questionnaire *Self-efficacy beliefs* → Pain Self-Efficacy Questionnaire	Patient-reported Retrospective (past 6 m) Frequency of non-pharmacological treatments (NPTs) use → subdivided into: - Clinician-directed NPTs (PT, TENS, chiropractic treatment, acupuncture, massage and psychoeducational courses (e.g., CBT)) *(amount; consultations)* - Self-directed NPTs (weight/strength training, yoga, tai chi, pool exercise/swimming and herbal medicine) *(amount; CAM use)* → For each of both types of NPTs an engagement score was calculated based on frequency of use and the different types of treatments within both categories used, resulting in 4 categories: no, low, moderate and high engagement.	To compare CEF between different engagement groups of clinician-directed NPTs. → One-way ANOVA
							To investigate whether depressive symptoms or self-efficacy scores were influencing the use of clinician-directed NPTs while also accounting for site, age, gender, opioid dose, ethnicity, education and pain disability. → Multinomial regression analysis
							To compare CEF between different engagement groups of self-directed NPTs. → One-way ANOVA
							To investigate whether depressive symptoms or self-efficacy scores were influencing the use of self-directed NPTs while also accounting for site, age, gender, dose, ethnicity, education and pain disability. → Multinomial regression analysis
Macfarlane (1999) [65]	UK	CS	Chronic widespread pain Pain duration: >3 m	252 35/65 18–32 y: 36 33–42 y: 45 43–52 y: 77 53–65 y: 94	*Symptom-related anxiety symptoms* → Illness Attitude Scale - Disease phobia subscale *Catastrophizing* → Illness Attitude Scale - Hypochondriacal beliefs subscale *Health worry* → Illness Attitude Scale - Worry about health and concerns about pain subscales *Psychological distress* → General Health Questionnaire → Scoring above the median yes/no for logistic regression *Symptom vigilance* → Illness Attitude Scale - Bodily preoccupations subscale *Thanatophobia* → Illness Attitude Scale - Thanatophobia subscale	Patient-reportedRetrospective (past month) Consultation w/GP for pain → yes/no *(type; primary care consultations)*	To compare the listed CEF between GP consulters and non-consulters. → Mann–Whitney U
							To investigate whether scoring > the median on psychological distress (reference: ≤median) was influencing the likelihood of having a GP consultation for pain (reference: no consultation) in men and women separately, while controlling for age. → Logistic regression
Macfarlane (2003) [66]	UK	CS	Orofacial pain Pain duration: <3 m: 137 ≥3 m: 279	555 34/66 18–25 y: 71 26–35 y: 120 36–45 y: 125 46–55 y: 151 56–65 y: 88	*Psychological distress* → General Health Questionnaire-12 → Subdivided into GHQ-12 score: - 0 - 1–3 - 4–12 *Perceived symptom-control* → Self-designed question for pain control → Subdivided into pain control score: - 0–2 - 3–4 - 5–6	Patient-reported Retrospective (past month) Consultation for orofacial pain → yes/no *(type; consultations)*	To investigate the association between level of psychological distress (reference score: 0) and pain control (reference score: 5–6) and having a healthcare consultation for orofacial pain (reference: no consultation). → Cox regression
Mann (2017) [67]	CA	CS	Community- dwelling individuals w/ chronic pain Pain duration: ≥3 m	702 51/49 59 ± 13 y	*Depressive symptoms* → Patient Health Questionnaire-9 *Self-efficacy beliefs* Pain self-efficacy → Pain Self-Efficacy Questionnaire	Patient-reported Retrospective (past year) Number of health-related visits for any reason to: - GP - Specialist - Walk-in clinic → the previous 3 were combined into 1 binary variable: high (top 10% in terms of frequency of visits) vs. low clinic use *(amount; consultations)* Number of ER visits → transformed into binary variable: high (top 10% in terms of frequency of visits) vs. low ER use *(amount; emergency HCU)*	To compare the level of the listed CEF between high vs. low clinic users and between high vs. low ER users. → Relative risk comparisons
							To investigate whether the listed CEF were influencing the likelihood of having high clinic and ER use (reference: low use) while also accounting for demographics (gender, marital status, education and annual household income), need factors (pain intensity, number of pain locations, pain frequency and presence of comorbidities, neuropathic component, back problems, arthritis, probable nerve damage, other pain diagnosis) and personal health behaviors (use of prescription medication, non-prescription medication, PT or exercise, chiropractic or massage therapy, invasive intervention and other therapy or intervention). → Logistic regression Independent factors were only included in the regression analysis if significant in univariate analysis. Independent variables were also omitted from the final model if not contributing significantly.
Mannion (2013) [68]	CH	CS	Low back pain (episode in the last month)	1071 48.6/51.4 53 ± 15.8 y	*Fear-avoidance beliefs* → Fear-Avoidance Beliefs Questionnaire - Activity and work beliefs subscales *Negative illness beliefs* → Back Beliefs Questionnaire *Psychological distress* → Euroqol (EQ5D) - Depression/anxiety subscale	Patient-reported Retrospective (past 4 w) Low back pain-related consultations to specialists, GP, PT or other practitioner → dichotomized into HCU yes/no *(type; consultations)*	To investigate if the listed CEF were influencing the likelihood of using healthcare for low back pain (reference: no HCU). → Univariate logistic regression
							To investigate if level of fear-avoidance beliefs (activity and work beliefs) and psychological distress were influencing the likelihood of using healthcare for low back pain (reference: no HCU) while controlling for sex, age, education, general health, working status, household composition (−18 y), income, low back pain frequency, low back pain intensity and limitations in ADL. → Multivariate logistic regression
							To investigate if level of back beliefs and psychological distress were influencing the likelihood of using healthcare for low back pain (reference: no HCU) while also accounting for sex, age, education, general health, working status, household composition (−18 y), income, low back pain frequency, low back pain intensity and limitations in ADL. → Multivariate logistic regression
McCracken (1997) [69]	US	CS	Chronic low back pain Pain duration: Median: 36 m Range: 3–360 m	80 41.2/58.8 48.0 ± 15.3 y	*Symptom vigilance* → Pain Vigilance and Awareness Questionnaire	Patient-reported Retrospective (past 9 m) Number of physician visits due to pain *(amount; consultations)*	To investigate the association between attention to pain and number of pain-related physician visits. → Correlations
							To investigate whether attention to pain was influencing the number of pain-related visits while also accounting for age, gender, education, pain duration and pain intensity *. → Hierarchical multiple regression * Fixed factor Other independent variables were omitted from the final model if not contributing significantly.
McCracken (2005—Pain) [109]	UK	C	Chronic pain Scheduled for interdisciplinary treatment (=PT and psychosocial therapy) Pain location: 49.6% low back 13.8% lower limb 12.2% upper limb 11.4% neck 13.0% other Pain duration: Median: 87.5 m Range: 12–528 m	118 36/64 44.2 ± 10.7 y	Assessed at baseline *Pain acceptance* → Chronic Pain Acceptance Questionnaire - Activities engagement and pain willingness subscales + total score	Patient-reported Retrospective (current use; measured at follow-up (3.9 m later on average)) Count of analgesic medications *(amount; pain medications use)*	To investigate the correlations between baseline pain acceptance scores and amount of pain medication use at follow-up. → Correlations
							To investigate whether baseline pain willingness and activities engagement scores were influencing the amount of pain medication use at follow-up while also accounting for pain intensity *, age, gender, education and duration of pain. → Multiple regression * Fixed factor Independent variables were omitted from the final model if not contributing significantly.
McCracken (2005—Beh Res Ther) [124]	UK	C	Chronic pain patients following an acceptance-based treatment Pain location: 49.6% low back 13.8% lower limb 12.2% upper limb 11.4% neck 13.0% other Pain duration: Mean: 132.5 ± 127.8 m Median: 92.0 m Range: 12–528 m	108 35.8/64.2 44.4 ± 10.7 y	Assessed at baseline (pre-treatment), start of treatment, end of treatment and 3 m follow-up *Pain acceptance* → Chronic Pain Acceptance Questionnaire - Activity engagement and pain willingness subscales + total score	Patient-reported Retrospective (current situation; assessed at baseline (pre-treatment), start of treatment, end of treatment and 3 m follow-up) Number of pain-related medication prescriptions *(amount; pain medication use)*	To investigate the correlation between changes in pain acceptance scores from pre- to post-treatment and changes in the number of pain medications used from pre- to post-treatment. → Correlations
McCracken (2007) [70]	UK	CS	Chronic pain Diagnoses: 32.8% fibromyalgia 21.3% other nonspecific musculoskeletal pain conditions 15.6% unknown 11.5% post lumbar surgery pain 18.8% other Pain duration: Median: 84.0 m Range: 8.0–552.0 m	260 35.4/64.6 47.5 ± 11.5 y	*Psychological flexibility* → Brief Pain Coping Inventory-2 - Psychological flexibility subscale	Patient-reported Retrospective (timing see below) - Current types of pain medications *(amount; pain medication use)* - Strong opioid use *(amount; pain medication use)* - Number of pain-related GP, specialist and ER visits in the past 6 m → Summed into an overall pain-related medical visits score *(amount; consultations)*	To investigate correlations between psychological flexibility and the listed HCU outcomes. → Correlations
							To investigate whether level of psychological flexibility * was influencing the number of pain medications used and the number of pain-related visits while also accounting for age, gender, years of education, duration of pain, pain intensity and pain management strategies *. → Multiple regressions * Fixed factors Independent variables were omitted from the final model if not contributing significantly.
Mourad (2016) [72]	SE	CS	Non-cardiac chest pain	552 49/51 64 ± 17 y	*Symptom-related anxiety symptoms* - Cardiac anxiety → Cardiac Anxiety Questionnaire - Fear subscale + total score - Fear of body sensations → Body Sensations Questionnaire *Depressive symptoms* → Patient Health Questionnaire-9 *Symptom vigilance* Heart-focused attention → Cardiac Anxiety Questionnaire - Heart-focused attention subscale	Patient-reported Retrospective (past year) Number of healthcare visits for chest pain → Categorization for univariate analyses: - Very high users (>3 visits) - High users (2–3 visits) - Low users (<2 visits) → Categorization for multivariate regression: - High users (≥ 2 visits) - Low users (1 visit) *(amount; consultations)*	To compare CEF between the different frequency of healthcare visits categories. → Kruskal–Wallis
							To investigate whether the listed CEF were influencing the frequency of healthcare visits while controlling for age, sex and multi-morbidity. → Multivariable logistic regression
Mourad (2018) [71]	SE	CS	Non-cardiac chest pain	552 49/51 64 ± 17 y	*Symptom-related anxiety symptoms* - Cardiac anxiety → Cardiac Anxiety Questionnaire - Fear of body sensations → Body Sensations Questionnaire *Depressive symptoms* → Patient Health Questionnaire-9	Patient-reported Retrospective (past year) Frequency of pain-related healthcare visits → categorized into: - 1 time - 2–3 times - >3 times *(amount; consultations)*	To model the relationship between the listed CEF and frequency of pain-related healthcare visits while also accounting for somatization. → Structural equation modelling
Musey (2018) ^5^ [110]	US	C	Low-risk chest pain	163 32/68 47.4 ± 10.8 y	Assessed at enrollment *General anxiety symptoms* → Hospital Anxiety and Depression Scale - Anxiety subscale (HADS-A) → dichotomized into: - High anxiety (HADS-A ≥ 8) - Low anxiety (HADS-A < 8)	Database extraction Retrospective (past 12 m before enrollment) ER visits → yes/no *(type; emergency HCU)*	To compare the proportion of patients having at least 1 ER visit (reference: no visit) between patients w/ high and low levels of anxiety symptoms. → Chi^2^
						Database extraction Prospective (30 d after enrollment) Number of ER return visits (ER recidivism) *(amount; emergency HCU)*	To compare amount of ER recidivism between patients w/ high and low levels of anxiety symptoms. → *t*-test
Navabi (2018) ^5^ [111]	US	C	IBS Mean duration of symptoms: 12.6 ± 0.5 y	432 47.2/52.8 42.3 ± 0.6 y	Assessed at baseline *Psychological distress* Anxiety and/or depressive symptoms → Hospital Anxiety and Depression Scale - Anxiety and depression subscale → Categorized as: depressive and/or anxiety symptoms (HADS-A or -D ≥8) vs. no symptoms (HADS-A or -D <8)	Database extraction Retrospective (during 18 m study duration) - Opiate use → yes/no *(type; opioid use)* - Corticosteroid use → yes/no *(type; prescription medication use)* - Number of ER visits for IBS symptoms *(amount; emergency HCU)* - Number of hospital admissions for IBD symptoms *(amount; hospitalizations)* - Number of imaging studies for IBS symptoms *(amount; consultations)* - Number of surgeries for IBS symptoms *(amount; invasive procedures)* → Dichotomized to history of surgery yes/no for logistic regression *(type; invasive procedures)*	To compare the listed HCU outcomes between patients w/ and w/o symptoms of depression and/or anxiety. → *t*-test, Chi^2^ or Fisher exact test
							To investigate whether a history of surgery, corticosteroid use and opiate use were influencing the presence of anxiety and/or depressive symptoms (reference: no symptoms) while also accounting for significant inflammation, age, disease duration, gender, Mesalamine use, immunomodulator use, anti-TNF use, history of extra-intestinal manifestations and tobacco use. → Logistic regression Independent variables in the model were selected based on their significance in univariate comparisons.
Ndao-Brumblay (2010) ^5^ [73]	US	CS	Chronic pain Mean pain duration: 45.82 ± 64.68 m	5,079 39.3/60.7 46.42 ± 15.0 y	*Depressive symptoms* → Beck Depression Inventory *Perceived symptom control* → Likert scale for perceived pain control	Patient-reported Retrospective (since the beginning of pain problem) Use of CAM modalities for pain: - Acupuncture - Manipulation - Biofeedback/relaxation → Dichotomized to yes/no for all + CAM use in general yes/no *(all: type; CAM use)*	To compare the level of the listed CEF between CAM users and non-users. → Bivariate analyses
							To investigate whether the level of the listed CEF were influencing the likelihood of using each of the listed CAM modalities for pain while also accounting for predisposing (age, gender, race, education, marital status and pain care perception), enabling (pain prediction and residence income) and need factors (comorbidities, number of operations, pain duration, pain severity and functional limitations). → Logistic regressions
Newman (2018) ^5^ [74]	US	CS	Low-income patients with chronic pain Mean pain duration: 16.6 ± 12.2 y	290 29.3/70.7 50.6 ± 8.9 y	*Catastrophizing* → Pain Catastrophizing Scale *Depressive symptoms* → Patient Health Questionnaire-9	Database extraction Retrospective (past 12 m) - Number of pain-related consultations *(amount; consultations)* - Opioid prescription → yes/no *(type; opioid use)*	To investigate the association between the listed CEF and number of pain-related consultations. → Pearson correlations
							To investigate the association between the listed CEF and opioid use. → Point-biserial correlation
							To investigate whether the listed CEF were influencing the number of pain-related consultations while also accounting for demographics (age, sex and race), socioeconomic variables (poverty status, education and literacy) and pain-related variables (physical function, pain severity, pain interference, number of pain sites and types and opioid use). → Hierarchical multiple regression
Nielsen (2015) [75]	AU	CS	Chronic non-cancer pain Median pain duration: Non-users: 10.0 y Past users: 10.0 y Current less than daily users: 12.0 y Current daily users: 12.0 y	1220 Non-users: n = 450 51.3/48.7 60.5 ± 13.6 y Past users: n = 372 39.5/60.5 56.6 ± 13.1 y Current less than daily users: n = 186 49.5/50.5 53.8 ± 12.6 y Current daily users: n = 212 39.2/60.8 54.5 ± 12.9 y	*General anxiety symptoms* → Generalized Anxiety Disorder-7 → Dichotomized to GAD-7 ≥10 vs. <10 *Depressive symptoms* → Patient Health Questionnaire-9 → Dichotomized to PHQ-9 ≥10 vs. <10 *Self-efficacy beliefs* Pain self-efficacy → Pain Self-Efficacy Questionnaire	Patient-reported Mixed (see below) Benzodiazepine (BZD) use: - Number of days on which medicine was used in the past month - Medication diary reporting all medications taken over a period of 7 d → Categorized into: - Non-users of BZD - Past users of BZD (not in the past month) - Current less than daily BZD users - Current daily users of BZD *(amount; pain medication use)*	To compare the likelihood of reporting clinically relevant symptoms of anxiety or depression between the different BZD use groups (reference: non-users). → Multinomial regression
							To compare level of pain self-efficacy between the different BZD use groups while controlling for pain severity. → ANCOVA
Osborne (2007) [129]	AU	C	Patients w/ chronic osteoarthritis who follow the Arthritis Self-Management Course	452 19/81 62.8 ± 12.4 y	Assessed at baseline (before treatment) and 6 m after treatment *Self-efficacy beliefs* Pain and fatigue self-efficacy → 4 items from the original 8-item Stanford Scale (overall score based on the mean of the 4 items) → Dichotomized to positive self-efficacy change vs. negative or no change	Patient-reported Retrospective (past 6 m; assessed at baseline (before treatment) and 6 m after treatment) - Number of doctor visits *(amount; consultations)* - Number of PT visits *(amount; consultations)* - Number of CAM visits *(amount; CAM use)* - Number of hospital admissions *(amount; hospitalizations)* - Length of hospital stay *(amount; hospitalizations)* → Dichotomous variables created for each of the above: >median number of uses vs. less	To investigate whether having an above median use of the listed healthcare services (reference: lower than median use) was influencing the likelihood of having a positive change in self-efficacy at 6 m post-treatment (reference: negative or no change) while also accounting for age, sex, education level, course attendance, baseline self-efficacy and baseline use of the respective HCU outcome. → Logistic regression
Pagé (2019) [112]	CA	C	Patients w/ chronic low back pain who follow a multidisciplinary pain treatment Mean pain duration: 7.7 ± 9.2 y Median pain duration: 4 y	686 44.2/55.8 56.5 ± 14.5 y	Assessed at baseline and 6 and 12 m after treatment initiation *Depressive symptoms* → Beck Depression Inventory	Patient-reported Retrospective (assessed at 6 and 12 m after treatment initiation for the previous 6 m) Use of any of these treatments: - Psychological treatment (psychotherapy or group therapy) *(type; primary care consultations)* - Self-management (relaxation, meditation, hypnosis, visualization, distraction, self-help group) *(type; CAM use)* → yes/no for both	To compare level of depressive symptoms between patients using psychological treatment and those who do not and between those who use self-management modalities and those who do not at 6 and 12 m after treatment initiation. → *t*-tests
Philpot (2018) ^5^ [125]	US	C	Patients w/ chronic non-cancer pain enrolled in Controlled Substance Agreements (CSA) for long-term opioid therapy	772 35/65 63.5 ± 14.9 y	Assessed at CSA enrollment *General anxiety symptoms* → Generalized Anxiety Disorder-7 (GAD-7) *Depressive symptoms* → Patient Health Questionnaire-9 (PHQ-9) → Both dichotomized to score ≥ 10 vs. score < 10	Database extraction Retrospective (12 m before and 12 m after CSA enrollment) Number of: - Outpatient primary care visits - Outpatient specialist visits - Hospitalizations - ER visits → Difference in frequency was calculated between pre- and post-CSA enrollment → Dichotomized into decrease yes/no for each HCU type according to the following rules for decrease: - Hospitalization: decrease ≥ 1 *(amount; hospitalizations)* - ER visits: decrease ≥ 1 *(amount; emergency HCU)* - Primary care visits: decrease ≥ 3 *(amount; consultations)* - Specialist visits: decrease ≥ 3 *(amount; consultations)*	To investigate whether baseline presence of anxiety or depressive symptoms (reference: no symptoms) was influencing the likelihood of having a particular decrease in the listed HCU outcomes (reference: lower or no decrease) after CSA enrollment. → Univariate logistic regression
							Idem, while also accounting for race, sex, age, employment status, marital status, household composition, comorbidity, education, condition, current pain intensity, PHQ-9 functional score, GAD-7 functional score, smoking, number of opioid classes prescribed, opioid prescription dose and the respective other CEF. → Multivariable logistic regression Independent variables in the model were selected based on their significance in univariate analyses.
Pierce (2019) ^5^ [76]	US	CS	Patients w/ chronic pain and current opioid use	1785 42.18/57.82 50.34 ± 14.76 y	*General anxiety symptoms* → Hospital Anxiety and Depression Scale - Anxiety subscale *Depressive symptoms* → Hospital Anxiety and Depression Scale - Depression subscale → Both dichotomized to score ≥ 10 vs. score < 10	Patient-reported Prospective Current Benzodiazepine use → yes/no *(type; prescription pain medication use)*	To compare level of depressive and general anxiety symptoms between benzodiazepine users and non-users. → *t*-tests
							To investigate whether level of depressive and anxiety symptoms was influencing the likelihood of using benzodiazepines while controlling for age, sex, pain severity, pain interference, fibromyalgia survey score, lifetime abuse history and interactions between anxiety and child, adult and cumulative abuse. → Logistic regression
Primavera (1994) ^5^ [127]	US	C	Patients w/ analgesic rebound headache hospitalized in a multidisciplinary headache center Chronic daily headache	30	*Health attribution* → Health Attribution Test - Internal; Powerful other and Chance subscales	- Length of stay *(amount; hospitalizations)* - Amount of medication use *(amount; pain medication use)*	To investigate associations between health attribution scores and length of stay and medication use. → Correlation
Rosenberg (2008) [77]	US	CS	Chronic noncancer pain Pain duration: Median: 54 m IQR: 24–120 m	463 32/68 53 ± 12.5 y	*Depressive symptoms* → self-designed question: depressive symptoms yes/no *Self-efficacy beliefs* Pain self-efficacy → Pain Self-efficacy Questionnaire	Patient-reported Retrospective (period not specified) CAM use: acupuncture/acupressure, chiropractic, aromatherapy, vitamin and mineral supplements, meditation/yoga, garlic preparations, traditional Chinese medicine, cod liver oil, massage, primrose oil, herbs, reflexologists, acupuncturists, root doctors, herbalists, chiropractors or other alternative practitioners → Dichotomized to CAM use yes/no *(type; CAM use)*	To investigate the influence of level of psychological distress and pain self-efficacy on the likelihood of using CAM services (reference: no CAM use). → Bivariate analyses
Shmagel (2016) [78]	US	CS	Chronic low back pain Pain duration: ≥3 m	700 44.2/55.8 20–29 y: 15.1% 30–39 y: 18.3% 40–49 y: 19.3% 50–59 y: 27.4% 60–69 y: 20.0%	*Depressive symptoms* → Patient Health Questionnaire-9 (PHQ-9) → Categorized according to PHQ-9 score into: - None (1–4) - Mild (5–9) - Moderate (10–14) - Moderately severe (15–19) - Severe (20–27)	Patient-reported Retrospective (past year) Number of healthcare visits → Dichotomized to frequent users (≥10 visits per year) and normal or low users *(amount; consultations)*	To investigate whether level of depressive symptoms was influencing the frequency of healthcare visits while also accounting for age, race, gender, education level and number of medical comorbidities. → Logistic regression
Talley (1998) [79]	AU	CS	Dyspepsia Pain duration ≥5 y:	93 Consulters (n = 65): 37/63 51 ± 14 y Non-consulters (n = 28): 39/61 46 ± 15 y	*Psychological distress* → General Health Questionnaire	Patient-reported Retrospective (past year and lifetime) Visits to physicians and alternative therapists for abdominal pain or discomfort → Bowel Symptoms Questionnaire → Categorized into consulters vs. non-consulters w/ dyspepsia *(type; consultations)*	To investigate whether level of psychological distress was influencing the likelihood of having consultations for pain (reference: no consultations) in the past year and at any time. → Univariate logistic regression
Thorstensson (2009) [80]	UK	CS	Chronic hip or knee pain Pain duration: >7.5 y	1119 38/62 67.7 ± 11.0 y	*Psychological distress* Depressive/anxiety symptoms → Euroqol EQ5D - Depression/anxiety subscale → Dichotomized to depressive/anxiety symptoms yes/no	Patient-reported Retrospective (see below) During the past 12 m: - GP visits for hip or knee pain *(type; primary care consultations)* During the past 3 m: - Allied health professional *(type; consultations)* - Alternative therapist visits during the past 3 m for hip or knee pain *(type; CAM use)* → Yes/no each + yes/no for combinations *(type; consultations)*	To investigate whether presence of depressive/anxiety symptoms (reference: no symptoms) was influencing the likelihood of having a consultation w/ an allied health professional or alternative therapist (reference for both: no consultation) while adjusting for age and sex. → Logistic regression
							To investigate whether presence of depressive/anxiety symptoms (reference: no symptoms) was influencing the likelihood of having a consultation w/ a GP or w/ a combination of GP/allied therapist/alternative therapist (reference for both: no consultation) while adjusting for age, sex, BMI, deprivation, living area, pain location, pain severity, mobility problems and comorbidities. → Logistic regression
Torrance (2013) [81]	UK	CS	Chronic pain, w/ or w/o neuropathic component Pain duration: ≥3 m	2010 W/ neuropathic component (n = 399): 36.8/63.2 18–39 y: 60 40–59 y: 167 ≥60 y: 166 W/o neuropathic component (n = 1611): 42.5/57.5 18–39 y: 234 40–59 y: 622 ≥60 y: 740 Untreated w/ neuropathic component: n = 117 Treated w/ neuropathic component: n = 98	*Psychological distress* → SF-12 - Mental component scale *Self-efficacy beliefs* → Pain Self-Efficacy Questionnaire	Patient-reported Retrospective (see below) Use of neuropathic pain drug for ≥3 m (=adequate trial) or stopped because of side effects → Dichotomized to treated patients (adequate trial of at least 1 neuropathic pain drug) and those left untreated *(type; prescription pain medication use)*	To compare level of pain self-efficacy between patients treated w/ neuropathic pain drug and those left untreated. → *t*-test
Trask (2001) [82]	CA	CS	Headache	292 29.8/70.2 39.83 y (16–74 y)	*Psychological distress* → Brief Symptom Inventory → Categorized into 3 clusters: low, medium or high distress	Patient-reported Retrospective Use of the following: - Biofeedback - Relaxation - Acupuncture - Chiropractor *(all above: type; CAM use)* - Psychological care *(type; primary care consultations)* → yes/no for all Amount of: - Symptomatic medications - Preventive medications *(both: amount; pain medication use)*	To compare the odds for having sought psychological care or having used the listed adjuvant techniques (reference for all: no use of respective service) between the 3 psychological distress clusters. → Chi^2^
							To compare the number of symptomatic and preventive medications used between the 3 distress clusters. → ANOVA
Tremblay (2018) [113]	CA	C	Non-cardiac chest pain	428 48.8/51.2 53.1 ± 15.6 y	Measured at baseline *Symptom-related anxiety symptoms* Heart-focused anxiety → Cardiac Anxiety Questionnaire *Depressive symptoms* → Hospital Anxiety and Depression Scale - Depression subscale	Patient-reported Retrospective (6 m; measured 6 m after baseline) Total number of healthcare visits (primary care, specialists and ER visits) → Abbreviated version of the Health Cost Interview *(amount; consultations)*	To investigate whether depressive symptoms and heart-focused anxiety were influencing the number of healthcare consultations. → Bivariate regression analyses
							To investigate whether depressive symptoms and heart-focused anxiety were influencing the number of healthcare consultations while also accounting for age, sex, presence of panic disorder, pain frequency, pain intensity, pain interference, presence of a medical condition and gastrointestinal symptoms. → Negative binomial regression Independent variables were omitted from the model if they did not improve model parameters.
Tsuji (2019) [83]	JP	CS	Osteoarthritis Mean time since arthrosis diagnosis: 9.5 ± 11.8 y	565 41.4/58.6 59.1 ± 12.1 y	*Depressive symptoms* → Patient Health Questionnaire-9 → Dichotomized to moderate/severe vs. mild/no symptoms	Patient-reported Retrospective (past 6 m) Number of: - Physician visits *(amount; consultations)* - ER visits *(amount; emergency HCU)* - Hospitalizations *(amount; hospitalizations)*	To compare the amount of use of the different healthcare services between patients w/ moderate/severe depressive symptoms and those w/ mild or no depressive symptoms. → Mann–Whitney U test
							To investigate whether level of depressive symptoms was influencing the amount of use of the listed healthcare services while controlling for age, marital status, employment status and smoking status. → Generalized linear regression model
Ullrich (2013) [114]	US	C	Spinal cord injury (SCI)	286 97/3 53 y n w/ pain: 146	Measured during study year 1 *Depressive symptoms* → Center for Epidemiological Studies Depression Scale → Patients categorized as: - Pain & depression (n = 54) - Pain alone (n = 92) - Depression alone (not included in review) - None (not included in review)	Database extraction Retrospective (during study duration = 3 y) - Number of inpatient admissions at SCI unit *(amount; hospitalizations)* - Number of inpatient days at the SCI unit *(amount; hospitalizations)* - Number of SCI service outpatient visits *(amount; consultations)* - Number of outpatient SCI psychologist visits *(amount; consultations)*	To compare the listed HCU outcomes between patients from the different pain and depression groups while controlling for age, medical comorbidities and level of SCI. → ANCOVA w/ post hoc testing
Valdes (2015) [84]	UK	CS	People who had total knee or hip replacement Average time between replacement and baseline: 1.27 ± 2.1 y	852 43.3/56.7 73.7 ± 8.8 y	*Catastrophizing* → Pain Catastrophizing Scale (PCS) → Dichotomized to PCS < 9 and PCS ≥ 9	Patient-reported Retrospective (current use) - Taking opioids *(type; opioid use)* - Taking strong opioids *(type; opioid use)* - Taking weak opioids *(type; opioid use)* - Taking non-steroidal anti-inflammatory drugs *(type; pain medication use)* - Taking other prescription medication for pain *(type; prescription pain medication)* - Not taking any pain medication *(type; pain medication use)* → yes/no for all	To investigate the influence of presence of pain catastrophizing (reference: no catastrophizing) on the listed HCU outcomes while also accounting for age, sex, BMI, back pain, WOMAC pain and body pain. → Logistic regression
van Tilburg (2008) [115]	US	C	Functional bowel disorders (IBS, functional diarrhea, functional constipation and functional abdominal pain)	1012 24.5/75.5 53.5 ± 14.0 y	*General anxiety symptoms* → Brief Symptom Inventory-18 - Anxiety subscale *Depressive symptoms* → Brief Symptom Inventory-18 - Depression subscale	Patient-reported Retrospective (past 3 m; assessed 6 m after index visit) Use of CAM (ginger root or tea, fennel seed, senna tea, psychotherapy, homeopathic, hypnotherapy, massage therapy, biofeedback, acupuncture, yoga, aromatherapy, evening primrose oil and others) → yes/no *(type; CAM use)*	To compare level of depressive and anxiety symptoms between patients using and not using CAM services. → *t*-tests
							To investigate the influence of level of depressive and anxiety symptoms on the likelihood of using CAM services (reference: no use of CAM) while also accounting for age, sex, education, marital status, IBS severity, distention symptoms, constipation symptoms, diarrhea symptoms, somatization, quality of life, pharmacy costs, lower gastro-intestinal costs, total healthcare expenditure, non-prescription costs, satisfaction w/ care, satisfactory relief of bowel symptoms and remarkable change in bowel symptoms. → Logistic regression Independent variables in the model were selected based on their significance in univariate analyses.
Vervoort (2019) [116]	NL	C	Fibromyalgia	199 5/95 43 y (range: 18–72 y)	Assessed at baseline *General anxiety symptoms* → Hospital Anxiety and Depression Scale - Anxiety subscale *Depressive symptoms* → Hospital Anxiety and Depression Scale - Depression subscale *Helplessness* → Illness Cognition Questionnaire - Helplessness subscale *Negative consequences beliefs* → Revised Fibromyalgia Illness Perception Questionnaire - Consequences subscale *Negative illness beliefs* → Revised Fibromyalgia Illness Perception Questionnaire - Acute/chronic timeline and cyclical timeline subscales *Psychological distress* → Revised Fibromyalgia Illness Perception Questionnaire - Emotional representations subscale *Illness coherence* → Revised Fibromyalgia Illness Perception Questionnaire - Coherence subscale *Pain acceptance* →Illness Cognition Questionnaire –Acceptance subscale *Perceived benefits* → Illness Cognition Questionnaire - Perceived benefits subscale *Perceived symptom control* → Revised Fibromyalgia Illness Perception Questionnaire - Personal control and treatment control subscales	Patient-reported Retrospective (assessed 18 m after fibromyalgia diagnosis for the past 6 m) Recurrent secondary HCU (yes/no) at 18 m post diagnosis: - Consultations w/ specialists - Diagnostic procedures - Admissions to healthcare institutions - Multimodal rehabilitation programs → Patients were considered a recurrent secondary healthcare user if secondary healthcare from at least 1 of the 4 categories was used in the past 6 m (dichotomized) *(type; secondary care consultations)*	To investigate the influence of the listed baseline CEF on the likelihood of being a recurrent secondary care user (reference: not a recurrent secondary care user) 18 m later. → Univariate logistic regression
							To investigate the influence of the listed baseline CEF on the likelihood of being a recurrent secondary care user (reference: not a recurrent secondary care user) 18 m later while also accounting for age, gender, education level, employment, comorbidity, severity of fibromyalgia, illness invalidation, pain coping and spouse response to well behaviors and pain behaviors. → Multivariate logistic regression Independent variables in the model were selected based on their significance in univariate analyses. Independent variables were omitted from the final model if not contributing significantly.
Villani (2010) ^5^ [85]	IT	CS	Migraine	465 Repeaters (n = 70): 18.6/81.4 36.4 ± 10.0 y Non-repeaters (n = 395): 18.5/81.5 34.4 ± 11.0 y	*General anxiety symptoms* → State-Trait Anxiety Inventory - State and trait form *Depressive symptoms* → Beck Depression Inventory	Patient-reported Retrospective (past 6 m) Number of ER visits → Categorized into 2 groups: - Repeaters: at least 3 ER visits, at least 1w apart during a 6 m period - Non-repeaters: all other migraine patients *(amount; emergency HCU)*	To investigate whether the listed CEF were influencing the likelihood of being a repeater of ER use (reference: no repeater). → Univariate logistic regression
Vina (2019) [86]	US	CS	Knee osteoarthritis with frequent pain	360 76.4/23.6 64.2 ± 8.8 y	*Depressive symptoms* → Patient Health Questionnaire-8 → Dichotomized to moderate to severe depressive symptoms vs. no or mild depressive symptoms	Patient-reported Prospective (current use) Use of the following analgesics for knee osteoarthritis: - acetaminophen - NSAIDs - COX-2 inhibitors - opioid medications → patients were subdivided into user categories: - oral opioids (w/ or w/o other oral analgesic treatments) *(type; opioid use)* - oral non-opioid analgesics *(type; pain medication use)* - no oral analgesic use	To investigate the influence of having moderate to severe depressive symptoms (reference: no depressive symptoms) on the likelihood of using non-opioid analgesics (reference: no oral analgesics) or oral opioids (reference: no oral analgesics and oral non-opioid analgesics). → Univariate multinomial regression
							To investigate the influence of having moderate to severe depressive symptoms (reference: no depressive symptoms) on the likelihood of using non-opioid analgesics (reference: no oral analgesics) or oral opioids (reference: no oral analgesics and oral non-opioid analgesics) while also accounting for age, sex, race income, WOMAC, comorbidity, BMI, social support and health literacy. → Multivariate multinomial regression
Von Korff (1991) ^5^ [87]	US	CS	Chronic pain → subdivided into: - Back pain - Headache - Abdominal pain - Chest pain - TMD pain Median pain duration: - Chest pain: 4 y - TMD: 6 y - Abdominal pain: 7 y - Back pain: 8 y - Headache: 12 y	Back pain n = 411 Headache n = 263 Abdominal pain n = 172 Chest pain n = 118 TMD pain n = 121 Chronic pain: 18–24 y: 9.7% 25–44 y: 64.1% 45–64 y: 18.6% 65+ y: 7.6% 41.6/58.4 TMD: 39 y 20/80	*Psychological distress* → Symptom Checklist Revised - Depression and anxiety subscales → Subdivided into 3 groups for Chi^2^ and logistic regression analyses: - Low psychological distress - Mild-moderate psychological distress - Severe psychological distress	Patient-reported Retrospective (past 6 m) Healthcare contact w/ doctor, PT, dentist, chiropractor or other professional for a pain problem → Dichotomized into seeking care for pain problem yes/no *(type; consultations)*	To compare the % of people reporting a consultation for pain between the 3 psychological distress groups in the listed pain samples. → Chi^2^
							To investigate whether level of psychological distress (reference: low distress) was influencing the likelihood of seeking care for pain (reference: no care seeking) while also accounting for age, sex, distant onset, persistent pain, pain severity and self-rated health. → Logistic regression
						Database extraction Retrospective (year before and after index visit) Total volume of ambulatory care (database extraction): count of primary care, specialty and emergency/walk-in visits (excluding optometry, PT, mental health and ancillary visits) → Dichotomized into seeking care for pain in general yes/no *(amount; consultations)*	To investigate the association between level of psychological distress and amount of healthcare use in the population sample and the TMD clinic sample. → Correlation
							To investigate whether level of psychological distress is influencing the amount of healthcare used while also accounting for age, sex, chronic pain status and self-rated health in the population sample and TMD clinic sample. → Multiple linear regression
Von Korff (2007) ^5^ [132]	US	CC	Back pain, headache and TMD pain	2010 Back pain (n = 807): 47/53 Headache (n = 831): 70/30 TMD pain (n = 372): 34/66	Assessed at baseline *Depressive symptoms* → Symptom Checklist - Depression subscale *Health worry* → 0–10 rating about the degree of worry about pain *Perceived symptom control* → 0–10 rating about perceived pain control *Self-efficacy beliefs* Readiness for self-management of pain → custom-made scale	Database extraction Prospective (3 y study duration) Number of ambulatory healthcare visits for acute disease, chronic disease or symptomatic/ill-defined conditions excluding the index pain condition → Categorized into: - Low frequency users (<12 visits) - High frequency users (≥12 visits) *(amount; consultations)*	To compare the listed CEF between high and low frequency users. → *t*-tests
Walker (2016) [88]	CA	CS	Women waiting for a gynecological surgery	590 0/100 48.3 ± 11.3 y	*Psychological distress* - Trait anxiety → State Trait Anxiety Inventory - Trait form - Depressive symptoms → Center for Epidemiologic Studies-Depression → Combined into 1 dummy: presence of anxiety and/or depression vs. no anxiety or depression	Patient-reported Retrospective (past 12 m) Pain-related HCU: number of visits to GP, specialists, walk-in clinics, ER or other healthcare professional → Transformed into: - GP visits: high (≥3 visits) vs. low (<3 visits) *(amount; consultations)* - Specialist visits: high (≥3 visits) vs. low (<3 visits) *(amount; consultations)* - Emergency HCU (walk-in clinic and ER visits): high (>0 visits) vs. low (0 visits) *(type; emergency HCU)*	To investigate the influencing of showing depressive and/or anxiety symptoms (reference: no symptoms) on the likelihood of the listed HCU outcomes. → Univariate logistic regression
							To investigate the influence of showing anxiety and/or depressive symptoms is influencing the likelihood on the listed HCU outcomes while also accounting for age, marital status, employment status, education, BMI, current smoker, previous abdominal surgery, waiting time before surgery, menstruation status, taking hormone replacement therapy, taking birth control pills, preoperative malignancy and pain intensity. → Multivariable logistic regression
Wideman (2011) [126]	CA	C	Patients w/ musculoskeletal back or neck injury (soft-tissue sprain or strain) undergoing a 7 w PT intervention Mean pain duration: 8.63 ± 3.35 w	202 39/61 36.57 ± 10.34 y	Measured directly after PT intervention *Depressive symptoms* → Beck Depression Inventory *Catastrophizing* → Pain Catastrophizing Scale *Fear-avoidance beliefs* Fear of movement → Tampa Scale of Kinesiophobia *Self-efficacy beliefs* Pain Self-efficacy → Pain Self-Efficacy Questionnaire	Patient-reported Retrospective (current use; assessed 1 y after baseline) Use of one of the following services for pain condition: PT, psychology, massage therapy and other medical services → yes/no for each, summed to a score between 0–4 (higher score indicates higher use of different services) *(amount; consultations)* Use of any of the following medications for pain condition: OTC NSAID’s, opioids, prescription anti-inflammatory drugs or psychotropic drugs → yes/no for each, summed to a score between 0–4 (higher score indicates higher use of different medications) *(amount; pain medication use)*	To investigate the association between the listed CEF and use of healthcare services and pain medication. → Correlations
							To investigate the influence of the level of the listed CEF on the likelihood of using healthcare services (reference: no use) and pain medication (reference: no use) while also accounting for age, sex, pain duration, pretreatment OTC NSAID use, pretreatment opioid use, pretreatment anti-inflammatory use and post-treatment pain intensity as independent variables. Independent variables in the model were selected based on their significance in univariate analyses.
Wijnhoven (2007) [89]	NL	CS	Musculoskeletal pain Pain duration: >3 m of pain in the last 12 m	2517 43/57 25–65 y Musculoskeletal pain was only present in a subsample (n not defined)	*Catastrophizing* → Pain Catastrophizing Scale → Subdivided into low, medium and high catastrophizing based on tertile scores	Patient-reported Retrospective (past 12 m) Contacts w/ GP, medical specialist or physiotherapist → Healthcare contact yes/no *(type; consultations)* Use of medicines for musculoskeletal pain → yes/no *(type; pain medication use)*	To investigate whether level of pain catastrophizing (reference: low catastrophizing) was influencing the likelihood of consulting a healthcare provider (reference: not consulting) and using pain medicines (reference: no use) while also accounting for age, household composition, educational level, smoking, overweight and physical activity. → Logistic regression Independent variables were omitted from the final model if not contributing significantly.
Williams (2006) [90]	US	CS	IBS Pain duration: repeated pain at least 12 w (not necessarily consecutive) in the past 12 m	337 29/71 <35 y: 8.3% 35–44 y: 23.1% 45–54 y: 37.7% ≥55 y: 30.9%	*Psychological distress* → K6 scale of non-specific psychological distress *Symptom-related anxiety symptoms* Fear that abdominal symptoms are related to cancer or other illness (instrument not clearly stated)	Patient-reported Retrospective (past 12 m) Having a doctor’s visit for abdominal symptoms → Dichotomized into healthcare seekers vs. non-seekers *(type; consultations)*	To compare level of psychological distress between healthcare seekers and non-seekers. → Wilcoxon two-sample test
							To investigate the influence of fear that symptoms are related to cancer or other serious illness on the likelihood of seeking healthcare (reference: not seeking care) in males and females. → Univariate logistic regression
Williams (2018) ^5^ [117]	US	C	Sickle cell disease	95 27.5 y (median; range: 18–58)	Assessed within 2 w after hospital/ER visit (study inclusion) for vaso-occlusive crisis in the 30 m period of HCU monitoring *General anxiety symptoms* → self-designed question *Depressive symptoms* → self-designed question	Database extraction Prospective (daily monitoring of files for a 30 m period) Frequency of: - ER visits *(amount; emergency HCU)* - Day hospital visits *(amount; consultations)* - Hospitalizations *(amount; hospitalizations)*	To compare the frequency of use of the listed healthcare services while controlling for study site between patients w/ and w/o depressive symptoms and w/ and w/o anxiety symptoms. → ANCOVA
Wong (2019) ^5^ [118]	US	C	Patients undergoing laparoscopic hysterectomy	125 46.5 ± 6.7 y 0/100	Assessed preoperatively *Depressive symptoms* → Patient Health Questionnaire-9 *General anxiety symptoms* → Generalized Anxiety Disorder-7 *Catastrophizing* → Pain Catastrophizing Scale	Patient-reported Retrospective (assessed at 1 and 2 w post-surgery for the entire 2 w postoperative period) Amount of opioids used in the acute post-operative period (2 w) → all reported use was transformed to morphine milligram equivalents for analyses *(amount; pain medication use)*	To investigate whether the listed preoperative CEF were associated with postoperative opioid use. → Spearman correlations
Woodhouse (2016) [119]	NO	C	Neck/low back pain Pain duration: <1 m of complaints	219 Conventional HCU (n = 93): 34/64 46 ± 11.9 y Alternative HCU (n = 18): 39/61 46 ± 11.5 y No HCU (n = 108): 45/55 46 ± 11.4 y	Assessed at baseline *General anxiety symptoms* → Hospital Anxiety and Depression Scale - Anxiety subscale *Depressive symptoms* → Hospital Anxiety and Depression Scale - Depression subscale → Both screen positive if score ≥ 8	Patient-reported Retrospective (past 1 m; assessed at 1 (baseline), 2, 3, 6 and 12 m after pain onset) - Use of pain medications - Contacts w/ healthcare providers for spinal pain (yes/no; if yes, which type of provider) → Results subdivided into: - Conventional care users (users of physicians, PT, chiropractors and psychologists; users of both conventional and alternative care; users of prescribed medications or patients on sick leave) - Alternative care users (users of osteopaths, naprapaths, homeopaths, acupuncturists or other alternative healthcare providers and alternative treatments) → Finally categorized into conventional care users vs. no conventional care use *(amount; HCU in general)*	To investigate if presence of baseline anxiety or depressive symptoms (reference: absence of symptoms) are significant predictors of future conventional care use (reference: no conventional care use) while controlling for age, sex, time of follow-up, marital status, work-related factors and socioeconomic status. → Logistic GEE regression
Zebenholzer (2016) [91]	AT	CS	Episodic and chronic headache	392 20.9/79.1	*Psychological distress* Anxiety and/or depressive symptoms → Hospital Anxiety and Depression Scale → Screens positive if HADS ≥ 8	Patient-reported Retrospective (past 12 m) Occurrence of healthcare consultations for headache: - Headache consultations (headache specialists, GP, PT, ER) → yes/no *(type; consultations)* - Headache-related examinations (MRI, CT, X-ray, eye test, blood tests) → yes/no *(type; consultations)* → Eurolight questionnaire	To compare rates of patients having a consultation or examination w/ a healthcare provider (reference: no consultation of examination) for headache between patients w/ and w/o depressive and/or anxiety symptoms. → Chi^2^
						Physician-reported Retrospective (the past) Use of prophylactic headache medications for ≥3 m → yes/no *(amount; pain medication use)*	To compare using prophylactic headache medications for ≥3 m (reference: shorter use) between patients w/ and w/o depressive and/or anxiety symptoms in patients presenting episodic and chronic headache, separately. → Chi^2^
Zondervan (2001) [92]	UK	CS	Chronic pelvic pain Pain duration: ≥6 m	475 Recent consulters (n = 153): 0/100 34.3 y Past consulters (n = 127): 0/100 37.7 y Non-consulters (n=195): 0/100 34.7 y	*Symptom-related anxiety symptoms* → Patient-reported self-designed question for pain anxiety	Patient-reported Retrospective (varying period, see below) - Consultation w/ GP or hospital doctor for any pelvic pain in the past 12 m → yes/no - Received a diagnosis or underwent an investigation for any pelvic pain in the past → yes/no → Categorized into: - Recent consulters (sought care in the past 12 m) - Past consulters (did not consult in the past 12 m but received a diagnosis or underwent an investigation in the past) -Non-consulters (never had a consultation, diagnosis or investigation for pelvic pain) *(amount; consultations)*	To investigate differences in the proportion of patients reporting pain anxiety symptoms in the 3 consulter groups. → Chi^2^

^1^ Unless otherwise mentioned. ^2^ Only reported if the assessment of CEF and HCU occurred on a different timepoint. ^3^ Patient-reported/Clinician-reported/Database extraction; retrospective/prospective. ^4^ Multivariate analyses: All considered independent factors for the multivariate model, including potential rules for exclusion from the model, were reported, if clearly mentioned in the original article. ^5^ Study rated as “high risk of bias”. **Abbreviations**: C: country (vide infra); D: study design (vide infra); n: sample size; SD: standard deviation; CEF: cognitive and emotional factor(s); HCU: healthcare utilization; m: month(s); y; year(s); PT: physical therapist/-y/physiotherapist; CAM: complementary and alternative medicine; TCA: tricyclic antidepressants; OTC: over-the-counter; TENS: transcutaneous electrical nerve stimulation; MS: multiple sclerosis; ROM: range of motion; OT: occupational therapist; ER: emergency room; w/: with; w/o: without; MD: medical doctor; GERD: gastroesophageal reflux disease; IHD; ischemic heart disease; IQR: interquartile range; GP: general practitioner; d: day(s); TMD: temporo-mandibular disorders; CBT: cognitive behavioral therapy; MED: morphine equivalent dose; VAS: visual analogue scale; PTSD: post-traumatic stress disorder; NSAID: non-steroidal anti-inflammatory drugs; BMI: body mass index; Q: quartile; IBS: irritable bowel syndrome; h: hours; ADL: activities of daily living; TNF: tumor necrosis factor; BZD: Benzodiazepine; WOMAC: Western Ontario and McMaster Universities Osteoarthritis Index; w: week(s); GEE: generalized estimating equations; MRI: magnetic resonance imaging; CT: computed tomography. **Countries** (ISO land codes): US: United States; UK: United Kingdom; NL: The Netherlands; SE: Sweden; CA: Canada; ES: Spain; DE: Germany; DK: Denmark; CH: Swiss; AU: Australia; JP: Japan; IT: Italy; NO: Norway; AT: Austria. **Study designs**: CS: cross-sectional study; C: cohort study; RCT: randomized controlled trial; CC: case-control study.

## Appendix C

**Table A3:** Complete risk of bias assessment based on a modified version of the Downs and Black Checklist.

	Reporting									External Validity									Internal Validity									
	Hypothesis	Main Outcomes	Patient Characteristics	Interventions	Distributions of Principal Confounders	Findings	Estimates of Random Variability	Characteristics of Patients LTFU	Actual Probability Values	Representativeness of Patients Asked	Representativeness of Included Patients	Representativeness of Treatment Accommodation	Blinding of Study Subjects	Blinding of Assessor	Data Dredging	Adjustment for Different Follow-Up Duration	Appropriateness of Statistics	Compliance with Intervention	Outcome Measures Valid/Reliable	HCU Primarily Registered for Scientific Research	Cases and Control from the Same Population	Cases and Controls Recruited over Same Period	Study Subjects Randomized to Intervention Groups	Randomized Intervention Assignment Concealed	Adjustment for Confounding	Losses of Patients or Missing Data Taken into Account	A priori Sample Size Calculation	Total
**Cross-Sectional Studies**																												**…/16**
Alschuler (2012) [48]	1	1	1	NA	0	1	1	NA	0	1	0	NA	NA	NA	1	NA	1	NA	1	1	NA	NA	NA	NA	0	1	0	11/16
Asmundson (2001) [49]	1	1	1	NA	0	1	1	NA	1	1	0	NA	NA	NA	1	NA	0	NA	1	0	NA	NA	NA	NA	0	1	0	10/16
Biggs (2003) [50]	1	1	1	NA	1	1	1	NA	1	1	1	NA	NA	NA	1	NA	1	NA	1	0	NA	NA	NA	NA	1	0	0	13/16
Boyer (2009) [51]	1	1	1	NA	1	1	1	NA	0	0	0	NA	NA	NA	1	NA	1	NA	1	1	NA	NA	NA	NA	0	0	0	10/16
Cronin (2018) [93]	1	1	1	NA	1	1	1	NA	1	1	0	NA	NA	NA	1	NA	1	NA	1	0	NA	NA	NA	NA	1	1	0	13/16
Cronin (2019) [52]	1	1	1	NA	1	1	1	NA	1	1	0	NA	NA	NA	1	NA	1	NA	1	1	NA	NA	NA	NA	1	1	1	15/16
de Boer (2012) [53]	1	1	1	NA	1	1	1	NA	0	1	0	NA	NA	NA	1	NA	1	NA	1	1	NA	NA	NA	NA	1	0	0	12/16
Elander (2003) [54]	1	1	1	NA	1	1	0	NA	0	1	1	NA	NA	NA	0	NA	0	NA	1	1	NA	NA	NA	NA	0	1	0	8/16
Elander (2014) [55]	1	1	1	NA	1	1	1	NA	0	0	0	NA	NA	NA	1	NA	1	NA	1	1	NA	NA	NA	NA	1	0	0	11/16
Fink-Miller (2014) [56]	1	1	1	NA	1	1	0	NA	1	1	0	NA	NA	NA	1	NA	1	NA	1	0	NA	NA	NA	NA	1	0	0	11/16
Grant (2000) [57]	1	1	1	NA	1	1	1	NA	0	0	0	NA	NA	NA	1	NA	1	NA	1	1	NA	NA	NA	NA	1	0	0	11/16
Harding (2019) [58]	1	1	1	NA	1	1	1	NA	1	1	0	NA	NA	NA	1	NA	1	NA	1	1	NA	NA	NA	NA	1	0	0	13/16
Hill (2007) [59]	1	1	1	NA	1	1	1	NA	0	1	1	NA	NA	NA	1	NA	1	NA	1	1	NA	NA	NA	NA	1	1	0	14/16
Howell (1999) [60]	1	1	1	NA	1	1	1	NA	1	1	0	NA	NA	NA	1	NA	1	NA	1	1	NA	NA	NA	NA	1	0	0	13/16
Jöud (2017) [7]	1	1	1	NA	1	1	1	NA	1	1	0	NA	NA	NA	1	NA	1	NA	1	1	NA	NA	NA	NA	1	0	0	13/16
Kapoor (2014) [61]	1	1	1	NA	1	1	1	NA	1	1	1	NA	NA	NA	1	NA	1	NA	1	0	NA	NA	NA	NA	1	0	0	12/16
Kratz (2018) [62]	1	1	1	NA	1	1	1	NA	1	1	0	NA	NA	NA	1	NA	1	NA	1	1	NA	NA	NA	NA	1	0	0	12/16
Lee (2008) [63]	1	1	1	NA	1	1	1	NA	1	1	0	NA	NA	NA	1	NA	1	NA	1	1	NA	NA	NA	NA	1	0	1	14/16
Lozier (2018) [64]	1	1	1	NA	1	1	1	NA	1	1	0	NA	NA	NA	1	NA	1	NA	1	1	NA	NA	NA	NA	1	0	0	13/16
Macfarlane (1999) [65]	1	1	1	NA	1	1	1	NA	0	1	0	NA	NA	NA	1	NA	1	NA	1	1	NA	NA	NA	NA	1	1	0	13/16
Macfarlane (2003) [66]	1	1	1	NA	1	1	1	NA	1	1	0	NA	NA	NA	1	NA	1	NA	1	1	NA	NA	NA	NA	1	0	0	13/16
Mann (2017) [67]	1	1	1	NA	1	1	1	NA	1	1	0	NA	NA	NA	1	NA	1	NA	1	1	NA	NA	NA	NA	1	0	0	13/16
Mannion (2013) [68]	1	1	1	NA	1	1	1	NA	1	1	0	NA	NA	NA	1	NA	1	NA	1	1	NA	NA	NA	NA	1	0	0	12/16
McCracken (1997) [69]	1	1	1	NA	1	1	1	NA	1	1	0	NA	NA	NA	0	NA	1	NA	1	1	NA	NA	NA	NA	1	0	0	12/16
McCracken (2007) [70]	1	1	1	NA	1	1	1	NA	0	1	0	NA	NA	NA	1	NA	1	NA	1	1	NA	NA	NA	NA	1	0	0	12/16
Mourad (2016) [72]	1	1	1	NA	1	1	1	NA	1	1	0	NA	NA	NA	1	NA	1	NA	1	1	NA	NA	NA	NA	1	1	0	14/16
Mourad (2018) [71]	1	1	1	NA	1	1	1	NA	1	1	0	NA	NA	NA	1	NA	1	NA	1	1	NA	NA	NA	NA	0	1	0	13/16
Ndao-Brumblay (2010) [73]	1	1	1	NA	1	1	1	NA	0	1	0	NA	NA	NA	1	NA	1	NA	0	0	NA	NA	NA	NA	1	0	0	10/16
Newman (2018) [74]	1	1	1	NA	1	1	1	NA	1	0	0	NA	NA	NA	1	NA	1	NA	1	0	NA	NA	NA	NA	1	0	0	11/16
Nielsen (2015) [75]	1	1	1	NA	1	1	1	NA	0	1	0	NA	NA	NA	1	NA	1	NA	1	1	NA	NA	NA	NA	1	0	0	12/16
Pierce (2019) [76]	1	1	1	NA	1	1	1	NA	1	1	0	NA	NA	NA	1	NA	1	NA	1	0	NA	NA	NA	NA	1	0	0	12/16
Rosenberg (2008) [77]	1	1	1	NA	1	1	1	NA	1	1	0	NA	NA	NA	1	NA	1	NA	1	1	NA	NA	NA	NA	1	0	0	13/16
Shmagel (2016) [78]	1	0	1	NA	1	1	1	NA	1	1	1	NA	NA	NA	1	NA	1	NA	1	1	NA	NA	NA	NA	1	0	0	13/16
Talley (1998) [79]	1	1	1	NA	1	1	1	NA	1	1	1	NA	NA	NA	1	NA	1	NA	1	1	NA	NA	NA	NA	1	0	0	14/16
Thorstensson (2009) [80]	1	1	1	NA	1	1	1	NA	0	1	1	NA	NA	NA	1	NA	1	NA	1	1	NA	NA	NA	NA	1	1	0	14/16
Torrance (2013) [81]	1	1	1	NA	1	1	1	NA	1	1	1	NA	NA	NA	1	NA	1	NA	1	1	NA	NA	NA	NA	0	0	0	13/16
Trask (2001) [82]	1	1	1	NA	1	1	1	NA	0	1	1	NA	NA	NA	0	NA	1	NA	0	0	NA	NA	NA	NA	0	0	0	9/16
Tsuji (2019) [83]	1	1	1	NA	1	1	1	NA	1	1	1	NA	NA	NA	1	NA	1	NA	1	1	NA	NA	NA	NA	1	0	0	14/16
Valdes (2015) [84]	1	1	1	NA	1	1	1	NA	1	0	0	NA	NA	NA	0	NA	1	NA	1	1	NA	NA	NA	NA	1	0	0	11/16
Villani (2010) [85]	0	1	1	NA	1	1	1	NA	0	1	0	NA	NA	NA	1	NA	1	NA	1	0	NA	NA	NA	NA	1	0	0	10/16
Vina (2019) [86]	1	1	1	NA	1	1	1	NA	1	1	1	NA	NA	NA	1	NA	1	NA	1	1	NA	NA	NA	NA	1	0	0	14/16
Von Korff (1991) [87]	1	1	1	NA	1	1	0	NA	0	1	0	NA	NA	NA	1	NA	1	NA	1	1	NA	NA	NA	NA	1	0	0	11/16
Walker (2016) [88]	1	1	1	NA	1	1	1	NA	0	0	0	NA	NA	NA	1	NA	1	NA	1	1	NA	NA	NA	NA	1	0	0	11/16
Wijnhoven (2007) [89]	1	1	1	NA	1	1	1	NA	0	1	0	NA	NA	NA	1	NA	1	NA	1	1	NA	NA	NA	NA	1	0	0	12/16
Williams (2006) [90]	1	1	1	NA	1	1	1	NA	1	1	1	NA	NA	NA	1	NA	1	NA	1	1	NA	NA	NA	NA	1	0	0	14/16
Zebenholzer (2016) [91]	1	1	1	NA	1	1	1	NA	1	1	1	NA	NA	NA	1	NA	1	NA	1	1	NA	NA	NA	NA	0	0	0	13/16
Zondervan (2001) [92]	1	1	1	NA	1	1	1	NA	1	1	1	NA	NA	NA	1	NA	1	NA	1	1	NA	NA	NA	NA	1	0	1	15/16
**Observational Cohort Studies**																												**…/18**
Buse (2012) [94]	1	1	1	NA	1	1	1	0	0	1	0	NA	NA	NA	1	1	1	NA	1	1	NA	NA	NA	NA	1	0	0	13/18
Carroll (2016) [96]	1	1	1	NA	1	1	1	1	0	1	0	NA	NA	NA	1	1	0	NA	1	0	NA	NA	NA	NA	0	1	0	12/18
Carroll (2018) [95]	1	1	1	NA	1	1	1	0	0	0	0	NA	NA	NA	1	0	1	NA	1	0	NA	NA	NA	NA	1	0	0	10/18
Citero (2007) [97]	1	1	1	NA	1	1	1	1	1	1	0	NA	NA	NA	1	0	1	NA	1	1	NA	NA	NA	NA	0	0	0	13/18
Demmelmaier (2010) [98]	1	1	1	NA	0	1	1	0	0	1	1	NA	NA	NA	1	0	1	NA	1	1	NA	NA	NA	NA	0	0	0	11/18
Dobkin (2006) [99]	1	1	1	NA	1	1	1	0	0	1	0	NA	NA	NA	1	0	1	NA	1	1	NA	NA	NA	NA	0	0	0	11/18
Engel (1996) [100]	1	1	1	NA	1	1	1	0	0	1	0	NA	NA	NA	1	1	1	NA	1	0	NA	NA	NA	NA	1	0	0	12/18
Gebauer (2019) [101]	1	1	1	NA	1	1	1	0	1	1	0	NA	NA	NA	1	0	1	NA	1	0	NA	NA	NA	NA	1	1	0	13/18
Gil (2004) [102]	1	1	1	NA	1	1	1	0	0	0	0	NA	NA	NA	1	1	1	NA	1	1	NA	NA	NA	NA	1	1	0	13/18
Hadlandsmyth (2013) [103]	1	1	1	NA	0	1	1	1	1	0	0	NA	NA	NA	1	0	1	NA	1	1	NA	NA	NA	NA	0	0	0	11/18
Jordan (2006) [104]	1	1	1	NA	1	1	1	0	0	1	0	NA	NA	NA	1	1	1	NA	1	0	NA	NA	NA	NA	1	0	0	12/18
Keeley (2008) [105]	1	1	1	NA	1	1	1	0	1	1	0	NA	NA	NA	1	0	1	NA	1	1	NA	NA	NA	NA	1	0	0	13/18
Kuijper (2014) [106]	1	1	1	NA	1	1	1	0	1	1	0	NA	NA	NA	1	0	1	NA	1	1	NA	NA	NA	NA	1	1	0	16/18
Lentz (2018) [107]	1	1	1	NA	1	1	1	1	1	1	1	NA	NA	NA	1	0	1	NA	1	1	NA	NA	NA	NA	1	1	1	17/18
Levenson (2008) [108]	1	1	1	NA	1	1	1	1	1	0	0	NA	NA	NA	1	0	1	NA	1	1	NA	NA	NA	NA	1	0	0	13/18
McCracken (2005; Pain) [109]	1	1	1	NA	1	1	1	1	0	1	1	NA	NA	NA	0	0	1	NA	1	1	NA	NA	NA	NA	1	0	0	13/18
Musey (2018) [110]	1	1	1	NA	1	1	1	0	1	0	0	NA	NA	NA	1	0	1	NA	1	0	NA	NA	NA	NA	1	0	1	12/18
Navabi (2018) [111]	1	1	1	NA	1	1	1	0	0	1	0	NA	NA	NA	1	0	1	NA	1	0	NA	NA	NA	NA	1	0	0	11/18
Pagé (2019) [112]	0	1	1	NA	1	1	1	0	1	1	1	NA	NA	NA	0	0	1	NA	1	1	NA	NA	NA	NA	1	0	0	12/18
Tremblay (2018) [113]	1	1	1	NA	1	1	1	1	1	1	0	NA	NA	NA	1	0	1	NA	1	1	NA	NA	NA	NA	1	0	0	14/18
Ullrich (2013) [114]	1	1	1	NA	1	1	1	0	0	1	1	NA	NA	NA	1	0	1	NA	1	0	NA	NA	NA	NA	1	0	0	12/18
Van Tilburg (2008) [115]	1	1	1	NA	1	1	1	0	0	1	1	NA	NA	NA	1	0	1	NA	1	1	NA	NA	NA	NA	1	0	0	12/18
Vervoort (2019) [116]	1	1	1	NA	1	1	1	1	1	1	0	NA	NA	NA	1	0	1	NA	1	1	NA	NA	NA	NA	1	1	0	15/18
Williams (2018) [117]	1	1	1	NA	1	1	1	0	1	1	0	NA	NA	NA	1	0	1	NA	0	1	NA	NA	NA	NA	1	0	0	12/18
Wong (2019) [118]	1	0	1	NA	1	1	0	0	0	1	0	NA	NA	NA	1	0	1	NA	1	1	NA	NA	NA	NA	0	1	0	10/18
Woodhouse (2016) [119]	1	1	1	NA	1	1	1	0	1	1	0	NA	NA	NA	1	0	1	NA	1	1	NA	NA	NA	NA	1	0	0	13/18
**Single-Group Interventional Cohort Studies**																												**…/21**
Ciechanowski (2003) [25]	1	1	1	0	0	1	1	1	0	1	1	0	NA	NA	1	0	1	0	1	1	NA	NA	NA	NA	1	1	0	14/21
Görge (2017) [120]	1	1	1	1	1	1	1	1	0	1	0	1	NA	NA	1	0	1	0	1	1	NA	NA	NA	NA	1	1	0	16/21
Huffman (2017) [121]	1	1	1	1	1	1	1	1	0	0	0	0	NA	NA	1	0	1	1	1	0	NA	NA	NA	NA	1	0	1	14/21
Jensen (1994) [128]	1	1	1	1	1	1	1	1	0	0	0	0	NA	NA	0	1	1	0	1	1	NA	NA	NA	NA	1	0	0	13/21
Jensen (2006) [122]	1	1	1	0	1	1	0	1	1	1	1	0	NA	NA	1	1	1	0	1	0	NA	NA	NA	NA	0	1	0	14/21
Kapoor (2012) [123]	1	1	1	0	1	1	1	0	1	1	0	1	NA	NA	1	0	1	1	1	0	NA	NA	NA	NA	1	0	0	13/21
McCracken (2005; Beh Res Ther) [124]	1	1	1	1	1	1	1	1	0	1	1	0	NA	NA	0	1	1	0	1	1	NA	NA	NA	NA	0	0	0	13/21
Osborne (2007) [129]	1	1	1	0	1	1	1	1	1	0	0	1	NA	NA	1	0	1	1	1	1	NA	NA	NA	NA	1	1	0	16/21
Philpot (2018) [125]	0	1	1	1	1	1	1	0	1	1	0	1	NA	NA	1	0	1	0	1	0	NA	NA	NA	NA	1	0	0	13/21
Primavera (1994) [127]	1	1	1	1	0	1	0	0	1	1	0	0	NA	NA	0	0	0	0	0	0	NA	NA	NA	NA	0	0	0	7/21
Wideman (2011) [126]	1	1	1	1	1	1	1	1	0	1	0	0	NA	NA	1	0	1	0	1	1	NA	NA	NA	NA	1	0	0	14/21
**Case-Control Studies**																												**…/19**
Harden (1997) [130]	1	0	1	NA	1	1	1	NA	0	0	0	NA	NA	0	1	NA	1	NA	1	0	1	1	NA	NA	1	0	1	12/19
Lozano-Calderon (2008) [131]	1	1	1	NA	1	1	1	NA	1	1	1	NA	NA	1	1	NA	1	NA	1	1	1	1	NA	NA	1	0	1	15/19
Von Korff (2007) [132]	1	1	1	NA	1	1	1	0	1	1	0	NA	NA	0	1	1	1	NA	1	0	1	0	NA	NA	0	0	0	13/19
**RCT and multiple-group cohort studies**																												**…/27**
Cronan (2002) [135]	1	1	1	0	0	1	1	0	1	1	0	0	0	0	1	0	1	0	1	0	1	0	1	0	1	0	0	13/27
Daltroy (1998) [133]	1	1	1	1	1	1	0	0	1	1	0	1	0	1	1	1	1	0	1	0	1	1	1	1	1	0	0	19/27
Durá-Ferrandis (2017) [134]	1	1	1	1	1	1	1	1	0	1	1	0	0	1	1	0	1	0	1	1	1	0	1	1	1	0	0	19/27

LTFU: loss to follow-up; HCU: healthcare use.

## Appendix D

**Table A4:** Associations between amount of HCU and CEF in people experiencing pain.

CEF	Type of HCU	Univariate Associations ^a^				Multivariate Associations ^a^			
		+	−	0	LoA ^b^	+	−	0	LoA ^b^
**MALADAPTIVE CEF CLUSTERS**									
Anger	Consultations					Görge [120]: 1		Görge [120]: 1	? <4
Anxiety symptoms (general)	Pain medication use	Elander [55]: 1 Levenson [108]: 1 Nielsen [75]: 1 Wong [118] ^c^: 1		Elander [55]: 1	++ 4/5 80%	Daltroy [133] ^c^: 1 Levenson [108]: 1			? <4
	Consultations	Hadlandsmyth [103]: 1	Philpot [125] ^c^: 2	Demmelmaier [98]: 2 Hadlandsmyth [103]: 1 Levenson [108]: 1 Lozier [64]: 1	00 1/8 13%	Hadlandsmyth [103]: 1	Philpot [125] ^c^: 1	Biggs [50]: 3 Philpot [125] ^c^: 1 Williams [117] ^c^: 1	00 1/7 14%
	Emergency HCU	Musey [110] ^c^: 1		Philpot [125] ^c^: 1 Villani [85] ^c^: 2	0 1/4 25%			Philpot [125] ^c^: 1 Williams [117] ^c^: 1	? <4
	Hospitalizations			Philpot [125] ^c^: 1	? <4	Daltroy [133] ^c^: 1		Philpot [125] ^c^: 1 Williams [117] ^c^: 1	? <4
	CAM use	Harding [58]: 1		Lozier [64]: 1	? <4			Harding [58]: 1	? <4
	HCU in general			Harding [58]: 1	? <4	Woodhouse [119]: 1		Harding [58]: 1	? <4
Anxiety symptoms (symptom-related)	Pain medication use	Elander [55]: 1		Elander [55]: 1	? <4			Carroll [95] ^c^: 1	? <4
	Consultations	Hadlandsmyth [103]: 2 Howell [60]: 3 Mourad [72]: 3 Tremblay [113]: 1 Zondervan [92]: 1		Carroll [95] ^c^: 1	++ 10/11 91%	Carroll [95] ^c^: 1 Görge [120]: 2 Hadlandsmyth [103]: 1 Howell [60]: 1 Mourad [72]: 1 Mourad [71]: 1 Tremblay [113]: 1		Biggs [50]: 3 Görge [120]: 1 Howell [60]: 2 Mourad [72]: 1 Mourad [71]: 1	? 8/16 50%
Catastrophizing	Pain medication use	Elander [55]: 1 Wideman [126]: 1 Wong [118] ^c^: 1		Elander [54]: 2 Elander [55]: 1	? 3/6 50%			Durá-Ferrandis [134]: 1 Wideman [126]: 1	? <4
	Consultations	Kapoor [123] ^c^: 2 Newman [74] ^b^: 1 Wideman [126]: 1	Jensen [128]: 1 Kapoor [61]: 1	Demmelmaier [98]: 2 Elander [54]: 1	? 4/9 44%			Ciechanowski [25]: 2 Kapoor [123] ^c^: 2 Kapoor [61]: 1 Newman [74] ^b^: 1 Wideman [126]: 1	00 0/7 0%
	Emergency HCU			CItero [97]: 4	00 0/4 0%			CItero [97]: 4	00 0/4 0%
	Hospitalizations			CItero [97]: 2	? <4			CItero [97]: 2	? <4
	HCU in general			CItero [97]: 2	? <4			CItero [97]: 2	? <4
Depressive symptoms	Pain medication use	Elander [55]: 1 Engel [100] ^c^: 1 Levenson [108]: 1 Nielsen [75]: 1 Wideman [126]: 1 Wong [118] ^c^: 1		Elander [55]: 1	++ 6/7 86%	Engel [100] ^c^: 1 Gil [102]: 1		Gil [102]: 2 Kratz [62]: 1 Levenson [108]: 1 Wideman [126]: 1	00 2/7 29%
	Consultations	Demmelmaier [98]: 1 Engel [100] ^c^: 2 Kapoor [123] ^c^: 1 Levenson [108]: 1 Lozier [64]: 1 Mann [67]: 1 Mourad [72]: 1 Newman [74] ^c^: 1 Tremblay [113]: 1 Tsuji [83]: 1 Von Korff [132] ^c^: 1 Wideman [126]: 1	Kapoor [61]: 1	Alschuler [48]: 7 Demmelmaier [98]: 1 Kapoor [123] ^c^: 1 Philpot [125] ^c^: 2	? 13/25 52%	Carroll [96]: 2 Gil [102]: 1 Görge [120]: 3 Kapoor [123] ^c^: 1 Kapoor [61]: 1 Newman [74] ^c^: 1 Shmagel [78]: 1 Tsuji [83]: 1 Ullrich [114]: 2		Alschuler [48]: 1 Biggs [50]: 3 Ciechanowski[25]: 2 Engel [100] ^c^: 2 Gil [102]: 2 Levenson [108]: 1 Lozier [64]: 1 Mann [67]: 1 Mourad [72]: 1 Mourad [71]: 1 Philpot [125] ^c^: 1 Tremblay [113]: 1 Wideman [126]: 1 Williams [117] ^c^: 1	? 13/32 41%
	Emergency HCU	Mann [67]: 1 Tsuji [83]: 1 Villani [85] ^c^: 1	Philpot [125] ^c^: 1	Alschuler [48]: 1 Levenson [108]: 2	? 3/7 43%	Tsuji [83]: 1	Philpot [125] ^c^: 1	Gil [102]: 3 Mann [67]: 1 Williams [117] ^c^: 1	00 1/7 14%
	Hospitalizations	Tsuji [83]: 1	Philpot [125] ^c^: 1	Levenson [108]: 1	? <4	Tsuji [83]: 1		Cronin [52]: 1 Gil [102]: 3 Philpot [125] ^c^: 1 Ullrich [114]: 2 Williams [117] ^c^: 1	00 1/9 11%
	CAM use	Harding [58]: 1		Alschuler [48]: 1 Lozier [64]: 1	? <4			Harding [58]: 1 Lozier [64]: 1	? <4
	HCU in general	Alschuler [48]: 1 Cronan [135] ^c^: 1		Alschuler [48]: 1 Cronan [135] ^c^: 1 Harding [58]: 1	? 2/5 40%	Görge [120]: 1 Woodhouse [119]: 1		Alschuler [48]: 2 Cronan [135] ^c^: 1 Grant [57]: 1 Harding [58]: 1	00 2/7 29%
Fear-avoidance beliefs	Pain medication use	Wideman [126]: 1			? <4			Wideman [126]: 1	? <4
	Consultations	Wideman [126]: 1		Demmelmaier [98]: 2	? <4	Keeley [105]: 1		Görge [120]: 1 Keeley [105]: 1 Wideman [126]: 1	00 1/4 25%
	HCU in general						Görge [120]: 1		? <4
Health worry	Consultations	Von Korff [132] ^c^: 1			? <4				
Helplessness	Consultations					Jensen [128]: 1			? <4
	HCU in general			Cronan [135] ^c^: 2	? <4			Cronan [135] ^c^: 1	? <4
Negative consequences beliefs	Consultations			Jensen [128]: 2	? <4			Biggs [50]: 3	? <4
Negative illness beliefs	Consultations			Jensen [128]: 3	? <4	Biggs [50]: 1		Biggs [50]: 2 Görge [120]: 1 Jensen [128]: 1	00 1/5 20%
	HCU in general					Görge [120]: 1			? <4
Psychological distress	Pain medication use			Trask [82]: 2 Zebenholzer [91]: 2	00 0/4 0%			Durá-Ferrandis [134]: 1	? <4
	Consultations	Lee [63]: 1 Navabi [111] ^c^: 1 Von Korff [87] ^c^: 4 Walker [88]: 2			+ 8/8 100%	Lee [63]: 1	Biggs [50]: 2	Biggs [50]: 1 Keeley [105]: 1 Kuijper [106]: 2 Von Korff [87] ^c^: 4 Walker [88]: 2	00 1/13 8%
	Emergency HCU	Navabi [111] ^c^: 1			? <4				
	Invasive procedures			Navabi [111] ^c^: 1	? <4				
	Hospitalizations	Navabi [111] ^c^: 1			? <4				
	HCU in general							Lentz [107]: 3	? <4
Stress	Pain medication use			Elander [55]: 2	? <4			Gil [102]: 3	? <4
	Consultations					Gil [102]: 1 Keeley [105]: 1		Gil [102]: 2 Keeley [105]: 1	? 2/5 40%
	Emergency HCU							Gil [102]: 3	? <4
	Hospitalizations							Gil [102]: 3	? <4
Symptom vigilance	Consultations	McCracken [69]: 1 Mourad [72]: 1		Demmelmaier [98]: 2	? 2/4 50%	McCracken [69]: 1			? <4
**POSITIVE CEF CLUSTERS**									
Pain acceptance	Pain medication use		Elander [55]: 1 McCracken [109]: 2	Elander [55]: 1 McCracken [109]: 1 McCracken [124]: 3	? 3/8 38%		Kratz [62]: 1 McCracken [109]: 1	Kratz [62]: 2 McCracken [109]: 1	? 2/5 40%
Perceived symptom control	Pain medication use							Daltroy [133] ^c^: 1 Durá-Ferrandis [134]: 1	? <4
	Consultations		Von Korff [132] ^c^: 1	Jensen [128]: 1	? <4			Biggs [50]: 3	? <4
	Hospitalizations							Daltroy [133] ^c^: 1	? <4
Positive mood	Pain medication use						Gil [102]: 1	Gil [102]: 2	? <4
	Consultations						Gil [102]: 1	Gil [102]: 2	? <4
	Emergency HCU						Gil [102]: 3		? <4
	Hospitalizations						Gil [102]: 2	Gil [102]: 1	? <4
Psychological flexibility	Pain medication use		McCracken [70]: 2		? <4		McCracken [70]: 1		? <4
	Consultations		McCracken [70]: 1		? <4		McCracken [70]: 1		? <4
Self-compassion	Pain medication use			Elander [55]: 2	? <4				
Self-efficacy beliefs	Pain medication use		Elander [55]: 1 Wideman [126]: 1	Elander [55]: 1	? <4		Nielsen [75]: 1 Wideman [126]: 1		? <4
	Consultations		Mann [67]: 1 Von Korff [132] ^c^: 1 Wideman [126]: 1	Demmelmaier [98]: 4 Lozier [64]: 1	? 3/8 38%		Mann [67]: 1	Lozier [64]: 1 Osborne [129]: 2 Wideman [126]: 1	00 1/5 20%
	Emergency HCU		Mann [67]: 1		? <4		Mann [67]: 1 Cronin [93] ^c^: 1		? <4
	Hospitalizations							Osborne [129]: 2	? <4
	CAM use			Lozier [64]: 1	? <4			Lozier [64]: 1 Osborne [129]: 1	? <4
	HCU in general		Cronan [135] ^c^: 2		? <4			Cronan [135] ^c^: 1	? <4
**OTHER CEF CLUSTERS**									
Health attributions	Pain medication use			Primavera [127] ^c^: 3	? <4				
	Hospitalizations			Primavera [127] ^c^: 3	? <4				
Locus of control	Consultations					Kuijper [106]: 1	Kuijper [106]: 1	Kuijper [106]: 4	00 1/6 17%

^a^ Name of the first author of the publication reporting positive, negative or absence of association(s) and the number of analyses investigating that particular association in the respective publication. ^b^ Level of association (LoA) was rated as follows: +/−: ≥60% of the analyses reported a +/− association; ?: 34–59% of the analyses reported a +/− association, or fewer than 4 studies investigated the association (<4); 0: ≤33% of the analyses reported an association; ++/−−/00: If after exclusion of high risk of bias studies the association (+/−) or absence of association (0) was still supported by, respectively, ≥ 60% or 0–33% of the analyses reporting a positive/negative association, the summary score was up/downgraded to ++/−−/00. ^c^ Study rated as “high risk of bias”. Abbreviations: CEF: cognitive emotional factor(s); HCU: healthcare utilization; LoA: level of association; CAM: complementary and alternative medicine.

**Table A5:** Associations between type of HCU (presence or absence of a certain type of HCU) and CEF in people experiencing pain.

CEF	Type of HCU	Univariate Associations ^a^				Multivariate Associations ^a^			
		+	−	0	LoA ^b^	+	−	0	LoA ^b^
**MALADAPTIVE CEF CLUSTERS**									
Anger	Prescription pain medication			Asmundson [49] ^c^: 1	? <4			Asmundson [49] ^c^: 1	? <4
	OTC pain medication			Asmundson [49] ^c^: 1	? <4			Asmundson [49] ^c^: 1	? <4
Anxiety symptoms (general)	Prescription pain medication	Pierce [76] ^c^: 1		Asmundson [49] ^c^: 1	? <4	Pierce [76] ^c^: 1		Asmundson [49] ^c^: 1	? <4
	OTC pain medication			Asmundson [49] ^c^: 1	? <4			Asmundson [49] ^c^: 1	? <4
	Opioids	Buse [94]: 3 Huffman [121] ^c^: 1		Harden [130] ^c^: 1 Jensen [122] ^c^: 1	+ 4/6 67%			Gebauer [101] ^c^: 2 Huffman [121] ^c^: 1	? <4
	Primary care consultations			Jordan [104] ^c^: 1	? <4			Jordan [104] ^c^: 1	? <4
	Secondary care consultations			Boyer [51]: 1 Vervoort [116]: 1	? <4			Vervoort [116]: 1	? <4
	Emergency HCU	Musey [110] ^c^: 1			? <4				
	CAM	van Tilburg [115]: 1			? <4	van Tilburg [115]: 1			? <4
Anxiety symptoms (symptom- related)	Prescription pain medication	Asmundson [49] ^c^: 1		Asmundson [49] ^c^: 2	? <4	Asmundson [49] ^c^: 1		Asmundson [49] ^c^: 2	? <4
	OTC pain medication			Asmundson [49] ^c^: 3	? <4	Asmundson [49] ^c^: 1		Asmundson [49] ^c^: 2	? <4
	Consultations	Williams [90]: 1		Williams [90]: 1	? <4				
	Primary care consultations	Howell [60]: 3		Macfarlane [65]: 1	++ 3/4 75%	Howell [60]: 1		Howell [60]: 2	? <4
	Invasive procedures			Lozano-Calderon [131]: 1	? <4				
Catastrophizing	Pain medication					de Boer [53]: 1 Valdes [84]: 1 Wijnhoven [89]: 2		de Boer [53]: 1 Valdes [84]: 1	++ 4/6 67%
	Prescription pain medication					Valdes [84]: 1			? <4
	Opioids	Jensen [122] ^c^: 1		Kapoor [61]: 1 Newman [74] ^c^: 1	? <4	Valdes [84]: 2		Valdes [84]: 1	? <4
	Consultations					Wijnhoven [89]: 2 Jöud [7]: 1			? <4
	Primary care consultations			Macfarlane [65]: 1	? <4				
	Secondary care consultations			Elander [54]: 1	? <4	de Boer [53]: 1		de Boer [53]: 1	? <4
	Tertiary care consultations	Fink-Miller [56] ^c^: 1			? <4	Fink-Miller [56] ^c^: 1			? <4
	Invasive procedures			Lozano-Calderon [131]: 1	? <4				
Depressive symptoms	Pain medication			Vina [86]: 1	? <4			Vina [86]: 1	? <4
	Prescription pain medication	Alschuler [48]: 1 Pierce [76] ^c^: 1		Alschuler [48]: 15 Asmundson [49] ^c^: 1	00 2/18 11%			Asmundson [49] ^c^: 1 Kratz [62]: 1 Pierce [76] ^c^: 1	? <4
	OTC pain medication	Alschuler [48]: 1		Alschuler [48]: 3 Asmundson [49] ^c^: 1	00 1/5 20%			Asmundson [49] ^c^: 1	? <4
	Opioids	Buse [94]: 3 Carroll [96] ^c^: 1 Huffman [121] ^c^: 1 Jensen [122] ^c^: 1 Vina [86]: 2		Harden [130] ^c^: 1 Kapoor [61]: 1 Newman [74] ^c^: 1	++ 8/11 73%	Gebauer [101] ^c^: 1 Vina [86]: 1		Gebauer [101] ^c^: 1 Huffman [121] ^c^: 1 Kratz [62]: 1 Vina [86]: 1	0 2/6 33%
	Primary care consultations	Pagé [112]: 2		Alschuler [48]: 4 Jordan [104] ^c^: 1	00 2/7 29%			Jordan [104] ^c^: 1	? <4
	Secondary care consultations	Vervoort [116]: 1		Boyer [51]: 1 Engel [100] ^c^: 1	? <4			Engel [100] ^c^: 1 Vervoort [116]: 1	? <4
	Tertiary care consultations			Fink-Miller [56] ^c^: 1	? <4				
	Invasive procedures	Alschuler [48]: 1		Alschuler [48]: 3 Lozano-Calderon [131]: 1	00 1/5 20%				
	Hospitalizations			Engel [100] ^c^: 1	? <4	Cronin [52]: 1			? <4
	CAM	Alschuler [48]: 3 Ndao-Brumblay [73] ^c^: 2 van Tilburg [115]: 1		Alschuler [48]: 18 Ndao-Brumblay [73] ^c^: 2 Pagé [112]: 2 Rosenberg [77]: 1	00 6/29 21%			Ndao-Brumblay [73] ^c^: 4 van Tilburg [115]: 1	0 0/5 0%
Fear-avoidance beliefs	Consultations	Mannion [68]: 2			? <4	Mannion [68]: 1		Mannion [68]: 1	? <4
Frustration	Pain medication	Hill [59]: 1			? <4	Hill [59]: 1			? <4
	Primary care consultations							Hill [59]: 1	? <4
Health worry	Primary care consultations			Macfarlane [65]: 2	? <4				
Helplessness	Secondary care consultations	Vervoort [116]: 1			? <4			Vervoort [116]: 1	? <4
Negative consequences beliefs	Pain medication	Hill [59]: 1			? <4	Hill [59]: 1			? <4
	Primary care consultations	Hill [59]: 1			? <4	Hill [59]: 1			? <4
	Secondary care consultations	Vervoort [116]: 1			? <4			Vervoort [116]: 1	? <4
Negative illness beliefs	Pain medication	Hill [59]: 1			? <4	Hill [59]: 1		Hill [59]: 1	? <4
	Consultations	Mannion [68]: 1			? <4			Mannion [68]: 1	? <4
	Primary care consultations	Hill [59]: 1			? <4	Hill [59]: 1		Hill [59]: 1	? <4
	Secondary care consultations			Vervoort [116]: 2	? <4				
Psychological distress	Pain medication	Hill [59]: 1			? <4	Hill [59]: 1			? <4
	Prescription pain medication	Navabi [111] ^c^: 1 Torrance [81]: 1			? <4			Navabi [111] ^c^: 1	? <4
	Opioids	Jensen [122] ^c^: 1 Navabi [111] ^c^: 1		Harden [130] ^c^: 1	? <4			Lentz [107]: 3 Navabi [111] ^c^: 1	00 0/4 0%
	Consultations			Macfarlane [66]: 1 Mannion [68]: 1 Talley [79]: 2 Von Korff [87] ^c^: 5 Williams [90]: 1 Zebenholzer [91]: 2	00 0/12 0%		Von Korff [87] ^c^: 1	Mannion [68]: 1 Thorstensson [80]: 2 Von Korff [87] ^c^: 4	0 1/8 13%
	Primary care consultations	Hill [59]: 1 Macfarlane [65]: 1 Trask [82]: 1			? <4	Hill [59]: 1 Macfarlane [65]: 1		Macfarlane [65]: 1 Thorstensson [80]: 1	? 2/4 50%
	Secondary care consultations			Vervoort [116]: 1	? <4			Lentz [107]: 3	? <4
	Tertiary care consultations			Dobkin [99]: 1	? <4				
	Emergency HCU	Walker [88]: 1			? <4	Walker [88]: 1		Lentz [107]: 3	00 1/4 25%
	Invasive procedures					Lentz [107]: 1 Navabi [111] ^c^: 1		Lentz [107]: 5	00 2/7 29%
	CAM			Trask [82]: 4	00 0/4 0%			Thorstensson [80]: 1	? <4
Symptom vigilance	Primary care consultations			Macfarlane [65]: 1	? <4				
Thanatophobia	Primary care consultations			Macfarlane [65]: 1	? <4				
**POSITIVE CEF CLUSTERS**									
Illness Coherence	Pain medication		Hill [59]: 1		? <4		Hill [59]: 1		? <4
	Primary care consultations							Hill [59]: 1	? <4
	Secondary care consultations			Vervoort [116]: 1	? <4				
Pain acceptance	Prescription pain medication						Kratz [62]: 1	Kratz [62]: 2	? <4
	Opioids						Kratz [62]: 2	Kratz [62]: 1	? <4
	Secondary care consultations			Vervoort [116]: 1	? <4				
Perceived benefits	Secondary care consultations			Vervoort [116]: 1	? <4				
Perceived symptom control	Pain medication		Hill [59]: 1		? <4	Hill [59]: 1		Hill [59]: 1	? <4
	Consultations		Macfarlane [66]: 1		? <4				
	Primary care consultations			Hill [59]: 1	? <4	Hill [59]: 1		Hill [59]: 1	? <4
	Secondary care consultations		Vervoort [116]: 1	Vervoort [116]: 1	? <4			Vervoort [116]: 1	? <4
	CAM	Ndao-Brumblay [73] ^c^: 3		Ndao-Brumblay [73] ^c^: 1	+ 3/4 75%	Ndao-Brumblay [73] ^c^: 3		Ndao-Brumblay [73] ^c^: 1	+ 3/4 75%
Self-efficacy beliefs	Prescription pain medication		Torrance [81]: 1		? <4				
	Secondary care consultations	Boyer [51]: 1		Boyer [51]: 3	00 1/4 25%				
	CAM			Rosenberg [77]: 1	? <4				
**OTHER CEF CLUSTERS**									
Locus of control	Secondary care consultations			Boyer [51]: 3	? <4				
Perceived cause of symptoms	Pain medication							Hill [59]: 1	? <4
	Primary care consultations							Hill [59]: 1	? <4

^a^ Name of the first author of the publication reporting positive, negative or absence of association(s) and the number of analyses investigating that particular association in the respective publication. ^b^ Level of association (LoA) was rated as follows: +/−: ≥60% of the analyses reported a +/− association; ?: 34–59% of the analyses reported a +/− association, or fewer than 4 studies investigated the association (<4); 0: ≤33% of the analyses reported an association; ++/−−/00: If after exclusion of high risk of bias studies the association (+/−) or absence of association (0) was still supported by, respectively, ≥60% or 0–33% of the analyses reporting a positive/negative association, the summary score was up/downgraded to ++/−−/00. ^c^ Study rated as “high risk of bias”. Abbreviations: CEF: cognitive emotional factor(s); HCU: healthcare utilization; LoA: Level of association; CAM: complementary and alternative medicine; OTC: over-the-counter.
