# Supplementary material for: The Relationship between Cognitive and Emotional Factors and Healthcare and Medication Use in People Experiencing Pain: A Systematic Review
Source: J Clin Med. 2020 Aug 3;9(8):2486. doi: 10.3390/jcm9082486 (PMC7464293; doi:10.3390/jcm9082486)
Supplement: Supplementary file 1 [file jcm-09-02486-s001.pdf]

Supplementary Material S1: Modified Downs and Black Checklist for risk of bias assessment

<u>Reporting</u>

1. Is the hypothesis/aim/objective of the study clearly described?

The question should be answered "Yes" if one of the items is clearly mentioned.

Yes = 1

No = 0

2. Are the main outcomes to be measured clearly described in the Introduction or Methods section?

If the main outcomes are first mentioned in the results section, the question should be answered "No". Focus should be on the outcome measures for healthcare use and cognitive and emotional factors. Is it clearly described what the outcome measure for healthcare use is containing? Are the cognitive and emotional factors clearly described?

Yes = 1

No = 0

3. Are the characteristics of the patients included in the study clearly described ?

In cohort studies and trials, inclusion and/or exclusion criteria should be given. In case-control studies, case-definition and the source of controls should be given. For cross-sectional studies descriptive statistics of relevant demographic variables should be reported in the methods or results.

Yes = 1

No = 0

4. Are the interventions of interest clearly described?

Treatments and placebo (where relevant) that are to be compared should be clearly described. This question should be answered "Not applicable (NA)" for studies without intervention (i.e., cross-sectional, observational cohort and case-control studies).

Yes = 1

No = 0

NA

5. Are the distributions of principal confounders in each group of subjects to be compared clearly described?

A list of principal confounders must be provided. Can be reported as a comparison of baseline data between two groups to be compared. The authors should clearly indicate the confounders, influential

factors, covariates and/or mediators, and not just present the baseline characteristics to describe the sample.

Yes = 1

No = 0

6. Are the main findings of the study clearly described?

Simple outcome data (including denominators and numerators) should be reported for all major findings so that the reader can check the major analyses and conclusions. (This question does not cover statistical tests which are considered below).

Yes = 1

No = 0

7. Does the study provide estimates of random variability in the data for the main outcomes?

In non-normally distributed data the inter-quartile range of results should be reported. In normally distributed data the standard error, standard deviation or confidence intervals should be reported. If the distribution of the data is not described, it must be assumed that the estimates used were appropriate and the question should be answered "Yes".
This question should be answered in particular for the outcome measures of interest for the present review question.

Yes = 1

No = 0

8. Have the characteristics of patients lost to follow-up been described?

This should be answered "Yes" if there were no losses to follow-up or where losses to follow-up were so small that findings would be unaffected by their inclusion. This should be answered "No" if a study does not report the number of patients lost to follow-up.
For studies without follow-up (i.e., cross-sectional and sometimes case-control studies), this question should be answered "Not applicable (NA)". If healthcare utilization is registered over a longer period, but there is only 1 moment of registration, it is still considered as 1 moment of assessment, and therefore no follow-up.

Yes = 1

No = 0

NA

9. Have actual probability values been reported (e.g. 0.035 rather than <0.05) for the main outcomes except where the probability value is less than 0.001?

This question should be answered in particular for the analyses relevant for this systematic review.

Yes = 1

No = 0

External validity

All the following criteria attempt to address the representativeness of the findings of the study and whether they may be generalized to the population from which the study subjects were derived.

10. Were the subjects asked to participate in the study representative of the entire population from which they were recruited?

The study must identify the source population and describe how the patients were selected. Patients are considered representative if they comprise the entire source population, an unselected sample of consecutive patients, or a random sample. Random sampling is only feasible if a list of all members of the relevant population exists. If a study does not report the proportion of the source population from which the patients are derived, the question should be answered as "Unable to determine". If an analysis was executed to investigate the comparison between patients asked and those of the population not asked to participate (e.g., when population data are available), and there appeared to be no differences between those two groups, the question should be answered "Yes".

Yes = 1

No = 0

Unable to determine = 0

11. Were those subjects who were prepared to participate representative of the entire population from which they were recruited?

The proportion of those who were asked and agreed to participate should be stated. Validation that the sample was representative would include demonstrating that the distribution of the main confounding factors was the same in the study sample and the source population.

If all consecutive patients agreed to participate, this question should be answered "Yes". If question 10 was answered "Yes" and there were no statistically significant differences between patients who agreed to participate and those who did not, this question should also be answered "Yes".

Yes = 1

No = 0

Unable to determine = 0

12. Were the staff, places, and facilities where the patients were treated, representative of the treatment the majority of patients receive?

For the question to be answered "Yes" the study should demonstrate that the intervention was representative of that in use in the source population. The question should be answered "No" if, for example, the intervention was undertaken in a specialist center unrepresentative of the hospitals most of the source population would attend. This question should be answered "Not applicable (NA)" for studies without intervention (i.e., cross-sectional, observational cohort and case-control studies).

Yes = 1

No = 0

Unable to determine = 0

NA

<u>Internal validity - bias</u>

13. Was an attempt made to blind study subjects to the intervention they have received?

For studies where the patients would have no way of knowing which intervention they received, this should be answered "Yes". For studies where blinding of patients is impossible due to the nature of the intervention/control intervention, the question should be answered "Not applicable (NA)". If the study did not comprise an intervention (i.e., cross-sectional, observational cohort and case-control studies) or there was no control group (i.e., single group interventional cohort studies), this question should be answered "Not applicable (NA)".

Yes = 1

No = 0

Unable to determine = 0

NA

14. Was an attempt made to blind those measuring the main outcomes of the intervention?

If the study did not comprise an intervention (i.e., cross-sectional, observational cohort and case-control studies) or there was no control group (i.e., single group interventional cohort studies), this question should be answered "Not applicable (NA)".

Yes = 1

No = 0

Unable to determine = 0

NA

15. If any of the results of the study were based on “data dredging”, was this made clear?

Any analyses that had not been planned at the outset of the study should be clearly indicated. If no retrospective unplanned subgroup analyses were reported, then answer “Yes”.

Yes = 1

No = 0

Unable to determine = 0

16. In trials and cohort studies, do the analyses adjust for different lengths of follow-up of patients, or in case-control studies, is the time period between the intervention and outcome the same for cases and controls ?

If follow-up was the same for all study patients the answer should be “Yes”. If different lengths of follow-up were adjusted for by, for example, survival analysis the answer should be “Yes”. Studies where differences in follow-up were ignored should be answered “No”. Also in studies with only one group of participants, follow-up should be more or less the same. For studies with only one moment of assessment (no follow-up) (i.e., cross-sectional and some case-control studies) this question should be answered “Not applicable (NA)”.

Yes = 1

No = 0

Unable to determine = 0

NA

17. Were the statistical tests used to assess the main outcomes appropriate?

The statistical techniques used must be appropriate for the data (distribution). For example nonparametric methods should be used for small sample sizes. Where little statistical analysis has been undertaken but where there is no evidence of bias, the question should be answered “Yes”. If the distribution of the data (normal or not) is not described it must be assumed that the estimates used were appropriate and the question should be answered “Yes”. If only very limited information is provided about the planned statistical analyses, the question should be answered “Unable to determine”.

Yes = 1

No = 0

Unable to determine = 0

18. Was compliance with the intervention(s) reliable?

Where there was non-compliance with the allocated treatment or where there was contamination of one group, the question should be answered “No”. For studies where the effect of any misclassification was likely to bias any association to the null, the question should be answered “Yes”. This question

should be answered "Not applicable (NA)" if the study did not include an intervention (i.e., cross-sectional, observational cohort and case-control studies).

Yes = 1

No = 0

Unable to determine = 0

NA

19. Were the main outcome measures used accurate (valid and reliable)?

For studies where the outcome measures are clearly described, the question should be answered "Yes". For studies which refer to other work or that demonstrate the outcome measures are accurate, the question should be answered as "Yes".

Yes = 1

No = 0

Unable to determine = 0

20. Was healthcare utilization primarily registered for scientific research?

For studies using secondary databases, the answer should be "No". For studies using self-reported methods, such as healthcare diaries or retrospective questionnaires, especially designed for the present study, the answer should be "Yes". If the study is a secondary analysis from another study, but healthcare use data were registered for scientific purposes, the question should be answered "Yes".

Yes = 1

No = 0

Unable to determine = 0

<u>Internal validity - confounding (selection bias)</u>

21. Were the patients in different intervention groups (trials and cohort studies) or were the cases and controls (case-control studies) recruited from the same population?

For example, patients for all comparison groups should be selected from the same hospital. The question should be answered "Unable to determine" for cohort and case-control studies where there is no information concerning the source of patients included in the study. This question should be answered "Not applicable (NA)" for cross-sectional and single group cohort studies.

Yes = 1

No = 0

Unable to determine = 0

NA

22. Were study subjects in different intervention groups (trials and cohort studies) or were the cases and controls (case-control studies) recruited over the same period of time?

For a study which does not specify the time period over which patients were recruited, the question should be answered "Unable to determine". This question should be answered "Not applicable (NA)" for cross-sectional and single group cohort studies.

Yes = 1

No = 0

Unable to determine = 0

NA

23. Were study subjects randomized to intervention groups?

Studies which state that subjects were randomized should be answered "Yes" except if the method of randomization would not ensure random allocation. For example alternate allocation would score "No" because it is predictable. This question should be answered "Not applicable (NA)" for studies without intervention (i.e., cross-sectional, observational cohort and case-control studies).

Yes = 1

No = 0

Unable to determine = 0

NA

24. Was the randomized intervention assignment concealed from both patients and health care staff until recruitment was complete and irrevocable?

If assignment was concealed from patients but not from staff, this question should be answered "No". This question should be answered "Not applicable (NA)" for studies without intervention (i.e., cross-sectional, observational cohort and case-control studies) and for single group studies (i.e., single group interventional cohort studies).

Yes = 1

No = 0

Unable to determine = 0

NA

25. Was there adequate adjustment for confounding in the analyses from which the main findings were drawn?

This question should be answered "No" for trials if: the main conclusions of the study were based on analyses of treatment rather than intention to treat; the distribution of known confounders in the

different treatment groups was not described; or the distribution of known confounders differed between the treatment groups but was not taken into account in the analyses. In nonrandomized studies if the effect of the main confounders was not investigated or confounding was demonstrated but no adjustment was made in the final analyses the question should be answered "No". If baseline differences were found between study groups, but the analyses did not control for these factors, the question should be answered "No". If the analyses did control for these factors the question should be answered "Yes". If it is unclear whether it was necessary to control for confounding factors, the question should be answered "Unable to determine".

Yes = 1

No = 0

Unable to determine = 0

NA

26. Were losses of patients to follow-up or missing data taken into account?

If the numbers of patients lost to follow-up are not reported, the question should be answered "Unable to determine". If the proportion lost to follow-up was too small to affect the main findings (< 5%), the question should be answered "Yes". If appropriate techniques were used to handle missing data and patients lost to follow-up in the analyses, with the exception of excluding patients due to missing data, the question should be answered "Yes".

Yes = 1

No = 0

Unable to determine = 0

NA

<u>Power</u>

27. Was an a priori sample size calculation performed and was the anticipated sample size reached, or was a post hoc power analysis performed which suggested that the results were sufficiently powered?

Yes = 1

No = 0

Unable to determine = 0

Table S2: Clustering of HCU outcome measures

Author (year)	HCU outcome measure	Category[1]	Subcategory[2]
Alschuler (2012) [1]	Number of visits with other healthcare providers for pain than primary care providers, MS specialists, other physicians, PT, OT, chiropractors, ER	Amount	Consultations
	Number of PT/OT visits	Amount	Consultations
	Number of primary care visits	Amount	Consultations
	Number of MS specialist visits	Amount	Consultations
	Number of other MD visits	Amount	Consultations
	Number of chiropractor visits	Amount	CAM use
	Number of ER visits	Amount	Emergency HCU
	Total number of visits	Amount	Consultations
	Total number of visits without PT/OT	Amount	Consultations
	Total number of pain treatments (see list of pain treatments below)	Amount	HCU in general
	Use of PT (yes/no)	Type	Primary care consultations
	Use of nerve blocks (yes/no)	Type	Invasive procedures
	Use of biofeedback/relaxation (yes/no)	Type	CAM use
	Acupuncture use (yes/no)	Type	CAM use
	Use of magnets (yes/no)	Type	CAM use
	Use of massage (yes/no)	Type	CAM use
	Use of hypnosis (yes/no)	Type	CAM use
	Use of counseling/psychotherapy (yes/no)	Type	Primary care consultations
	Mexiletine use (yes/no)	Type	Prescription pain medication use
	Neurontin use (yes/no)	Type	Prescription pain medication use
	TCA use (yes/no)	Type	Prescription pain medication use
	Narcotics use (yes/no)	Type	Prescription pain medication use
	Acetaminophen use (yes/no)	Type	OTC pain medication use
	Use of Advil, Aspirin, Aleve (yes/no)	Type	OTC pain medication use
	Use of Diazepam, Alprazolam (yes/no)	Type	Prescription pain medication use
	Tegretol use (yes/no)	Type	Prescription pain medication use
	Baclofen use (yes/no)	Type	Prescription pain medication use
	TENS unit use (yes/no)	Type	CAM use
	Use of Dilantin or other anticonvulsant (yes/no)	Type	Prescription pain medication use
	Chiropractic adjustment (yes/no)	Type	CAM use
	Use of heat (yes/no)	Type	CAM use

	Use of ice (yes/no)	Type	CAM use
	Marijuana use (yes/no)	Type	Prescription pain medication use
	Use of strengthening exercises (yes/no)	Type	CAM use
	Use of mobility exercises or ROM (yes/no)	Type	CAM use
	Implanted nerve stimulator (yes/no)	Type	Invasive procedure
	Implanted medication pump (yes/no)	Type	Invasive procedure
Asmundson (2001) [2]	OTC headache medication use (yes/no)	Type	OTC pain medication use
	Prescription headache medication use (yes/no)	Type	Prescription pain medication use
Biggs (2003) [3]	Number of consultations with healthcare providers	Amount	Consultations
	Number of GP visits	Amount	Consultations
	Number of consultations with other providers than GP	Amount	Consultations
Boyer (2009) [4]	Attending either a rheumatology setting or primary care setting	Type	Secondary care consultations
Buse (2012) [5]	Non-users, previous, current non-dependent and current probable dependent opioid use (yes/no for each)	Type	Opioid use
Carroll (2016) [6]	Patients on chronic opioid therapy vs not on chronic opioid therapy	Type	Opioid use
	Days with calls to healthcare providers	Amount	Consultations
	Days with medical visits	Amount	Consultations
Carroll (2018) [7]	Frequency of use of Sickle Cell Infusion Center	Amount	Consultations
	Opioid dose used	Amount	Pain medication use
Ciechanowski (2003) [8]	Having ≥weekly healthcare visits (reference: less)	Amount	Consultations
	Having ≥monthly healthcare visits (reference: ≥weekly)	Amount	Consultations
Citero (2007) [9]	Sum of hospitalizations, ER visits and ambulatory care visits	Amount	HCU in general
	Number of unscheduled doctor visits	Amount	Emergency HCU
	Number of ER visits	Amount	Emergency HCU
	Number of hospital admissions	Amount	Hospitalizations
Cronan (2002) [10]	Number of contacts, prescribed medical tests and medication at baseline and post-intervention → combined into 1 HCU outcome	Amount	HCU in general
Cronin (2018) [11]	Number of acute ER visits and hospitalizations for vaso-occlusive pain episodes → combined into 1 emergency HCU variable	Amount	Emergency HCU
Cronin (2019) [12]	Being hospitalized (yes/no)	Type	Hospitalizations
	Being readmitted to the hospital (yes/no)	Amount	Hospitalizations
Daltroy (1998) [13]	Length of stay	Amount	Hospitalizations
	Amount of postoperative pain medication use	Amount	Pain medication use
De Boer (2012) [14]	Specialist consultation (yes/no)	Type	Secondary care consultations
	Pain medication use (yes/no)	Type	Pain medication use

Demmelmaier (2010) [15]	Number of consultations with 6 different healthcare providers	Amount	Consultations
Dobkin (2006) [16]	Tertiary care use vs community patients	Type	Tertiary care consultations
Durá-Ferrandis (2017) [17]	Frequency of self-medication	Amount	Pain medication use
Elander (2003) [18]	Use of comprehensive care center or another hemophilia center (yes/no)	Type	Secondary care consultations
	Number of days when prescription medication was used	Amount	Pain medication use
	Number of days when OTC medication was used	Amount	Pain medication use
	Number of healthcare visits	Amount	Consultations
Elander (2014) [19]	Frequency of OTC pain killer use	Amount	Pain medication use
	Frequency of prescription pain medication use	Amount	Pain medication use
Engel (1996) [20]	Number of back pain primary care visits	Amount	Consultations
	Specialty care visits (yes/no)	Type	Secondary care consultations
	Number of radiologic procedures	Amount	Consultations
	Back pain admissions (yes/no)	Type	Hospitalizations
	Number of pain medicine fills	Amount	Pain medication use
Fink-Miller (2014) [21]	Primary vs tertiary care	Type	Tertiary care consultations
Gebauer (2019) [22]	Taking 1-50mg/day MED opioids vs none	Type	Opioid use
	Taking >50mg/day MED opioids vs none	Type	Opioid use
Gil (2004) [23]	Frequency of doctor calls on the same day, the next day or 2 days later	Amount	Consultations
	Frequency of hospitalizations on the same day, the next day or 2 days later	Amount	Hospitalizations
	Frequency of ER visits on the same day, the next day or 2 days later	Amount	Emergency HCU
	Frequency of prescription pain medication intake on the same day, the next day or 2 days later	Amount	Pain medication use
Görge (2017) [24]	Frequency of GP visits	Amount	Consultations
	Frequency of specialist visits	Amount	Consultations
	Frequency of PT visits	Amount	Consultations
	Frequency of psychotherapy visits	Amount	Consultations
	Total amount of HCU based on visits with GP, specialist, PT and psychotherapist, complementary therapist, massage therapist and hospital admissions	Amount	HCU in general
Grant (2000) [25]	Frequency of HCU (consultations with healthcare providers, ER visits, hospitalizations) at baseline and 6 months after rehabilitation	Amount	HCU in general
Hadlandsmyth (2013) [26]	Number of caregivers seen and frequency of treatment	Amount	Consultations

Harden (1997) [27]	Taking daily opioids vs not taking opioids	Type	Opioid use
Harding (2019) [28]	Number of different types of provider management (massage, osteopathic manipulation, trigger point injection, spine/joint/facet injections, spinal cord stimulation, counseling/talk therapy and surgery)	Amount	HCU in general
	Number of different types of self-management strategies (water therapy/swimming, other exercise, heat or cold therapy, TENS, ultrasound, brace or corset use, self-help books and relaxation)	Amount	CAM use
Hill (2007) [29]	Consultations with GP (yes/no)	Type	Primary care consultations
	Pain medication consumption (no/some)	Type	Pain medication use
Howell (1999) [30]	Frequency of GP visits	Amount	Consultations
	GP visits (yes/no)	Type	Primary care consultations
Huffman (2017) [31]	Chronic opioid therapy (no use/low dose/high dose)	Type	Opioid use
Jensen (1994) [32]	Number of pain-related physician visits	Amount	Consultations
Jensen (2006) [33]	Opioid use (yes/no)	Type	Opioid use
Jordan (2006) [34]	Primary care visits for knee pain (yes/no)	Type	Primary care consultations
Jöud (2017) [35]	Pain-related healthcare consultation (yes/no)	Type	Consultations
Kapoor (2012) [36]	Number of visits to rural healthcare center	Amount	Consultations
Kapoor (2014) [37]	Prescription of opioids (yes/no)	Type	Opioid use
	Total number of healthcare visits	Amount	Consultations
Keeley (2008) [38]	Total number of contacts with healthcare services (including hospitalizations etc.)	Amount	Consultations
Kratz (2018) [39]	Total number of pain medications used	Amount	Pain medication use
	Opioid use (yes/no)	Type	Opioid use
	Gabapentin use (yes/no)	Type	Prescription medication use
Kuijper (2014) [40]	Number of visits with healthcare providers for joint symptoms (GP, medical specialist, PT and alternative providers)	Amount	Consultations
Lee (2008) [41]	Number of GP visits for bowel symptoms	Amount	Consultations
Lentz (2018) [42]	Presence of HCU after PT treatment (yes/no)	Amount	HCU in general
	Presence of opioid use (yes/no)	Type	Opioid use
	Use of injections (yes/no)	Type	Invasive procedures
	Surgeries (yes/no)	Type	Invasive procedures
	diagnostic tests/imaging (yes/no)	Type	Secondary care consultations
	ER visits (yes/no)	Type	Emergency HCU
Levenson (2008) [43]	Number of scheduled physician visits	Amount	Consultations

	Number of ER visits	Amount	Emergency HCU
	Number of unscheduled physician	Amount	Emergency HCU
	Number of hospitalizations	Amount	Hospitalizations
	Amount of opioids used	Amount	Pain medication use
Lozano-Calderon (2008) [44]	Patients opting for surgery (yes/no)	Type	Invasive procedures
Lozier (2018) [45]	Frequency of use of clinician-directed NPTs (PT, TENS, chiropractic treatment, acupuncture, massage and psychoeducational courses (e.g. cognitive-behavioral therapy))	Amount	Consultations
	Frequency of use of self-directed NPTs (weight/strength training, yoga, tai chi, pool exercise/swimming and herbal medicine)	Amount	CAM use
Macfarlane (1999) [46]	Having a GP consultation for pain (yes/no)	Type	Primary care consultations
Macfarlane (2003) [47]	Having a consultation for orofacial pain (yes/no)	Type	Consultations
Mann (2017) [48]	Number of health-related visits for any reason to GP, specialist, walk-in clinic	Amount	Consultations
	Number of ER visits	Amount	Emergency HCU
Mannion (2013) [49]	LBP-related consultations to specialist, GP, physiotherapist or other practitioner (yes/no)	Type	Consultations
McCracken (1997) [50]	Number of physician visits	Amount	Consultations
McCracken (2005; Pain) [51]	Count of analgesic medications	Amount	Pain medication use
McCracken (2005; Beh Res Ther) [52]	Number of pain-related medication prescriptions	Amount	Pain medication use
McCracken (2007) [53]	Amount of strong opioid use	Amount	Pain medication use
	Number of types of pain medication used	Amount	Pain medication use
	Number of pain-related medical visits to GP, specialist and ER	Amount	Consultations
Mourad (2016) [54]	Number of healthcare visits (number of times the patient has visited a physician)	Amount	Consultations
Mourad (2018) [55]	Frequency of pain-related healthcare visits	Amount	Consultations
Musey (2018) [56]	ER visits (yes/no)	Type	Emergency HCU
	ER recidivism (count)	Amount	Emergency HCU
Navabi (2018) [57]	Opiate use (yes/no)	Type	Opioid use
	Corticosteroid use (yes/no)	Type	Prescription pain medication use
	Number of ER visits	Amount	Emergency HCU
	Number of hospital admissions	Amount	Hospitalizations
	Number of imaging studies	Amount	Consultations
	Number of surgeries	Amount	Invasive procedure

	History of surgery	Type	Invasive procedures
Ndao-Brumblay (2010) [58]	Use of biofeedback and relaxation (yes/no)	Type	CAM use
	Use of acupuncture (yes/no)	Type	CAM use
	Use of manipulation (yes/no)	Type	CAM use
	Use of CAM services in general (yes/no)	Type	CAM use
Newman (2018) [59]	Number of pain-related consultations	Amount	Consultations
	Opioid prescription (yes/no)	Type	Opioid use
Nielsen (2015) [60]	BZD use (number of days on which BZD's were used); transformed into past BZD users, current less than daily users and daily users	Amount	Pain medication use
Osborne (2007) [61]	Number of doctor visits	Amount	Consultations
	Number of PT visits	Amount	Consultations
	Number of CAM visits	Amount	CAM use
	Number of hospital admissions	Amount	Hospitalizations
	Length of stay	Amount	Hospitalizations
Pagé (2019) [62]	Using psychological treatment (yes/no)	Type	Primary care consultations
	Using self-management approaches (training in relaxation, meditation, hypnosis, visualization, distraction, self-help support group) (yes/no)	Type	CAM use
Philpot (2018) [63]	Decreases in primary care visits	Amount	Consultations
	Decreases in specialist visits	Amount	Consultations
	Decreases in hospitalizations	Amount	Hospitalizations
	Decreases in ER visits	Amount	Emergency HCU
Pierce (2019) [64]	BZD use (yes/no)	Type	Prescription pain medication use
Primavera (1994) [65]	Length of stay	Amount	Hospitalizations
	Amount of medication use	Amount	Pain medication use
Rosenberg (2008) [66]	CAM use (acupuncture/acupressure, chiropractic, aromatherapy, vitamin and mineral supplements, meditation/yoga, garlic preparations, traditional Chinese medicine, cod liver oil, massage, primrose oil, herbs, reflexologists, acupuncturists, root doctors, herbalists, chiropractors or other alternative practitioners) (yes/no)	Type	CAM use
Shmagel (2016) [67]	Number of healthcare visits	Amount	Consultations
Talley (1998) [68]	Visits to physicians and alternative therapists for abdominal pain or discomfort (Bowel Symptoms Questionnaire) (yes/no)	Type	Consultations
Thorstensson (2009) [69]	GP visits (yes/no)	Type	Primary care consultations
	Allied health professional visits (yes/no)	Type	Consultations

	Alternative therapist visits (yes/no)	Type	CAM use
	Combinations of visits with GP/allied health professionals/ alternative therapists (yes/no)	Type	Consultations
Torrance (2013) [70]	Adequate trial of neuropathic pain medications (yes/no)	Type	Prescription pain medication use
Trask (2001) [71]	Seeking psychological care (yes/no)	Type	Primary care consultations
	Biofeedback use (yes/no)	Type	CAM use
	Relaxation use (yes/no)	Type	CAM use
	Acupuncture use (yes/no)	Type	CAM use
	Chiropractor use (yes/no)	Type	CAM use
	Number of symptomatic medications used for headache	Amount	Pain medication use
	Number of preventive medications used for headache	Amount	Pain medication use
Tremblay (2018) [72]	Total number of healthcare visits (primary care, specialists and ER visits)	Amount	Consultations
Tsuji (2019) [73]	Number of physician visits	Amount	Consultations
	Number of ER visits	Amount	Emergency HCU
	Number of hospitalizations	Amount	Hospitalizations
Ullrich (2013) [74]	Number of inpatient admissions at the spinal cord injury unit	Amount	Hospitalizations
	Total number of inpatient days	Amount	Hospitalizations
	Number of spinal cord injury service outpatient visits	Amount	Consultations
	Number of outpatient spinal cord injury psychologist visits	Amount	Consultations
Valdes (2015) [75]	Taking opioids (yes/no)	Type	Opioid use
	Taking weak opioids (yes/no)	Type	Opioid use
	Taking strong opioids (yes/no)	Type	Opioid use
	Taking NSAID’s (yes/no)	Type	Pain medication use
	Other prescription medication use (yes/no)	Type	Prescription pain medication use
	Not taking any pain medication (yes/no)	Type	Pain medication use
Van Tilburg (2008) [76]	Use of CAM services (ginger root or tea, fennel seed, senna tea, psychotherapy, homeopathic, hypnotherapy, massage therapy, biofeedback, acupuncture, yoga, aromatherapy, evening primrose oil and others) (yes/no)	Type	CAM use
Vervoort (2019) [77]	Recurrent secondary HCU (specialist consultations, diagnostic procedures, admissions to healthcare institutions, multimodal rehabilitation programs) (yes/no)	Type	Secondary care consultations
Villani (2010) [78]	Number of emergency department visits → repeaters/non-repeaters	Amount	Emergency HCU

Vina (2019) [79]	Oral opioid use vs oral non-opioid analgesic use	Type	Opioid use
	Oral opioid use vs no oral analgesic use	Type	Opioid use
	Oral non-opioid analgesic use vs no oral analgesics use	Type	Pain medication use
Von Korff (1991) [80]	Healthcare contact with a doctor, PT, dentist, chiropractor or other professional for a pain problem (yes/no)	Type	Consultations
	Amount of ambulatory care (primary care, specialists, ER visits) for pain in general	Amount	Consultations
Von Korff (2007) [81]	Number of ambulatory healthcare visits	Amount	Consultations
Walker (2016) [82]	Number of GP visits (high vs low use)	Amount	Consultations
	Number of Specialist visits (high vs low use)	Amount	Consultations
	Urgent HCU (yes/no)	Type	Emergency HCU
Wideman (2011) [83]	Use of one of the following services for pain condition: PT, psychology, massage therapy and other medical services (yes/no for each; summed to a 0-4 score for use of different healthcare services)	Amount	Consultations
	Use of any of the following medications for pain condition: OTC NSAID's, opioids, prescription anti-inflammatory drugs or psychotropic drugs (yes/no for each; summed to a 0-4 score for use of different pain medications)	Amount	Pain medication use
Wijnhoven (2007) [84]	Contacts with GP, medical specialist or physiotherapist (yes/no)	Type	Consultations
	Use of medicines for musculoskeletal pain (yes/no)	Type	Pain medication use
Williams (2006) [85]	Having a doctor's visit for abdominal symptoms (yes/no)	Type	Consultations
Williams (2018) [86]	Frequency of ER visits	Amount	Emergency use
	Frequency of day hospital visits	Amount	Consultations
	Frequency of hospitalizations	Amount	Hospitalizations
Wong (2019) [87]	Amount of postoperative opioid use	Amount	Pain medication use
Woodhouse (2016) [88]	- conventional care (physicians, PT, chiropractors and psychologists; both conventional and alternative care; prescribed medications; sick leave)<br>- alternative care (osteopaths, naprapaths, homeopaths, acupuncturists or other alternative healthcare providers and treatments)<br>Categorized into conventional care users (yes/no)	Amount	HCU in general
Zebenholzer (2016) [89]	Consultations for headache (headache specialist, GP, hospital emergency room, nurse, PT)	Type	Consultations
	Examinations (MRI, CT, X-ray, eye test, blood tests)	Type	Consultations

	Intake of prophylactic medication for headache for ≥3m	Amount	Pain medication use
Zondervan (2001) [90]	Consultation with GP or hospital doctor for any pelvic pain (yes/no)<br>- Received a diagnosis or underwent an investigation for any pelvic pain in the past (yes/no)<br>→ Categorized into:<br>- Recent consulters (sought care in the past 12 m)<br>- Past consulters (did not consult in the past 12 m but received a diagnosis or underwent an investigation in the past)<br>- Non-consulters (never had a consultation, diagnosis or investigation for pelvic pain)	Amount	Consultations

[1]Two main categories of HCU outcomes: (1) amount or frequency of HCU and (2) type of HCU

[2]Subcategories for “Amount of HCU”: pain medication use, consultations, emergency HCU, hospitalizations, complementary and alternative medicine (CAM) use, invasive procedures and HCU in general (in case the study did not make any further specifications). Subcategories for “Type of HCU”: pain medication (in case no further specification was made), OTC pain medication, prescription pain medication (excluding opioids), opioids, consultations (in case no further specification was made), primary care consultations, secondary care consultations, tertiary care consultations, emergency HCU, invasive procedures, hospital admissions and CAM use. Outcome measures that combined consultations with hospitalizations were categorized as “HCU in general”, those combining consultations, CAM consultations and/or ER visits were categorized as “consultations”.

Abbreviations: HCU: healthcare use; MS: multiple sclerosis; PT: physical therapy/-ist; OT: occupational therapy/-ist; ER: emergency room; MD: medical doctor; CAM: complementary and alternative medicine; TCA: tricyclic antidepressants; TENS: transcutaneous electrical nerve stimulation; OTC: over-the-counter; ROM: range of motion; GP: general practitioner; NPT: non-pharmacological therapy; MED: morphine equivalent dose; BZD: benzodiazepine; NSAID: non-steroidal anti-inflammatory drug; m: month(s); vs: versus; mg: milligram(s); magnetic resonance imaging; CT: computed tomography

Table S3: Clustering of outcome measures for cognitive and emotional factors (CEF)

CEF cluster	Assessment tool	Author (year)
**Maladaptive clusters**		
Anger	Pain Coping Questionnaire – Anger subscale	Görge (2017) [24]
	State-Trait Anger Expression Inventory - Trait form	Asmundson (2001) [2]
Anxiety symptoms (general)	Brief symptom Inventory - Anxiety subscale	Van Tilburg (2008) [76]
	Depression, Anxiety and Stress Scale – Anxiety subscale	Elander (2014) [19]<br>Hadlandsmyth (2013) [26]<br>Huffman (2017) [31]
	Generalized Anxiety Disorder-7	Buse (2012) [5]<br>Levenson (2008) [43]<br>Lozier (2018) [45]<br>Nielsen (2015) [60]<br>Philpot (2018) [63]<br>Wong (2019) [87]
	Hospital Anxiety and Depression Scale – Anxiety subscale	Biggs (2003) [3]<br>Boyer (2009) [4]<br>Demmelmaier (2010) [15]<br>Jensen (2006) [33]<br>Jordan (2006) [34]<br>Musey (2018) [56]<br>Pierce (2019) [64]<br>Vervoort (2019) [77]<br>Woodhouse (2016) [88]
	NIH PROMIS Emotional distress – Anxiety subscale	Harding (2019) [28]
	Self-designed question(naire)	Gebauer (2019) [22]<br>Williams (2018) [86]
	State-Trait Anxiety Inventory – Trait form	Asmundson (2001) [2]<br>Harden (1997) [27]<br>Villani (2009) [78]
	State-Trait Anxiety Inventory – State form	Daltroy (1998) [13]<br>Villani (2009) [78]
Anxiety symptoms (symptom-related)	Albany Panic and Phobia Questionnaire – Interoceptive fear subscale	Hadlandsmyth (2013) [26]
	Body Sensations Questionnaire for fear of body sensations	Mourad (2016) [54]<br>Mourad (2018) [55]
	Cardiac Anxiety Questionnaire – Fear subscale	Mourad (2016) [54]

	Cardiac Anxiety Questionnaire – Total	Mourad (2016) [54]<br>Mourad (2018) [55]<br>Tremblay (2018) [72]
	Health Anxiety Questionnaire	Biggs (2003) [3]
	Illness attitude scale – Disease phobia subscale	Macfarlane (1999) [46]
	Pain Anxiety Symptoms Scale - Total	Carroll (2018) [7]<br>Elander (2014) [19]<br>Lozano-Calderon (2008) [44]
	Pain Anxiety Symptom Scale – Fearful appraisals of pain subscale	Asmundson (2001) [2]
	Pain Anxiety Symptom Scale – Pain-specific cognitive anxiety subscale	Asmundson (2001) [2]
	Pain Anxiety Symptom Scale – Pain-specific physiological anxiety subscale	Asmundson (2001) [2]
	Pain Coping Questionnaire – Pain-related anxiety subscale	Görge (2017) [24]
	Self-designed question for fear of serious illness	Howell (1999) [30]
	Self-designed question for fear that pain might be cancer	Howell (1999) [30]<br>Williams (2006) [85]
	Self-designed question for pain anxiety	Howell (1999) [30]<br>Zondervan (2001) [90]
Catastrophizing	Coping Strategies Questionnaire – Catastrophizing subscale	Ciechanowski (2003) [8]<br>Citero (2007) [9]<br>Demmelmaier (2010) [15]<br>Jensen (1994) [32]<br>Jensen (2006) [33]
	Haemophilia-adapted Coping Strategies Questionnaire - Negative thoughts subscale	Elander (2003) [18]
	Illness attitude scale – Hypochondriacal beliefs subscale	Macfarlane (1999) [46]
	Pain Catastrophizing Scale	de Boer (2012) [14]<br>Durá-Ferrandis (2017) [17]<br>Elander (2014) [19]<br>Fink-Miller (2014) [21]<br>Jöud (2017) [35]<br>Kapoor (2012) [36]<br>Kapoor (2014) [37]<br>Lozano-Calderon (2008) [44]<br>Newman (2018) [59]<br>Valdes (2015) [75]<br>Wideman (2011) [83]<br>Wijnhoven (2007) [84]

		Wong (2019) [87]
Depressive symptoms	Beck Depression Inventory	Asmundson (2001) [2]<br>Fink-Miller (2014) [21]<br>Harden (1997) [27]<br>Ndao Brumblay (2010) [58]<br>Pagé (2019) [62]<br>Villani (2009) [78]<br>Wideman (2011) [83]
	Brief Symptom Inventory - Depression subscale	van Tilburg (2008) [76]
	Center for Epidemiologic Studies Depression Scale	Carroll (2016) [6]<br>Ciechanowski (2003) [8]<br>Cronan (2002) [10]<br>Grant (2000) [25]<br>Kapoor (2012) [36]<br>Kapoor (2014) [37]<br>Lozano-Calderon (2008) [44]<br>Ullrich (2013) [74]
	Daily Mood Scale - Negative mood subscale	Gil (2004) [23]
	Depression, Anxiety and Stress Scale-21 – Depression subscale	Elander (2014) [19]<br>Huffman (2017) [31]
	Hospital Anxiety and Depression Scale – Depression subscale	Biggs (2003) [3]<br>Boyer (2009) [4]<br>Demmelmaier (2010) [15]<br>Jensen (2006) [33]<br>Jordan (2006) [34]<br>Pierce (2019) [64]<br>Tremblay (2018) [72]<br>Vervoort (2019) [77]<br>Woodhouse (2016) [88]
	NIH PROMIS Emotional Distress - Depression subscale	Harding (2019) [28]
	Pain Coping Questionnaire – Depression and helplessness subscale	Görge (2017) [24]
	Patient Health Questionnaire-2	Cronin (2019) [11]<br>Gebauer (2019) [22]
	Patient Health Questionnaire-8	Vina (2019) [79]

	Patient Health Questionnaire-9	Alschuler (2012) [1]<br>Buse (2012) [5]<br>Kratz (2017) [39]<br>Levenson (2008) [43]<br>Lozier (2018) [45]<br>Mann (2017) [48]<br>Mourad (2016) [54]<br>Mourad (2018) [55]<br>Newman (2018) [59]<br>Nielsen (2015) [60]<br>Philpot (2018) [63]<br>Shmagel (2016) [67]<br>Tsuji (2019) [73]<br>Wong (2019) [87]
	Self-designed question for depressive symptoms	Rosenberg (2008) [66]<br>Williams (2018) [86]
	Symptom Checklist-90 - Depression subscale	Engel (1996) [20]<br>Von Korff (2007) [81]
Fear-avoidance beliefs	Fear Avoidance Beliefs Questionnaire – Activity beliefs subscale	Görge (2017) [24]<br>Keeley (2008) [38]<br>Mannion (2013) [49]
	Fear Avoidance Beliefs Questionnaire – Work beliefs subscale	Keeley (2008) [38]<br>Mannion (2013) [49]
	Tampa Scale for Kinesiophobia	Demmelmaier (2010) [15]<br>Wideman (2011) [83]
Frustration	Arthritis Impact Measurement Scale – Frustration subscale	Hill (2007) [29]
Health worry	Illness attitude scale – Concerns about pain subscale	Macfarlane (1999) [46]
	Illness attitude scale – Worry about health subscale	Macfarlane (1999) [46]
	Numeric rating scale for perceived pain worry	Von Korff (2007) [81]
Helplessness	Arthritis Helplessness Index	Cronan (2002) [10]
	Illness Cognition Questionnaire – Helplessness subscale	Vervoort (2019) [77]
	Coping Strategies Questionnaire – Helplessness (factor created based on factor analysis of subscales)	Jensen (1994) [32]
Negative consequences of symptoms beliefs	Illness Perception Questionnaire – Consequences subscale	Biggs (2003) [3]
	Illness Perception Questionnaire-Revised – Consequences subscale	Hill (2007) [29]<br>Vervoort (2019) [77]

	Survey of Pain Attitudes – Disability beliefs subscale	Jensen (1994) [32]
	Survey of Pain attitudes – Harm subscale	Jensen (1994) [32]
Negative illness beliefs	Back Beliefs Questionnaire	Mannion (2013) [49]
	Control Beliefs Concerning Illness and Health Questionnaire - Fatalistic external locus of control subscale	Görge (2017) [24]
	Illness Perception Questionnaire – Timeline acute/chronic subscale	Biggs (2003) [3]
	Illness Perception Questionnaire-Revised – Timeline acute/chronic subscale	Hill (2007) [29]<br>Vervoort (2019) [77]
	Illness Perception Questionnaire-Revised – Timeline cyclical subscale	Hill (2007) [29]<br>Vervoort (2019) [77]
	Survey of Pain attitudes – Medical cure subscale	Jensen (1994) [32]
	Survey of Pain Attitudes – Medication beliefs subscale	Jensen (1994) [32]
	Survey of Pain Attitudes – Pain as illness belief (factor created based on factor analysis of subscales)	Jensen (1994) [32]
	Survey of Pain attitudes – Solicitude subscale	Jensen (1994) [32]
Psychological distress	Brief Symptoms Inventory-18	Durá-Ferrandis (2017) [17]<br>Trask (2001) [71]
	Combination of Center for Epidemiological Studies-Depression Scale and State Trait Anxiety Inventory – Trait form	Walker (2016) [82]
	EQ-5D - Anxiety/Depression subscale	Thorstensson (2009) [69]<br>Mannion (2013) [49]
	General Health Questionnaire-28	Lee (2008) [41]<br>Macfarlane (1999) [46]<br>Macfarlane (2003) [47]<br>Talley (1998) [68]
	Hospital Anxiety and Depression Scale – Total	Keeley (2008) [38]<br>Navabi (2018) [57]<br>Zebenholzer (2016) [89]
	Illness Perception Questionnaire-revised/brief – Emotional representations subscale	Hill (2007) [29]<br>Vervoort (2019) [77]
	K6 scale of non-specific psychological distress	Williams (2006) [85]
	Multidimensional Pain Inventory - Affective Distress Subscale	Harden (1997) [27]
	OSPRO-YF (shortened 10-item version + remaining items)	Lentz (2018) [42]
	SF-36 – Mental Health Subscale	Biggs (2003) [3]<br>Jensen (2006) [33]
	SF-36 – Mental component scale	Kuijper (2014) [40]

	SF-12 – Mental component scale	Torrance (2013) [70]
	Symptom Checklist 90-R	Dobkin (2006) [16]<br>Von Korff (1991) [80]
Stress	Depression, Anxiety and Stress Scale-21 – Stress subscale	Elander (2014) [19]
	Life Events and Difficulties Schedule – Back pain-related social stress subscale	Keeley (2008) [38]
	Life Events and Difficulties Schedule – Back pain-independent social stress subscale	Keeley (2008) [38]
	VAS for perceived level of overall stress of the day	Gil (2004) [23]
Symptom vigilance	Cardiac Anxiety Questionnaire – Heart-focused attention subscale	Mourad (2016) [54]
	Illness attitude scale – Bodily preoccupation subscale	Macfarlane (1999) [46]
	Pain Vigilance and Awareness Questionnaire	Demmelmaier (2010) [15]<br>McCracken (1997) [50]
Thanatophobia symptoms	Illness attitude scale – Thanatophobia subscale	Macfarlane (1999) [46]
**Positive clusters**		
Illness coherence	Illness Perception Questionnaire-Revised – Coherence subscale	Hill (2007) [29]<br>Vervoort (2019) [77]
Pain acceptance	Chronic Pain Acceptance Questionnaire – Total score	Elander (2014) [19]<br>Kratz (2018) [39]<br>McCracken (2005; Pain) [51]<br>McCracken (2005; Beh Res Ther) [52]
	Chronic Pain Acceptance Questionnaire – Pain willingness subscale	Kratz (2018) [39]<br>McCracken (2005; Pain) [51]<br>McCracken (2005; Beh Res Ther) [52]
	Chronic Pain Acceptance Questionnaire – Activities engagement subscale	Kratz (2018) [39]<br>McCracken (2005; Pain) [51]<br>McCracken (2005; Beh Res Ther) [52]
	Illness Cognition Questionnaire – Acceptance subscale	Vervoort (2019) [77]
Perceived benefits	Illness Cognition Questionnaire – Perceived benefits subscale	Vervoort (2019) [77]
Perceived symptom control	Illness Perception Questionnaire – Cure subscale	Biggs (2003) [3]
	Illness Perception Questionnaire-Revised – Personal control	Hill (2007) [29]<br>Vervoort (2019) [77]
	Illness Perception Questionnaire-Revised – Treatment control subscale	Hill (2007) [29]<br>Vervoort (2019) [77]
	Likert scale to assess pain control	Ndao- Brumblay (2010) [58]

	Numeric rating scale for perceived pain control	Von Korff (2007) [81]
	Self-designed question	Daltroy (1998) [13]
	Survey of pain attitudes – Perceived control subscale	Durá-Ferrandis (2017) [17]<br>Jensen (1994) [32]
Positive mood	Daily Mood Scale – Positive mood subscale	Gil (2004) [23]
Psychological flexibility	Brief Pain Coping Inventory-2 – Psychological flexibility subscale	McCracken (2007) [53]
Self-compassion	Self-Compassion Scale Short Form	Elander (2014) [19]
Self-efficacy beliefs	Arthritis Self-Efficacy Scale	Cronan (2002) [10]
	Chronic Pain Self-Efficacy Scale - Total	Boyer (2009) [4]
	Chronic Pain Self-Efficacy Scale – Self-efficacy for pain management subscale	Boyer (2009) [4]
	Chronic Pain Self-Efficacy Scale – Self-efficacy for symptoms management subscale	Boyer (2009) [4]
	Chronic Pain Self-Efficacy Scale – Self-efficacy for physical functioning subscale	Boyer (2009) [4]
	Custom-made scale for readiness for self-management of pain	Von Korff (2007) [81]
	Pain Self-Efficacy Questionnaire	Elander (2014) [19]<br>Lozier (2018) [45]<br>Mann (2017) [48]<br>Nielsen (2015) [60]<br>Rosenberg (2008) [66]<br>Torrance (2013) [70]<br>Wideman (2011) [83]
	Self-Efficacy Scale	Demmelmaier (2010) [15]
	Self-Efficacy for Exercise Scale	Demmelmaier (2010) [15]
	Sickle Cell Self-Efficacy Scale	Cronin (2018) [12]
	Stanford Scale	Osborne (2007) [61]
**Other clusters** (not classifiable as maladaptive or positive)		
Health attribution	Health Attribution Test	Primavera (1993) [65]
Locus of control	Multidimensional Pain Locus of Control Scale – Internal subscale	Boyer (2009) [4]<br>Kuijper (2014) [40]
	Multidimensional Pain Locus of Control Scale – External subscale	Kuijper (2014) [40]
	Multidimensional Pain Locus of Control Scale – Chance subscale	Boyer (2009) [4]<br>Kuijper (2014) [40]
	Multidimensional Pain Locus of Control Scale – Fate subscale	Boyer (2009) [4]
Perceived cause of symptoms	Illness Perception Questionnaire-Revised – Psychological attributions subscale (part of causes subscale)	Hill (2007) [29]

Abbreviations: CEF: cognitive and emotional factors

Table S4: Comprehensive overview of the results of analyses investigating associations between CEF and amount of HCU

**Author (year)**	**Sample**<br>n<br>Type of patients	**Outcome CEF**[1]	**Outcome HCU**[1]	**Investigated association**[2]	**U/M**	**Findings**[3]	**Level of association**[4]
**MALADAPTIVE CEF CLUSTERS**							
Anger x consultations							
Görge (2017) [24]	688<br>Chronic low back pain	Pain Coping Questionnaire – Anger subscale (FESV-AG) (baseline or change between baseline and immediately post-rehabilitation)	Number of psychotherapy visits 6m post-rehabilitation	Regression investigating the influence of change in FESV-AG score on the number of psychotherapy visits post-rehabilitation while also accounting for baseline psychotherapy visits (S), employment (NS), hours of work (NS), days on sick leave (S), disability (NS) and helplessness and depressive symptoms (S).	M	β=.088<br>NS	<u>Multivariate</u><br>?<br><4
			Number of GP visits 6m post-rehabilitation	Regression investigating the influence of baseline FESV-AG score on the number of GP visits post-rehabilitation while also accounting for baseline GP visits (S), hours of work (S), days on sick leave (S), state of heath (S), SF-12 physical component score (NS), chronicity (NS) and anxiety symptoms (NS).	M	β=.180<br>**p=.01**	
General anxiety symptoms x pain medication use							
Daltroy (1998)[5] [13]	222<br>Scheduled for knee or hip arthroplasty	State-Trait Anxiety Inventory – State form (baseline/ preoperative)	Postoperative pain medication use	General linear model investigating the influence of baseline state anxiety on postoperative pain medication use while also accounting for age (S), knee surgery (vs hip surgery) (S), poor preoperative sleep quality (S), surgeon (NS), information (NS) and relaxation training (NS).	M	**p<.051**	<u>Univariate</u><br>++<br>4/5 – 80%<br><br><u>Multivariate</u><br>?<br><4
Elander	112	Depression, Anxiety and	Frequency of prescription pain medication use	Correlation	U	r=.23<br>**p<.05**	

(2014) [19]	General population w/ pain	Stress Scale – Anxiety subscale	Frequency of OTC pain medication use	Correlation	U	r=-.10<br>NS	
Levenson<br>(2008) [43]	232<br>Sickle cell disease (SCD)	General Anxiety Disorder-7<br>→ Anxiety symptoms yes vs no<br>(baseline)	% days using opioids for SCD in 6m period from baseline	Comparison of the % of days using opioids between patients w/ and w/o anxiety symptoms.	U	positive association<br>**p<.05**	
				Comparison of the % of days using opioids between patients w/ and w/o anxiety symptoms controlling for age and income.	M	positive association<br>**p<.05**	
Nielsen<br>(2015) [60]	1,220<br>Chronic non-cancer pain	Generalized Anxiety Disorder-7<br>→ Anxiety symptoms yes vs no	4 categories of Benzodiazepine (BZD) use: no use; past use; current less than daily use; current daily use	Regression comparing the likelihood of having anxiety symptoms (reference: no symptoms) between patients from the different BZD use groups (reference: no use).	U	Past: OR: 1.46; 95%CI: 1.01-2.09<br><Daily: OR: 2.07; 95%CI: 1.36-3.13<br>Daily: OR: 3.22; 95%CI: 2.19-4.73<br>**p<.05**	
Wong<br>(2019)[5] [87]	125<br>Scheduled for hysterectomy	Generalized Anxiety Disorder-7<br>(preoperative)	Amount of postoperative opioid use	Correlation	U	positive association<br>**p<.001**	
General anxiety symptoms x consultations							
Biggs<br>(2003) [3]	151<br>Abdominal or chest pain	Hospital Anxiety and Depression Scale (HADS-A)	Number of consultations w/ healthcare providers	Regression investigating the influence of HADS-A score on the number of consultations while also accounting for education, access to confidant, pain score, recent social stress, exposure to death of a father or mother during childhood, reported childhood adversity (antipathy from father or mother, neglect, physical abuse or psychological abuse), depressive symptoms, symptom-related anxiety symptoms, negative illness perceptions (consequences and timeline), perceived symptom control, SF-36 scores (role limitations physical and mental, social function, energy and	M	NS (omitted from final model)	<u>Univariate</u><br>00<br>1/8 – 13% positive associations<br><br><u>Multivariate</u><br>00<br>1/7 – 14% positive associations

			vitality and pain) (all above NS – omitted from final model), sex (S), SF-36 scores (physical function, health perception and mental health) (S), marital status (S), diagnosis (S), death of a sibling (S) and reported sexual abuse (S).			
		Number of GP consultations	Regression investigating the influence of HADS-A score on the number of GP consultations while also accounting for education, access to confidant, pain score, recent social stress, exposure to death of a father or mother during childhood, reported childhood adversity (sexual abuse, antipathy from mother, neglect, physical abuse or psychological abuse), depressive symptoms, symptom-related anxiety symptoms, negative consequences beliefs, perceived symptom control, SF-36 scores (role limitations physical and mental, social function, energy and vitality, physical function, health perception and mental health) (all above NS – omitted from final model), sex (S), SF-36 pain score (S), marital status (S), illness perception timeline score (S), diagnosis (S), death of a sibling (S) and reported antipathy from father (S).	M	NS (omitted from final model)	
		Number of consultations w/ other providers than GP	Regression investigating the influence of HADS-A score on the number of consultations w/ other providers than GP while also accounting for marital	M	NS (omitted from final model)	

				status, diagnosis, education, access to confidant, recent social stress, exposure to death of a sibling, father or mother during childhood, reported childhood adversity (sexual abuse, antipathy from father or mother, neglect, physical abuse or psychological abuse), depressive symptoms, symptom-related anxiety symptoms, negative illness perceptions (consequences and timeline), perceived symptom control, SF-36 scores (pain score, role limitations physical and mental, social function, energy and vitality, physical function, health perception and mental health) (all above NS – omitted from final model), sex (S), SF-36 (mental health, health perception and physical function) (S) and pain score (S).		
Demmelmaier (2010) [15]	42<br>First-episode back pain	Hospital Anxiety and Depression Scale – Anxiety subscale (baseline)	Number of consultations w/ healthcare providers at follow-up	Correlation	U	NS
	271<br>Chronic back pain	Hospital Anxiety and Depression Scale – Anxiety subscale (baseline)	Number of consultations w/ healthcare providers at follow-up	Correlation	U	NS
Hadlandsmyth (2013) [26]	Baseline: 196<br>Follow-up: 70<br>Non-cardiac chest pain	Depression, Anxiety and Stress Scale – Anxiety subscale (baseline)	Frequency of healthcare visits and/or treatments at baseline	Correlation	U	r=.20<br>**p<.05**
				Regression investigating the influence of level of baseline anxiety on the frequency of healthcare visits at baseline while also accounting for chest pain.	M	β=.16<br>**p=.04**

			Frequency of healthcare visits and/or treatments at follow-up	Correlation	U	r=.17<br>p=.17
Levenson (2008) [43]	232<br>Sickle cell disease (SCD)	General Anxiety Disorder-7<br>→ Anxiety symptoms yes/no (baseline)	% of days having scheduled physician visits for SCD during 6m follow-up	Comparison of the % of days having scheduled visits during follow-up between patients w/ and w/o anxiety symptoms.	U	NS
Lozier (2018) [45]	517<br>Chronic pain	Generalized Anxiety Disorder-7 Scale	Engagement in clinician-directed non-pharmacological treatments<br>→ no/low/moderate/high engagement	Comparison of level of anxiety symptoms between engagement groups of clinician-directed non-pharmacological treatments.	U	High engagement: 9.1<br>Moderate engagement: 6.9<br>Low engagement: 6.3<br>No engagement: 6.7<br>p=.08
Philpot (2018)[5] [63]	772<br>Chronic non-cancer pain	Generalized Anxiety Disorder-7 (GAD-7)<br>→ Anxiety symptoms yes/no (baseline)	Decreases in specialist visits<br>→ yes/no (post-treatment)	Regression investigating the presence of anxiety symptoms (reference: no symptoms) on the likelihood of a decrease in specialist visits (reference: no decrease).	U	negative association<br>**p=.03**
				Regression investigating the influence of presence of anxiety symptoms (reference: no symptoms) on the likelihood of a decrease in specialist visits (reference: no decrease) while also accounting for race (NS), comorbidity index (S), GAD-7 functional status (NS) and opioid prescription dose (S).	M	NS
			Decreases in primary care visits after therapy	Logistic regression to investigate whether presence of anxiety symptoms (reference: no symptoms) is related to a decrease in primary care visits (reference: no decrease).	U	negative association<br>**p=.005**

				Logistic regression to investigate whether presence of anxiety symptoms (reference: no symptoms) is related to a decrease in primary care visits (reference: no decrease) while also accounting for race (NS), gender (NS), marital status (NS), comorbidity index (S), depressive symptoms (PHQ-9) (NS), PHQ-9 functional status (NS) and GAD functional status (NS) (selected based on significance level in univariate analyses).	M	OR: 3.3; 95%CI: 1.2-9.3<br>**p=.02**	
Williams (2018)[5] [86]	95<br>Sickle cell disease pain	Self-designed question: anxiety symptoms<br>→ yes/no	Number of day hospital visits	Comparison of number of day hospital visits between patients w/ and w/o anxiety symptoms while controlling for study site.	M	p=.578	
General anxiety symptoms x emergency HCU							
Musey (2018)[5] [56]	163<br>Chest pain	Hospital Anxiety and Depression Scale – Anxiety subscale<br>→ High vs low anxiety symptoms	Number of ER return visits	To compare the number of ER return visits between patients showing high vs low levels of anxiety symptoms.	U	**p=.001**	<u>Univariate</u><br>0<br>1/4 – 25% positive associations<br><br><u>Multivariate</u><br>?<br><4
Philpot (2018)[5] [63]	772<br>Chronic non-cancer pain	Generalized Anxiety Disorder-7 (GAD-7)<br>→ Anxiety symptoms yes/no (baseline)	Decreases in ER visits<br>→ yes/no (post-treatment)	Regression investigating the influence of presence of anxiety symptoms (reference: no symptoms) on the likelihood of a decrease in ER visits (reference: no decrease).	U	NS	
				Regression investigating the influence of presence of anxiety symptoms (reference: no symptoms) on the likelihood of a decrease in ER visits (reference: no decrease) while also accounting for race (S), marital status (NS), comorbidity index	M	NS	

				(NS), depressive symptoms (PHQ-9) (S), PHQ-9 functional status (NS) and GAD-7 functional status (NS).			
Villani (2010)[5] [78]	465 Migraine	State and Trait Anxiety Inventory – State form	Number of ER visits → Repeaters vs non-repeaters of ER visits	Regression investigating the influence of level of state anxiety symptoms on the likelihood of repeated ER use (reference: no repeated ER use).	U	OR : 1.708 ; 95%CI : .683-4.268 p=.2	
		State and Trait Anxiety Inventory – Trait form	Number of ER visits → Repeaters vs non-repeaters of ER visits	Regression investigating the influence of level of trait anxiety symptoms on the likelihood of repeated ER use (reference: no repeated ER use).	U	OR : .579 ; 95%CI : .213-1.676 p=.3	
Williams (2018)[5] [86]	95 Sickle cell disease pain	Self-designed question: anxiety symptoms → yes/no	Number of ER visits	Comparison of number of ER visits between patients w/ and w/o anxiety symptoms while controlling for study site.	M	p=.856	
General anxiety symptoms x hospitalizations							
Daltroy (1998)[5] [13]	222 Scheduled for knee or hip arthroplasty	State-Trait Anxiety Inventory – State form (baseline/ preoperative)	Length of stay	General linear model investigating the influence of baseline state anxiety on length of stay while also accounting for age (S), reliance in God (S), surgeon (S), date of surgery (S), comorbidities (S), cemented joint (S), greater desire for information (NS), smaller passive range of motion (NS), lack of a discharge plan (NS), greater denial (NS), greater perceived pain control (NS), provision of information (NS) and relaxation training (NS).	M	positive association **p<.054**	<u>Univariate</u><br>?<br><4<br><br><u>Multivariate</u><br>?<br><4
Philpot (2018)[5] [63]	772 Chronic non-cancer pain	Generalized Anxiety Disorder-7 (GAD-7)	Decreases in hospitalizations → yes/no (post-treatment)	Regression investigating the influence of presence of anxiety symptoms (reference: no symptoms) on the likelihood of a	U	NS	

		→ Anxiety symptoms yes/no (baseline)		decrease in hospitalizations (reference: no decrease).			
				Regression investigating the influence of presence of anxiety symptoms (reference: no symptoms) on the likelihood of a decrease in hospitalizations (reference: no decrease) while also accounting for race (NS), marital status (NS), comorbidity index (S), education (NS), current pain (NS), presence of depressive symptoms (PHQ-9) (NS), PHQ-9 functional status (NS), GAD-7 functional status (NS) and prescribed opioid dose (NS).	M	NS	
Williams (2018)[5] [86]	95<br>Sickle cell disease pain	Self-designed question: anxiety symptoms<br>→ yes/no	Number of hospital admissions	Comparison of number of hospital admissions between patients w/ and w/o anxiety symptoms while controlling for study site.	M	p=.926	
General anxiety symptoms x CAM use							
Harding (2019) [28]	127<br>Chronic pain	PROMIS Emotional Distress – Anxiety subscale	Number of different types of self-management strategies used	Correlation	U	r=.19<br>**p<.05**	<u>Univariate</u><br>?<br><4<br><br><u>Multivariate</u><br>?<br><4
				Regression investigating the influence of anxiety symptoms on the number of different types of self-management strategies used while also accounting for age (NS), gender (NS), pain intensity (NS), pain interference (NS), depressive symptoms (NS), PTSD (NS) and sleep (NS).	M	β=-.01; 95%CI: -.09-.06<br>p=.719	

Lozier<br>(2018) [45]	517<br>Chronic pain	Generalized Anxiety Disorder-7 Scale	Engagement in self-directed non-pharmacological treatments<br>→ no/low/moderate/high engagement	Comparison of level of anxiety symptoms score between engagement groups of self-directed non-pharmacological treatments.	U	High engagement: 7.6<br>Moderate engagement: 6.7<br>Low engagement: 6.5<br>No engagement: 6.7<br>p=.65	
General anxiety symptoms x HCU in general							
Harding<br>(2019) [28]	127<br>Chronic pain	PROMIS Emotional distress – Anxiety subscale	Number of different types of provider management used for pain	Correlation	U	r=.17<br>p>.05	<u>Univariate</u><br>?<br><4<br><br><u>Multivariate</u><br>?<br><4
				Regression investigating the influence of anxiety symptoms on the number of different types of provider management used while also accounting for age (NS), gender (NS), pain intensity (NS), pain interference (NS), depressive symptoms (NS), PTSD (NS) and sleep (NS).	M	β=.02; 95%CI: -.03-.08<br>p=.356	
Woodhouse<br>(2016) [88]	219<br>Neck/low back pain	Hospital Anxiety and Depression Scale – Anxiety subscale<br>→ Anxiety symptoms yes/no<br>(baseline)	Future conventional care use (physicians, PT, chiropractors, psychologists, prescribed medications and use of both alternative en conventional care)<br>→ yes/no<br>(assessed at several follow-up moments)	Regression investigating whether baseline presence of anxiety symptoms (reference: no symptoms) is predicting use of conventional care (reference: no conventional care) while controlling for age, sex, time of follow-up, marital status, work-related factors and socioeconomic status.	M	RD: 11<br>**95%CI: 2-20**	
Symptom-related anxiety symptoms x pain medication use							
Carroll<br>(2018)[5] [7]	73<br>Sickle cell disease	Pain Anxiety Symptom Scale (PASS-20)<br>(baseline)	Within-visit acute opioid dose	Linear mixed model investigating the influence of PASS-20 score on within-visit opioid dose while also accounting for demographics (age (NS) and sex(NS)), disease-related variables (genotype (NS), hemoglobin (NS), acute chest (NS), avascular necrosis (NS), prior hydroxyurea (NS), chronic transfusion (NS), total daily	M	β=-.27<br>NS	<u>Univariate</u><br>?<br><4<br><br><u>Multivariate</u><br>?<br><4

				opioids (S) and utilization (S)), socioeconomic status (S) and psychiatric variables (family history (S), psychiatric treatment (NS) and substance use family (NS)).			
Elander (2014) [19]	112 General population w/ pain	Pain Anxiety Symptoms Scale	Frequency of prescription pain medication use	Correlation	U	r=.41 **p<.001**	
			Frequency of OTC pain medication use	Correlation	U	r=-.13 NS	
Symptom-related anxiety symptoms x consultations							
Biggs (2003) [3]	151 Abdominal or chest pain	Health Anxiety Questionnaire (HAQ)	Number of consultations w/ healthcare providers	Regression investigating the influence of HAQ score on the number of consultations while also accounting for education, access to confidant, pain score, recent social stress, exposure to death of a father or mother during childhood, reported childhood adversity (antipathy from father or mother, neglect, physical abuse or psychological abuse), depressive symptoms, general anxiety symptoms, negative illness perceptions (consequences and timeline), perceived symptom control, SF-36 scores (role limitations physical and mental, social function, energy and vitality and pain) (all above NS – omitted from final model), sex (S), SF-36 scores (physical function, health perception and mental health) (S), marital status (S), diagnosis (S), death of a sibling (S) and reported sexual abuse (S).	M	NS (omitted from final model)	<u>Univariate</u><br>++<br>10/11 – 91%<br><br><u>Multivariate</u><br>?<br>8/16 – 50% positive associations
			Number of GP consultations	Regression investigating the influence of HAQ score on the number of GP consultations while also accounting for	M	NS (omitted from final model)	

			education, access to confidant, pain score, recent social stress, exposure to death of a father or mother during childhood, reported childhood adversity (sexual abuse, antipathy from mother, neglect, physical abuse or psychological abuse), depressive symptoms, general anxiety symptoms, negative consequences beliefs, perceived symptom control, SF-36 scores (role limitations physical and mental, social function, energy and vitality, physical function, health perception and mental health) (all above NS – omitted from final model), sex (S), SF-36 pain score (S), marital status (S), illness perception timeline score (S), diagnosis (S), death of a sibling (S) and reported antipathy from father (S).			
		Number of consultations w/ other providers than GP	Regression investigating the influence of HAQ score on the number of consultations w/ other providers than GP while also accounting for marital status, diagnosis, education, access to confidant, recent social stress, exposure to death of a sibling, father or mother during childhood, reported childhood adversity (sexual abuse, antipathy from father or mother, neglect, physical abuse or psychological abuse), depressive symptoms, general anxiety symptoms, negative illness perceptions (consequences and timeline), perceived symptom	M	NS (omitted from final model)	

				control, SF-36 scores (pain score, role limitations physical and mental, social function, energy and vitality, physical function, health perception and mental health) (all above NS – omitted from final model), sex (S), SF-36 (mental health, health perception and physical function) (S) and pain score (S).		
Carroll (2018)[5] [7]	73<br>Sickle cell disease	Pain Anxiety Symptom Scale (baseline)	Use of sickle cell infusion center in the following year → no use vs typical use vs high use	Comparison of baseline level of pain anxiety between utilization groups.	U	No use: 41.56 ± 20.13<br>Typical use: 48.30 ± 22.90<br>High use: 49.78 ± 16.39<br>p=.318
				Regression investigating the influence of baseline level of pain anxiety on the level of utilization while also accounting for demographics (age (NS) and sex (NS)), disease-related variables (genotype (NS), hemoglobin (NS), acute chest (S), avascular necrosis (NS), prior hydroxyurea (S), chronic transfusion (S), total daily opioids (S), socioeconomic status (S) and psychiatric variables (family history (NS), psychiatric treatment (S) and substance use family (NS)).	M	β=.02<br>**p<.05**
Görge (2017) [24]	688<br>Chronic low back pain	Pain Coping Questionnaire – Anxiety subscale (FESV-AX) (baseline or change between baseline and immediately post-rehabilitation)	Number of GP visits 6m post-rehabilitation	Regression investigating the influence of baseline level of pain anxiety on the number of GP visits post-rehabilitation while also accounting for baseline GP visits (S), hours of work (S), days on sick leave (S), state of health (S), SF-12 physical component score (NS), chronicity (NS) and anger symptoms (S).	M	β=-.091<br>NS
				Regression investigating the influence of change in level of	M	β=.085<br>**p<.05**

				pain anxiety on the number of GP visits post-rehabilitation while also accounting for baseline GP visits (S), hours of work (S), days on sick leave (S), state of health (S), SF-12 physical component score (NS), chronicity (NS) and anger symptoms (S) and change in sick leave (S) and coping (experience of competencies) (NS).		
			Number of specialist visits 6m post-rehabilitation	Regression investigating the influence of change in level of pain anxiety on the number of specialist visits post-rehabilitation while also accounting for baseline specialist visits (S), days on sick leave (S), state of health (S) and change in sick leave (S), helplessness and depression (S) and pain function and disability (NS).	M	β=.118<br>**p<.05**
Hadlandsmyth (2013) [26]	Baseline: 196<br>Follow-up: 70<br>Non-cardiac chest pain	Albany Panic and Phobia Questionnaire – Interoceptive fear subscale (baseline)	Frequency of healthcare visits and/or treatments at baseline	Correlation	U	r=.24<br>**p<.05**
				Regression investigating the influence of baseline interoceptive fear on the frequency of healthcare visits at baseline while also accounting for chest pain.	M	β=.20<br>**p=.01**
			Frequency of healthcare visits and/or treatments at follow-up	Regression investigating the influence of baseline interoceptive fear on the frequency of healthcare visits.	U	β=.25<br>**p<.05**
Howell (1999) [30]	614<br>Dyspepsia	Self-designed questionnaire<br>→ none; a little; moderate; considerable; extreme pain-related anxiety	Frequent GP visits (≥6) for dyspepsia symptoms<br>→ yes/no	Chi²	U	<u>% having frequent visits</u><br>None: 12.6%<br>A little: 21.6%<br>Moderate: 24.6%<br>Considerable: 37.1%<br>Extreme: 52.8%<br>**p=.001**

				Regression investigating the influence of level of pain-related anxiety symptoms (reference: none) on the likelihood of having frequent (≥6) GP visits (reference: <6) while also accounting for neuroticism, marital status, ethnicity, smoking status, NSAID use, age, pain duration, pain severity, fear of serious illness, fear that pain might be cancer (all above: NS – omitted from final model), gender (S), alcohol consumption (S) and pain frequency (S).	M	<u>ORs (95%CI); p</u><br>A little: 1.74 (.84-3.59); .14<br>Moderate: 2.05 (1.00-4.19); .05<br>Considerable: 3.65 (1.76-7.55); **.005**<br>Extreme: 6.08 (2.43-15.18); **.0001**	
		Fear of serious illness<br>→ yes/no	Frequent GP visits for dyspepsia symptoms<br>→ yes/no	Chi^2	U	<u>% having frequent visits</u><br>Fear: 30.7%<br>No fear: 17.8%<br>**p=.001**	
				Regression investigating the influence of having fear of serious illness (reference: no such fear) on the likelihood of having frequent (≥6) GP visits (reference: <6) while also accounting for neuroticism, marital status, ethnicity, smoking status, NSAID use, age, pain duration, pain severity, fear that pain might be cancer (all above: NS – omitted from final model), gender (S), alcohol consumption (S), pain-related anxiety symptoms (S) and pain frequency (S).	M	NS (omitted from final model)	
		Fear that pain might be cancer<br>→ yes/no	Frequent GP visits for dyspepsia symptoms<br>→ yes/no	Chi^2	U	<u>% having frequent visits</u><br>Fear: 33.5%<br>No fear: 21.5%<br>**p=.001**	
				Regression investigating the influence of having fear that pain	M	NS (omitted from final model)	

				might be cancer (reference: no such fear) on the likelihood of having frequent (≥6) GP visits (reference: <6) while also accounting for neuroticism, marital status, ethnicity, smoking status, NSAID use, age, pain duration, pain severity, fear of serious illness (all above: NS – omitted from final model), gender (S), alcohol consumption (S), pain-related anxiety symptoms (S) and pain frequency (S).		
Mourad (2018) [55]	552<br>Non-cardiac chest pain	Body Sensations Questionnaire	Frequency of seeking care for pain	Structural equation model investigating the influence of level of fear of body sensations on the frequency of healthcare visits while also accounting for somatization (NS), depressive symptoms (NS) and cardiac anxiety (S).	M	NS
		Cardiac Anxiety Questionnaire	Frequency of seeking care for pain	Structural equation model investigating the influence of level of cardiac anxiety on the frequency of healthcare visits while also accounting for somatization (NS), depressive symptoms (NS) and fear of body sensations (NS).	M	β=0.61<br>**p<.01**
Mourad (2016) [54]	552<br>Non-cardiac chest pain	Body Sensations Questionnaire	Frequency of pain-related visits<br>→ low: <2; high: 2-3; very high: >3 visits	Kruskal Wallis comparing level of fear of body sensations between the frequency of visits groups.	U	Very high: 37.9 ± 13.1<br>High: 32.7 ± 12.0<br>Low: 29.3 ± 11.3<br>**p<.001**
			Frequency of pain-related visits<br>→ low: ≤ 1 visit; high: ≥ 2 visits	Regression investigating the influence of level of fear of body sensations on the frequency of visits (reference: low frequency) while also accounting for age, sex, multi-morbidity, cardiac	M	OR: .99; 95%CI: .97-1.01<br>p=.172

				anxiety (S) and depressive symptoms (NS).		
		Cardiac Anxiety Questionnaire - Total score	Frequency of pain-related visits → low: <2; high: 2-3; very high: >3 visits	Comparison of cardiac anxiety levels between the frequency of visits groups.	U	Very high: 36.1 ± 12.5<br>High: 29.2 ± 11.8<br>Low: 20.0 ± 11.1<br>**p<.001**
			Frequency of pain-related visits → low: ≤ 1 visit; high: ≥ 2 visits	Regression investigating the influence of level of cardiac anxiety on the frequency of visits (reference: low frequency) while also accounting for age, sex, multi-morbidity, fear of body sensations (NS) and depressive symptoms (NS).	M	OR: 1.08; 95%CI: 1.06-1.10<br>**p<.001**
		Cardiac Anxiety Questionnaire (CAQ) – Fear subscale	Frequency of pain-related visits → low: <2; high: 2-3; very high: >3 visits	Comparison of CAQ fear score between the frequency of visits groups.	U	Very high: 2.3 ± .7<br>High: 1.9 ± .7<br>Low: 1.4 ± .8<br>**p<.001**
Tremblay (2018) [72]	428<br>Non-cardiac chest pain	Cardiac Anxiety Questionnaire	Number of healthcare visits (primary care, specialists and ER)	Regression to investigate the influence of level of cardiac anxiety on the number of healthcare visits.	U	IRR: 1.03; 95%CI: 1.01-1.04<br>**p<.001**
				Regression to investigate the influence of level of cardiac anxiety on the number of healthcare visits while adjusting for depressive symptoms (NS), presence of panic disorder (NS), pain frequency (S), pain intensity (NS), pain interference (S), presence of medical condition (S) and gastrointestinal symptoms (NS).	M	IRR: 1.01; 95%CI: 1.00-1.02<br>**p=.02**
Zondervan (2001) [90]	475<br>Chronic pelvic pain	Pain anxiety → Self-designed question pain anxiety yes/no	Recent consulters vs past consulters vs non-consulters	Comparing the proportion of patients reporting anxiety symptoms (reference: no anxiety symptoms) between the 3 consulter groups.	U	Recent consulters: 41%<br>Past consulters: 32%<br>Non-consulters: 22%<br>**p=.001**
Catastrophizing x pain medication use						

Elander (2003) [18]	68<br>Haemophilia	Coping Strategies Questionnaire	Frequency of OTC pain medication use	Correlation	U	r=.14<br>NS	<u>Univariate</u><br>?<br>3/6 – 50% positive associations<br><br><u>Multivariate</u><br>?<br><4
			Frequency of prescription pain medication use	Correlation	U	r=.21<br>NS	
Elander (2014) [19]	112<br>Pain	Pain Catastrophizing Scale	Frequency of prescription pain medication use	Correlation	U	r=.44<br>**p<.001**	
			Frequency of OTC pain medication use	Correlation	U	r=-.17<br>NS	
Durá-Ferrandis (2017) [17]	72<br>TMD participating in CBT intervention study	Pain Catastrophizing Score (PCS) (change pre-post-treatment)	Frequency of self-medication (change pre-post-treatment)	SEM investigating whether change in PCS score was a potential mediator of the treatment effect on frequency of self-medication next to psychological distress (NS), pain intensity (NS), perceived control (NS) and coping strategies (distraction (S) and mental self-control (NS)).	M	SEM loading: .09<br>NS	
Wideman (2011) [83]	202<br>Musculoskeletal neck/back injury undergoing a 7w PT intervention	Pain Catastrophizing Scale (assessed after PT intervention)	Use of OTC NSAID's, opioids, prescription anti-inflammatory drugs or psychotropic drugs → yes/no for each, summed into 0-4 score for use of different pain medications (assessed 1y after baseline)	Correlation	U	r=.375<br>**p<.01**	
				Regression investigating the influence of level of pain catastrophizing on the amount of different pain medications used while controlling for sex (S), pain duration (NS), pre-treatment opioid use (S) and post-treatment pain intensity (S), depressive symptoms (NS), kinesiophobia (NS) and pain self-efficacy (S).	M	β=.091<br>NS	
Wong (2019)[5] [87]	125<br>Undergoing laparoscopic hysterectomy	Pain Catastrophizing Scale	Amount of opioid use	Correlation	U	**p<.001**	
Catastrophizing x consultations							
Ciechanowski (2003) [8]	111<br>Chronic pain	Coping Strategy Questionnaire – Catastrophizing subscale	Frequency of pain-related visits post-treatment → ≥weekly vs <weekly	Logistic regression investigating the influence of baseline catastrophizing score on the likelihood of having ≥weekly	M	β=-.45; SE=.49 (reference: <weekly)<br>NS	<u>Univariate</u><br>?<br>4/9 – 44%

		(baseline)		visits (reference: <weekly) while also accounting for age (NS), gender (NS), baseline pain-related HCU (NS), attachment style (secure (NS), preoccupied (S), fearful (NS) and dismissing (NS)) and depressive symptoms (NS).			positive associations<br><br><u>Multivariate</u><br>00<br>0/7 – 0%
			Frequency of pain-related visits post-treatment → ≥monthly vs ≥weekly	Regression investigating the influence of baseline catastrophizing score on the likelihood of having ≥monthly visits (reference: ≥weekly) while also accounting for age (NS), gender (NS), baseline pain-related HCU (S), attachment style (secure, preoccupied, fearful, dismissing) (NS) and depressive symptoms (NS).	M	β=.40; SE=.32 (reference: ≥weekly)<br>NS	
Demmelmaier (2010) [15]	42<br>First-episode back pain	Coping Strategy Questionnaire - Catastrophizing Subscale (baseline)	Number of consultations w/ healthcare providers at follow-up	Correlation	U	NS	
	271<br>Chronic back pain	Coping Strategy Questionnaire - Catastrophizing Subscale (baseline)	Number of consultations w/ healthcare providers at follow-up	Correlation	U	NS	
Elander (2003) [18]	68<br>Haemophilia	Coping Strategies Questionnaire – Catastrophizing subscale	Number of healthcare visits	Correlation	U	r=-.05<br>NS	
Jensen (1994) [32]	94<br>Chronic pain participating in multidisciplinary program	Coping Strategies Questionnaire – Catastrophizing subscale (change score pre-post-treatment)	Number of pain-related physician visits (change score pre-post-treatment)	Correlation	U	r=-.26<br>**p<.01**	

Kapoor (2012)[5] [36]	64 Chronic non-cancer pain	Pain Catastrophizing Scale (baseline)	Number of healthcare visits (pre-treatment)	Correlation	U	Positive correlation **significant**
				Regression investigating the influence of baseline pain catastrophizing on the number of pre-treatment healthcare visits while also accounting for depressive symptoms (S).	M	β=.004 NS
		Pain Catastrophizing Scale (post-treatment)	Number of healthcare visits (post-treatment)	Correlation	U	Positive association **significant**
				Regression investigating the influence of post-treatment pain catastrophizing on the number of post-treatment healthcare visits while also accounting for perceived disability (NS).	M	β=.099 NS
Kapoor (2014) [37]	64 Chronic pain	Pain Catastrophizing Scale	Number of healthcare visits	Correlation	U	r=-.260 **p<.05**
				Regression investigating the influence of catastrophizing on the number of healthcare visits while also accounting for comorbidities (NS), pain intensity (NS) and depressive symptoms (S).	M	IRR=.22; 95%CI: .022-.220 p=.639
Newman (2018)[5] [59]	290 Chronic pain	Pain Catastrophizing Scale	Number of pain-related consultations	Correlation	U	r=.23 **p<.01**
				Regression investigating the influence of level of pain catastrophizing on the number of pain-related consultations while also accounting for demographics (age (NS), sex (NS) and race (S)), socioeconomic variables (poverty status (NS), education (NS) and literacy (NS)) and pain-related variables (physical function (NS), pain severity (NS), pain interference (NS), number of pain sites (NS)	M	β=.06 p=.49

				and types (NS), opioid use (S) and depressive symptoms (S)).			
Wideman (2011) [83]	202 Musculoskeletal neck/back injury undergoing a 7w PT intervention	Pain Catastrophizing Scale (assessed after PT intervention)	Use of PT, psychology, massage therapy and other medical services → yes/no for each, summed into 0-4 score for use of different healthcare services (assessed 1y after baseline)	Correlation	U	r=.289 **p<.01**	
				Regression investigating the influence of level of pain catastrophizing on the amount of different healthcare services used while controlling for pre-treatment opioid use (S) and post-treatment pain intensity (S), depressive symptoms (NS), kinesiophobia (NS) and pain self-efficacy (NS).	M	β=.072 NS	
Catastrophizing x emergency HCU							
Citero (2007) [9]	220 Sickle cell disease	Coping Strategies Questionnaire (CSQ) – Catastrophizing subscale (baseline)	Number of unscheduled doctor visits on crisis days	Regression investigating the influence of baseline catastrophizing on the number of unscheduled doctor visits on crisis days.	U	NS	<u>Univariate</u> 00 0/4 – 0% <u>Multivariate</u> 00 0/4 – 0%
				Regression investigating the influence of baseline catastrophizing on the number of unscheduled doctor visits on crisis days while controlling for depression.	M	NS	
			Number of ER visits on crisis days	Regression investigating the influence of baseline catastrophizing on the number of ER visits on crisis days.	U	NS	
				Regression investigating the influence of baseline catastrophizing on the number of ER visits on crisis days while controlling for depression.	M	NS	
			Number of unscheduled doctor visits on non-crisis days	Regression investigating the influence of baseline catastrophizing on the number of unscheduled doctor visits on non-crisis days.	U	NS	

				Regression investigating the influence of baseline catastrophizing on the number of unscheduled doctor visits on non-crisis days while controlling for depression.	M	NS	
			Number of ER visits on non-crisis days	Regression investigating the influence of baseline catastrophizing on the number of ER visits on non-crisis days.	U	NS	
				Regression investigating the influence of baseline catastrophizing on the number of ER visits on non-crisis days while controlling for depression.	M	NS	
Catastrophizing x hospitalizations							
Citero (2007) [9]	220 Sickle cell disease	Coping Strategies Questionnaire – Catastrophizing subscale (baseline)	Number of hospitalizations on crisis days	Regression investigating the influence of baseline catastrophizing on the number of hospitalizations on crisis days.	U	NS	Univariate ? <4
				Regression investigating the influence of baseline catastrophizing on the number of hospitalizations on crisis days w/ CSQ score while controlling for depression.	M	NS	Multivariate ? <4
			Hospitalizations on non-crisis days	Regression investigating the influence of baseline catastrophizing on the number of ER visits.	U	NS	
				Regression investigating the influence of baseline catastrophizing on the number of ER visits while controlling for depression.	M	NS	
Catastrophizing x HCU in general							
Citero (2007) [9]	220 Sickle cell disease	Coping Strategies Questionnaire – Catastrophizing subscale	Amount of HCU (unscheduled doctor visits + ER visits +	Regression investigating the influence of baseline catastrophizing score on the amount of HCU on crisis days.	U	β=-.03 p=.75	Univariate ? <4

		(baseline)	hospitalizations) on crisis days	Regression investigating the influence of baseline catastrophizing score on the amount of HCU on crisis days while controlling for depression.	M	β=.01<br>p=.96	Multivariate<br>?<br><4
			Amount of HCU (unscheduled doctor visits + ER visits + hospitalizations) on non-crisis days	Regression investigating the influence of baseline catastrophizing on the amount of HCU on non-crisis days.	U	β=.02<br>p=.74	
				Regression investigating the influence of baseline catastrophizing on the amount of HCU on non-crisis days while controlling for depression.	M	β=.03<br>p=.73	
Depressive symptoms x pain medication use							
Elander (2014) [19]	112<br>General population w/ pain	Depression, Anxiety and Stress Scale – Depression subscale	Frequency of prescription pain medication use	Correlation	U	r=.23<br>**p<.05**	Univariate<br>++<br>6/7 – 86%<br><br>Multivariate<br>00<br>2/7 – 29% positive associations
			Frequency of OTC pain medication use	Correlation	U	r=-.09<br>NS	
Engel (1996)[5] [20]	1,059<br>Spinal pain	Symptom Checklist-90 – Depression subscale (baseline)	Having ≥8 pain medicine fills vs <8<br>(11m follow-up)	Regression investigating the influence of level of baseline depressive symptoms on the likelihood of having ≥8 pain medicine fills (reference: <8) at follow-up.	U	positive association<br>**p<.001**	
				Regression investigating the influence of level of baseline depressive symptoms on the likelihood of having ≥8 pain medicine fills (reference: <8) at follow-up while also accounting for age, gender, education, chronic pain grade (S), days in pain (S), disability pay (S) and diagnosis (NS).	M	positive association<br>**p<.001**	
Gil (2004) [23]	41<br>Sickle cell disease	Daily Mood Scale - Negative mood subscale (baseline)	Using prescription pain medication on the same day<br>→ yes/no	Regression investigating the influence of negative mood on the likelihood of using prescription pain medication on	M	β=.09<br>**p<.001**	

				the same day (reference: no use) while controlling for level of pain.		
			Using prescription pain medication on the next day → yes/no	Regression investigating the influence of negative mood on the likelihood of using prescription pain medication on the next day (reference: no use) while controlling for level of pain.	M	NS
			Using prescription pain medication 2d later → yes/no	Regression investigating the influence of negative mood on the likelihood of using prescription pain medication 2d later (reference: no use) while controlling for level of pain.	M	NS
Kratz (2018) [39]	120 Spinal cord injury w/ chronic pain	Patient Health Questionnaire-9	Number of pain medications used	Regression investigating the influence of level of depressive symptoms on the number of pain medications used while also accounting for pain intensity (NS), number of painful body areas (S) and pain acceptance (S).	M	β=-.02<br>p=.34
Levenson (2008) [43]	232 Sickle cell disease (SCD)	Patient Health Questionnaire-9 → Depressive symptoms yes/no (baseline)	% days using opioids for SCD in 6m period from baseline	Comparison of opioid use between patients w/ and w/o depressive symptoms.	U	w/ symptoms: 58.3 ± 39.0<br>w/o symptoms: 40.5 ± 40.2<br>**p=.003**
				Comparison of opioid use between patients w/ and w/o depressive symptoms while controlling for age and income.	M	p=.21
Nielsen (2015) [60]	1,220 Chronic non-cancer pain	Patient Health Questionnaire-9 → depressive symptoms yes/no	4 categories of BZD use: no use; past use; current less than daily use; current daily use	Regression comparing the likelihood of showing depressive symptoms (reference: no symptoms) between patients from the different BZD use groups (reference: no use).	U	Past: OR: 1.79; 95%CI: 1.24-2.57<br><Daily: OR: 2.25; 95%CI: 1.47-3.43<br>Daily: OR: 3.86; 95%CI: 2.61-5.71<br>**p<.01**
Wideman (2011) [83]	202 Musculoskeletal neck/back injury undergoing a 7w PT intervention	Beck Depression Inventory (assessed after PT intervention)	Use of OTC NSAID's, opioids, prescription anti-inflammatory drugs or psychotropic drugs	Correlation	U	r=.396<br>**p<.01**
				Regression investigating the influence of level of depressive symptoms on the amount of	M	β=.043<br>NS

			→ yes/no for each, summed into 0-4 score for use of different pain medications (assessed 1y after baseline)	different pain medications used while controlling for sex (S), pain duration (NS), pre-treatment opioid use (S) and post-treatment pain intensity (S), pain catastrophizing (NS), kinesiophobia (NS) and pain self-efficacy (S).			
Wong (2019)[5] [87]	125 Scheduled for hysterectomy	Patient Health Questionnaire-9 (preoperative)	Amount of postoperative opioid use	Correlation	U	positive association **p<.001**	
Depressive symptoms x consultations							
Alschuler (2012) [1]	161 Multiple sclerosis w/ pain	Patient Health Questionnaire-9 (PHQ-9)	Total number of visits w/ healthcare providers	Regression investigating the influence of PHQ-9 score on the number of visits.	U	β=.07 p=.472	<u>Univariate</u> ? 13/25 – 52% positive associations <u>Multivariate</u> ? 13/32 – 41% positive associations
				Regression investigating the influence of PHQ-9 score on the number of visits while controlling for pain intensity.	M	β=.005 p=.63	
		Patient Health Questionnaire-9 → Depressive symptoms yes/no	Number of visits w/ primary care providers	Comparison of the number of primary care visits between patients w/ and w/o depressive symptoms.	U	w/: 1.85 ± 1.91 w/o: 1.48 ± 2.99 NS	
			Number of visits w/ MS specialist	Comparison of the number of MS specialist visits between patients w/ and w/o depressive symptoms.	U	w/: .54 ± .97 w/o: .63 ± .81 NS	
			Number of visits w/ other MDs than primary care providers and MS specialists	Comparison of the number of visits w/ other MDs between patients w/ and w/o depressive symptoms.	U	w/: 1.62 ± 2.22 w/o: .38 ± .90 NS	
			Number of visits w/ PT/OT	Comparison of the number of PT/OT visits between patients w/ and w/o depressive symptoms.	U	w/: 8.69 ± 20.29 w/o: 2.21 ± 7.39 NS	
			Number of visits w/ other healthcare providers than MDs, PT/OT, chiropractors and ER	Comparison of the number of visits w/ other providers between patients w/ and w/o depressive symptoms.	U	w/: .38 ± .96 w/o: 1.38 ± 6.67 NS	

			Number of visits w/ healthcare providers w/o PT/OT	Comparison of the number of visits w/ providers except PT/OT between patients w/ and w/o depressive symptoms.	U	w/: 8.32 ± 5.44<br>w/o: 5.24 ± 8.88<br>NS
Biggs (2003) [3]	151<br>Abdominal or chest pain	Hospital Anxiety and Depression Scale – Depression subscale (HADS-D)	Number of consultations w/ healthcare providers	Regression investigating the influence of HADS-D score on the number of consultations while also accounting for education, access to confidant, pain score, recent social stress, exposure to death of a father or mother during childhood, reported childhood adversity (antipathy from father or mother, neglect, physical abuse or psychological abuse), general and symptom-related anxiety symptoms, negative illness perceptions (consequences and timeline), perceived symptom control, SF-36 scores (role limitations physical and mental, social function, energy and vitality and pain) (all above NS – omitted from final model), sex (S), SF-36 scores (physical function, health perception and mental health) (S), marital status (S), diagnosis (S), death of a sibling (S) and reported sexual abuse (S).	M	NS (omitted from final model)
			Number of GP consultations	Regression investigating the influence of HADS-D score on the number of GP consultations while also accounting for education, access to confidant, pain score, recent social stress, exposure to death of a father or mother during childhood, reported childhood adversity (sexual abuse, antipathy from mother, neglect, physical abuse	M	NS (omitted from final model)

			or psychological abuse), general and symptom-related anxiety symptoms, negative consequences beliefs, perceived symptom control, SF-36 scores (role limitations physical and mental, social function, energy and vitality, physical function, health perception and mental health) (all above NS – omitted from final model), sex (S), SF-36 pain score (S), marital status (S), illness perception timeline score (S), diagnosis (S), death of a sibling (S) and reported antipathy from father (S).			
		Number of consultations w/ other providers than GP	Regression investigating the influence of HADS-D score on the number of consultations w/ other providers than GP while also accounting for marital status, diagnosis, education, access to confidant, recent social stress, exposure to death of a sibling, father or mother during childhood, reported childhood adversity (sexual abuse, antipathy from father or mother, neglect, physical abuse or psychological abuse), general and symptom-related anxiety symptoms, negative illness perceptions (consequences and timeline), perceived symptom control, SF-36 scores (pain score, role limitations physical and mental, social function, energy and vitality, physical function, health perception and mental health) (all above NS – omitted from final model), sex (S), SF-36	M	NS (omitted from final model)	

				(mental health, health perception and physical function) (S) and pain score (S).		
Carroll (2016)[5] [6]	83<br>Sickle cell disease	Center for Epidemiologic Studies Depression Scale	Days w/ calls to providers	Regression predicting days w/ calls to providers w/ being on chronic opioid therapy as the independent variable and level of depressive symptoms as a covariate.	M	β=.31<br>**p<.05**
			Days w/ medical visits	Regression predicting days w/ medical visits w/ being on chronic opioid therapy as the independent variable and level of depressive symptoms as a covariate.	M	β=.29<br>**p<.05**
Ciechanowski (2003) [8]	111<br>Chronic pain	Center for Epidemiological Studies Depression Scale (baseline)	Frequency of pain-related visits post-treatment → ≥weekly vs <weekly	Regression investigating the influence of baseline level of depressive symptoms on the likelihood of having ≥weekly visits (reference: <weekly) while also accounting for age (NS), gender (NS), baseline pain-related HCU (NS), attachment style (secure (NS), preoccupied (S), fearful (NS) and dismissing (NS)) and catastrophizing (NS).	M	β=.02; SE=.05 (reference: <weekly)<br>NS
			Frequency of pain-related visits post-treatment → ≥monthly vs ≥weekly	Regression investigating the influence of baseline level of depressive symptoms on the likelihood of having ≥monthly visits (reference: ≥weekly) while also accounting for age (NS), gender (NS), baseline pain-related HCU (S), attachment style (secure, preoccupied, fearful, dismissing) (NS) and catastrophizing (NS).	M	β=-.02; SE=.04 (reference: ≥weekly)<br>NS
Demmelmaier (2010) [15]	42<br>First-episode back pain	Hospital Anxiety and Depression Scale – Depression	Number of consultations w/ healthcare providers at follow-up	Regression investigating the influence of baseline depressive symptoms on the number of consultations at follow-up.	U	r^2=.093; 95%CI: .00-.08<br>**p=.05**

		subscale (baseline)				
	271<br>Chronic back pain	Hospital Anxiety and Depression Scale – Depression subscale (baseline)	Number of consultations w/ healthcare providers at follow-up	Correlation	U	NS
Engel (1996)[5] [20]	1,059<br>Spinal pain	Symptom Checklist-90 – Depression subscale (baseline)	≥2 primary care back pain visits vs <2 at 11m follow-up	Regression investigating the influence of level of baseline depressive symptoms on the likelihood having ≥2 primary care visits (reference: <2).	U	**p<.01**
				Regression investigating the influence of level of baseline depressive symptoms on the likelihood having ≥2 primary care visits (reference: <2) while also accounting for age, gender, education chronic pain grade (S), days in pain (S), disability pay (S) and diagnosis (NS).	M	NS
			≥2 back pain radiologic procedures vs <2 at 11m follow-up	Regression investigating the influence of level of baseline depressive symptoms on the likelihood having ≥2 radiologic procedures (reference: <2).	U	**p<.05**
				Regression investigating the influence of level of baseline depressive symptoms on the likelihood having ≥2 radiologic procedures (reference: <2) while also accounting for age, gender, education chronic pain grade (S), days in pain (S), disability pay (NS) and diagnosis (S).	M	NS
Gil (2004) [23]	41<br>Sickle cell disease	Daily Mood Scale – Negative mood subscale (Baseline)	Doctor call on the same day<br>→ yes/no	Regression investigating the influence of negative mood on the likelihood of having a doctor call on the same day (reference:	M	β=.04<br>**p<.05**

				no call) while controlling for level of pain.		
			Doctor call on the next day → yes/no	Regression investigating the influence of negative mood on the likelihood of having a doctor call on the next day (reference: no call) while controlling for level of pain.	M	NS
			Doctor call 2d later → yes/no	Regression investigating the influence of negative mood on the likelihood of having a doctor call 2d later (reference: no call) while controlling for level of pain.	M	NS
Görge (2017) [24]	688 Chronic low back pain	Pain Coping Questionnaire – Helplessness and depression subscale (FESV-D) (baseline or change pre vs immediately post-treatment)	Number of PT visits 6m post-rehabilitation	Regression investigating the influence of baseline FESV-D score on the number of PT visits post-rehabilitation while also accounting for baseline PT visits (S) gender (S), inability to work (S), employment (S), hours of work (NS), days on sick leave (S), coping (experience of competencies) (NS), fatalistic external locus of control (NS) and activity beliefs (NS).	M	β=.167 **p<.01**
			Number of psychotherapy 6m post-rehabilitation	Regression investigating the influence of baseline FESV-D score on the number of psychotherapy visits post-rehabilitation while also accounting for baseline psychotherapy visits (S), employment (NS), hours of work (NS), days on sick leave (S) and disability (NS).	M	β=.208 **p<.01**

			Number of specialist visits 6m post-rehabilitation	Regression investigating the influence of change in FESV-D score on the number of specialist visits post-rehabilitation while also accounting for baseline specialist visits (S), days on sick leave (S) and state of health (S) and change in sick leave (S), anxiety symptoms (S) and pain function and disability (NS).	M	β=-.104<br>Categorized as a positive relationship between presence of depressive symptoms and consultations as larger improvements (decreases) in depressive symptoms are related to lower healthcare visits.<br>**p<.05**
Kapoor (2014) [37]	64<br>Chronic pain	Center for Epidemiologic Studies Depression Scale	Number of healthcare visits	Correlation	U	r=-.399<br>**p<.001**
				Regression investigating the influence of depressive symptoms on the number of healthcare visits while also accounting for comorbidities (NS), pain intensity (NS) and pain catastrophizing (NS).	M	IRR=5.66; 95%CI: .004-.038<br>**p=.017**
Kapoor (2012)[5] [36]	64<br>Chronic pain	Center for Epidemiological Studies Depression Scale (baseline)	Number of healthcare visits pre-treatment	Correlation	U	Positive correlation<br>**significant**
				Regression investigating the influence of depressive symptoms on the number of visits pre-treatment while also accounting for catastrophizing (NS).	M	β=.362<br>**p=.014**
		Center for Epidemiological Studies Depression Scale (post-treatment)	Number of healthcare visits post-treatment	Correlation	U	NS
Levenson (2008) [43]	232<br>Sickle cell disease (SCD)	Patient Health Questionnaire-9 → Depressive symptoms yes/no (baseline)	% of days having scheduled physician visits for SCD during 6m follow-up	Comparison of the % of days having scheduled visits during follow-up between patients w/ and w/o depressive symptoms.	U	Median %:<br>w/ symptoms: 2.82%<br>w/o symptoms: 1.89%<br>**p=.02**
				Comparison of the % of days having scheduled visits during follow-up between patients w/	M	p=.09

				and w/o depressive symptoms while controlling for age and income.		
Lozier (2018) [45]	517<br>Chronic pain	Patient Health Questionnaire	Engagement in clinician-directed non-pharmacological treatments<br>→ no/low/moderate/high engagement	Comparison of level of depressive symptoms between engagement groups of clinician-directed non-pharmacological treatments.	U	High engagement: 12.2<br>Moderate engagement: 9.5<br>Low engagement: 9.2<br>No engagement: 9.4<br>**p=.03**
				Regression investigating the influence of level of depressive symptoms on level of engagement in clinician directed non-pharmacological treatments while also accounting for site (NS), age (S), gender (NS), opioid dose (NS), ethnicity (NS), education (NS), pain disability (S) and self-efficacy (NS). (Resulting in an aOR presenting the chance of being in a higher engagement category.)	M	OR: 1.00; 95%CI: .97-1.04<br>NS
Mann (2017) [48]	702<br>Chronic pain	Patient Health Questionnaire-9<br>→ None/mild depressive symptoms vs moderate/high	Number of visits w/ GP, specialist or walk-in clinic<br>→ high vs low clinic use	Relative risk analysis investigating the influence of showing moderate/high depressive symptoms (reference: none/mild) on the likelihood of having high clinic use (reference: low use).	U	RR: 2.24<br>**95%CI : 1.42-3.53**
				Regression investigating the influence of showing moderate/high depressive symptoms (reference: no/mild symptoms) on the likelihood of having high clinic use (reference: low use) while also accounting for neuropathic mechanisms, pain timing, pain intensity, diagnosis of back problems, diagnosis of probable nerve damage, use of prescription medication, use of invasive	M	NS (omitted from final model)

				therapy (all above NS – omitted from final model), self-efficacy scores (S), number of pain locations (S) and presence of comorbidities (S).		
Mourad (2016) [54]	552<br>Non-cardiac chest pain	Patient Health Questionnaire-9	Frequency of pain-related visits<br>→ low: <2; high: 2-3; very high: >3 visits	Comparison of level of depressive symptoms between the different frequency of visits groups.	U	Very high: 10.4 ± 7.1<br>High: 7.3 ± 5.9<br>Low: 5.1 ± 5.0<br>**p<.001**
			Frequency of pain-related visits<br>→ low: ≤ 1 visit; high: ≥ 2 visits	Regression investigating the influence of level of depressive symptoms on the frequency of pain-related visits (reference: low frequency) while also accounting for age, sex, multi-morbidity, cardiac anxiety (S) and fear of body sensations (NS).	M	OR: 1.02; 95%CI: .98-1.06<br>NS
Mourad (2018) [55]	552<br>Non-cardiac chest pain	Patient Health Questionnaire-9	Frequency of seeking care for pain	Structural equation model investigating the influence of level of depressive symptoms on the frequency of healthcare visits while also accounting for somatization (NS), fear of body sensations (NS) and cardiac anxiety (S).	M	NS
Newman (2018)[5] [59]	290<br>Chronic pain	Patient Health Questionnaire-9	Number of pain-related consultations	Correlation	U	r=.26<br>**p<.01**
				Regression investigating the influence of level of depressive symptoms on the number of pain-related consultations while also accounting for demographics (age (NS), sex (NS) and race (S)), socioeconomic variables (poverty status (NS), education (NS) and literacy (NS)) and pain-related variables (physical function (NS), pain severity (NS), pain interference (NS), number of pain sites (NS)	M	β=.21<br>**p=.02**

				and types (NS), opioid use (S) and pain catastrophizing (NS)).		
Philpot (2018)[5] [63]	772<br>Chronic non-cancer pain participating in an opioid management program	Patient Health Questionnaire-9 (PHQ-9)<br>→ Depressive symptoms yes/no (baseline)	Decreases in specialist visits<br>→ yes/no (post-treatment)	Regression investigating the influence of presence of depressive symptoms (reference: no symptoms) on the likelihood of a decrease in specialist visits (reference: no decrease).	U	NS
			Decreases in primary care visits<br>→ yes/no (post-treatment)	Regression investigating the influence of presence of depressive symptoms (reference: no depressive symptoms) on the likelihood of a decrease in primary care visits (reference: no decrease).	U	p<.15
				Regression investigating the influence of presence of depressive symptoms (reference: no depressive symptoms) on the likelihood of a decrease in primary care visits (reference: no decrease), while also accounting for race (NS), gender (NS), marital status (NS), comorbidity (S), PHQ-9 functional status (NS), anxiety symptoms (GAD-7) (S) and GAD-7 functional status (NS).	M	NS
Shmagel (2016) [67]	700<br>Chronic low back pain	Patient Health Questionnaire-9<br>→ Mild, moderate, moderately severe and severe depressive symptoms	Number of healthcare visits<br>→ Frequent/normal users vs low users	Regression investigating the influence of presence of mild, moderate, moderately severe and severe depression (reference: no depression) on the likelihood of having frequent HCU (reference: low use) while controlling for age, gender, race, education level and number of medical comorbidities.	M	Mild: aOR: 1.74; 95%CI: .82 – 3.65<br>Moderate: aOR: 2.63; 95%CI: 1.19-5.86<br>Moderately severe: aOR: 5.09; 95%CI: 2.58-10.03<br>Severe: aOR: 5.55; 95%CI: 1.27-24.18<br>**p<.0001**
Tremblay (2018) [72]	428<br>Non-cardiac chest pain	Hospital Anxiety and Depression Scale –	Number of healthcare visits (primary care, specialists and ER)	Regression to investigate the influence of level of depressive symptoms on the number of healthcare visits.	U	IRR: 1.07; 95%CI: 1.03-1.11<br>**p<.001**

		Depression subscale		Regression to investigate the influence of level of depressive symptoms on the number of healthcare visits while also accounting for cardiac anxiety (S), presence of panic disorder (NS), pain frequency (S), pain intensity (NS), pain interference (S), presence of medical condition (S) and gastrointestinal symptoms (NS).	M	IRR: 1.01; 95%CI: .98-1.05<br>p=.52
Tsuji<br>(2019) [73]	565<br>Osteoarthritis	Patient Health Questionnaire-9<br>→ moderate/severe vs mild/no depressive symptoms	Number of physician visits	Comparison of number of physician visits between patients w/ moderate/severe depressive symptoms and those w/ mild/no depressive symptoms.	U	Moderate/severe: 21.3 ± 29.5<br>Mild/no: 10.7 ± 13.6<br>**p<.001**
				Regression investigating the influence of level of depressive symptoms on the number of physician visits while controlling for age, marital status, employment status and smoking status.	M	Adjusted means:<br>Moderate/severe: 21.69 ± 3.10<br>Mild/no: 11.79 ± .68<br>**p<.001**
Ullrich<br>(2013) [74]	146<br>Spinal cord injury (SCI) w/ pain	Center for Epidemiological Studies Depression Scale<br>→ depressive symptoms yes vs no<br>(measured in study year 1)	Number of SCI service visits (during 3y study duration)	Comparison of the number of outpatient visits between patients w/ pain and depression and those w/ pain only while controlling for age, medical comorbidities and level of SCI.	M	Positive association<br>**significant**
			Number of SCI psychologist visits<br>(during 3y study duration)	Comparison of the number of psychologist visits between patients w/ pain and depression and those w/ pain only while controlling for age, medical comorbidities and level of SCI.	M	Positive association<br>**significant**
Von Korff<br>(2007)[5] [81]	2,010<br>Back pain, TMD pain and headache	Symptom Checklist - Depression subscale	Number of ambulatory healthcare visits<br>→ high vs low frequency users	Comparison of level of depressive symptoms between high vs low frequency healthcare users.	U	Low frequency: .86 ± .73<br>High frequency: 1.06 ± .81<br>**p<.0001**
Wideman	202			Correlation	U	r=.306

(2011) [83]	Musculoskeletal neck/back injury undergoing a 7w PT intervention	Beck Depression Inventory (assessed after PT intervention)	Use of PT, psychology, massage therapy and other medical services → yes/no for each, summed into 0-4 score for use of different healthcare services (assessed 1y after baseline)			**p<.01**	
				Regression investigating the influence of level of depressive symptoms on the amount of different healthcare services used while controlling for pre-treatment opioid use (S) and post-treatment pain intensity (S), pain catastrophizing (NS), kinesiophobia (NS) and pain self-efficacy (NS).	M	β=.117<br>NS	
Williams (2018)[5] [86]	95<br>Sickle cell disease pain	Self-designed question: depressive symptoms → yes/no	Number of day hospital visits	Comparison of number of day hospital visits between patients w/ and w/o depressive symptoms while controlling for study site.	M	p=.587	
Depressive symptoms x emergency HCU							
Alschuler (2012) [1]	161<br>Multiple sclerosis w/ pain	Patient Health Questionnaire-9 → Depressive symptoms yes vs no	Number of ER visits	Comparison of the number of ER visits between patients w/ and w/o depressive symptoms.	U	w/: .54 ± 1.66<br>w/o: .05 ± .26<br>NS	<u>Univariate</u><br>?<br>3/7 – 43% positive associations<br><br><u>Multivariate</u><br>00<br>1/7 – 14% positive associations
Gil (2004) [23]	41<br>Sickle cell disease	Daily Mood Scale – Negative mood subscale (baseline)	ER visit on the same day → yes/no	Regression investigating the influence of negative mood on the likelihood of having an ER visit on the same day (reference: no visit) while controlling for level of pain.	M	NS	
			ER visit on the next day → yes/no	Regression investigating the influence of negative mood on the likelihood of having an ER visit on the next day (reference: no visit) while controlling for level of pain.	M	NS	
			ER visit 2d later → yes/no	Regression investigating the influence of negative mood on the likelihood of having an ER visit 2d later (reference: no visit) while controlling for level of pain.	M	NS	

Levenson (2008) [43]	232<br>Sickle cell disease (SCD)	Patient Health Questionnaire-9<br>→ Depressive symptoms yes/no (baseline)	% of days having unscheduled physician visits for SCD during 6m follow-up	Comparison of the % of days having unscheduled visits during follow-up between patients w/ and w/o depressive symptoms.	U	Median %:<br>w/ symptoms: 1.63%<br>w/o symptoms: 1.26%<br>p=.31
			% of days having ER visits for SCD during 6m follow-up	Comparison of the % of days having ER visits between patients w/ and w/o depressive symptoms.	U	Median %:<br>w/ symptoms: 1.65%<br>w/o symptoms: 1.35%<br>p=.34
Mann (2017) [48]	702<br>Chronic pain	Patient Health Questionnaire-9<br>→ None/mild depressive symptoms vs moderate/high	Number of ER visits<br>→ high vs low ER use	Relative risk analysis investigating the influence of presence of moderate/high depressive symptoms (reference: none/mild) on the likelihood of high ER use (reference: low use).	U	RR: 2.02<br>**95%CI : 1.42-2.89**
				Regression investigating the influence of showing moderate/high depressive symptoms (reference: no/mild symptoms) on the likelihood of having high ER use (reference: low use) while also accounting for marital status, diagnosis of other pain condition, pain timing, neuropathic mechanisms, diagnosis of probable nerve damage, diagnosis of arthritis, use of prescription medication, use of chiropractic and/or massage therapy (all above NS – omitted from final model), pain self-efficacy (S), presence of comorbidities (S) and use of other therapy or intervention (S).	M	NS (omitted from final model)
Philpot (2018)[5] [63]	772<br>Chronic non-cancer pain participating in an opioid management program	Patient Health Questionnaire-9 (PHQ-9)<br>→ Depressive symptoms yes/no (baseline)	Decreases in ER visits<br>→ yes/no (post-treatment)	Regression investigating the influence of presence of depressive symptoms (reference: no symptoms) on the likelihood of a decrease in ER visits (reference: no decrease).	U	Negative association<br>**p=.003**
				Regression investigating the influence of presence of	M	OR: 2.5; 95%CI: 1.2-5.2<br>**p=.02**

				depressive symptoms (reference: no symptoms) on the likelihood of a decrease in ER visits (reference: no decrease) while also accounting for race (S), marital status (NS), comorbidity index (NS), PHQ-9 functional status (NS), presence of anxiety symptoms (GAD) (NS) and GAD functional status (NS).			
Tsuji (2019) [73]	565 Osteoarthritis	Patient Health Questionnaire-9 → Moderate/severe vs mild/no depressive symptoms	Number of ER visits	Comparison of number of ER visits between patients w/ moderate/severe depressive symptoms and those w/ mild/no depressive symptoms.	U	Moderate/severe: 3.0 ± 13.4<br>Mild/no: .3 ± 2.1<br>**p<.001**	
				Regression investigating the influence of level of depressive symptoms on the number of ER visits while controlling for age, marital status, employment status and smoking status.	M	Adjusted means:<br>Moderate/severe: 3.12 ± .52<br>Mild/no: .31 ± .03<br>**p<.001**	
Villani (2010)[5] [78]	465 Migraine	Beck Depression Inventory	Number of ER visits → Repeaters vs non-repeaters of ER visits	Regression investigating the influence of level of depressive symptoms on the likelihood of repeated ER use (reference: no repeated ER use).	U	OR: 4.250; 95%CI: 1.463-12.351<br>**p=.008**	
Williams (2018)[5] [86]	95 Sickle cell disease pain	Self-designed question: depressive symptoms → yes/no	Number of ER visits	Comparison of number of ER visits between patients w/ and w/o depressive symptoms while controlling for study site.	M	p=.638	
Depressive symptoms x hospitalizations							
Cronin (2019) [12]	201 Sickle cell disease	Patient Health Questionnaire-2 (PHQ-2)	Having a readmission to the hospital → yes/no	Logistic regression investigating the influence of PHQ-2 score on the likelihood of being readmitted to the hospital (reference: no readmission) while also accounting for age (NS), sex (NS), education (NS), ability to pay bills (S), literacy	M	OR: 1.18; 95%CI: .94-1.49<br>p=.145	Univariate<br>?<br><4<br><br>Multivariate<br>00<br>1/9 – 11%

				(NS), spirituality (S) and social support (NS).			positive associations
Gil (2004) [23]	41 Sickle cell disease	Daily Mood Scale – Negative mood subscale (baseline)	Hospitalization on the same day → yes/no	Regression investigating the influence of negative mood on the likelihood of being hospitalized on the same day (reference: no hospitalization) while controlling for level of pain.	M	NS	
			Hospitalization on the next day → yes/no	Regression investigating the influence of negative mood on the likelihood of being hospitalized on the next day (reference: no hospitalization) while controlling for level of pain.	M	NS	
			Hospitalization 2d later → yes/no	Regression investigating the influence of negative mood on the likelihood of being hospitalized 2d later (reference: no hospitalization) while controlling for level of pain.	M	NS	
Levenson (2008) [43]	232 Sickle cell disease (SCD)	Patient Health Questionnaire-9 → Depressive symptoms yes/no (baseline)	% of days having a hospitalization for SCD during 6m follow-up	Comparison of the % of days having a hospitalization during follow-up between patients w/ and w/o depressive symptoms.	U	Median %:<br>w/ symptoms: 2.77%<br>w/o symptoms: 2.81%<br>p=.51	
Philpot (2018)[5] [63]	772 Chronic non-cancer pain participating in an opioid management program	Patient Health Questionnaire-9 (PHQ-9) → Depressive symptoms yes/no (baseline)	Decreases in hospitalizations → yes/no (post-treatment)	Regression investigating the influence of presence of depressive symptoms (reference: no symptoms) on the likelihood of a decrease in hospitalizations (reference: no decrease).	U	Negative association<br>**p=.009**	
				Regression investigating the influence of presence of depressive symptoms (reference: no symptoms) on the likelihood of a decrease in hospitalizations (reference: no decrease) while also accounting for race (NS), marital status (NS), comorbidity	M	NS	

				index (S), education (NS), current pain (NS), PHQ-9 functional status (NS), presence of anxiety symptoms (GAD-7) (NS), GAD-7 functional status (NS) and prescribed opioid dose (NS).			
Tsuji (2019) [73]	565<br>Osteoarthritis	Patient Health Questionnaire-9<br>→ moderate/severe vs mild/no depressive symptoms	Number of hospitalizations	Comparison of number of hospitalizations between patients w/ moderate/severe depressive symptoms and those w/ mild/no depressive symptoms.	U	Moderate/severe: 4.3 ± 15.7<br>Mild/no: 1.3 ± 5.6<br>**p=.002**	
				Regression investigating the influence of level of depressive symptoms on the number of hospitalizations while controlling for age, marital status, employment status and smoking status.	M	Adjusted means:<br>Moderate/severe: 5.15 ± .88<br>Mild/no: 1.39 ± .10<br>**p<.001**	
Ullrich (2013) [74]	146<br>Spinal cord injury (SCI) w/ pain	Center for Epidemiological Studies Depression Scale<br>→ depressive symptoms yes vs no<br>(measured in study year 1)	Number of inpatient admissions at SCI unit<br>(during 3y study duration)	Comparison of the number of inpatient admissions between patients w/ pain and depression and those w/ pain only, while controlling for age, medical comorbidities and level of SCI.	M	Pain & depression: 3.8 (mean)<br>Pain alone: 3.6 (mean)<br>NS	
			Number of inpatient days at SCI unit<br>(during 3y study duration)	Comparison of the number of inpatient days between patients w/ pain and depression and those w/ pain only while controlling for age, medical comorbidities and level of SCI.	M	Pain & depression: 52.0 (mean)<br>Pain alone: 42.6 (mean)<br>NS	
Williams (2018)[5] [86]	95<br>Sickle cell disease pain	Self-designed question: depressive symptoms<br>→ yes/no	Number of hospitalizations	Comparison of number of hospitalizations between patients w/ and w/o depressive symptoms while controlling for study site.	M	p=.701	
Depressive symptoms x CAM use							
Alschuler (2012) [1]	161<br>Multiple sclerosis w/ pain	Patient Health Questionnaire-9	Number of chiropractor visits	Comparison of the number of chiropractor visits between	U	w/: 1.69 ± 5.53<br>w/o: 1.31 ± 4.82<br>NS	<u>Univariate</u><br>?<br><4

		→ depressive symptoms yes vs no		patients w/ and w/o depressive symptoms.			<u>Multivariate</u><br>?<br><4
Harding (2019) [28]	127<br>Chronic pain	PROMIS Emotional distress – Depression subscale	Number of different types of self-management strategies used	Correlation	U	r=.22<br>**p<.05**	
				Regression investigating the influence of depressive symptoms on the number of different types of self-management strategies used while also accounting for age (NS), gender (NS), pain intensity (NS), pain interference (NS), anxiety symptoms (NS), PTSD (NS) and sleep (NS).	M	β=.03; 95%CI: -.05-.11<br>p=.512	
Lozier (2018) [45]	517<br>Chronic pain	Patient Health Questionnaire	Engagement in self-directed non-pharmacological treatments<br>→ no/low/ moderate/high engagement	Comparison of level of depressive symptoms between engagement groups of self-directed non-pharmacological treatments.	U	High engagement: 11.0<br>Moderate engagement: 9.1<br>Low engagement: 9.3<br>No engagement: 9.5<br>p=.24	
				Regression investigating the influence of level of depressive symptoms on the level of engagement in self-directed non-pharmacological treatments while also accounting for site (NS), age (NS), gender (NS), opioid dose (NS), ethnicity (NS), education (S), pain disability (S) and self-efficacy (NS). (Resulting in an aOR presenting the chance of being in a higher engagement category.)	M	OR: 1.00; 95%CI: .97-1.04<br>NS	
Depressive symptoms x HCU in general							
Alschuler (2012) [1]	161<br>Multiple sclerosis w/ pain	Patient Health Questionnaire-9 (PHQ-9)	Total number of pain treatments currently used	Regression investigating the influence of PHQ-9 score on the number of pain treatments currently used.	U	β=.09<br>p=.340	<u>Univariate</u><br>?<br>2/5 – 40% positive associations
				Regression investigating the influence of PHQ-9 score on the	M	β=.000<br>p=.98	

				number of pain treatments currently used while controlling for pain intensity.			<u>Multivariate</u><br>00<br>2/7 – 29% positive associations
			Total number of pain treatments previously used	Regression investigating the influence of PHQ-9 score on the number of pain treatments previously used.	U	β=.29<br>**p=.002**	
				Regression investigating the influence of PHQ-9 score on the number of pain treatments previously used while controlling for pain intensity.	M	β=.15<br>p=.10	
Cronan (2002)[5] [10]	600<br>Fibromyalgia	Center for Epidemiological Studies Depression Scale (CES-D) (baseline)	Total HCU (number of contacts, tests and medication) during past year	Correlation	U	r=.09<br>**p<.05**	
			Total HCU (number of contacts, tests and medication) 1y after treatment initiation	Correlation	U	r=.02<br>p>.05	
				Regression investigating the influence of baseline CES-D score on the amount of HCU at follow-up while also accounting for baseline health status (NS), ethnicity (S), comorbidity (S), education (NS), income (NS), age (S), employment (NS), social support (NS), baseline HCU (S), coping (NS), helplessness (NS) and self-efficacy (NS).	M	b=.00; 95%CI: -.00-.01<br>p=.5	
Görge (2017) [24]	688<br>Chronic low back pain	Pain Coping Questionnaire – Helplessness and depression subscale (FESV-D)	Total HCU (GP, PT, specialists, psychotherapy, complementary and massage therapists and hospital admissions)	Regression investigating the influence of FESV-D score on the amount of HCU while also accounting for gender (S), hours of work (S), days on sick leave (S), activity beliefs (S) and fatalistic external locus of control (S).	M	β=.187<br>**p<.001**	
Grant (2000) [25]	43<br>Sickle cell disease	Center for Epidemiological Studies – Depression Scale	Frequency of HCU (ER visits, hospitalizations and consultations w/ providers)	Regression investigating the influence of depressive symptoms on HCU while also	M	NS	

				accounting for age, sex, phenotype and complications.			
Harding (2019) [28]	127<br>Chronic pain	PROMIS Emotional distress - Depression subscale	Number of different types of provider management used for pain	Correlation	U	r=.12<br>p>.05	
				Regression investigating the influence of depressive symptoms on the number of different types of provider management used while also accounting for age (NS), gender (NS), pain intensity (NS), pain interference (NS), anxiety symptoms (NS), PTSD (NS) and sleep (NS).	M	β=.01; 95%CI: -.05-.07<br>p=.722	
Woodhouse (2016) [88]	219<br>Neck/low back pain	Hospital Anxiety and Depression Scale – Depression subscale<br>→ Depressive symptoms yes/no (baseline)	Future conventional care use (physicians, PT, chiropractors, psychologists, prescribed medications and use of both alternative en conventional care)<br>→ yes/no	Regression investigating whether baseline presence of depressive symptoms (reference: no depressive symptoms) is predicting use of conventional care (reference: no conventional care) while controlling for age, sex, time of follow-up, marital status, work-related factors and socioeconomic status.	M	RD: 13<br>**95%CI: 1-25**	
Fear-avoidance beliefs x pain medication use							
Wideman (2011) [83]	202<br>Musculoskeletal neck/back injury undergoing a 7w PT intervention	Tampa Scale or Kinesiophobia (assessed after PT intervention)	Use of OTC NSAID's, opioids, prescription anti-inflammatory drugs or psychotropic drugs<br>→ yes/no for each, summed into 0-4 score for use of different pain medications<br>(assessed 1y after baseline)	Correlation	U	r=.217<br>**p<.01**	Univariate<br>?<br><4<br><br>Multivariate<br>?<br><4
				Regression investigating the influence of level of kinesiophobia on the amount of different pain medications used while controlling for sex (S), pain duration (NS), pre-treatment opioid use (S) and post-treatment pain intensity (S), pain catastrophizing (NS), depressive symptoms (NS) and pain self-efficacy (S).	M	β=.013<br>NS	
Fear-avoidance beliefs x consultations							

Demmelmaier (2010) [15]	42<br>First-episode back pain	Tampa Scale for Kinesiophobia-2 (baseline)	Number of consultations w/ healthcare providers at follow-up	Correlation	U	NS	<u>Univariate</u><br>?<br><4<br><br><u>Multivariate</u><br>00<br>1/4 – 25% positive associations
	271<br>Chronic back pain	Tampa Scale for Kinesiophobia-2 (baseline)	Number of consultations w/ healthcare providers at follow-up	Correlation	U	NS	
Görge (2017) [24]	688<br>Chronic low back pain	Fear-Avoidance Beliefs Questionnaire – Physical activity beliefs subscale (baseline)	Number of PT visits 6m post-rehabilitation	Regression investigating the influence of baseline activity beliefs on the number of PT visits post-rehabilitation while also accounting for baseline PT visits (S), gender (S), inability to work (S), hours of work (NS), days on sick leave (S), helplessness and depression (S), coping (experience of competencies) (NS) and fatalistic external locus of control (NS).	M	β=-.071<br>NS	
Keeley (2008) [38]	108<br>Chronic low back pain	Fear Avoidance Beliefs Questionnaire – Physical activity beliefs subscale (baseline)	Number of consultations w/ healthcare providers during follow-up	Regression investigating the influence of baseline activity beliefs on the number of healthcare consultations at follow-up while also accounting for age (NS), education (NS), cause of pain (S), duration of pain (NS), depressive and/or anxiety symptoms (NS), work beliefs (S) and social stress (back pain-related (S) and -independent (NS)).	M	IRR=1.01<br>p=.46	
		Fear Avoidance Beliefs Questionnaire – Work beliefs subscale (baseline)	Number of consultations w/ healthcare providers during follow-up	Regression investigating the influence of baseline work beliefs on the number of healthcare consultations at follow-up while also accounting for age (NS), education (NS), cause of pain (S), duration of pain (NS), depressive and/or anxiety symptoms (NS), activity beliefs (NS) and social stress (back pain-related (S) and -independent (NS)).	M	IRR=1.02<br>**p=.009**	

Wideman (2011) [83]	202 Musculoskeletal neck/back injury undergoing a 7w PT intervention	Tamp Scale of Kinesiophobia (assessed after PT intervention)	Use of PT, psychology, massage therapy and other medical services → yes/no for each, summed into 0-4 score for use of different healthcare services (assessed 1y after baseline)	Correlation	U	r=.191 **p<.01**	
				Regression investigating the influence of level of kinesiophobia on the amount of different healthcare services used while controlling for pre-treatment opioid use (S) and post-treatment pain intensity (S), pain catastrophizing (NS), depressive symptoms (NS) and pain self-efficacy (NS).	M	β=.005 NS	
Fear-avoidance beliefs x HCU in general							
Görge (2017) [24]	688 Chronic low back pain	Fear-Avoidance Beliefs Questionnaire – Physical activity beliefs subscale (baseline)	Total HCU (GP, PT, specialists, psychotherapy, complementary and massage therapists and hospital admissions)	Regression investigating the influence of activity beliefs score on the amount of HCU while also accounting for gender (S), hours of work (S), days on sick leave (S), helplessness and depressive symptoms (S) and fatalistic external locus of control (S).	M	β=-.081 **p<.05**	<u>Multivariate</u> ? <4
Health worry x consultations							
Von Korff (2007)[5] [81]	2,010 Back pain, TMD pain and headache	Numeric Rating Scale for pain worry	Number of ambulatory healthcare visits → High vs low frequency users	Comparison of pain worry score between high vs low frequency healthcare users.	U	Low frequency: 5.6 ± 3.0 High frequency: 5.9 ± 3.1 **p=.05**	<u>Univariate</u> ? <4
Helplessness x consultations							
Jensen (1994) [32]	94 Chronic pain participating in multidisciplinary program	Survey of Pain Attitudes – Helplessness (result of factor analysis) (change score pre-post-treatment)	Number of pain-related physician visits (change score pre-post-treatment)	Regression investigating the influence of changes in helplessness score on changes in number of physician visits while also accounting for pain as illness belief score, cognitive coping attempts, coping ratings (exercise and relaxation, illness focus strategies and keeping busy) (all above: NS – omitted from final model) and baseline amount of physician visits (S).	M	β=.34 **p<.001**	<u>Multivariate</u> ? <4
Helplessness x HCU in general							

Cronan (2002)[5] [10]	600 Fibromyalgia	Arthritis helplessness index (baseline)	Total HCU (number of contacts, tests and medication) during past year	Correlation	U	r=.08<br>NS	Univariate<br>?<br><4<br><br>Multivariate<br>?<br><4
			Total HCU (number of contacts, tests and medication) 1y after treatment initiation	Correlation	U	r=.01<br>NS	
				Regression investigating the influence of baseline level of helplessness on the amount of HCU at follow-up while also accounting for baseline health status (NS), ethnicity (S), comorbidity (S), education (NS), income (NS), age (S), employment (NS), social support (NS), baseline HCU (S), coping (NS), depressive symptoms (NS) and self-efficacy (NS).	M	b=-.07; 95%CI: -.20--.05<br>p=.24	
Negative consequences beliefs x consultations							
Biggs (2003) [3]	151 Abdominal or chest pain	Illness Perceptions Questionnaire – Consequences subscale	Number of consultations w/ healthcare providers	Regression investigating the influence of negative consequences score on the number of consultations while also accounting for education, access to confidant, pain score, recent social stress, exposure to death of a father or mother during childhood, reported childhood adversity (antipathy from father or mother, neglect, physical abuse or psychological abuse), depressive symptoms, general and symptom-related anxiety symptoms, negative illness perceptions (timeline), perceived symptom control, SF-36 scores (role limitations physical and mental, social function, energy and vitality and pain) (all above NS – omitted from final model), sex (S), SF-36	M	NS (omitted from final model)	Univariate<br>?<br><4<br><br>Multivariate<br>?<br><4

			scores (physical function, health perception and mental health) (S), marital status (S), diagnosis (S), death of a sibling (S) and reported sexual abuse (S).		
		Number of GP consultations	Regression investigating the influence of negative consequences score on the number of GP consultations while also accounting for education, access to confidant, pain score, recent social stress, exposure to death of a father or mother during childhood, reported childhood adversity (sexual abuse, antipathy from mother, neglect, physical abuse or psychological abuse), depressive symptoms, general and symptom-related anxiety symptoms, perceived symptom control, SF-36 scores (role limitations physical and mental, social function, energy and vitality, physical function, mental health and health perception) (all above NS – omitted from final model), sex (S), SF-36 pain score (S), marital status (S), illness perception timeline score (S), diagnosis (S), death of a sibling (S) and reported antipathy from father (S).	M	NS (omitted from final model)
		Number of consultations w/ other providers than GP	Regression investigating the influence of negative consequences score on the number of consultations w/ other providers than GP while also accounting for marital status, diagnosis, education, access to confidant, recent social	M	NS (omitted from final model)

				stress, exposure to death of a sibling, father or mother during childhood, reported childhood adversity (sexual abuse, antipathy from father or mother, neglect, physical abuse or psychological abuse), depressive symptoms, general and symptom-related anxiety symptoms, negative illness perceptions (timeline), perceived symptom control, SF-36 scores (pain score, role limitations physical and mental, social function, energy and vitality, physical function and health perception) (all above NS – omitted from final model), sex (S), SF-36 (health perception, mental health and physical function) (S) and pain score (S).			
Jensen (1994) [32]	94 Chronic pain participating in multidisciplinary program	Survey of Pain Attitudes – Harm subscale (change score pre-post-treatment)	Number of pain-related physician visits (change score pre-post-treatment)	Correlation	U	r=-.09<br>NS	
		Survey of Pain Attitudes – Disability subscale (change score pre-post-treatment)	Number of pain-related physician visits (change score pre-post-treatment)	Correlation	U	r=-.15<br>NS	
Negative illness beliefs x consultations							
Biggs (2003) [3]	151 Abdominal or chest pain	Illness Perceptions Questionnaire – Timeline subscale	Number of consultations w/ healthcare providers	Regression investigating the influence of the timeline score on the number of consultations while also accounting for education, access to confidant, pain score, recent social stress,	M	NS (omitted from final model	<u>Univariate</u><br>?<br><4<br><br><u>Multivariate</u><br>00

				exposure to death of a father or mother during childhood, reported childhood adversity (antipathy from father or mother, neglect, physical abuse or psychological abuse), depressive symptoms, general and symptom-related anxiety symptoms, negative illness perceptions (consequences), perceived symptom control, SF-36 scores (role limitations physical and mental, social function, energy and vitality and pain) (all above NS – omitted from final model), sex (S), SF-36 scores (physical function, health perception and mental health) (S), marital status (S), diagnosis (S), death of a sibling (S) and reported sexual abuse (S).			1/5<br>20%
			Number of GP consultations	Regression investigating the influence of the timeline score on the number of GP consultations while also accounting for education, access to confidant, pain score, recent social stress, exposure to death of a father or mother during childhood, negative illness perceptions (consequences), reported childhood adversity (sexual abuse, antipathy from mother, neglect, physical abuse or psychological abuse), depressive symptoms, general and symptom-related anxiety symptoms, perceived symptom control, SF-36 scores (role limitations physical and mental, social function, energy and	M	positive association **significant**	

			vitality, physical function, mental health and health perception) (all above NS – omitted from final model), sex (S), SF-36 pain score (S), marital status (S), diagnosis (S), death of a sibling (S) and reported antipathy from father (S).			
		Number of consultations w/ other providers than GP	Regression investigating the influence of the timeline score on the number of consultations w/ other providers than GP while also accounting for marital status, diagnosis, education, access to confidant, recent social stress, exposure to death of a sibling, father or mother during childhood, reported childhood adversity (sexual abuse, antipathy from father or mother, neglect, physical abuse or psychological abuse), depressive symptoms, general and symptom-related anxiety symptoms, negative illness perceptions (consequences), perceived symptom control, SF-36 scores (pain score, role limitations physical and mental, social function, energy and vitality, physical function and health perception) (all above NS – omitted from final model), sex (S), SF-36 (health perception, mental health and physical function) (S) and pain score (S).	M	NS (omitted from final model)	

Görge (2017) [24]	688 Chronic low back pain	Control Beliefs Concerning Illness and Health Questionnaire – Fatalistic external locus of control (baseline)	Number of PT visits 6m post-rehabilitation	Regression investigating the influence of baseline fatalistic external locus of control score on the number of PT visits post-rehabilitation while also accounting for baseline PT visits (S) gender (S), inability to work (S), employment (S), hours of work (NS), days on sick leave (S), helplessness and depressive symptoms (S), coping (experience of competencies) (NS) and activity beliefs (NS).	M	β=.085 NS
Jensen (1994) [32]	94 Chronic pain participating in multidisciplinary program	Survey of Pain Attitudes – Pain as illness belief (result of factor analysis) (change score pre-post-treatment)	Number of pain-related physician visits (change score pre-post-treatment)	Regression investigating the influence of changes in pain as illness belief score on changes in number of physician visits while also accounting for cognitive coping attempts, coping ratings (exercise and relaxation, illness focus strategies and keeping busy) (all above: NS – omitted from final model), helplessness change score (S) and baseline amount of physician visits (S).	M	NS (omitted from final model)
		Survey of Pain Attitudes – Medical cure subscale (change score pre-post-treatment)	Number of pain-related physician visits (change score pre-post-treatment)	Correlation	U	r=-.22 NS
		Survey of Pain Attitudes – Solicitude subscale (change score pre-post-treatment)	Number of pain-related physician visits (change score pre-post-treatment)	Correlation	U	r=-.16 NS

		Survey of Pain Attitudes – Medication subscale (change score pre-post-treatment)	Number of pain-related physician visits (change score pre-post-treatment)	Correlation	U	$r=-.13$<br>NS	
Negative illness beliefs x HCU in general							
Görge (2017) [24]	688<br>Chronic low back pain	Control Beliefs Concerning Illness and Health Questionnaire – Fatalistic external locus of control	Total HCU (GP, PT, specialists, psychotherapy, complementary and massage therapists and hospital admissions)	Regression investigating the influence of fatalistic external locus of control score on the amount of HCU while also accounting for gender (S), hours of work (S), days on sick leave (S), helplessness and depressive symptoms (S) and activity beliefs (S).	M	$\beta=.097$<br>**$p<.05$**	<u>Multivariate</u><br>?<br><4
Psychological distress x pain medication use							
Durá-Ferrandis (2017) [17]	72<br>TMD<br>Participating in CBT intervention study	Brief Symptom Inventory (BSI)	Frequency of self-medication (change pre-post-treatment)	SEM investigating whether change in BSI score was a potential mediator of the treatment effect on frequency of self-medication next to catastrophizing (NS), pain intensity (NS), perceived control (NS) and coping strategies (distraction (S) and mental self-control (NS)).	M	SEM loading: .06<br>NS	<u>Univariate</u><br>00<br>0/4 – 0%<br><br><u>Multivariate</u><br>?<br><4
Trask (2001) [71]	292<br>Headache	Brief Symptom Inventory<br>→ low/medium/ high distress	Number of symptomatic medications used	To compare the number of symptomatic medications used between the distress clusters.	U	Low: 1.94 ± 1.04<br>Medium: 1.82 ± 1.01<br>High: 2.02 ± .98<br>NS	
			Number of preventive medications used	To compare the number of preventive medications used between the distress clusters.	U	Low: 1.37 ± 1.25<br>Medium: 1.42 ± 1.34<br>High: 1.52 ± 1.32<br>NS	
Zebenholzer (2016) [89]	232<br>Episodic headache	Hospital Anxiety and Depression Scale	Prophylactic medication use for headache for ≥3m<br>→ yes/no	Chi²	U	w/ symptoms: 85.1% using ≥3m<br>w/o symptoms: 73.4% using ≥3m<br>NS	

		→ Anxiety and/or depressive symptoms yes/no					
	160<br>Chronic headache	Hospital Anxiety and Depression Scale<br>→ Anxiety and/or depressive symptoms yes/no	Prophylactic medication use for headache for ≥3m<br>→ yes/no	Chi²	U	w/ symptoms: 82% using ≥3m<br>w/o symptoms: 80.8% using ≥3m<br>NS	
Psychological distress x consultations							
Biggs<br>(2003) [3]	151<br>Abdominal or chest pain	SF-36 Mental health	Number of consultations w/ healthcare providers	Regression investigating the influence of SF-36 mental health score on the number of consultations while also accounting for education, access to confidant, pain score, recent social stress, exposure to death of a father or mother during childhood, reported childhood adversity (antipathy from father or mother, neglect, physical abuse or psychological abuse), depressive symptoms, general and symptom-related anxiety symptoms, negative illness perceptions (consequences and timeline), perceived symptom control, SF-36 scores (role limitations physical and mental, social function, energy and vitality and pain) (all above NS – omitted from final model), sex (S), SF-36 scores (physical function, health perception) (S), marital status (S), diagnosis (S), death of a sibling (S) and reported sexual abuse (S).	M	β=.030 (negative association w/ HCU due to scoring SF-36)<br>**p=.009**	<u>Univariate</u><br>+<br>8/8 - 100%<br><br><u>Multivariate</u><br>00<br>1/13 – 8% positive associations

		Number of GP consultations	Regression investigating the influence of SF-36 mental health score on the number of GP consultations while also accounting for education, access to confidant, pain score, recent social stress, exposure to death of a father or mother during childhood, reported childhood adversity (sexual abuse, antipathy from mother, neglect, physical abuse or psychological abuse), depressive symptoms, general and symptom-related anxiety symptoms, negative consequences beliefs, perceived symptom control, SF-36 scores (role limitations physical and mental, social function, energy and vitality, physical function and health perception) (all above NS – omitted from final model), sex (S), SF-36 pain score (S), marital status (S), illness perception timeline score (S), diagnosis (S), death of a sibling (S) and reported antipathy from father (S).	M	NS (omitted from final model)	
		Number of consultations w/ other providers than GP	Regression investigating the influence of SF-36 mental health score on the number of consultations w/ other providers than GP while also accounting for marital status, diagnosis, education, access to confidant, recent social stress, exposure to death of a sibling, father or mother during childhood, reported childhood adversity (sexual abuse, antipathy from father or mother, neglect,	M	**significant** (direction not stated; in line w/ results for total consultations a negative association was chosen)	

				physical abuse or psychological abuse), depressive symptoms, general and symptom-related anxiety symptoms, negative illness perceptions (consequences and timeline), perceived symptom control, SF-36 scores (pain score, role limitations physical and mental, social function, energy and vitality, physical function and health perception) (all above NS – omitted from final model), sex (S), SF-36 (health perception and physical function) (S) and pain score (S).		
Keeley (2008) [38]	108<br>Chronic low back pain	Hospital Anxiety and Depression Scale (HADS) – Total score (baseline)	Number of consultations w/ healthcare providers during follow-up	Regression investigating the influence of baseline HADS score on the number of healthcare consultations at follow-up while also accounting for age (NS), education (NS), cause of pain (S), duration of pain (NS), activity beliefs (NS), work beliefs (S) and social stress (back pain-related (S) and -independent (NS)).	M	IRR=1.00<br>p=.83
Kuijper (2014) [40]	330<br>Arthralgia w/o synovitis	SF-36 - Mental component score (baseline)	Number of visits w/ healthcare providers for joint symptoms 6m later	Regression investigating the influence of baseline SF-36 mental component score on the number of healthcare visits 6m later while also accounting for duration of symptoms, locus of control (chance and internal), coping, ethnicity, education, household composition, employment, BMI, fatigue, diagnosis, comorbidities (all above NS – omitted from final analysis), month (S – fixed factor), age (NS – fixed factor), sex (NS -fixed factor), pain (S), SF-	M	NS (omitted from final model)

				36 physical component (S) and locus of control (external (S)).		
	244<br>Rheumatoid arthritis	SF-36 – Mental component score (baseline)	Number of visits w/ healthcare providers for joint symptoms 6m later	Regression investigating the influence of baseline SF-36 mental component score on the number of healthcare visits 6m later while also accounting for ethnicity, education, household composition, employment, BMI, fatigue, diagnosis, comorbidities, coping, locus of control (external and internal), pain (all above NS – omitted from final analysis), month (S – fixed factor), age (NS – fixed factor), sex (NS -fixed factor), duration of symptoms (S), SF-36 physical component (S) and locus of control (chance (S)).	M	NS (omitted from final model)
Lee<br>(2008) [41]	420<br>Functional bowel disease	General Health Questionnaire-28	Number of symptom-related GP visits	Correlation	U	positive asssociation<br>**p<.05**
				Regression investigating the influence of psychological distress on number of GP visits while also accounting for more severe IBS score, symptom severity, pain duration (all above: NS – omitted from final model), duration of IBS symptoms (S), employment (S), >3 bowel movements/day (S), pain relieve by opening bowels (S) and bowel passing (S).	M	IRR: 1.01; 95%CI: 1.00-1.02<br>**p=.014**
Navabi<br>(2018)[5] [57]	432<br>Irritable bowel disease	Hospital Anxiety and Depression Scale<br>→ Depressive and/or anxiety symptoms yes/no	Number of imaging studies	Comparison of the number of imaging studies between patients w/ and w/o depressive and/or anxiety symptoms.	U	w/o symptoms: .77 ± .1<br>w/ symptoms: 1.23 ± .2<br>**p<.05**
Von Korff	816<br>Chronic pain	Symptom Checklist Revised	Ambulatory care in the year before the index visit	Correlation	U	r=.10<br>**p≤.01**

(1991)[5] [80]			(primary care, specialist visits and ER visits)	Regression investigating the influence of level of psychological distress on amount of ambulatory care use in the year before the index visit while also accounting for age (S), sex (S), chronic pain status (S) and self-rated health (S).	M	p=.153
			Ambulatory care in the year after the index visit (primary care, specialist visits and ER visits)	Correlation	U	r=.11<br>**p≤.001**
				Regression investigating the influence of level of psychological distress on amount of ambulatory care use in the year after the index visit while also accounting for age (S), sex (S), chronic pain status (S) and self-rated health (S).	M	p=.579
	203<br>TMD clinic patients	Symptom Checklist Revised	Ambulatory care in the year before the index visit (primary care, specialist visits and ER visits)	Correlation	U	r=.20<br>**p≤.01**
				Regression investigating the influence of level of psychological distress on amount of ambulatory care use in the year before the index visit while also accounting for age (NS), sex (NS), chronic pain status (S) and self-rated health (S).	M	p=.679
			Ambulatory care in the year after the index visit (primary care, specialist visits and ER visits)	Correlation	U	r=.15<br>**p≤.05**
				Regression investigating the influence of level of psychological distress on amount of ambulatory care use in the year after the index visit while also accounting for age (NS), sex (NS), chronic pain status (S) and self-rated health (S).	M	p=.939
Walker	590	Center for Epidemiologic	Number of visits to GP → high vs low use	Regression investigating the influence of presence of	U	OR: 1.92; 95%CI: 1.30-2.84<br>**p<.05**

(2016) [82]	Undergoing gynecological surgery	Studies-Depression & State Trait Anxiety Inventory – Trait form<br>→ Depressive and/or anxiety symptoms vs not		depressive and/or anxiety symptoms (reference: no symptoms) on the likelihood of having a high amount of GP visits (reference: low amount).			
				Regression investigating the influence of presence of depressive and/or anxiety symptoms (reference: no symptoms) on the likelihood of having a high amount of GP visits (reference: low amount) while also accounting for age*pain intensity (S), marital status (NS), employment status (NS), education (NS), BMI (NS), current smoker (NS), previous abdominal surgery (S), waiting time before surgery (NS), menstruation status (NS), taking hormone replacement therapy (NS), taking birth control pills (NS) and preoperative malignancy (NS).	M	aOR: 1.10; 95%CI: .69-1.75<br>NS	
			Number of specialist visits<br>→ high vs low use	Regression investigating the influence of presence of depressive and/or anxiety symptoms (reference: no symptoms) on the likelihood of having a high amount of specialist visits (reference: low amount).	U	OR: 1.95; 95%CI: 1.24-3.05<br>**p<.05**	
				Regression investigating the influence of presence of depressive and/or anxiety symptoms (reference: no symptoms) on the likelihood of having a high amount of specialist visits (reference: low amount) while also accounting for age (NS), marital status (NS), employment status (NS),	M	aOR: 1.21; 95%CI: .73-2.02<br>NS	

				education (NS), BMI (NS), current smoker (NS), previous abdominal surgery (NS), waiting time before surgery (NS), menstruation status (NS), taking hormone replacement therapy (NS), taking birth control pills (NS), preoperative malignancy (NS) and pain intensity (S).			
Psychological distress x emergency HCU							
Navabi (2018)[5] [57]	432 Irritable bowel disease	Hospital Anxiety and Depression Scale → Depressive and/or anxiety symptoms yes/no	Number of ER visits	Comparison of the number of ER visits between patients w/ and w/o depressive and/or anxiety symptoms.	U	w/o symptoms: .44 ± .1<br>w/ symptoms: .95 ± .2<br>**p<.05**	<u>Univariate</u><br>?<br><4
Psychological distress x invasive procedures							
Navabi (2018)[5] [57]	432 Irritable bowel disease	Hospital Anxiety and Depression Scale → presence of depressive and/or anxiety symptoms yes/no	Number of surgeries	Comparison of the number of surgeries between patients w/ and w/o depressive and/or anxiety symptoms.	U	w/o symptoms: .30 ± .1<br>w/ symptoms: .69 ± .1<br>p=.06	<u>Univariate</u><br>?<br><4
Psychological distress x hospitalizations							
Navabi (2018)[5] [57]	432 Irritable bowel disease	Hospital Anxiety and Depression Scale → Depressive and/or anxiety symptoms yes/no	Number of hospital admissions	Comparison of the number of hospital admissions between patients w/ and w/o depressive and/or anxiety symptoms.	U	w/o symptoms: .36 ± .1<br>w/ symptoms: .60 ± .1<br>**p<.05**	<u>Univariate</u><br>?<br><4
Psychological distress x HCU in general							
Lentz (2018) [42]	246 Musculoskeletal pain	OSPRO Yellow Flag Tool – 10-item version (OSPRO-YF-10) (baseline)	Use of any healthcare (opioid use, injections, surgery, diagnostic tests or imaging or ER visits) after PT treatment	Regression investigating the influence of baseline OSPRO-YF-10 score on the likelihood of using any healthcare (reference: no use) after PT treatment while	M	NS (omitted from final model)	<u>Multivariate</u><br>?<br><4

			→ yes/no	also accounting for age, sex, race, anatomical region of pain, insurance, surgery for current condition, OSPRO Review of Systems score (10-item + 13 items), baseline OSPRO-YF remaining 7 items score, OSPRO-YF-10 change score, baseline pain intensity, change in disability, (all above: NS – omitted from final model), chronicity (NS), comorbidity (S), baseline disability (S) and change in pain intensity (S).		
		OSPRO Yellow Flag Tool (OSPRO-YF) – remaining 7 items (baseline)	Use of any healthcare (opioid use, injections, surgery, diagnostic tests or imaging or ER visits) after PT treatment → yes/no	Regression investigating the influence of baseline OSPRO-YF remaining 7 items score on the likelihood of using any healthcare (reference: no use) after PT treatment while also accounting for age, sex, race, anatomical region of pain, insurance, surgery for current condition, OSPRO Review of Systems score (10-item + 13 items), baseline 10-item OSPRO-YF score, 10-item OSPRO-YF change score, baseline pain intensity, change in disability, (all above: NS – omitted from final model), chronicity (NS), comorbidity (S), baseline disability (S) and change in pain intensity (S).	M	NS (omitted from final model)
		OSPRO Yellow Flag Tool - 10-item version (OSPRO-YF-10) (baseline-to-4w change score)	Use of any healthcare (opioid use, injections, surgery, diagnostic tests or imaging or ER visits) after PT treatment → yes/no	Regression investigating the influence of baseline-to-4w change in OSPRO-YF-10 score on the likelihood of using any healthcare (reference: no use) after PT treatment while also accounting for age, sex, race, anatomical region of pain,	M	NS (omitted from final model)

				insurance, surgery for current condition, OSPRO Review of Systems score (10-item + 13 items), baseline 10-item OSPRO-YF score, OSPRO-YF 7 remaining items score, baseline pain intensity, change in disability, (all above: NS – omitted from final model), chronicity (NS), comorbidity (S), baseline disability (S) and change in pain intensity (S).			
Stress x pain medication use							
Elander (2014) [19]	112 General population w/ pain	Depression, Anxiety and Stress Scale – Stress subscale	Frequency of prescription pain medication use	Correlation	U	r=.17 NS	<u>Univariate</u> ? <4
			Frequency of OTC pain medication use	Correlation	U	r=-.09 NS	
Gil (2004) [23]	41 Sickle cell disease	VAS for stress (baseline)	Using prescription pain medication on the same day → yes/no	Regression investigating the influence of stress on the likelihood of using prescription pain medication on the same day (reference: no use) while controlling for level of pain.	M	NS	<u>Multivariate</u> ? <4
			Using prescription pain medication on the next day → yes/no	Regression investigating the influence of stress on the likelihood of using prescription pain medication on the next day (reference: no use) while controlling for level of pain.	M	NS	
			Using prescription pain medication 2d later → yes/no	Regression investigating the influence of stress on the likelihood of using prescription pain medication 2d later (reference: no use) while controlling for level of pain.	M	NS	
Stress x consultations							
Gil (2004) [23]	41 Sickle cell disease	VAS for stress (baseline)	Doctor call on the same day → yes/no	Regression investigating the influence of stress on the likelihood of having a doctor call on the same day (reference: no	M	β=.01 **p<.05**	<u>Multivariate</u> ? 2/5 – 40%

				call) while controlling for level of pain.			positive associations
			Doctor call on the next day → yes/no	Regression investigating the influence of stress on the likelihood of having a doctor call on the next day (reference: no call) while controlling for level of pain.	M	NS	
			Doctor call 2d later → yes/no	Regression investigating the influence of stress on the likelihood of having a doctor call 2d later (reference: no call) while controlling for level of pain.	M	NS	
Keeley (2008) [38]	108<br>Chronic low back pain	Life Events and Difficulties Schedule – Back pain-related social stress (baseline)	Number of consultations w/ healthcare providers during follow-up	Regression investigating the influence of baseline back pain-related social stress on the number of healthcare consultations at follow-up while also accounting for age (NS), education (NS), cause of pain (S), duration of pain (NS), depressive and/or anxiety symptoms (NS), activity beliefs (NS), work beliefs (S) and back pain-independent social stress (NS).	M	IRR=1.16; 95%CI: 1.02-1.31<br>**p=.027**	
		Life Events and Difficulties Schedule – Back pain-independent social stress (baseline)	Number of consultations w/ healthcare providers during follow-up	Regression investigating the influence of baseline back pain-independent social stress on the number of healthcare consultations at follow-up while also accounting for age (NS), education (NS), cause of pain (S), duration of pain (NS), depressive and/or anxiety symptoms (NS), activity beliefs (NS), work beliefs (S) and back pain-related social stress (S).	M	IRR=1.06; 95%CI: 1.02-1.31<br>p=.08	
Stress x emergency HCU							
Gil	41<br>Sickle cell disease	VAS for stress (baseline)	Having an ER visit on the same day	Regression investigating the influence of stress on the	M	NS	Multivariate<br>?

(2004) [23]			→ yes/no	likelihood of having an ER visit on the same day (reference: no visit) while controlling for level of pain.			<4
			Having an ER visit on the next day<br>→ yes/no	Regression investigating the influence of stress on the likelihood of having an ER visit on the next day (reference: no visit) while controlling for level of pain.	M	NS	
			Having an ER visit 2d later<br>→ yes/no	Regression investigating the influence of stress on the likelihood of having an ER visit 2d later (reference: no visit) while controlling for level of pain.	M	NS	
Stress x hospitalizations							
Gil<br>(2004) [23]	41<br>Sickle cell disease	VAS for stress (baseline)	Hospitalization on the same day<br>→ yes/no	Regression investigating the influence of stress on the likelihood of being hospitalized on the same day (reference: no hospitalization) while controlling for level of pain.	M	NS	<u>Multivariate</u><br>?<br><4
			Hospitalization on the next day<br>→ yes/no	Regression investigating the influence of stress on the likelihood of being hospitalized on the next day (reference: no hospitalization) while controlling for level of pain.	M	NS	
			Hospitalization 2d later<br>→ yes/no	Regression investigating the influence of stress on the likelihood of being hospitalized 2d later (reference: no hospitalization) while controlling for level of pain.	M	NS	
Symptom vigilance x consultations							
Demmelmaier<br>(2010) [15]	42<br>First-episode back pain	Pain Vigilance and Awareness Questionnaire (baseline)	Number of consultations w/ healthcare providers at follow-up	Correlation	U	NS	<u>Univariate</u><br>?<br>2/4 – 50% positive associations<br><br><u>Multivariate</u>
	271<br>Chronic back pain	Pain Vigilance and Awareness	Number of consultations w/ healthcare providers at follow-up	Correlation	U	NS	

		Questionnaire (baseline)					?<br><4
McCracken (1997) [50]	80<br>Chronic low back pain	Pain Vigilance and Awareness Questionnaire	Number of pain-related physician visits	Correlation	U	r=.36<br>**p<.001**	
				Regression the influence of pain attention on the number of physician visits while also accounting for age (S), education (S) and pain intensity (NS – fixed factor).	M	β=.40<br>**p=.0005**	
Mourad (2016) [54]	552<br>Non-cardiac chest pain	Cardiac Anxiety Questionnaire – Heart-focused attention	Frequency of pain-related visits → low: <2; high: 2-3; very high: >3 visits	Comparison of level of heart-focused attention between frequency of visits groups.	U	Very high: 1.7 ± .8<br>High: 1.3 ± .7<br>Low: .8 ± .6<br>**p<.001**	
**POSITIVE CEF CLUSTERS**							
Pain acceptance x pain medication use							
Elander (2014) [19]	112<br>General population w/ pain	Chronic Pain Acceptance Questionnaire	Frequency of prescription pain medication use	Correlation	U	r=-.55<br>**p<.01**	<u>Univariate</u><br>?<br>3/8 – 38% negative associations<br><br><u>Multivariate</u><br>?<br>2/5 – 40% negative associations
			Frequency of OTC pain medication use	Correlation	U	r=-.01<br>NS	
Kratz (2018) [39]	120<br>Spinal cord injury w/ chronic pain	Chronic Pain Acceptance Questionnaire – Total score	Number of pain medications used	Regression investigating the influence of level of pain acceptance on the number of pain medications used while also accounting for pain intensity (NS), number of painful body areas (S) and depressive symptoms (NS).	M	β=-.02<br>**p=.005**	
		Chronic Pain Acceptance Questionnaire (CPAQ) – pain willingness	Number of pain medications used	Regression investigating the influence of level of CPAQ pain willingness score on the number of pain medications used while also accounting for pain intensity (NS), number of painful body areas (S), depressive symptoms (NS) and CPAQ activities engagement (NS).	M	β=-.02<br>p=.1	
		Chronic Pain Acceptance Questionnaire	Number of pain medications used	Regression investigating the influence of level of CPAQ activities engagement score on	M	β=-.02<br>p=.09	

		(CPAQ) – activities engagement		the number of pain medications used while also accounting for pain intensity (NS), number of painful body areas (S), depressive symptoms (NS) and CPAQ pain willingness (NS).		
McCracken (2005 – Pain) [51]	118<br>Chronic pain	Chronic Pain Acceptance Questionnaire – Total score (baseline)	Amount of pain medication use at follow-up	Correlation	U	r=-.25<br>**p<.01**
		Chronic Pain Acceptance Questionnaire (CPAQ) – Pain willingness subscale (baseline)	Amount of pain medication use at follow-up	Correlation	U	r=-.27<br>**p<.01**
				Regression investigating the influence of CPAQ pain willingness score on the amount of pain medication use at follow-up while also accounting for age, gender, years of education, duration of pain (all above: NS – omitted from final model), CPAQ activities engagement (NS – fixed factor) and pain intensity (S).	M	β=-.23<br>**p<.05**
		Chronic Pain Acceptance Questionnaire (CPAQ) – Activities engagement subscale (baseline)	Amount of pain medication use at follow-up	Correlation	U	r=-.14<br>NS
				Regression investigating the influence of CPAQ activities engagement score on the amount of pain medication use at follow-up while also accounting for age, gender, years of education, duration of pain (all above: NS – omitted from final model), CPAQ pain willingness score (S) and pain intensity (S).	M	β=-.053<br>NS
McCracken (2005 – Beh Res Ther) [52]	108<br>Chronic pain Following treatment	Chronic Pain Acceptance Questionnaire – Total score	Number of pain medications used (changes pre-post treatment)	Correlation	U	r=-.04<br>NS

		(changes pre-post treatment)					
		Chronic Pain Acceptance Questionnaire – Pain willingness subscale (changes pre-post treatment)	Number of pain medications used (changes pre-post treatment)	Correlation	U	r=-.03<br>NS	
		Chronic Pain Acceptance Questionnaire – Activities engagement subscale (changes pre-post-treatment)	Number of pain medications used (changes pre-post treatment)	Correlation	U	r=-.05<br>NS	
Perceived symptom control x pain medication use							
Daltroy (1998)[5] [13]	222<br>Scheduled for knee or hip arthroplasty	Self-designed question for perceived pain control	Postoperative pain medication use	General linear model investigating the influence of baseline state anxiety on postoperative pain medication use while also accounting for sex, reliance in God, date of surgery, comorbidities, cemented joint, desire for information, passive range of motion, lack of a discharge plan, denial (all above: NS- omitted from final model), age (S), knee vs hip surgery (S), poor preoperative sleep quality (S), surgeon (NS), information (NS) and relaxation training (NS) .	M	NS (omitted from final model)	Multivariate<br>?<br><4
Durá-Ferrandis (2017) [17]	72<br>TMD participating in CBT intervention study	Survey of Pain Attitudes-35 – Perceived control (change pre-post-treatment)	Frequency of self-medication (change pre-post-treatment)	SEM investigating whether change in perceived control score was a potential mediator of the treatment effect on frequency of self-medication next to psychological distress (NS), pain intensity (NS), pain catastrophizing (NS) and coping	M	SEM loading: -.03<br>NS	

				strategies (distraction (S) and mental self-control (NS)).			
Perceived symptom control x consultations							
Biggs (2003) [3]	151<br>Abdominal or chest pain	Illness Perceptions Questionnaire – cure subscale	Number of consultations w/ healthcare providers	Regression investigating the influence of level of perceived symptom control on the number of consultations while also accounting for education, access to confidant, pain score, recent social stress, exposure to death of a father or mother during childhood, reported childhood adversity (antipathy from father or mother, neglect, physical abuse or psychological abuse), depressive symptoms, general and symptom-related anxiety symptoms, negative illness perceptions (consequences and timeline), SF-36 scores (role limitations physical and mental, social function, energy and vitality and pain) (all above NS – omitted from final model), sex (S), SF-36 scores (physical function, health perception and mental health) (S), marital status (S), diagnosis (S), death of a sibling (S) and reported sexual abuse (S).	M	NS (omitted from final model)	<u>Univariate</u><br>?<br><4<br><br><u>Multivariate</u><br>?<br><4
			Number of GP consultations	Regression investigating the influence of the level of perceived symptom control on the number of GP consultations while also accounting for education, access to confidant, pain score, recent social stress, exposure to death of a father or mother during childhood, negative illness perceptions (consequences), reported	M	NS (omitted from final model)	

				childhood adversity (sexual abuse, antipathy from mother, neglect, physical abuse or psychological abuse), depressive symptoms, general and symptom-related anxiety symptoms, SF-36 scores (role limitations physical and mental, social function, energy and vitality, physical function, mental health and health perception) (all above NS – omitted from final model), sex (S), SF-36 pain score (S), negative illness perceptions (timeline), marital status (S), diagnosis (S), death of a sibling (S) and reported antipathy from father (S).		
			Number of consultations w/ other providers than GP	Regression investigating the influence of the level of perceived symptom control on the number of consultations w/ other providers than GP while also accounting for marital status, diagnosis, education, access to confidant, recent social stress, exposure to death of a sibling, father or mother during childhood, reported childhood adversity (sexual abuse, antipathy from father or mother, neglect, physical abuse or psychological abuse), depressive symptoms, general and symptom-related anxiety symptoms, negative illness perceptions (consequences and timeline), SF-36 scores (pain score, role limitations physical and mental, social function, energy and vitality, physical	M	NS (omitted from final model)

				function and health perception) (all above NS – omitted from final model), sex (S), SF-36 (health perception, mental health and physical function) (S) and pain score (S).			
Jensen (1994) [32]	94<br>Chronic pain participating in multidisciplinary program	Survey of Pain Attitudes – pain control subscale (change score pre-post-treatment)	Number of pain-related physician visits (change score pre-post-treatment)	Correlation	U	r=.18<br>NS	
Von Korff (2007)[5] [81]	2,010<br>Back pain, TMD pain and headache	Numeric Rating Scale for pain control	Number of ambulatory healthcare visits<br>→ high vs low frequency users	Comparison of pain control score between high vs low frequency healthcare users	U	Low frequency: 4.2 ± 3.1<br>High frequency: 3.7 ± 3.0<br>**p<.001**	
Perceived symptom control x hospitalizations							
Daltroy (1998)[5] [13]	222<br>Scheduled for knee or hip arthroplasty	Self-designed question for perceived pain control	Length of stay	General linear model investigating the influence of baseline pain control on length of stay while also accounting for age (S), reliance in God (S), surgeon (S), date of surgery (S), comorbidities (S), cemented joint (S), greater trait anxiety (S), greater desire for information (NS), smaller passive range of motion (NS), lack of a discharge plan (NS), greater denial (NS), provision of information (NS) and relaxation training (NS).	M	p<.50	<u>Multivariate</u><br>?<br><4
Positive mood x pain medication use							
Gil (2004) [23]	41<br>Sickle cell disease	Daily Mood Scale - Positive mood subscale (baseline)	Using prescription pain medication on the same day<br>→ yes/no	Regression investigating the influence of positive mood on the likelihood of using prescription pain medication on the same day (reference: no use) while controlling for level of pain.	M	β=-.12<br>**p<.0001**	<u>Multivariate</u><br>?<br><4
			Using prescription pain medication on the next day	Regression investigating the influence of positive mood on	M	NS	

			→ yes/no	the likelihood of using prescription pain medication on the next day (reference: no use) while controlling for level of pain.			
			Using prescription pain medication 2d later<br>→ yes/no	Regression investigating the influence of positive mood on the likelihood of using prescription pain medication 2d later (reference: no use) while controlling for level of pain.	M	NS	
Positive mood x consultations							
Gil<br>(2004) [23]	41<br>Sickle cell disease	Daily Mood Scale – Positive mood subscale (baseline)	Doctor call on the same day<br>→ yes/no	Regression investigating the influence of positive mood on the likelihood of having a doctor call on the same day (reference: no call) while controlling for level of pain.	M	β=-.10<br>**p<.0001**	<u>Multivariate</u><br>?<br><4
			Doctor call on the next day<br>→ yes/no	Regression investigating the influence of positive mood on the likelihood of having a doctor call on the next day (reference: no call) while controlling for level of pain.	M	NS	
			Doctor call 2d later<br>→ yes/no	Regression investigating the influence of positive mood on the likelihood of having a doctor call 2d later (reference: no call) while controlling for level of pain.	M	β=-.06<br>NS	
Positive mood x emergency HCU							
Gil<br>(2004) [23]	41<br>Sickle cell disease	Daily Mood Scale – Positive mood subscale (baseline)	ER visit on the same day<br>→ yes/no	Regression investigating the influence of positive mood on the likelihood of having an ER visit on the same day (reference: no visit) while controlling for level of pain.	M	β=-.22<br>**p<.001**	<u>Multivariate</u><br>?<br><4
			ER visit on the next day<br>→ yes/no	Regression investigating the influence of positive mood on the likelihood of having an ER visit on the next day (reference:	M	β=-.12<br>**p<.001**	

				no visit) while controlling for level of pain.			
			ER visit 2d later → yes/no	Regression investigating the influence of positive mood on the likelihood of having an ER visit 2d later (reference: no visit) while controlling for level of pain.	M	β=-.08 **p<.05**	
Positive mood x hospitalizations							
Gil (2004) [23]	41 Sickle cell disease	Daily Mood Scale – Positive mood subscale (baseline)	Hospitalization on the same day → yes/no	Regression investigating the influence of positive mood on the likelihood of being hospitalized on the same day (reference: no hospitalization) while controlling for level of pain.	M	β=-.11 **p<.001**	<u>Multivariate</u> ? <4
			Hospitalization on the next day → yes/no	Regression investigating the influence of positive mood on the likelihood of being hospitalized on the next day (reference: no hospitalization) while controlling for level of pain.	M	β=-.08 **p<.01**	
			Hospitalization 2d later → yes/no	Regression investigating the influence of positive mood on the likelihood of being hospitalized 2d later (reference: no hospitalization) while controlling for level of pain.	M	NS	
Psychological flexibility x pain medication use							
McCracken (2007) [53]	260 Chronic pain	Brief Pain Coping Inventory-2 – Psychological flexibility subscale	Number of different pain medications used	Correlation	U	r=-.20 **p<.01**	<u>Univariate</u> ? <4
				Regression investigating the influence of level of psychological flexibility on the number of different pain medications used while also accounting for pain intensity (S) and pain management strategies (NS).	M	β=-.18 **p<.01**	<u>Multivariate</u> ? <4
			Amount of strong opioids used	Correlation	U	r=-.18 **p<.01**	
Psychological flexibility x consultations							
McCracken	260			Correlation	U	r=-.20	<u>Univariate</u>

(2007) [53]	Chronic pain	Brief Pain Coping Inventory-2 – Psychological flexibility subscale	Number of pain-related visits w/ GP, specialists and ER			**p<.01**	?<br><4
				Regression investigating the influence of level of psychological distress on the number of visits while also accounting for pain intensity (S) and pain management strategies (NS).	M	β=-.16<br>**p<.05**	Multivariate<br>?<br><4
Self-compassion x pain medication use							
Elander (2014) [19]	112<br>General population w/ pain	Self-compassion scale	Frequency of prescription pain medication use	Correlation	U	r=.05<br>NS	Univariate<br>?<br><4
			Frequency of OTC pain medication use	Correlation	U	r=-.02<br>NS	
Self-efficacy beliefs x pain medication use							
Elander (2014) [19]	112<br>General population w/ pain	Pain Self-Efficacy Scale	Frequency of prescription pain medication use	Correlation	U	r=-.34<br>**p<.001**	Univariate<br>?<br><4
			Frequency of OTC pain medication use	Correlation	U	r=.02<br>NS	
Nielsen (2015) [60]	1,220<br>Chronic non-cancer pain	Pain Self-Efficacy Scale	4 categories of BZD use: no use; past use; current less than daily use; current daily use	Comparison of level of self-efficacy between patients from the different BZD use groups (reference: no use) while controlling for pain severity.	M	Past: OR: .98; 95%CI: .97-.99<br><Daily: OR: .97; 95%CI: .96-.99<br>Daily: OR: .96; 95%CI: .94-.97<br>**p<.01**	Multivariate<br>?<br><4
Wideman (2011) [83]	202<br>Musculoskeletal neck/back injury undergoing a 7w PT intervention	Pain Self-Efficacy Scale (assessed after PT intervention)	Use of OTC NSAID's, opioids, prescription anti-inflammatory drugs or psychotropic drugs<br>→ yes/no for each, summed into 0-4 score for use of different pain medications<br>(assessed 1y after baseline)	Correlation	U	r=-.412<br>**p<.01**	
				Regression investigating the influence of level of pain self-efficacy on the amount of different pain medications used while controlling for sex (S), pain duration (NS), pre-treatment opioid use (S) and post-treatment pain intensity (S), pain catastrophizing (NS), kinesiophobia (NS) and depressive symptoms (NS).	M	β=-.198<br>**p<.05**	
Self-efficacy beliefs x consultations							
Demmelmaier (2010) [15]	42<br>First-episode back pain	Functional Self-Efficacy Scale (baseline)	Number of consultations w/ healthcare providers at follow-up	Correlation	U	NS	Univariate<br>?<br>3/8 – 38%

		Self-Efficacy Scale for Exercise (baseline)	Number of consultations w/ healthcare providers at follow-up	Correlation	U	NS	negative associations<br><br><u>Multivariate</u><br>00<br>1/5 – 20% negative associations
	271<br>Chronic back pain	Functional Self-Efficacy Scale (baseline)	Number of consultations w/ healthcare providers at follow-up	Correlation	U	NS	
		Self-Efficacy Scale for Exercise (baseline)	Number of consultations w/ healthcare providers at follow-up	Correlation	U	NS	
Lozier (2018) [45]	517<br>Chronic pain	Pain Self-Efficacy Scale	Engagement in clinician-directed non-pharmacological treatments<br>→ no/low/moderate/high engagement	Comparison of level of pain self-efficacy between engagement groups of clinician-directed non-pharmacological treatments.	U	High engagement: 30.9<br>Moderate engagement: 35.9<br>Low engagement: 35.9<br>No engagement: 36.1<br>p=.15	
				Regression investigating the influence of the level of pain self-efficacy on the level of engagement in clinician directed non-pharmacological treatments while also accounting for site (NS), age (S), gender (NS), opioid dose (NS), ethnicity (NS), education (NS), pain disability (S) and depressive symptoms (NS). (Resulting in an aOR presenting the chance of being in a higher engagement category.)	M	OR: 1.00; 95%CI: .99-1.02<br>NS	
Mann (2017) [48]	702<br>Chronic pain	Pain Self-Efficacy Scale<br>→ High vs low levels of self-efficacy	Number of visits w/ GP, specialist or walk-in clinic<br>→ high vs low clinic use	Relative risk analysis investigating the influence of showing low self-efficacy (reference: high self-efficacy) on the likelihood of having high clinic use (reference: low use).	U	RR: 2.99<br>**95%CI : 1.91-4.68**	
				Regression investigating the influence of showing low self-efficacy (reference: high self-efficacy) on the likelihood of having high clinic use (reference: low use) while also accounting for depressive symptoms,	M	aOR: 2.60; 95%CI: 1.50-4.51<br>**p<.01**	

				neuropathic mechanisms, pain timing, pain intensity, diagnosis of back problems, diagnosis of probable nerve damage, use of prescription medication, use of invasive therapy (all above NS – omitted from final model), number of pain locations (S) and presence of comorbidities (S).		
Osborne (2007) [61]	452<br>Chronic osteoarthritis following a self-management course	Stanford scale<br>→ Positive change in self-efficacy after treatment vs negative or no change	Number of doctor visits<br>→ >median vs less (post-treatment)	Regression investigating the influence of having an above >median number of post-treatment doctor visits on the likelihood of having a positive change in self-efficacy scores (reference: negative/no change), while controlling for age, sex, education level, course attendance, baseline level of self-efficacy and baseline number of doctor visits.	M	OR: 1.01; 95%CI: .97-1.05<br>NS
			Number of PT visits<br>→ >median vs less (post-treatment)	Regression investigating the influence of having an above >median number of post-treatment PT visits on the likelihood of having a positive change in self-efficacy scores (reference: negative/no change), while controlling for age, sex, education level, course attendance, baseline level of self-efficacy and baseline number of PT visits.	M	OR: 1.02; 95%CI: .98-1.07<br>NS
Von Korff (2007)[5] [81]	2,010<br>Back pain, TMD pain and headache	Readiness for self-management scale	Number of ambulatory healthcare visits<br>→ high vs low frequency users	Comparison of readiness for self-management scale score between high vs low frequency healthcare users.	U	Low frequency: 2.57 ± .72<br>High frequency: 2.34 ± .65<br>**p<.0001**
Wideman (2011) [83]	202<br>Musculoskeletal neck/back injury	Pain Self-Efficacy Scale (assessed after PT intervention)	Use of PT, psychology, massage therapy and other medical services	Correlation	U	r=-.236<br>**p<.01**
				Regression investigating the influence of level of pain self-	M	β=.105<br>NS

	undergoing a 7w PT intervention		→ yes/no for each, summed into 0-4 score for use of different healthcare services<br>(assessed 1y after baseline)	efficacy on the amount of different healthcare services used while controlling for pre-treatment opioid use (S) and post-treatment pain intensity (S), pain catastrophizing (NS), kinesiophobia (NS) and pain self-efficacy (NS).			
Self-efficacy beliefs x emergency HCU							
Cronin (2018)[5] [11]	67<br>Sickle cell disease	Sickle Cell Self-Efficacy Scale	Number of unscheduled ER and hospital visits	Regression investigating the influence of level of self-efficacy on the amount of emergency HCU while also accounting for age (NS), sex (S), SCD phenotypes (NS), disease-modifying therapy (NS) and Patient Activation Measure (S).	M	IRR=.947; 95%CI: .901-.996<br>**p=.038**	<u>Univariate</u><br>?<br><4<br><br><u>Multivariate</u><br>?<br><4
Mann (2017) [48]	702<br>Chronic pain	Pain Self-Efficacy Scale<br>→ High vs low levels of self-efficacy	Number of ER visits<br>→ high vs low ER use	Relative risk analysis investigating the influence of showing low self-efficacy (reference: high self-efficacy) on the likelihood of having high ER use (reference: low use).	U	RR: 1.85<br>**95%CI : 1.32-2.59**	
				Regression investigating the influence of showing low self-efficacy (reference: high self-efficacy) on the likelihood of having high ER use (reference: low use) while also accounting for depressive symptoms, marital status, diagnosis of other pain condition, pain timing, neuropathic mechanisms, diagnosis of probable nerve damage, diagnosis of arthritis, use of prescription medication, use of chiropractic and/or massage therapy (all above NS – omitted from final model), presence of comorbidities (S) and	M	aOR: 2.01; 95%CI: 1.28-3.15<br>**p<.01**	

				use of other therapy or intervention (S).			
Self-efficacy beliefs x hospitalizations							
Osborne (2007) [61]	452<br>Chronic osteoarthritis<br>Following self-management course	Stanford scale<br>→ Positive change in self-efficacy after treatment vs negative or no change	Number of hospital admissions<br>→ >median vs less (post-treatment)	Regression investigating the influence of having an above >median number of post-treatment hospital admissions on the likelihood of having a positive change in self-efficacy scores (reference: negative/no change), while controlling for age, sex, education level, course attendance, baseline level of self-efficacy and baseline number of hospital admissions.	M	OR: 1.21; 95%CI: .79-1.86<br>NS	<u>Multivariate</u><br>?<br><4
			Number of nights in the hospital<br>→ >median vs less (post-treatment)	Regression investigating the influence of having an above >median number of post-treatment length of hospital stay on the likelihood of having a positive change in self-efficacy scores (reference: negative/no change), while controlling for age, sex, education level, course attendance, baseline level of self-efficacy and baseline length of hospital stay.	M	OR: 1.01; 95%CI: .94-1.09<br>NS	
Self-efficacy beliefs x CAM use							
Lozier (2018) [45]	517<br>Chronic pain	Pain Self-Efficacy Scale	Engagement in self-directed non-pharmacological treatments<br>→ no/low/moderate/high engagement	Comparison of level of pain self-efficacy between engagement groups of self-directed non-pharmacological treatments.	U	High engagement: 32.8<br>Moderate engagement: 38.1<br>Low engagement: 34.4<br>No engagement: 36.0<br>p=.11	<u>Univariate</u><br>?<br><4<br><br><u>Multivariate</u><br>?<br><4
				Regression investigating the influence of level of pain self-efficacy on the level of engagement in self-directed non-pharmacological treatments while also accounting for site (NS), age (NS), gender (NS),	M	aOR: 1.01; 95%CI: 1.00-1.02<br>NS	

				opioid dose (NS), ethnicity (NS), education (S), pain disability (S) and depressive symptoms (NS). (Resulting in an aOR presenting the chance of being in a higher engagement category.)			
Osborne (2007) [61]	452<br>Chronic osteoarthritis<br>Following self-management course	Stanford scale<br>→ Positive change in self-efficacy after treatment vs negative or no change	Number of alternative therapist visits<br>→ >median vs less (post-treatment)	Regression investigating the influence of having an above >median number of post-treatment alternative therapist visits on the likelihood of having a positive change in self-efficacy scores (reference: negative/no change), while controlling for age, sex, education level, course attendance, baseline level of self-efficacy and baseline number of alternative therapist visits.	M	OR: .97; 95%CI: .91-1.04<br>NS	
Self-efficacy beliefs x HCU in general							
Cronan (2002)[5] [10]	600<br>Fibromyalgia	Arthritis self-efficacy scale (baseline)	Total HCU (number of contacts, tests and medication) during past year	Correlation	U	r=-.12<br>**p<.01**	<u>Univariate</u><br>?<br><4
			Total HCU (number of contacts, tests and medication) 1y after treatment initiation	Correlation	U	r=-.08<br>**p<.05**	<u>Multivariate</u><br>?<br><4
				Regression investigating the influence of baseline level of self-efficacy on the amount of HCU at follow-up while also accounting for baseline health status (NS), ethnicity (S), comorbidity (S), education (NS), income (NS), age (S), employment (NS), social support (NS), baseline HCU (S), coping (NS), depressive symptoms (NS) and helplessness (NS).	M	b=-.00; 95%CI: -.01-.00<br>p=.6	
**OTHER CEF CLUSTERS**							
Health attributions x pain medication use							

Primavera (1994)5 [65]	30 Headache	Health Attribution Test – Internal subscale	Pain medication use	Correlation	U	r=-.0329 p=.43	Univariate ? <4
		Health Attribution Test – Powerful others subscale	Pain medication use	Correlation	U	r=.2471 p=.09	
		Health Attribution Test – Chance subscale	Pain medication use	Correlation	U	r=.1801 p=.17	
Health attributions x hospitalizations							
Primavera (1994)5 [65]	30 Headache	Health Attribution Test – Internal subscale	Length of stay	Correlation	U	r=-.0917 p=.31	Univariate ? <4
		Health Attribution Test – Powerful others subscale	Length of stay	Correlation	U	r=.0959 p=.30	
		Health Attribution Test – Chance subscale	Length of stay	Correlation	U	r=.2308 p=.10	
Locus of control x consultations							
Kuijper (2014) [40]	330 Arthralgia w/o synovitis	Multidimensional Health Locus of Control Questionnaire – Internal subscale (baseline)	Number of visits w/ healthcare providers for joint symptoms 6m later	Regression investigating the influence of baseline internal locus of control score on the number of healthcare visits 6m later while also accounting for duration of symptoms, locus of control (chance), coping, ethnicity, education, household composition, employment, BMI, fatigue, diagnosis, comorbidities, SF-36 mental component score (all above NS – omitted from final analysis), month (S – fixed factor), age (NS – fixed factor),	M	NS (omitted from final model)	Multivariate 00 1/6 17%

			sex (NS -fixed factor), pain (S), SF-36 physical component (S) and locus of control (external (S)).		
	Multidimensional Health Locus of Control Questionnaire – External subscale (baseline)	Number of visits w/ healthcare providers for joint symptoms 6m later	Regression investigating the influence of baseline external locus of control score on the number of healthcare visits 6m later while also accounting for month (S – fixed factor), age (NS – fixed factor), sex (NS - fixed factor), pain (S) and SF-36 physical component (S).	M	IRR: 1.036; 95%CI: 1.006-1.066 **p=.018**
	Multidimensional Health Locus of Control Questionnaire – Chance subscale (baseline)	Number of visits w/ healthcare providers for joint symptoms 6m later	Regression investigating the influence of baseline chance locus of control score on the number of healthcare visits 6m later while also accounting for duration of symptoms, locus of control (internal), coping, ethnicity, education, household composition, employment, BMI, fatigue, diagnosis, comorbidities, SF-36 mental component score (all above NS – omitted from final analysis), month (S – fixed factor), age (NS – fixed factor), sex (NS -fixed factor), pain (S), SF-36 physical component (S) and locus of control (external (S)).	M	NS (omitted from final model)
244 Rheumatoid arthritis	Multidimensional Health Locus of Control Questionnaire – Internal subscale (baseline)	Number of visits w/ healthcare providers for joint symptoms 6m later	Regression investigating the influence of baseline internal locus of control score on the number of healthcare visits 6m later while also accounting for ethnicity, education, household composition, employment, BMI, fatigue, diagnosis, comorbidities, coping, locus of control (external), pain, SF-36 mental component score (all above NS – omitted from final analysis),	M	NS (omitted from final model)

				month (S – fixed factor), age (NS – fixed factor), sex (NS -fixed factor), duration of symptoms (S), SF-36 physical component (S) and locus of control (chance (S)).		
		Multidimensional Health Locus of Control Questionnaire – External subscale (baseline)	Number of visits w/ healthcare providers for joint symptoms 6m later	Regression investigating the influence of baseline external locus of control score on the number of healthcare visits 6m later while also accounting for ethnicity, education, household composition, employment, BMI, fatigue, diagnosis, comorbidities, coping, locus of control (internal), pain, SF-36 mental component score (all above NS – omitted from final analysis), month (S – fixed factor), age (NS – fixed factor), sex (NS -fixed factor), duration of symptoms (S), SF-36 physical component (S) and locus of control (chance (S)).	M	NS (omitted from final model)
		Multidimensional Health Locus of Control Questionnaire – Chance subscale (baseline)	Number of visits w/ healthcare providers for joint symptoms 6m later	Regression investigating the influence of baseline chance locus of control score on the number of healthcare visits 6m later while also accounting for month (S – fixed factor), age (NS – fixed factor), sex (NS -fixed factor), duration of symptoms (S) and SF-36 physical component (S).	M	IRR: .972; 95%CI: .953-.991 **p=.004**

[1]If outcomes for CEF and HCU were measured at the same moment, the moment of assessment was not mentioned. If there was a difference in moment of assessment, than this was mentioned between brackets under the respective outcome.
[2]Multivariate analyses: If the independent variable of interest (CEF/HCU outcome) was part of the final model, then the remaining independent variables in the final model were mentioned (for information on potential other considered independent variables, see Table A2 with study characteristics), including their significance in the model. If the independent variable of interest (CEF/HCU outcome) was omitted from the final model, then all independent variables considered for the multivariate model were reported including information on whether they were retained in the model, and if so, their significance in the model.
[3]Effect sizes were reported if available, otherwise only the p-value and, if available, the direction of the relationship was reported (+/-).
[4]Level of association was rated as follows:

+/-: ≥ 60% of the analyses reported a +/- association
?: 34-59% of the analyses reported a +/- association, or fewer than 4 studies investigated the association (<4)
0: ≤ 33% of the analyses reported an association
++/--/00: If after exclusion of high risk of bias studies the association (+/-) or absence of association (0) was still supported by ≥ 60% of the analyses the summary score was up/downgraded to ++/--/00.
[5]Study rated as 'high risk of bias'
Abbreviations: n: sample size; CEF: cognitive and emotional factors; HCU: healthcare utilization; U: univariate; M: multivariate; m: month(s); β: regression coefficient; 95%CI: 95% confidence interval; NS: non-significant; S: significant; GP: general practitioner; p: p-value; r: Pearson's correlation coefficient; OTC: over-the-counter; BZD: Benzodiazepine; w/:with; w/o: without; OR: odd's ratio; ER: emergency room; PTSD: post-traumatic stress disorder; RD: relative difference; CAM: complementary and alternative medicine; SEM: structural equation modelling; NSAID: non-steroidal anti-inflammatory drugs; CBT: cognitive behavioral therapy; y: year(s); PT: physical therapist/physiotherapist; SE: standard error; IRR: incidence rate ratio; OT: occupational therapist; MD: medical doctor; aOR: adjusted odd's ratio; RR: relative risk; BMI: body mass index; VAS: visual analogue scale; d: day(s); TMD: temporo-mandibular disorder; w: week(s)

Table S5: Comprehensive overview of the results of analyses investigating associations between CEF and type of HCU

Author (year)	Sample<br>n<br>Type of population	Outcome CEF[1]	Outcome HCU[1]	Investigated association[2]	U/M	Findings[3]	Level of association[4]
**MALADAPTIVE CEF CLUSTERS**							
Anger symptoms x prescription pain medication use							
Asmundson (2001)[5] [2]	108<br>Chronic headache	State-Trait Anger Expression Inventory – Trait form	Prescription headache medication<br>→ yes/no	Correlation	U	r=-.06<br>NS	<u>Univariate</u><br>?<br><4<br><br><u>Multivariate</u><br>?<br><4
				Regression investigating the influence of level of anger on the likelihood of using prescription pain medication (reference: no use) while also accounting for cognitive anxiety, fearful appraisals of pain, trait anxiety, depressive symptoms, fear of social concerns, mental incapacitation, distressing nature of headache, degree of lifestyle change (all above: NS – omitted from final model), physiological anxiety (S), fear of physical catastrophe (S) and pain severity (S).	M	NS (omitted from final model)	
Anger symptoms x OTC pain medication use							
Asmundson (2001)[5] [2]	108<br>Chronic headache	State-Trait Anger Expression Inventory – Trait form	OTC headache medication<br>→ yes/no	Correlation	U	r=.08<br>NS	<u>Univariate</u><br>?<br><4<br><br><u>Multivariate</u><br>?<br><4
				Regression investigating the influence of level of anger on the likelihood of using OTC pain medication (reference: no use) while also accounting for physiological anxiety, fearful appraisals of pain, trait anxiety, depressive symptoms, fear of social concerns, mental incapacitation, distressing nature of headache, degree of lifestyle change, fear of physical	M	NS (omitted from final model)	

				catastrophe (all above: NS – omitted from final model), cognitive anxiety (S) and pain severity (NS – fixed factor).			
General anxiety symptoms x prescription pain medication use							
Asmundson (2001)[5] [2]	108<br>Chronic headache	State-Trait Anxiety Inventory – Trait form (STAI-T)	Prescription headache medication<br>→ yes/no	Correlation	U	r=-.01<br>NS	<u>Univariate</u><br>?<br><4<br><br><u>Multivariate</u><br>?<br><4
				Regression investigating the influence of STAI-T score on the likelihood of using prescription pain medication (reference: no use) while also accounting for cognitive anxiety, fearful appraisals of pain, depressive symptoms, trait anger, fear of social concerns, mental incapacitation, distressing nature of headache, degree of lifestyle change (all above: NS – omitted from final model), physiological anxiety (S), fear of physical catastrophe (S) and pain severity (S).	M	NS (omitted from final model)	
Pierce (2019)[5] [64]	1,785<br>Chronic pain	Hospital Anxiety and Depression Scale – Anxiety subscale	Benzodiazepine use<br>→ yes/no	To compare level of general anxiety symptoms between users and non-users of benzodiazepines.	U	Non-users: 8.51 ± 4.30<br>Users: 11.05 ± 4.85<br>**p<.001**	
				Regression investigating the influence of level of general anxiety symptoms on the likelihood of using benzodiazepines (reference: no use) while also accounting for age (NS), sex (NS), pain severity (NS), pain interference (NS), fibromyalgia survey score (S), depressive symptoms (NS), lifetime abuse (NS) and interactions between anxiety and child (NS), adult (NS) and cumulative (S) abuse.	M	OR: 1.07; 95%CI: 1.027-1.124<br>**p=.002**	

General anxiety symptoms x OTC pain medication use							
Asmundson (2001)[5] [2]	108<br>Chronic headache	State-Trait Anxiety Inventory – Trait form (STAI-T)	OTC headache medication<br>→ yes/no	Correlation	U	r=.04<br>NS	<u>Univariate</u><br>?<br><4<br><br><u>Multivariate</u><br>?<br><4
				Regression investigating the influence of STAI-T score on the likelihood of using OTC pain medication use (reference: no use) while also accounting for physiological anxiety, fearful appraisals of pain, depressive symptoms, trait anger, fear of social concerns, mental incapacitation, distressing nature of headache, degree of lifestyle change, fear of physical catastrophe (all above: NS – omitted from final model), cognitive anxiety (S) and pain severity (NS – fixed factor).	M	NS (omitted from final model)	
General anxiety symptoms x opioid use							
Buse (2012) [5]	5,796<br>Migraine	Generalized Anxiety Disorder-7<br>→ General anxiety symptoms yes/no	Opioid use<br>→ non-users (reference) vs previous users	Regression investigating the influence of presence of anxiety symptoms (reference: no symptoms) on the likelihood of previous opioid use (reference: no use).	U	OR: 1.63; 95%CI: 1.27-2.08<br>**p<.001**	<u>Univariate</u><br>+<br>4/6 – 67%<br><br><u>Multivariate</u><br>?<br><4
			Opioid use<br>→ non-users (reference) vs current non-dependent users	Regression investigating the influence of presence of anxiety symptoms (reference: no symptoms) on the likelihood of being a current non-dependent opioid user (reference: no use).	U	OR: 2.22; 95%CI: 1.76-2.80<br>**p<.001**	
			Opioid use<br>→ non-users (reference) vs current dependent users	Regression investigating the influence of presence of anxiety symptoms (reference: no symptoms) on the likelihood of being a current dependent opioid user (reference: no use).	U	OR: 4.32; 95%CI: 2.95-6.33<br>**p<.001**	
Gebauer	327	Self-designed question	1-50mg/d MED opioid use vs no use	Regression investigating the influence of presence of anxiety	M	OR: .69; 95%CI: .33-1.43<br>NS	

(2019)[5] [22]	Chronic low back pain	→ anxiety symptoms yes/no		symptoms (reference: no symptoms) on the likelihood of using 1-50mg/d MED (reference: no opioid use) while also accounting for moment of assessment (NS), collecting disability (NS), age (NS), race (NS), sex (NS), education (NS), pain severity (NS), pain duration (NS), health-related quality of life (pain interference (NS), physical functioning (NS), role physical (NS) and general health (S)), comorbidities (NS), overweight/obesity (NS), depressive symptoms (NS), other treatments (NS), having a written pain contract (S) and continuity of care (S).		
			>50mg/d MED opioid use vs no use	Regression investigating the influence of presence of anxiety symptoms (reference: no symptoms) on the likelihood of using >50mg/d MED (reference: no opioid use) while also accounting for moment of assessment (S), collecting disability (S), age (NS), race (NS), sex (NS), education (NS), pain severity (NS), pain duration (NS), health-related quality of life (pain interference (NS), physical functioning (NS), role physical (NS) and general health (NS)), comorbidities (NS), overweight/obesity (NS), depressive symptoms (S), other treatments (NS), having a written pain contract (S) and continuity of care (NS).	M	OR: .84; 95%CI: .23-3.09<br>NS

Harden (1997)[5] [27]	200<br>Chronic pain	State-Trait Anxiety Inventory – Trait form (STAI-T)	Taking daily opioids<br>→ yes/no	To compare STAI-trait score between patients taking daily opioids and those who do not.	U	Daily opioids: 45.5 ± 10.8<br>No opioids: 41.7 ± 13.3<br>p>.1	
Huffman (2017)[5] [31]	1,457<br>Chronic non-cancer pain following an interdisciplinary program	Depression, Anxiety and Stress Scale – Anxiety subscale (baseline or post-discharge)	Chronic opioid use<br>→ no use (reference); low dose; high dose (baseline)	Comparison of baseline anxiety symptoms score between the 3 opioid use groups.	U	No use: 13.36 ± 9.99<br>Low dose: 13.10 ± 9.47<br>High dose: 14.74 ± 9.89<br>**p=.03**	
				Linear mixed model investigating the influence of baseline level of opioid use (reference: no use) on the level of anxiety symptoms post-discharge while controlling for marital status (S), age (NS), gender (NS) and baseline score for anxiety symptoms (S).	M	Low dose : β=.49; p=.94<br>High dose: β=-.07 ; p=.93	
Jensen (2006)[5] [33]	160<br>Chronic non-cancer pain	Hospital Anxiety and Depression Scale – Anxiety subscale<br>→ anxiety symptoms yes/no	Opioid use<br>→ yes/no	Chi²	U	NS	
General anxiety symptoms x primary care consultations							
Jordan (2006)[5] [34]	1,797<br>Knee pain	Hospital Anxiety and Depression Scale – Anxiety subscale<br>→ Most vs less symptoms (baseline)	Future primary care visit for knee pain<br>→ yes/no	Regression investigating the influence of showing most anxiety symptoms (reference: less symptoms) on the likelihood of having a future primary care consultation for knee pain (reference: no consultation).	U	OR: 1.17; 95%CI: .89-1.54<br>NS	<u>Univariate</u><br>?<br><4<br><br><u>Multivariate</u><br>?<br><4
				Regression investigating the influence of showing most anxiety symptoms (reference: less symptoms) on the likelihood of having a future primary care consultation for knee pain (reference: no consultation) while also accounting for BMI (S), depressive symptoms (NS), widespread pain (NS), favorable	M	OR: .98; 95%CI: .71-1.35<br>NS	

				evaluation (NS) and frequency of consulting (S).			
General anxiety symptoms x secondary care consultations							
Boyer (2009) [4]	315 Fibromyalgia	Hospital Anxiety and Depression Scale – Anxiety subscale	Attending rheumatology setting vs primary care	Comparison of anxiety symptoms score between users of a rheumatology setting and primary care users.	U	Rheumatology: 57.93 ± 23.28<br>Primary care: 58.68 ± 20.83<br>NS	<u>Univariate</u><br>?<br><4
Vervoort (2019) [77]	199 Fibromyalgia	Hospital Anxiety and Depression Scale – Anxiety subscale (Baseline)	Recurrent secondary care user at 18m follow-up<br>→ yes/no	Regression investigating the influence of baseline level of anxiety symptoms on the likelihood of recurrent secondary care use (reference: no secondary care use).	U	OR: 1.06; 95%CI: .99-1.14<br>p=.09	<u>Multivariate</u><br>?<br><4
				Regression investigating the influence of baseline level of anxiety symptoms on the likelihood of recurrent secondary care use (reference: no secondary care use) while also accounting for severity of fibromyalgia, depressive symptoms, illness perceptions (consequences and personal control), active pain coping, helplessness (all above: NS – omitted from final model) and comorbidity (S).	M	NS (omitted from final model)	
General anxiety symptoms x emergency HCU							
Musey (2018)[5] [56]	163 Chest pain	Hospital Anxiety and Depression Scale – Anxiety subscale<br>→ High vs low anxiety symptoms	ER visit<br>→ yes/no	Chi²	U	High anxiety: 52% had ≥1 ER visit<br>Low anxiety: 79% had ≥1 ER visit<br>**Significant**	<u>Univariate</u><br>?<br><4
General anxiety symptoms x CAM use							
van Tilburg (2008) [76]	1,012 Functional bowel disorder	Brief Symptom Inventory – Anxiety subscale	CAM use<br>→ yes/no	Comparison of level of anxiety symptoms between CAM users and non-users.	U	Users: 4.5 ± 5.0<br>Non-users: 2.9 ± 3.7<br>**p<.001**	<u>Univariate</u><br>?<br><4
				Regression investigating the influence of level of anxiety	M	β=.09<br>**p=.008**	<u>Multivariate</u>

				symptoms on the likelihood of using CAM services (reference: no CAM use) while also accounting for age (NS), sex (S), education (S), IBS severity (NS), suffering from distention (NS), depressive symptoms (NS), somatization (NS), quality of life (NS), non-prescription costs (NS) and satisfactory relief of bowel symptoms (NS).			?<br><4
Symptom-related anxiety symptoms x prescription pain medication use							
Asmundson (2001)[5] [2]	108<br>Chronic headache	Pain Anxiety Symptom Scale – Pain-specific cognitive anxiety subscale	Prescription headache medication<br>→ yes/no	Correlation	U	r=.10<br>NS	<u>Univariate</u><br>?<br><4<br><br><u>Multivariate</u><br>?<br><4
				Regression investigating the influence of level of cognitive anxiety on the likelihood of using prescription pain medication (reference: no use) while also accounting for fearful appraisals of pain, depressive symptoms, trait anger, trait anxiety, fear of social concerns, mental incapacitation, distressing nature of headache, degree of lifestyle change (all above: NS – omitted from final model), physiological anxiety (S), fear of physical catastrophe (S) and pain severity (S).	M	NS (omitted from final model)	
		Pain Anxiety Symptom Scale – Pain-specific physiological anxiety subscale	Prescription headache medication<br>→ yes/no	Correlation	U	r=.10<br>NS	
				Regression investigating the influence of level of physiological anxiety on the likelihood of using prescription pain medication (reference: no use) while also accounting for depressive symptoms, cognitive anxiety, fearful appraisals of pain, trait anxiety, trait anger, fear of social	M	β=.241<br>**p=.018**	

				concerns, mental incapacitation, distressing nature of headache, degree of lifestyle change (all above: NS – omitted from final model), fear of physical catastrophe (S) and pain severity (S).			
		Pain Anxiety Symptom Scale – Fearful appraisals of pain subscale	Prescription headache medication → yes/no	Correlation	U	r=-.20<br>**p<.05**	
				Regression investigating the influence of level of fearful appraisals of pain on the likelihood of using prescription pain medication (reference: no use) while also accounting for depressive symptoms, cognitive anxiety, trait anxiety, trait anger, fear of social concerns, mental incapacitation, distressing nature of headache, degree of lifestyle change (all above: NS – omitted from final model), physiological anxiety (S), fear of physical catastrophe (S) and pain severity (S).	M	NS (omitted from final model)	
Symptom-related anxiety symptoms x OTC pain medication use							
Asmundson (2001)[5] [2]	108<br>Chronic headache	Pain Anxiety Symptom Scale – Pain-specific cognitive anxiety subscale	OTC headache medication<br>→ yes/no	Correlation	U	r=.19<br>NS	<u>Univariate</u><br>?<br><4<br><br><u>Multivariate</u><br>?<br><4
				Regression investigating the influence of level of cognitive anxiety on the likelihood of using OTC pain medication (reference: no use) while also accounting for physiological anxiety, fearful appraisals of pain, trait anxiety, trait anger, depressive symptoms, fear of social concerns, mental incapacitation, distressing nature of headache, degree of lifestyle change, fear of physical catastrophe (all above:	M	β=.208<br>**p=.04**	

				NS – omitted from final model) and pain severity (NS – fixed factor).			
		Pain Anxiety Symptom Scale – Pain-specific physiological anxiety subscale	OTC headache medication → yes/no	Correlation	U	r=.14<br>NS	
				Regression investigating the influence of level of physiological anxiety on the likelihood of using OTC pain medication (reference: no use) while also accounting for depressive symptoms, fearful appraisals of pain, trait anxiety, trait anger, fear of social concerns, mental incapacitation, distressing nature of headache, degree of lifestyle change, fear of physical catastrophe (all above: NS – omitted from final model), cognitive anxiety (S) and pain severity (NS – fixed factor).	M	NS (omitted from final model)	
		Pain Anxiety Symptom Scale – Fearful appraisals of pain subscale	OTC headache medication → yes/no	Correlation	U	r=.08<br>NS	
				Regression investigating the influence of level of fearful appraisals of pain on the likelihood of using OTC pain medication (reference: no use) while also accounting for depressive symptoms, physiological anxiety, trait anxiety, trait anger, fear of social concerns, mental incapacitation, distressing nature of headache, degree of lifestyle change, fear of physical catastrophe (all above: NS – omitted from final model), cognitive anxiety (S) and pain severity (NS – fixed factor).	M	NS (omitted from final model)	
Symptom-related anxiety x consultations							
Williams	98	Fear that symptoms might	Doctor's visit for abdominal symptoms	Regression investigating the influence of fear that symptoms	U	OR: 1.2; 95%CI: .54-2.6<br>NS	<u>Univariate</u><br>?

(2006) [85]	Males w/ irritable bowel syndrome	be related to cancer<br>→ yes/no	→ yes/no	might be related to cancer (reference: no fear) on the likelihood of seeking care (reference: not seeking care).			<4
	239<br>Females w/ irritable bowel syndrome	Fear that symptoms might be related to cancer<br>→ yes/no	Doctor's visit for abdominal symptoms<br>→ yes/no	Regression investigating the influence of fear that symptoms might be related to cancer (reference: no fear) on the likelihood of seeking care (reference: not seeking care).	U	OR: 1.8; 95%CI: 1.1-3.1<br>**p<.05**	
Symptom-related anxiety symptoms x primary care consultations							
Howell<br>(1999) [30]	614<br>Dyspepsia	Self-designed questionnaire<br>→ none; a little; moderate; considerable; extreme pain-related anxiety	GP visits for dyspepsia symptoms<br>→ yes vs no	Chi²	U	<u>% having a visit</u><br>None: 63.2%<br>A little: 79.6%<br>Moderate: 81.9%<br>Considerable: 85.5%<br>Extreme: 91.7%<br>**p=.001**	<u>Univariate</u><br>++<br>3/4 - 75%<br><br><u>Multivariate</u><br>?<br><4
				Logistic regression investigating the influence of level of pain-related anxiety (reference: none) on the likelihood of having had GP visits (reference: no visits) in the past while also accounting for gender, alcohol consumption, marital status, ethnicity, smoking status, NSAID use, age, pain duration, pain severity, fear of serious illness, fear that pain might be cancer (all above: NS – omitted from final model), neuroticism (S) and pain frequency (S).	M	<u>ORs (95%CI); p</u><br>A little: 2.08 (1.17-3.70); **p=.01**<br>Moderate: 2.28 (1.27-4.09); **p=.01**<br>Considerable: 2.70 (1.38-5.27); **p=.004**<br>Extreme: 4.66 (1.31-16.60); **p=.02**	
		Fear of serious illness<br>→ yes/no	GP visits for dyspepsia symptoms<br>→ yes/no	Chi²	U	<u>% having a visit</u><br>Fear: 83.9%<br>No fear: 71.6%<br>**p=.001**	
				Logistic regression investigating the influence of presence of fear of serious illness (reference: no fear) on the likelihood of having	M	NS (omitted from final model)	

				had GP visits (reference: no visits) in the past while also accounting for gender, alcohol consumption, marital status, ethnicity, smoking status, NSAID use, age, pain duration, pain severity, fear that pain might be cancer (all above: NS – omitted from final model), symptom-related anxiety (S), neuroticism (S) and pain frequency (S).			
		Fear that pain might be cancer<br>→ yes/no	GP visits for dyspepsia symptoms<br>→ yes/no	Chi²	U	<u>% having a visit</u><br>Fear: 85.2%<br>No fear: 76.0%<br>**p=.001**	
				Logistic regression investigating the influence of presence of fear that pain might be cancer (reference: no fear) on the likelihood of having had GP visits (reference: no visits) in the past while also accounting for gender, alcohol consumption, marital status, ethnicity, smoking status, NSAID use, age, pain duration, pain severity, fear of serious illness (all above: NS – omitted from final model), symptom-related anxiety (S), neuroticism (S) and pain frequency (S).	M	NS (omitted from final model)	
Macfarlane (1999) [46]	252<br>Chronic widespread pain	Illness Attitutde Scale – Disease phobia subscale	GP consultation for pain<br>→ yes/no	Comparison of the level of disease phobia between consulters and non-consulters.	U	NS	
Symptom-related anxiety symptoms x invasive procedures							
Lozano-Calderon (2008) [44]	72<br>Trapezio-metacarpal joint arthrosis	Pain Anxiety Symptoms Scale	Opting for surgery<br>→ yes/no	Comparison of level of pain anxiety symptoms between patients opting for surgery and those who do not.	U	Surgery: 47 ± 24.5<br>No surgery: 46 ± 34<br>p=.87	<u>Univariate</u><br>?<br><4
Catastrophizing x pain medication use							

de Boer (2012) [14]	150 Pain center patients	Pain Catastrophizing Scale (PCS)	Pain medication use → yes/no	Regression investigating the influence of PCS score on the likelihood of using pain medication (reference: no use) while also accounting for age (NS), sex (NS) and pain intensity (NS).	M	OR: .94; 95%CI: .97-1.03<br>NS	<u>Multivariate</u><br>++<br>4/6 – 67%
	137 Community sample w/ pain	Pain Catastrophizing Scale (PCS)	Pain medication use → yes/no	Regression investigating the influence of PCS score on the likelihood of using pain medication (reference: no use) while also accounting for age (S), sex (S) and pain intensity (S).	M	OR: 1.04; 95%CI: 1.00-1.09<br>**p<.05**	
Valdes (2015) [75]	852 Total knee or hip replacement	Pain Catastrophizing Scale → High/low catastrophizing	Taking NSAID's → yes/no	Regression investigating the influence of showing high catastrophizing (reference: low catastrophizing) on the likelihood of taking NSAID's (reference: no NSAID use) while accounting for age, sex, BMI, back pain, WOMAC pain, body pain and illness behavior.	M	OR: 1.27; 95%CI: .67-2.41<br>p=.46	
			Not using pain medication → yes/no	Regression investigating the influence of showing high catastrophizing (reference: low catastrophizing) on the likelihood of not taking pain medication (reference: taking pain medication) while accounting for age, sex, BMI, back pain, WOMAC pain, body pain and illness behavior.	M	OR: .52; 95%CI: .36-.76<br>**p=.0007**	
Wijnhoven (2007) [84]	1,082 Men w/ musculoskeletal pain	Pain Catastrophizing Scale → Low (reference), medium and high catastrophizing	Pain medication use → yes/no	Regression investigating the influence of level of pain catastrophizing (reference: low catastrophizing) on the likelihood of using pain medication (reference: no use) in men while also accounting for age (S), household composition (S) and smoking (NS).	M	Medium catastrophizing:<br>PR: 1.48; 95%CI:.99-2.22; NS<br>High catastrophizing:<br>PR: 2.55; 95%CI: 1.78-3.65; **p<.05**	

	1,435<br>Women w/ musculoskeletal pain	Pain Catastrophizing Scale<br>→ Low (reference), medium and high catastrophizing	Pain medication use<br>→ yes/no	Regression investigating the influence of level of pain catastrophizing (reference: low catastrophizing) on the likelihood of using pain medication (reference: no use) in women while also accounting for age (S), household composition (NS) and smoking (S).	M	Medium catastrophizing:<br>PR: 1.35; 95%CI: 1.02-1.80; **p<.05**<br>High catastrophizing:<br>PR: 1.91; 95%CI: 1.47-2.48; **p<.05**	
Catastrophizing x prescription pain medication use							
Valdes (2015) [75]	852<br>Total knee or hip arthroplasty	Pain Catastrophizing Scale<br>→ High/low catastrophizing	Taking prescription pain medication (other than opioids or NSAID's)<br>→ yes/no	Regression investigating the influence of showing high catastrophizing (reference: low catastrophizing) on the likelihood of taking prescription pain medications (reference: no use) while accounting for age, sex (S), BMI (S), back pain (S), WOMAC pain (S), body pain (S) and illness behavior (NS).	M	OR: 2.52; 95%CI: 1.61-3.95<br>**p<.0001**	<u>Multivariate</u><br>?<br><4
Catastrophizing x opioid use							
Jensen (2006)[5] [33]	160<br>Chronic non-cancer pain	Coping Strategies Questionnaire (CSQ) – Catastrophizing subscale	Opioid use<br>→ yes/no	Comparison of CSQ catastrophizing scores between patients using and not using opioids.	U	Higher catastrophizing in opioid users<br>**p=.024**	<u>Univariate</u><br>?<br><4<br><br><u>Multivariate</u><br>?<br><4
Kapoor (2014) [37]	64<br>Chronic pain	Pain Catastrophizing Scale	Opioid use<br>→ yes/no	Correlation	U	r=.209<br>NS	
Newman (2018)[5] [59]	290<br>Chronic pain	Pain Catastrophizing Scale	Opioid use<br>→ yes/no	Correlation	U	r=.03<br>NS	
Valdes (2015) [75]	852<br>Total knee/hip arthroplasty	Pain Catastrophizing Scale<br>→ High vs low catastrophizing	Taking opioids<br>→ yes/no	Regression investigating the influence of showing high catastrophizing (reference: low catastrophizing) on the likelihood of taking opioids (reference: no opioid use) while accounting for age, sex, BMI, back pain,	M	OR: 1.66; 95%CI: 1.13-2.43<br>**p=.0094**	

				WOMAC pain, body pain and illness behavior.			
			Taking strong opioids → yes/no	Regression to investigate the influence of showing high catastrophizing (reference: low catastrophizing) on the likelihood of taking strong opioids (reference: no use of strong opioids) while accounting for age, sex, BMI, back pain, WOMAC pain, body pain and illness behavior.	M	OR: 1.97; 95%CI: .62-6.25<br>p<.25	
			Taking weak opioids → yes/no	Regression to investigate the influence of showing high catastrophizing (reference: low catastrophizing) on the likelihood of taking weak opioids (reference: no use of weak opioids) while accounting for age, sex, BMI, back pain, WOMAC pain, body pain and illness behavior.	M	OR: 1.66; 95%CI: 1.14-2.44<br>**p<.009**	
Catastrophizing x consultations							
Jöud (2017) [35]	7,792<br>People w/ pain	Pain Catastrophizing Scale (PCS)<br>→ PCS>17; PCS 10-17; PCS<10 (reference)	Pain-related healthcare consultation<br>→ yes/no	Regression investigating the influence of level of PCS score (reference: PCS<10) on the likelihood of having a pain-related consultation (reference: no consultation) while accounting for age, education, sex (NS), pain spread (NS), pain intensity (S) and pain duration (NS).	M	PR; 95%CI (reference: PCS<10)<br>PCS>17: 1.5; 1.37-1.66<br>PCS 10-17: 1.1; 1.04-1.25<br>**p<.0001**	<u>Multivariate</u><br>?<br><4
Wijnhoven (2007) [84]	1,082<br>Men w/ musculoskeletal pain	Pain Catastrophizing Scale<br>→ Low (reference), medium and high catastrophizing	Contact w/ GP, specialists or PT<br>→ yes/no	Regression investigating the influence of level of pain catastrophizing (reference: low catastrophizing) on the likelihood of having healthcare consultations (reference: no consultations) in men while also accounting for age (S),	M	Medium catastrophizing:<br>PR: 1.09; 95%CI: .91-1.31; NS<br>High catastrophizing:<br>PR: 1.28; 95%CI: 1.09-1.50; **p<.05**	

				educational level (S) and smoking (NS).			
	1,435<br>Women w/ musculoskeletal pain	Pain Catastrophizing Scale<br>→ Low (reference), medium and high catastrophizing	Contact w/ GP, specialists or PT<br>→ yes/no	Regression investigating the influence of level of pain catastrophizing (reference: low catastrophizing) on the likelihood of having healthcare consultations (reference: no consultations) in women while also accounting for age (S), educational level (NS) and smoking (S).	M	Medium catastrophizing:<br>PR: 1.34; 95%CI: 1.14-1.57; **p<.05**<br>High catastrophizing:<br>PR: 1.50; 95%CI: 1.29-1.74; **p<.05**	
Catastrophizing x primary care consultations							
Macfarlane (1999) [46]	252<br>Chronic widespread pain	Illness Attitude Scale – Hypochondrial beliefs subscale	GP consultation for pain<br>→ yes/no	Comparison of the level of hypochondrial beliefs between consulters and non-consulters.	U	NS	<u>Univariate</u><br>?<br><4
Catastrophizing x secondary care consultations							
de Boer (2012) [14]	150<br>Pain center patients	Pain Catastrophizing Scale (PCS)	Specialist consultation<br>→ yes/no	Regression investigating the influence of PCS score on the likelihood of having a specialist consultation (reference: no consultation) while also accounting for age (NS), sex (NS) and pain intensity (NS).	M	OR: 1.03; 95%CI: .99-1.07<br>NS	<u>Univariate</u><br>?<br><4<br><br><u>Multivariate</u><br>?<br><4
	137<br>Community sample w/ pain	Pain Catastrophizing Scale (PCS)	Specialist consultation<br>→ yes/no	Regression investigating the influence of PCS score on the likelihood of having a specialist consultation (reference: no consultation) while also accounting for age (NS), sex (NS) and pain intensity (NS).	M	OR: 1.05; 95%CI: 1.01-1.10<br>**p<.05**	
Elander (2003) [18]	68<br>Haemophilia	Coping Strategies Questionnaire (CSQ)	Comprehensive care users vs other haemophilia center users	Comparison of CSQ scores between patients attending comprehensive haemophilia center and those attending other centers.	U	Comprehensive care: 2.7 ± 1.1<br>Other center: 2.7 ± .73<br>NS	
Catastrophizing x tertiary care consultations							

Fink-Miller (2014)[5] [21]	233<br>Chronic non-cancer pain	Pain Catastrophizing Scale (PCS)	Attending tertiary care (reference: primary care)	Comparison of PCS scores between tertiary and primary care patients.	U	Tertiary care: 21.43<br>Primary care: 12.91<br>**p<.001**	<u>Univariate</u><br>?<br><4<br><br><u>Multivariate</u><br>?<br><4
				Regression investigating whether attending tertiary care (reference: primary care) is significantly influencing PCS scores while adjusting for age.	M	Primary care patients: 8.57-unit lower PCS score compared to tertiary care patient<br>**p<.001**	
Catastrophizing x invasive procedures							
Lozano-Calderon (2008) [44]	72<br>Trapezio-metacarpal joint arthrosis	Pain Catastrophizing Scale	Opting for surgery<br>→ yes/no	Comparison of level of pain catastrophizing between patients opting for surgery and those who do not.	U	Surgery: 21 ± 7.3<br>No surgery: 20 ± 7.5<br>p=.61	<u>Univariate</u><br>?<br><4
Depressive symptoms x pain medication use							
Vina (2019) [79]	360<br>Knee osteoarthritis	Patient Health Questionnaire-8<br>→ moderate/severe depressive symptoms vs no/mild symptoms	Non-opioid analgesics vs no oral analgesics	Regression investigating the influence of showing moderate to severe depressive symptoms (reference: no/mild symptoms) on the use of non-opioid analgesics (reference: no oral analgesics use).	U	RRR: 1.87; 95%CI: .82-4.23<br>p=.135	<u>Univariate</u><br>?<br><4<br><br><u>Multivariate</u><br>?<br><4
				Regression investigating the influence of showing moderate to severe depressive symptoms (reference: no/mild symptoms) on the use of non-opioid analgesics (reference: no oral analgesics use) while also accounting for social support (NS), health literacy (NS), age, sex, race, income, WOMAC, comorbidity and BMI.	M	RRR: 1.93; 95%CI: .72-5.12<br>p=.189	
Depressive symptoms x prescription pain medication use							
Alschuler (2012) [1]	161<br>Multiple sclerosis w/ pain	Patient Health Questionnaire-9<br>→ depressive symptoms yes/no	Current Neurontin use<br>→ yes/no	Chi²	U	Depressive symptoms: 22.6%<br>No symptoms: 16.3%<br>NS	<u>Univariate</u><br>00<br>2/18 – 11%<br><br><u>Multivariate</u><br>?<br><4
			Past Neurontin use<br>→ yes/no	Chi²	U	Depressive symptoms: 19.4%<br>No symptoms: 17.4%<br>NS	
			Current TCA use	Chi²	U	Depressive symptoms: 12.9%	

		→ yes/no			No symptoms: 18.6%<br>NS
		Past TCA use<br>→ yes/no	Chi²	U	Depressive symptoms: 3.2%<br>No symptoms: 3.5%<br>NS
		Current narcotics use<br>→ yes/no	Chi²	U	Depressive symptoms: 25.8%<br>No symptoms: 30.2%<br>NS
		Past narcotics use<br>→ yes/no	Chi²	U	Depressive symptoms: 38.7%<br>No symptoms: 17.4%<br>**p<.05**
		Current Diazepam/Alprazolam use<br>→ yes/no	Chi²	U	Depressive symptoms: 9.7%<br>No symptoms: 16.3%<br>NS
		Past Diazepam/Alprazolom use<br>→ yes/no	Chi²	U	Depressive symptoms: 3.2%<br>No symptoms: 11.6%<br>NS
		Current Tegretol use<br>→ yes/no	Chi²	U	Depressive symptoms: 9.7%<br>No symptoms: 8.1%<br>NS
		Past Tegretol use<br>→ yes/no	Chi²	U	Depressive symptoms: 6.5%<br>No symptoms: 2.3%<br>NS
		Current Baclofen use<br>→ yes/no	Chi²	U	Depressive symptoms: 22.6%<br>No symptoms: 19.8%<br>NS
		Past Baclofen use<br>→ yes/no	Chi²	U	Depressive symptoms: 25.8%<br>No symptoms: 32.6%<br>NS
		Current Dilantin/other anticonvulsant use<br>→ yes/no	Chi²	U	Depressive symptoms: 0.0%<br>No symptoms: 2.3%<br>NS
		Past Dilantin/other anticonvulsant use<br>→ yes/no	Chi²	U	Depressive symptoms: 6.5%<br>No symptoms: 1.2%<br>NS
		Current Marijuana use<br>→ yes/no	Chi²	U	Depressive symptoms: 9.7%<br>No symptoms: 5.8%<br>NS

			Past Marijuana use<br>→ yes/no	Chi²	U	Depressive symptoms: 19.4%<br>No symptoms: 5.8%<br>NS
Asmundson (2001)[5] [2]	108<br>Chronic headache	Beck Depression Inventory (BDI)	Prescription headache medication<br>→ yes/no	Correlation	U	r=.04<br>NS
				Regression investigating the influence of BDI score on the likelihood of using prescription pain medication (reference: no use) while also accounting for cognitive anxiety, fearful appraisals of pain, trait anxiety, trait anger, fear of social concerns, mental incapacitation, distressing nature of headache, degree of lifestyle change (all above: NS – omitted from final model), physiological anxiety (S), fear of physical catastrophe (S) and pain severity (S).	M	NS (omitted from final model)
Kratz (2018) [39]	120<br>Spinal cord injury w/ chronic pain	Patient Health Questionnaire-9	Gabapentin use<br>→ yes/no	Regression investigating the influence of level of depressive symptoms on the likelihood of using gabapentin (reference: no use) while also accounting for pain intensity (NS), number of painful body areas (S) and pain acceptance (S).	M	OR: .98; 95%CI: .89-1.07<br>p=.61
Pierce (2019)[5] [64]	1,785<br>Chronic pain	Hospital Anxiety and Depression Scale – Depression subscale	Benzodiazepine use<br>→ yes/no	To compare level of depressive symptoms between users and non-users of benzodiazepines.	U	Non-users: 9.21 ± 4.37<br>Users: 11.02 ± 4.40<br>**p<.001**
				Regression investigating the influence of level of depressive symptoms on the likelihood of using benzodiazepines (reference: no use) while also accounting for age (NS), sex (NS), pain severity (NS), pain interference (NS), fibromyalgia survey score (S), anxiety symptoms (S), lifetime abuse	M	OR: .98; 95%CI: .937-1.026<br>p=.394

				(NS) and interactions between anxiety and child (NS), adult (NS) and cumulative (S) abuse.			
Depressive symptoms x OTC pain medication use							
Alschuler<br>(2012) [1]	161<br>Multiple sclerosis w/ pain	Patient Health Questionnaire-9<br>→ Depressive symptoms yes/no	Current Acetaminophen use<br>→ yes/no	Chi^2	U	Depressive symptoms: 29.0%<br>No symptoms: 39.5%<br>NS	<u>Univariate</u><br>00<br>2/18 – 11%<br><br><u>Multivariate</u><br>?<br><4
			Past Acetaminophen use<br>→ yes/no	Chi^2	U	Depressive symptoms: 54.8%<br>No symptoms: 31.4%<br>**p<.05**	
			Current Advil/Aspirin/Aleve use<br>→ yes/no	Chi^2	U	Depressive symptoms: 19.4%<br>No symptoms: 32.6%<br>NS	
			Past Advil/Aspirin/Aleve use<br>→ yes/no	Chi^2	U	Depressive symptoms: 61.3%<br>No symptoms: 48.8%<br>NS	
Asmundson<br>(2001)[5] [2]	108<br>Chronic headache	Beck Depression Inventory (BDI)	OTC headache medication<br>→ yes/no	Correlation	U	r=.14<br>NS	
				Regression investigating the influence of BDI score on the likelihood of using OTC pain medication (reference: no use) while also accounting for physiological anxiety, fearful appraisals of pain, trait anxiety, trait anger, fear of social concerns, mental incapacitation, distressing nature of headache, degree of lifestyle change, fear of physical catastrophe (all above: NS – omitted from final model), cognitive anxiety (S) and pain severity (NS – fixed factor).	M	NS (omitted from final model)	
Depressive symptoms x opioid use							
Buse<br>(2012) [5]	5,796<br>Migraine	Patient Health Questionnaire-9<br>→ Depressive symptoms yes/no	Opioid use<br>→ non-users (reference) vs previous users	Regression investigating the influence of presence of depressive symptoms (reference: no symptoms) on the likelihood	U	OR: 1.95; 95%CI: 1.62-2.34<br>**p<.001**	<u>Univariate</u><br>++<br>8/11 – 73%

				of being a previous opioid users (reference: no use).			Multivariate<br>0<br>3/6 – 33%
			Opioid use<br>→ non-users (reference) vs current non-dependent users	Regression investigating the influence of presence of depressive symptoms (reference: no symptoms) on the likelihood of being a current non-dependent opioid user (reference: non-user).	U	OR: 2.41; 95%CI: 2.01-2.88<br>**p<.001**	
			Opioid use<br>→ non-users (reference) vs current dependent users	Regression investigating the influence of presence of depressive symptoms (reference: no symptoms) on the likelihood of being a current dependent opioid user (reference: no use).	U	OR: 6.26; 95%CI: 4.50-8.69<br>**p<.001**	
Carroll (2016)[5] [6]	83<br>Sickle cell disease	Center for Epidemiological Studies Depression Scale	Chronic opioid therapy<br>→ yes/no	Comparison of depression scores between patients on chronic opioid therapy and those who are not.	U	Chronic opioids: 20.2 ± 13.9<br>No chronic opioids: 12.0 ± 8.1<br>**p<.01**	
Gebauer (2019)[5] [22]	327<br>Chronic low back pain	Patient Health Questionnaire-2<br>→ Depressive symptoms yes/no	1-50mg/d MED opioid use vs no use	Regression investigating the influence of presence of depressive symptoms (reference: no symptoms) on the likelihood of using 1-50mg/d MED (reference: no opioid use) while also accounting for moment of assessment (NS), collecting disability (NS), age (NS), race (NS), sex (NS), education (NS), pain severity (NS), pain duration (NS), health-related quality of life (pain interference (NS), physical functioning (NS), role physical (NS) and general health (S)), comorbidities (NS), overweight/obesity (NS), anxiety symptoms (NS), other treatments (NS), having a written pain contract (S) and continuity of care (S).	M	OR: 1.24 ; 95%CI: .65-2.40<br>NS	

			>50mg/d MED opioid use vs no use	Regression investigating the influence of presence of depressive symptoms (reference: no symptoms) on the likelihood of using >50mg/d MED (reference: no opioid use) while also accounting for moment of assessment (S), collecting disability (S), age (NS), race (NS), sex (NS), education (NS), pain severity (NS), pain duration (NS), health-related quality of life (pain interference (NS), physical functioning (NS), role physical (NS) and general health (NS)), comorbidities (NS), overweight/obesity (NS), anxiety symptoms (NS), other treatments (NS), having a written pain contract (S) and continuity of care (NS).	M	OR: 5.32 ; 95%CI: 1.47-19.28<br>**p<.05**
Harden (1997)[5] [27]	200<br>Chronic pain	Beck Depression Inventory (BDI)	Taking daily opioids<br>→ yes/no	Comparison of BDI score between patients taking daily opioids and those who do not.	U	Daily opioids: 19.5 ± 9.7<br>No opioids: 15.7 ± 9.5<br>p<.1
Huffman (2017)[5] [31]	1,457<br>Chronic non-cancer pain following an interdisciplinary program	Depression, Anxiety and Stress Scale – Depression subscale (baseline or post-discharge)	Chronic opioid use<br>→ no use (reference); low dose; high dose (baseline)	Comparison of baseline depressive symptoms score between the 3 opioid use groups.	U	No use: 17.89 ± 12.32<br>Low dose: 18.84 ± 12.34<br>High dose: 21.08 ± 12.61<br>**p<.001**
				Linear mixed model investigating the influence of baseline level of opioid use (reference: no use) on the level of depressive symptoms post-discharge while controlling for marital status (S), age (NS), gender (S) and baseline score for depressive symptoms (S).	M	Low dose: β=-.12; p=.84<br>High dose: β=.22 ; p=.73
Jensen (2006)[5] [33]	160<br>Chronic non-cancer pain	Hospital Anixety and Depression Scale – Depression subscale	Opioid use<br>→ yes/no	Chi²	U	Users: 28%<br>Non-users: 19%<br>**p=.012**

		→ Depressive symptoms yes/no				
Kapoor (2014) [37]	64<br>Chronic pain	Center for Epidemiologic Studies Depression Scale	Opioid use<br>→ yes/no	Correlation	U	r=.141<br>NS
Kratz (2018) [39]	120<br>Spinal cord injury w/ chronic pain	Patient Heealth Questionnaire-9	Opioid use<br>→ yes/no	Regression investigating the influence of level of depressive symptoms on the likelihood of using opioids (reference: no use) while also accounting for pain intensity (NS), number of painful body areas (NS) and pain acceptance (S).	M	OR: .93; 95%CI: .85-1.03<br>p=.16
Newman (2018)[5] [59]	290<br>Chronic pain	Patient Health Questionnaire-9	Opioid use<br>→ yes/no	Correlation	U	r=.03<br>NS
Vina (2019) [79]	360<br>Knee osteoarthritis	Patient Health Questionnaire-8<br>→<br>moderate/severe depressive symptoms vs no/mild symptoms	Oral opioid vs no oral analgesics use	Regression investigating the influence of showing moderate to severe depressive symptoms (reference: no/mild symptoms) on the use of oral opioids (reference: no oral analgesics use).	U	RRR: 4.38; 95%CI: 1.89-10.15<br>**p=.001**
				Regression investigating the influence of showing moderate to severe depressive symptoms (reference: no/mild symptoms) on the use of oral opioids (reference: no oral analgesics use) while accounting for social support (NS), health literacy (NS), age, sex, race, income, WOMAC, comorbidity and BMI.	M	OR: 2.96; 95%CI: 1.08-8.07<br>**P=.035**
			Oral opioids vs oral non-opioid analgesic use	Regression investigating the influence of showing moderate to severe depressive symptoms (reference: no/mild symptoms) on the use of oral opioids (reference: oral non-opioid analgesics use).	U	OR: 2.35; 95%CI: 1.42-3.87<br>**p=.001**

				Regression to investigating the influence of showing moderate to severe depressive symptoms (reference: no/mild symptoms) on the use of oral opioids (reference: oral non-opioid analgesics use) while accounting for social support (NS), health literacy (NS), age, sex, race, income, WOMAC, comorbidity and BMI.	M	OR: 1.53; 95%CI: .87-2.71<br>p=.140	
Depressive symptoms x primary care consultations							
Alschuler<br>(2012) [1]	161<br>Multiple sclerosis w/ pain	Patient Health Questionnaire-9<br>→ Depressive symptoms yes/no	Current use of PT<br>→ yes/no	Chi²	U	Depressive symptoms: 45.2%<br>No symptoms: 44.2%<br>NS	<u>Univariate</u><br>00<br>2/7 – 29%<br><br><u>Multivariate</u><br>?<br><4
			Past use of PT<br>→ yes/no	Chi²	U	Depressive symptoms: 12.9%<br>No symptoms: 12.8%<br>NS	
			Current use of psychotherapy<br>→ yes/no	Chi²	U	Depressive symptoms: 19.4%<br>No symptoms: 14.0%<br>NS	
			Past use of psychotherapy<br>→ yes/no	Chi²	U	Depressive symptoms: 6.5%<br>No symptoms: 1.2%<br>NS	
Jordan<br>(2006)[5] [34]	1,797<br>Knee pain	Hospital Anxiety and Depression Scale – Depression subscale<br>→ Most vs less symptoms (baseline)	Future primary care visit for knee pain<br>→ yes/no	Regression investigating the influence of showing most depressive symptoms (reference: less symptoms) on the likelihood of having a future primary care consultation for knee pain (reference: no consultation).	U	OR: 1.25; 95%CI: .94-1.65<br>NS	
				Regression investigating the influence of showing most depressive symptoms (reference: less symptoms) on the likelihood of having a future primary care consultation for knee pain (reference: no consultation) while also accounting for BMI (S), general anxiety symptoms (NS),	M	OR: 1.09; 95%CI: .77-1.55<br>NS	

				widespread pain (NS), favorable evaluation (NS) and frequency of consulting (S).			
Pagé<br>(2019) [62]	686<br>Chronic low back pain	Beck Depression Inventory<br>(at 6m follow-up)	Psychological treatment<br>→ yes/no<br>(12m follow-up)	Comparison of level of depressive symptoms between users and non-users of psychological treatment.	U	Users: 26.0 ± 11.6<br>Non-users: 16.4 ± 10.0<br>**p<.001**	
		Beck Depression Inventory<br>(at 12m follow-up)	Psychological treatment<br>→ yes/no<br>(12m follow-up)	Comparison of level of depressive symptoms between users and non-users of psychological treatment.	U	Users: 26.5 ± 13.6<br>Non-users: 15.9 ± 9.9<br>**p<.001**	
Depressive symptoms x secondary care consultations							
Boyer<br>(2009) [4]	315<br>Fibromyalgia	Hospital Anxiety and Depression Scale – Depression subscale	Attending rheumatology setting vs primary care	Comparison of level of depressive symptoms between users of a rheumatology setting and primary care users.	U	Rheumatology: 35.96 ± 22.97<br>Primary care: 33.14 ± 20.86<br>NS	Univariate<br>?<br><4<br><br>Multivariate<br>?<br><4
Engel<br>(1996)[5] [20]	1,059<br>Spinal pain	Symptom Checklist-90 – Depression subscale<br>(baseline)	Specialist visits<br>→ yes/no<br>(11m follow-up)	Regression investigating the influence of level of baseline depressive symptoms on the likelihood of having specialist visits (reference: no visits) at follow-up.	U	NS	
				Regression investigating the influence of level of baseline depressive symptoms on the likelihood of having ≥1 specialist visits (reference: <1) at follow-up while also accounting for age, gender, education, chronic pain grade (S), days in pain (S), disability pay (NS) and diagnosis (S).	M	NS	
Vervoort<br>(2019) [77]	199<br>Fibromyalgia	Hospital Anxiety and Depression Scale – Depression subscale<br>(Baseline)	Recurrent secondary care user at 18m follow-up<br>→ yes/no	Regression investigating the influence of baseline level of depressive symptoms on the likelihood of recurrent secondary care use (reference: no secondary care use).	U	OR: 1.10; 95%CI: 1.02-1.19<br>**p=.02**	

				Regression investigating the influence of baseline level of depressive symptoms on the likelihood of recurrent secondary care use (reference: no secondary care use) while also accounting for severity of fibromyalgia, anxiety symptoms, illness perceptions (consequences and personal control), active pain coping, helplessness (all above: NS – omitted from final model) and comorbidity (S).	M	NS (omitted from final model)	
Depressive symptoms x tertiary care consultations							
Fink-Miller (2014)[5] [21]	233<br>Chronic non-cancer pain	Beck Depression Inventory (BDI)	Attending tertiary care (reference: primary care)	Comparison of BDI scores between tertiary and primary care patients.	U	Tertiary care: 12.94<br>Primary care: 14.24<br>NS	Univariate<br>?<br><4
Depressive symptoms x invasive procedures							
Alschuler (2012) [1]	161<br>Multiple sclerosis w/ pain	Patient Health Questionnaire-9<br>→ Depressive symptoms yes/no	Current use of nerve blocks<br>→ yes/no	Chi²	U	Depressive symptoms: 9.7%<br>No symptoms: 1.2%<br>**p<.05**	Univariate<br>00<br>1/5 – 20%
			Current use of implanted nerve stimulator<br>→ yes/no	Chi²	U	Depressive symptoms: 12.9%<br>No symptoms: 9.3%<br>NS	
			Past use of implanted nerve stimulator<br>→ yes/no	Chi²	U	Depressive symptoms: 12.9%<br>No symptoms: 20.9%<br>NS	
			Past use of implanted medication pump<br>→ yes/no	Chi²	U	Depressive symptoms: 0.0%<br>No symptoms: 3.5%<br>NS	
Lozano-Calderon (2008) [44]	72<br>Trapezio-metacarpal joint arthrosis	Center for the Epidemiological Study of Depression	Opting for surgery<br>→ yes/no	Comparison of level of depressive symptoms between patients opting for surgery and those who do not.	U	Surgery: 12.2 ± 9.3<br>No surgery: 11.2 ± 8.3<br>p=.65	
Depressive symptoms x hospitalizations							
Cronin (2019) [12]	201<br>Sickle cell disease	Patient Health Questionnaire-2 (PHQ-2)	Hospitalization → yes/no	Regression investigating the influence of PHQ-2 score on the likelihood of being hospitalized	M	OR: 1.34; 95%CI: 1.04-1.72<br>**p=.021**	Univariate<br>?<br><4

				(reference: no hospitalization) while also accounting for age (NS), sex (NS), education (NS), ability to pay bills (S), literacy (NS), spirituality (NS) and social support (NS).			Multivariate<br>?<br><4
Engel (1996)[5] [20]	1,059<br>Spinal pain	Symptom Checklist-90 – Depression subscale (baseline)	Hospital admissions<br>→ yes/no<br>(11m follow-up)	Regression investigating the influence of level of baseline depressive symptoms on the likelihood of having hospital admissions (reference: no admission) at follow-up.	U	NS	
Depressive symptoms x CAM use							
Alschuler (2012) [1]	161<br>Multiple sclerosis w/ pain	Patient Health Questionnaire-9<br>→ depressive symptoms yes/no	Current use of biofeedback/ relaxation<br>→ yes/no	Chi²	U	Depressive symptoms: 9.7%<br>No symptoms:1.2%<br>**p<.05**	Univariate<br>00<br>6/29 – 21%<br><br>Multivariate<br>0<br>0/5 – 0%
			Past use of biofeedback/ relaxation<br>→ yes/no	Chi²	U	Depressive symptoms: 0.0%<br>No symptoms: 1.2%<br>NS	
			Current use of acupuncture<br>→ yes/no	Chi²	U	Depressive symptoms: 16.1%<br>No symptoms: 17.4%<br>NS	
			Past use of acupuncture<br>→ yes/no	Chi²	U	Depressive symptoms: 3.2%<br>No symptoms: 1.2%<br>NS	
			Current use of magnets<br>→ yes/no	Chi²	U	Depressive symptoms: 9.7%<br>No symptoms: 11.6%<br>NS	
			Past use of magnets<br>→ yes/no	Chi²	U	Depressive symptoms: 0.0%<br>No symptoms: 1.2%<br>NS	
			Current use of massage<br>→ yes/no	Chi²	U	Depressive symptoms: 45.2%<br>No symptoms: 39.5%<br>NS	
			Past use of massage<br>→ yes/no	Chi²	U	Depressive symptoms: 3.2%<br>No symptoms: 10.5%<br>NS	

		Current use of hypnosis<br>→ yes/no	Chi²	U	Depressive symptoms: 3.2%<br>No symptoms: 2.3%<br>NS
		Current use of TENS unit<br>→ yes/no	Chi²	U	Depressive symptoms: 25.8%<br>No symptoms: 9.3%<br>**p<.05**
		Past use of TENS unit<br>→ yes/no	Chi²	U	Depressive symptoms: 0.0%<br>No symptoms: 3.5%<br>NS
		Current use of chiropractic adjustment<br>→ yes/no	Chi²	U	Depressive symptoms: 22.6%<br>No symptoms: 23.3%<br>NS
		Past use of chiropractic adjustment<br>→ yes/no	Chi²	U	Depressive symptoms: 9.7%<br>No symptoms: 8.1%<br>NS
		Current use of heat<br>→ yes/no	Chi²	U	Depressive symptoms: 29.0%<br>No symptoms: 30.2%<br>NS
		Past use of heat<br>→ yes/no	Chi²	U	Depressive symptoms: 45.2%<br>No symptoms:14.0%<br>**p<.001**
		Current use of ice<br>→ yes/no	Chi²	U	Depressive symptoms: 41.9%<br>No symptoms: 38.4%<br>NS
		Past use of ice<br>→ yes/no	Chi²	U	Depressive symptoms: 29.0%<br>No symptoms: 14.0%<br>NS
		Current use of strengthening exercises<br>→ yes/no	Chi²	U	Depressive symptoms: 16.1%<br>No symptoms: 23.3%<br>NS
		Past use of strengthening exercises<br>→ yes/no	Chi²	U	Depressive symptoms: 54.8%<br>No symptoms: 48.8%<br>NS
		Current use of mobility exercises<br>→ yes/no	Chi²	U	Depressive symptoms: 16.1%<br>No symptoms: 14.0%<br>NS

			Past use of mobility exercises<br>→ yes/no	Chi²	U	Depressive symptoms: 45.2%<br>No symptoms: 34.9%<br>NS
Ndao-Brumblay (2010)[5] [58]	5,079<br>Chronic pain	Beck Depression Inventory	CAM use (acupuncture, manipulation and biofeedback/relaxation use)<br>→ yes/no	Comparison of level of depressive symptoms between CAM users and non-users.	U	Users: 17.00 ± 10.54<br>Non-users: 16.08 ± 10.62<br>**p≤.005**
				Regression investigating the influence of level of depressive symptoms on the likelihood of using CAM modalities (reference: no use) while accounting for age (S), gender (NS), race (S), education (S), marital status (NS), pain care perception (S), perceived pain control (S), pain prediction (NS), residence income (NS), comorbidities (S), number of operations (NS), pain duration (S), pain severity (S) and functional limitations (S).	M	OR: .999<br>NS
			Acupuncture<br>→ yes/no	Comparison of level of depressive symptoms between acupuncture users and non-users.	U	Users: 16.4 ± 10.00<br>Non-users: 16.4 ± 10.6<br>NS
				Regression investigating the influence of the level of depressive symptoms on the likelihood of using acupuncture (reference: no use) while accounting for age (S), gender (NS), race (NS), education (S), marital status (NS), pain care perception (S), perceived pain control (NS), pain prediction (NS), residence income (NS), comorbidities (NS), number of operations (NS), pain duration (S), pain severity (S) and functional limitations (NS).	M	OR: .996<br>NS
			Biofeedback/relaxation<br>→ yes/no	Comparison of level of depressive symptoms between	U	Users: 18.41 ± 11.06<br>Non-users: 16.10 ± 10.50

				biofeedback/relaxation users and non-users.		**p≤.005**
				Regression investigating the influence of level of depressive symptoms on the likelihood of using biofeedback/relaxation (reference: no use) while accounting for age (S), gender (NS), race (S), education (S), marital status (NS), pain care perception (NS), perceived pain control (S), pain prediction (NS), residence income (NS), comorbidities (S), number of operations (NS), pain duration (S), pain severity (S) and functional limitations (S).	M	OR: 1.002<br>NS
			Manipulation<br>→ yes/no	Comparison of level of depressive symptoms between manipulation users and non-users.	U	Users: 16.73 ± 10.31<br>Non-users: 16.29 ± 10.69<br>NS
				Regression investigating the influence of the level of depressive symptoms on the likelihood of using manipulation services (reference: no use) while accounting for age (NS), gender (NS), race (S), education (S), marital status (NS), pain care perception (S), perceived pain control (S), pain prediction (NS), residence income (NS), comorbidities (S), number of operations (NS), pain duration (S), pain severity (S) and functional limitations (NS).	M	NS (omitted from final model)
Pagé<br>(2019) [62]	686<br>Chronic low back pain	Beck Depression Inventory<br>(6m follow-up)	Self-management modalities<br>→ yes/no<br>(12m follow-up)	Comparison of level of depressive symptoms between users and non-users of self-management modalities.	U	p>.05

		Beck Depression Inventory (12m follow-up)	Self-management modalities → yes/no (12m follow-up)	Comparison of level of depressive symptoms between users and non-users of self-management modalities.	U	p>.05	
Rosenberg (2008) [66]	463 Chronic noncancer pain	Self-designed question → Depressive symptoms yes/no	CAM use → yes/no	Bivariate analysis investigating the influence of presence of depressive symptoms (reference: no symptoms) on the likelihood of using CAM services (reference: no use).	U	OR: 1.16; 95%CI: .78-1.71<br>p=.46	
van Tilburg (2008) [76]	1,012 Functional bowel disorder	Brief Symptom Inventory – Depression subscale	CAM use → yes/no	Comparison of level of depressive symptoms between CAM users and non-users.	U	Users: 5.0 ± 4.4<br>Non-users: 3.5 ± 4.8<br>**p<.001**	
				Regression investigating the influence of level of depressive symptoms on the likelihood of using CAM services (reference: no CAM use) while also accounting for age (NS), sex (S), education (S), symptom severity (NS), suffering from distention (NS), anxiety symptoms (S), somatization (NS), quality of life (NS), non-prescription costs (NS) and satisfactory relief of bowel symptoms (NS).	M	NS	
Fear-avoidance beliefs x consultations							
Mannion (2013) [49]	1,071 Low back pain	Fear-Avoidance Beliefs Questionnaire – Activity beliefs subscale	Consultation w/ specialist, GP, PT or other practitioner → yes/no	Regression investigating the influence of level of activity fear-avoidance beliefs on the likelihood of having a consultation (reference: no consultation).	U	OR: 1.047; 95%CI: 1.033-1.060<br>**p<.0001**	Univariate<br>?<br><4<br><br>Multivariate<br>?<br><4
				Regression investigating the influence of level of activity fear-avoidance beliefs on the likelihood of having a consultation (reference: no consultation) while also accounting for sex (S), age (NS), education (NS), general health	M	OR: 1.017; 95%CI: .982-1.053<br>p=.34	

				(NS), anxiety/depression (NS), working status (NS), household -18y (NS), income (NS), low back pain frequency (S),low back pain intensity (NS), limitations in ADL (S) and work fear-avoidance beliefs (S).			
		Fear-Avoidance Beliefs Questionnaire – Work beliefs subscale	Consultation w/ specialist, GP, PT or other practitioner → yes/no	Regression investigating the influence of level of work fear-avoidance beliefs on the likelihood of having a consultation (reference: no consultation).	U	OR: 1.086; 95%CI: 1.060-1.112 **p<.0001**	
				Regression investigating the influence of level of work beliefs on the likelihood of having a consultation (reference: no consultation) while also accounting for sex (S), age (NS), education (NS), general health (NS), anxiety/depression (NS), working status (NS), household -18y (NS), income (NS), low back pain frequency (S), low back pain intensity (NS), limitations in ADL (S) and activity fear-avoidance beliefs (NS).	M	OR: 1.025; 95%CI: 1.005-1.044 **p=.012**	
Frustration x pain medication use							
Hill (2007) [29]	2,113 Musculoskeletal hand problems	Arthritis Impact Measurement Scale-2 – Frustration subscale → frustration on few or all days vs no days w/ frustration	Pain medication use → yes/no	Regression investigating the influence of frustration on few days or more (reference: no frustration days) on the likelihood of using pain medication (reference: no use).	U	OR: 3.40; 95%CI: 2.63-4.40 **Significant**	<u>Univariate</u><br>?<br><4<br><br><u>Multivariate</u><br>?<br><4
				Regression investigating the influence of showing frustration on few days or more (reference: no frustration days) on the likelihood of using pain medication (reference: no use) while also accounting for sex (NS	M	OR: 1.91; 95%CI: 1.28-2.85 **Significant**	

				– fixed factor), age (NS – fixed factor), diagnosis (S – fixed factor) and Illness Perception Questionnaire subscales (timeline cyclical (S), identity (S), consequences (S), emotional representations (S), treatment control (S) and illness coherence (S)).			
Frustration x primary care consultations							
Hill (2007) [29]	2,113 Musculoskeletal hand problems	Arthritis Impact Measurement Scale-2 – Frustration subscale → frustration on few or all days vs no days w/ frustration	GP consultation → yes/no	Regression investigating the influence of showing frustration on few days or more (reference: no frustration days) on the likelihood of having a GP consultation (reference: no consultation) while also accounting for Illness Perception Questionnaire items (timeline cyclical, personal control, illness coherence and psychological attributions; all NS – omitted from final model), sex (NS – fixed factor), age (S – fixed factor), diagnosis (NS – fixed factor) and remaining Illness Perception Questionnaire items (timeline acute/chronic, consequences, treatment control, emotional representations and identity; all S).	M	NS (omitted from final model)	<u>Multivariate</u><br>?<br><4
Health worry x primary care consultations							
Macfarlane (1999) [46]	252 Chronic widespread pain	Illness Attitude Scale – Worry about health subscale	GP consultation for pain → yes/no	Comparison of the level of worry about health between consulters and non-consulters.	U	NS	<u>Univariate</u><br>?<br><4
		Illness Attitude Scale – Concerns about pain subscale	GP consultation for pain → yes/no	Comparison of the level of concerns about pain between consulters and non-consulters.	U	NS	
Helplessness x secondary care consultations							

Vervoort (2019) [77]	199 Fibromyalgia	Illness Cognition Questionnaire – Helplessness subscale (Baseline)	Recurrent secondary care user at 18m follow-up → yes/no	Regression investigating the influence of baseline level of helplessness on the likelihood of recurrent secondary care use (reference: no secondary care use).	U	OR: 1.08; 95%CI: 1.00-1.17 **p=.05**	<u>Univariate</u> ? <4 <u>Multivariate</u> ? <4
				Regression investigating the influence of baseline level of helplessness on the likelihood of recurrent secondary care use (reference: no secondary care use) while also accounting for severity of fibromyalgia, depressive and anxiety symptoms, illness perceptions (consequences and personal control), active pain coping (all above: NS – omitted from final model) and comorbidity (S).	M	NS (omitted from final model)	
Negative consequences beliefs x pain medication use							
Hill (2007) [29]	2,113 Musculoskeletal hand problems	Illness Perception Questionnaire – Revised (IPQ-R) – Consequences subscale	Pain medication use → yes/no	Regression investigating the influence of IPQ-R consequences score on the likelihood of using pain medication (reference: no use).	U	OR: 1.21; 95%CI: 1.17-1.23 **Significant**	<u>Univariate</u> ? <4 <u>Multivariate</u> ? <4
				Regression investigating the influence of IPQ-R consequences score on the likelihood of using pain medication (reference: no use) while also accounting for sex (NS – fixed factor), age (NS – fixed factor), diagnosis (S – fixed factor), IPQ-R subscales (timeline cyclical (S), identity (S), emotional representations (S), treatment control (S), and illness coherence (S)) and frustration (S).	M	OR: 1.12; 95%CI: 1.08-1.16 **Significant**	
Negative consequences beliefs x primary care consultations							
Hill	2,113	Illness Perception Questionnaire –	GP consultation → yes/no	Regression investigating the influence of IPQ-R consequences	U	OR: 1.16; 95%CI: 1.14-1.19 **Significant**	<u>Univariate</u> ?

(2007) [29]	Musculoskeletal hand problems	Revised (IPQ-R) – Consequences subscale		score on the likelihood of having a GP consultation (reference: no consultation).			<4<br><br>Multivariate<br>?<br><4
				Regression investigating the influence of IPQ-R consequences score on the likelihood of having a GP consultation (reference: no consultation) while also accounting for sex (NS – fixed factor), age (S – fixed factor), diagnosis (NS – fixed factor) and IPQ-R subscales (timeline acute/chronic, emotional representations, identity and treatment control; all S).	M	OR: 1.09; 95%CI: 1.05-1.14<br>**Significant**	
Negative consequences beliefs x secondary care consultations							
Vervoort (2019) [77]	199 Fibromyalgia	Revised Fibromyalgia Illness Perception Questionnaire – Consequences subscale (Baseline)	Recurrent secondary care user at 18m follow-up → yes/no	Regression to investigate the influence of level of IPQR consequences subscale on the likelihood of recurrent secondary care use (reference: no secondary care use).	U	OR: 1.08; 95%CI: 1..01-1.16<br>**p=.02**	Univariate<br>?<br><4<br><br>Multivariate<br>?<br><4
				Regression investigating the influence of baseline level of negative consequences beliefs on the likelihood of recurrent secondary care use (reference: no secondary care use) while also accounting for severity of fibromyalgia, depressive and anxiety symptoms, helplessness perceived personal symptom control, active pain coping (all above: NS – omitted from final model) and comorbidity (S).	M	NS (omitted from final model)	
Negative illness beliefs x pain medication use							
Hill (2007) [29]	2,113 Musculoskeletal hand problems	Illness Perception Questionnaire – Revised (IPQ-R) – Timeline cyclical subscale	Pain medication use → yes/no	Regression investigating the influence of IPQ-R timeline cyclical score on the likelihood of using pain medication (reference: no use).	U	OR: 1.03; 95%CI: 1.00-1.05<br>**significant**	Univariate<br>?<br><4<br><br>Multivariate

				Regression investigating the influence of IPQ-R timeline cyclical score on the likelihood of using pain medication (reference: no use) while also accounting for sex (NS – fixed factor), age (NS – fixed factor), diagnosis (S – fixed factor), IPQ-R subscales (consequences (S), identity (S), emotional representations (S), treatment control (S), and illness coherence (S)) and frustration (S).	M	OR: 1.05; 95%CI: 1.01-1.09<br>**significant**	?<br><4
		Illness Perception Questionnaire – Revised (IPQ-R) – Timeline acute/chronic subscale<br>→ low vs high score	Pain medication use → yes/no	Regression investigating the influence of IPQ-R timeline acute/chronic on (reference: low score) on the likelihood of using pain medication (reference: no use) while also accounting for IPQ-R items (personal control and psychological attributions; both NS – omitted from final model), sex (NS – fixed factor), age (NS – fixed factor) and diagnosis (S – fixed factor), remaining IPQ-R items (timeline cyclical, consequences, treatment control, emotional representations, illness coherence and identity; all S) and frustration score (S).	M	NS (omitted from final model)	
Negative illness beliefs x consultations							
Mannion (2013) [49]	1,071<br>Low back pain	Back Beliefs Questionnaire (BBQ)	Consultation w/ specialist, GP, PT or other practitioner<br>→ yes/no	Regression investigating whether BBQ score was influencing the likelihood of having a consultation (reference: no consultation).	U	OR: 1.018; 95%CI: 1.005-1.032<br>**p=.007**	<u>Univariate</u><br>?<br><4
				Regression investigating whether BBQ score was influencing the likelihood of having a consultation (reference: no	M	OR: .991; 95%CI: .974-1.008<br>p=.991	<u>Multivariate</u><br>?<br><4

				consultation) while also accounting for sex (NS), age (NS), education (NS), general health (NS), anxiety/depression (NS), working status (NS), household - 18y (NS), income (NS), LBP frequency (S), LBP intensity (S) and limitations in ADL (S).			
Negative illness beliefs x primary care consultations							
Hill<br>(2007) [29]	2,113<br>Musculoskeletal hand problems	Illness Perception Questionnaire (IPQ-R) – Timeline cyclical subscale	GP consultation → yes/no	Regression investigating the influence of IPQ-R timeline cyclical score on the likelihood of pain medication use (reference: no use) while also accounting for IPQ-R items (personal control, illness coherence and psychological attributions), frustration (all above: NS – omitted from final model), sex (NS – fixed factor), age (S – fixed factor), diagnosis (NS – fixed factor) and remaining IPQ-R items (timeline acute/chronic, consequences, treatment control, emotional representations and identity; all S).	M	NS (omitted from final model)	<u>Univariate</u><br>?<br><4<br><br><u>Multivariate</u><br>?<br><4
		Illness perception Questionnaire – Revised (IPQ-R) – Timeline acute/chronic subscale	GP consultation → yes/no	Regression investigating the influence of having a high IPQ-R timeline acute/chronic score (reference: low score) on the likelihood of having a GP consultation (reference: no consultation).	U	OR: 2.19; 95%CI: 1.76-2.72<br>**Significant**	

		→ low vs high score		Regression investigating the influence of having a high IPQ-R timeline acute/chronic score (reference: low score) on the likelihood of having a GP consultation (reference: no consultation) while also accounting for sex (NS – fixed factor), age (S – fixed factor), diagnosis (NS – fixed factor) and IPQ-R subscales (emotional representations, identity, consequences and treatment control; all S).	M	OR: 1.65; 95%CI: 1.17-2.34<br>**Significant**	
Negative illness beliefs x secondary care consultations							
Vervoort (2019) [77]	199 Fibromyalgia	Revised Fibromyalgia Illness Perception Questionnaire – Timeline acute/chronic subscale (baseline)	Recurrent secondary care user at 18m follow-up<br>→ yes/no	Regression investigating the influence of baseline level of IPQR timeline acute/chronic subscale on the likelihood of recurrent secondary care use (reference: no secondary care use).	U	NS	<u>Univariate</u><br>?<br><4
		Revised Fibromyalgia Illness Perception Questionnaire – Timeline cyclical subscale (baseline)	Recurrent secondary care user at 18m follow-up<br>→ yes/no	Regression investigating the influence of baseline level of IPQR timeline cyclical subscale on the likelihood of recurrent secondary care use (reference: no secondary care use).	U	OR: 1.03; 95%CI: .94-1.12<br>p=.52	
Psychological distress x pain medication use							
Hill (2007) [29]	2,113 Musculoskeletal hand problems	Illness Perception Questionnaire – Revised (IPQ-R) – Emotional representations subscale	Pain medication use<br>→ yes/no	Regression investigating the influence of IPQ-R emotional representations score on the likelihood of using pain medication (reference: no use).	U	OR: 1.16; 95%CI: 1.13-1.18<br>**Significant**	<u>Univariate</u><br>?<br><4
				Regression investigating the influence of IPQ-R emotional representations score on the likelihood of using pain	M	OR: 1.04; 95%CI: 1.00-1.08<br>**Significant**	<u>Multivariate</u><br>?<br><4

				medication (reference: no use) while also accounting for sex (NS – fixed factor), age (NS – fixed factor), diagnosis (S – fixed factor), IPQ-R subscales (timeline cyclical (S), identity (S), consequences (S), treatment control (S), and illness coherence (S)) and frustration (S).			
Psychological distress x prescription pain medication use							
Navabi (2018)[5] [57]	432<br>Irritable bowel disease	Hospital Anxiety and Depression Scale<br>→ presence of depressive and/or anxiety symptoms yes/no	Corticosteroid use<br>→ yes/no	Chi²	U	w/o symptoms: n=88/185<br>w/ symptoms: n=97/185<br>**p<.01**	<u>Univariate</u><br>?<br><4<br><br><u>Multivariate</u><br>?<br><4
	283<br>Patients w/ endoscopic evaluation of irritable bowel disease	Hospital Anxiety and Depression Scale<br>→ presence of depressive and/or anxiety symptoms yes/no	Corticosteroid use<br>→ yes/no	Regression investigating the influence of using corticosteroids (reference: no use) on the likelihood of showing depressive and/or anxiety symptoms (reference: no symptoms), while also accounting for significant inflammation (NS), age (NS), disease duration (S), female gender (S), mesalamine use (NS), immunomodulator use (NS), Anti-TNF use (NS) and history of surgery (S), extra-intestinal manifestations (S), tobacco use (S) and opiate use (NS).	M	OR: 1.14; 95%CI: .67-1.95<br>p=.62	
Torrance (2013) [70]	215<br>Chronic pain w/ neuropathic component	SF-12<br>→ Mental Component Scale	Adequate trial of neuropathic pain drug<br>→ yes/no	Comparison of SF-12 mental component score between patients w/ and w/o adequate trial of a neuropathic pain drug.	U	w/ trial: 40.5 ± 11.7<br>w/o trial: 46.6 ± 11.0<br>(positive association due to scoring SF-12)<br>**p<.001**	
Psychological distress x opioid use							
Harden (1997)[5] [27]	200<br>Chronic pain	Multidimensional Pain Inventory	Taking daily opioids<br>→ yes/no	To compare MPI – affective distress score between patients	U	Daily opioids: 3.6 ± 1.2<br>No opioids: 3.4 ± 1.2<br>p>.1	<u>Univariate</u><br>?<br><4

		(MPI) – Affective distress subscale		taking daily opioids and those who do not.			<u>Multivariate</u>
Jensen (2006)[5] [33]	160<br>Chronic non-cancer pain	SF-36 – Mental health subscale	Opioid use<br>→ yes/no	Comparison of SF-36 mental health scores between patients using and not using opioids.	U	Lower SF-36 scores in opioid users<br>**p=.009**	00<br>0/4 – 0%
Lentz (2018) [42]	246<br>Musculoskeletal pain	OSPRO Yellow Flag Tool - 10-item version (OSPRO-YF-10)<br>(baseline)	Use of opioids after PT treatment<br>→ yes/no	Regression investigating the influence of baseline OSPRO-YF-10 score on the likelihood of using opioids (reference: no use) after PT treatment while also accounting for surgery for current condition (NS), comorbidity (S), baseline pain intensity (S), change in pain intensity (S) and baseline OSPRO-YF remaining 7 items (NS).	M	OR: 1.00<br>p=.96	
		OSPRO Yellow Flag Tool (OSPRO-YF) – remaining 7 items<br>(baseline)	Use of opioids after PT treatment<br>→ yes/no	Regression investigating the influence of baseline OSPRO-YF remaining 7 items score on the likelihood of using opioids (reference: no use) after PT treatment while also accounting for surgery for current condition (NS), comorbidity (S), baseline pain intensity (S), change in pain intensity (S) and baseline 10-item OSPRO-YF score (NS).	M	OR: .91<br>p=.07	
		OSPRO Yellow Flag Tool → 10-item shortened version (OSPRO-YF-10<br>(baseline-to-4w change score)	Use of opioids after PT treatment<br>→ yes/no	Regression investigating the influence of baseline-to-4w change in OSPRO-YF-10 score on the likelihood of using opioids (reference: no use) after PT treatment while also accounting for age, sex, race, anatomical region of pain, OSPRO Review of Systems score (10-item + 13 items), baseline disability, insurance, chronicity, change in disability (all above: NS – omitted from final model), baseline 10-item OSPRO-YF (NS), baseline	M	NS (omitted from final model)	

				OSPRO-YF remaining 7 items (NS), surgery for current condition (NS), comorbidity (S), baseline pain intensity (S) and change in pain intensity (S).			
Navabi (2018)[5] [57]	432<br>Irritable bowel disease	Hospital Anxiety and Depression Scale<br>→ Presence of depressive and/or anxiety symptoms yes/no	Current opiate use<br>→ yes/no	Chi²	U	w/o symptoms: n=32/82<br>w/ symptoms: n=50/82<br>**p<.001**	
	283<br>Patients w/ endoscopic evaluation of irritable bowel disease	Hospital Anxiety and Depression Scale<br>→ Presence of depressive and/or anxiety symptoms yes/no	History of opiate use<br>→ yes/no	Regression investigating the influence of having a history of opiate use (reference: no previous use) on the likelihood of showing depressive and/or anxiety symptoms (reference: no symptoms), while also accounting for significant inflammation (NS), age (NS), disease duration (S), female gender (S), mesalamine use (NS), immunomodulator use (NS), Anti-TNF use (NS), corticosteroid use (NS) and history of surgery (S), extra-intestinal manifestations (S) and tobacco use (S).	M	OR: 1.62; 95%CI: .85-3.10<br>p=.14	
Psychological distress x consultations							
Macfarlane (2003) [47]	555<br>Orofacial pain	General Health Questionnaire-12<br>→ Subdivided into score groups:<br>- 0<br>- 1-3<br>- 4-12	Consultation for orofacial pain<br>→ yes/no	Regression investigating the influence of level of psychological distress (reference score: 0) on the likelihood of having a healthcare consultation for orofacial pain (reference: no consultation).	U	Score 1-3: RR:.95; 95%CI: .75-1.20<br>Score 4-12: RR: 1.09; 95%CI: .88-1.35<br>p=.44	<u>Univariate</u><br>00<br>0/12 – 0%<br><br><u>Multivariate</u><br>0<br>1/8 – 13%
Mannion (2013) [49]	1,071<br>Low back pain	Euroquol (EQ5D) – Anxiety/ depression subscale	Consultation w/ specialist, GP, PT or other practitioner<br>→ yes/no	Regression investigating the influence of level of psychological distress on the likelihood of having a	U	OR: 1.266; 95%CI: .961-1.667<br>p=.093	

				consultation (reference: no consultation).		
				Regression investigating the influence of level of psychological distress on the likelihood of having a consultation (reference: no consultation) while also accounting for sex (S), age (NS), education (NS), general health (NS), working status (NS), household -18y (NS), income (NS), low back pain frequency (S),low back pain intensity (NS), limitations in ADL (S), FABQ activity beliefs (NS) and FABQ work beliefs (S).	M	OR: .975; 95%CI: .678-1.402<br>p=.891
Talley<br>(1998) [68]	93<br>Dyspepsia	General Health Questionnaire	Physician and alternative therapist consultations for abdominal pain in the past year<br>→ yes/no	Regression investigating the influence of level of psychological distress on the likelihood of having a consultation for abdominal pain in the past year (reference: no consultation).	U	OR: 1.00; 95%CI: .93-1.08<br>NS
			Physician and alternative therapist consultations for abdominal pain at any time<br>→ yes/no	Regression investigating the influence of level of psychological distress on the likelihood of having a consultation for abdominal pain at any time (reference: no consultation).	U	OR: .97; 95%CI: .90-1.04<br>NS
Thorstensson<br>(2009) [69]	1,119<br>Chronic hip/knee pain	Euroqol EQ5D<br>→ Anxiety/ depression subscale<br>→ Anxiety/ depressive symptoms yes/no	Visits w/ GP, allied health professional or alternative therapist for knee/hip pain<br>→ yes/no	Regression investigating the influence of presence of anxiety/depressive symptoms (reference: no symptoms) on the likelihood of consulting a health professional (reference: no consultation) while also accounting for age (NS), sex (NS), BMI (S), deprivation (NS), living area (NS), pain location (NS),	M	OR: 1.04; 95%CI: .73-1.49<br>NS

				pain severity (S), mobility problems (S) and comorbidities (NS).		
			Visits w/ allied health professional → yes/no	Regression investigating the influence of presence of anxiety/depressive symptoms (reference: no symptoms) on the likelihood of consulting an allied health professional (reference: no consultation) while also accounting for age (NS) and sex (NS).	M	OR: 1.84; 95%CI: .93-3.65<br>NS
Von Korff (1991)[5] [80]	411<br>Back pain	Symptom Checklist Revised → low vs mild/moderate vs severe psychological distress	Visit w/ doctor, PT, dentist, chiropractor or other healthcare professional → yes/no	Comparison of level of psychological distress between care seekers and non-seekers.	U	% seeking care:<br>Low distress: 25.3%<br>Mild/moderate distress: 27.7%<br>Severe distress: 29.2%<br>NS
				Regression investigating the influence of level of psychological distress (reference: low distress) on the likelihood of seeking care for pain (reference: no care seeking) while also accounting for age (NS), sex (NS), distant onset (S), persistent pain (S), pain severity (S) and self-rated health (NS).	M	Mild/moderate distress:<br>OR: .7; NS<br>Severe distress:<br>OR: .9; NS
	263<br>Headache	Symptom Checklist Revised → low vs mild/moderate vs severe psychological distress	Visit w/ doctor, PT, dentist, chiropractor or other healthcare professional → yes/no	Comparison of level of psychological distress between care seekers and non-seekers.	U	% seeking care:<br>Low distress: 19.0%<br>Mild/moderate distress: 17.1%<br>Severe distress: 32.7%<br>p<.1
				Regression investigating the influence of level of psychological distress (reference: low distress) on the likelihood of seeking care for pain (reference: no care seeking) while also accounting for age (S), sex (NS), distant onset (S), persistent pain	M	Mild/moderate distress:<br>OR: .5; p<.1<br>Severe distress:<br>OR: 1.3; NS

			(S), pain severity (S) and self-rated health (S).		
172<br>Abdominal pain	Symptom Checklist Revised<br>→ low vs mild/moderate vs severe psychological distress	Visit w/ doctor, PT, dentist, chiropractor or other healthcare professional<br>→ yes/no	Comparison of level of psychological distress between care seekers and non-seekers.	U	% seeking care:<br>Low distress: 22.5%<br>Mild/moderate distress: 28.8%<br>Severe distress: 41.0%<br>NS
			Regression investigating the influence of level of psychological distress (reference: low distress) on the likelihood of seeking care for pain (reference: no care seeking) while also accounting for age (S), sex (S), distant onset (S), persistent pain (S), pain severity (S) and self-rated health (NS).	M	Mild/moderate distress:<br>OR: 1.6; NS<br>Severe distress:<br>OR: 2.2; NS
118<br>Chest pain	Symptom Checklist Revised<br>→ low vs mild/moderate vs severe psychological distress	Visit w/ doctor, PT, dentist, chiropractor or other healthcare professional<br>→ yes/no	Comparison of level of psychological distress between care seekers and non-seekers.	U	% seeking care:<br>Low distress: 37.5%<br>Mild/moderate distress: 27.5%<br>Severe distress: 40.0%<br>NS
			Regression investigating the influence of level of psychological distress (reference: low distress) on the likelihood of seeking care for pain (reference: no care seeking) while also accounting for age (NS), sex (NS), distant onset (NS), persistent pain (NS), pain severity (S) and self-rated health (NS).	M	Mild/moderate distress:<br>OR: .3; **p<.05**<br>Severe distress:<br>OR: 1.0; NS
121<br>TMD pain	Symptom Checklist Revised<br>→ low vs mild/moderate vs severe psychological distress	Visit w/ doctor, PT, dentist, chiropractor or other healthcare professional<br>→ yes/no	Comparison of level of psychological distress between care seekers and non-seekers.	U	% seeking care:<br>Low distress: 27.8%<br>Mild/moderate distress: 15.4%<br>Severe distress: 25.9%<br>NS
			Regression investigating the influence of level of psychological distress (reference: low distress) on the likelihood of	M	Mild/moderate distress:<br>OR: .4; NS<br>Severe distress:<br>OR: 1.3; NS

				seeking care for pain (reference: no care seeking) while also accounting for age (S), sex (NS), distant onset (S), persistent pain (NS), pain severity (NS) and self-rated health (NS).			
Williams (2006) [85]	337<br>IBS	K6 scale for psychological distress	Doctor's visit for abdominal symptoms → yes/no	Comparison of level of psychological distress between healthcare seekers and non-seekers.	U	Seekers: 8.0<br>Non-seekers: 7.7<br>p=.70	
Zebenholzer (2016) [89]	392<br>Episodic and chronic headache	Hospital Anxiety and Depression Scale<br>→ Anxiety and/or depressive symptoms yes/no	Consultations for headache<br>→ yes/no	Chi²	U	NS	
			Headache-related examinations<br>→ yes/no	Chi²	U	NS	
Psychological distress x primary care consultations							
Hill (2007) [29]	2,113<br>Musculoskeletal hand problems	Illness Perception Questionnaire - Revised (IPQ-R) – Emotional representations subscale	Having a GP consultation → yes/no	Regression investigating the influence of IPQ-R emotional representations score on the likelihood of having a GP consultation (reference: no consultation).	U	OR: 1.16; 95%CI: 1.13-1.19<br>**Significant**	<u>Univariate</u><br>?<br><4<br><br><u>Multivariate</u><br>?<br>2/4 – 50%
				Regression investigating the influence of IPQ-R emotional representations score on the likelihood of having a GP consultation (reference: no consultation) while also accounting for sex (NS – fixed factor), age (S – fixed factor), diagnosis (NS – fixed factor) and IPQ-R subscales (timeline acute/chronic, identity, consequences and treatment control; all S).	M	OR: 1.09; 95%CI: 1.04-1.14<br>**Significant**	
Macfarlane (1999) [46]	252<br>Chronic widespread pain	General Health Questionnaire	GP consultation for pain<br>→ yes/no	Comparison of level of psychological distress between consulters and non-consulters.	U	**p≤.03**	

		General Health Questionnaire (GHQ)<br>→ Psychological distress >median/≤median	GP consultation for pain<br>→ yes/no	Regression investigating the influence of scoring >median on the GHQ (reference: ≤median) on the likelihood of having a GP visit for pain (reference: no visit) while adjusting for age in men.	M	NS	
				Regression investigating the influence of scoring >median on the GHQ (reference: ≤median) on the likelihood of having a GP visit for pain (reference: no visit) while adjusting for age in women.	M	**Significant**	
Thorstensson (2009) [69]	1,119<br>Chronic hip/knee pain	Euroqol EQ5D<br>→ Anxiety/ depression subscale<br>→ Anxiety/ depressive symptoms yes/no	GP visits for knee/hip pain<br>→ yes/no	Regression investigating the influence of presence of anxiety/depressive symptoms (reference: no symptoms) on the likelihood of consulting a GP (reference: no consultation) while also accounting for age (NS), sex (NS), BMI (S), deprivation (NS), living area (S), pain location (NS), pain severity (S), mobility problems (S) and comorbidities (NS).	M	OR: .88; 95%CI: .58-1.33<br>NS	
Trask (2001) [71]	292<br>Headache	Brief Symptom Inventory<br>→ Low/medium/ high distress	Psychological care<br>→ yes/no	Chi²	U	% using psychological care:<br>Low distress: 8.7%<br>Medium distress: 9.9%<br>High distress: 30.6%<br>**p<.05**	
Psychological distress x secondary care consultations							
Lentz (2018) [42]	246<br>musculoskeletal pain	OSPRO Yellow Flag Tool – 10-item version (OSPRO-YF-10) (baseline)	Diagnostic tests or imaging consultations after PT treatment<br>→ yes/no	Regression investigating the influence of baseline OSPRO-YF-10 score on the likelihood of having diagnostic tests (reference: no tests) after PT treatment while also accounting for age, sex, race, anatomical region of pain, OSPRO Review of Systems score (10-item + 13 items), baseline OSPRO-YF	M	NS (omitted from final model)	Univariate<br>?<br><4<br><br>Multivariate<br>?<br><4

			remaining 7 items, OSPRO-YF-10 change score, insurance, baseline pain intensity, chronicity, change in disability, surgery for current condition (all above: NS – omitted from final model), comorbidity (S), baseline disability (S) and change in pain intensity (S).		
	OSPRO Yellow Flag Tool – remaining 7 items (baseline)	Diagnostic tests or imaging consultations after PT treatment → yes/no	Regression investigating the influence of baseline OSPRO-YF remaining 7 items score on the likelihood of having diagnostic tests (reference: no tests) after PT treatment while also accounting for age, sex, race, anatomical region of pain, OSPRO Review of Systems score (10-item + 13 items), baseline 10-item OSPRO-YF score, 10-item OSPRO-YF change score, insurance, baseline pain intensity, chronicity, change in disability, surgery for current condition (all above: NS – omitted from final model), comorbidity (S), baseline disability (S) and change in pain intensity (S).	M	NS (omitted from final model)
	OSPRO Yellow flag tool - 10-item version (OSPRO-YF-10) (baseline-to-4w change score)	Having diagnostic tests or imaging consultations after PT treatment → yes/no	Regression to investigate whether baseline-to-4w change in OSPRO-YF-10 score is influencing the likelihood of having diagnostic tests or imaging consultations (reference: no use) after PT treatment while also accounting for age (NS), sex (NS), race (NS), anatomical pain region (NS), insurance (NS), chronicity (NS), surgery for	M	NS

				current condition (NS), comorbidity index (in final model), baseline disability (in final model), baseline pain intensity (NS), baseline OSPRO-YF-10 score (NS), baseline OSPRO-YF remaining 7 items (NS), baseline OSPRO-ROS score (NS) and baseline-to-4w change in pain intensity (in final model) and region-specific disability (NS).			
Vervoort (2019) [77]	199 Fibromyalgia	Revised Fibromyalgia Illness Perception Questionnaire – Emotional representations subscale	Recurrent secondary care user at 18m follow-up → yes/no	Regression investigating the influence of baseline level of IPQR emotional representations subscale on the likelihood of recurrent secondary care use (reference: no secondary care use).	U	OR: 1.01; 95%CI: .95-1.07 p=.72	
Psychological distress x tertiary care consultations							
Dobkin (2006) [16]	142 Fibromyalgia	Symptom Checklist 90-R	Attending tertiary care → yes/no	Comparison of psychological distress levels between tertiary care and community patients.	U	Tertiary care: 1.40 Community: 1.18 p=.063	Univariate ? <4
Psychological distress x emergency HCU							
Lentz (2018) [42]	246 Musculoskeletal pain	OSPRO Yellow Flag Tool – 10-item version (OSPRO-YF-10) (baseline)	Having ER visits after PT treatment → yes/no	Regression investigating the influence of baseline OSPRO-YF-10 score on the likelihood of having ER visits (reference: no visits) after PT treatment while also accounting for sex, race, comorbidity, OSPRO Review of Systems score (10-item + 13 items), baseline OSPRO-YF remaining 7 items score, baseline pain intensity, chronicity, change in disability, (all above: NS – omitted from final model), age (S), anatomical region of pain (S), insurance (S), surgery for current condition (NS), baseline disability (S), OSPRO-YF-10 change score	M	NS (omitted from final model)	Univariate ? <4<br><br>Multivariate 00 1/4 25%

				(NS) and change in pain intensity (S).		
		OSPRO Yellow Flag Tool (OSPRO-YF) – remaining 7 items (baseline)	Having ER visits after PT treatment → yes/no	Regression investigating the influence of baseline OSPRO-YF 7 remaining items score on the likelihood of having ER visits (reference: no visits) after PT treatment while also accounting for sex, race, comorbidity, OSPRO Review of Systems score (10-item + 13 items), baseline 10-item OSPRO-YF score, baseline pain intensity, chronicity, change in disability, (all above: NS – omitted from final model), age (S), anatomical region of pain (S), insurance (S), surgery for current condition (NS), baseline disability (S), OSPRO-YF-10 change score (NS) and change in pain intensity (S).	M	NS (omitted from final model)
		OSPRO Yellow Flag Tool - 10-item version (OSPRO-YF-10) (baseline-to-4w change score)	Having ER visits after PT treatment → yes/no	Regression investigating the influence of baseline-to-4w change in OSPRO-YF-10 score on the likelihood of having ER visits (reference: no visits) after PT treatment while also accounting for age (S), anatomical region of pain (S), insurance (S), surgery for current condition (NS), baseline disability (S) and change in pain intensity (S).	M	OR: 1.15<br>p=.05
Walker (2016) [82]	590 Undergoing gynecological surgery	Center for Epidemiologic Studies-Depression & State Trait Anxiety Inventory – Trait form	ER visits → yes/no	Regression investigating the influence of presence of depressive and/or anxiety symptoms (reference: no symptoms) on the likelihood of having at least 1 ER visit (reference: no visit).	U	OR: 2.27; 95%CI: 1.54-3.34<br>**p<.05**
				Regression investigating the influence of presence of	M	aOR: 2.00; 95%CI: 1.29-3.11<br>**p<.05**

		→ Depressive and/or anxiety symptoms vs not		depressive and/or anxiety symptoms (reference: no symptoms) on the likelihood of having at least 1 ER visit (reference: no visit) while also accounting for pain intensity (S), age (S), marital status (NS), employment status (S), education (NS), BMI (NS), current smoker (NS), previous abdominal surgery (NS), waiting time before surgery (S), menstruation status (NS), taking hormone replacement therapy (NS), taking birth control pills (NS) and preoperative malignancy (NS).			
Psychological distress x invasive procedures							
Lentz (2018) [42]	246 Musculoskeletal pain	OSPRO Yellow Flag Tool – 10-item version (OSPRO-YF-10) (baseline)	Receiving injections after PT treatment → yes/no	Regression investigating the influence of baseline OSPRO-YF-10 score on the likelihood of receiving injections (reference: no injections) after PT treatment while also accounting for age, sex, comorbidity, anatomical region of pain, OSPRO Review of Systems score (10-item + 13 items), baseline OSPRO-YF remaining 7 items score, OSPRO-YF-10 change score, insurance, baseline pain intensity, change in disability, surgery for current condition, change in pain intensity (all above: NS – omitted from final model), baseline disability (S), race (NS) and chronicity (NS).	M	NS (omitted from final model)	

	OSPRO Yellow Flag Tool (OSPRO-YF) – remaining 7 items (baseline)	Receiving injections after PT treatment → yes/no	Regression investigating the influence of baseline OSPRO-YF remaining 7 items score on the likelihood of receiving injections (reference: no injections) after PT treatment while also accounting for age, sex, comorbidity, anatomical region of pain, OSPRO Review of Systems score (10-item + 13 items), baseline 10-item OSPRO-YF score, OSPRO-YF-10 change score, insurance, baseline pain intensity, change in disability, surgery for current condition, change in pain intensity (all above: NS – omitted from final model), baseline disability (S), race (NS) and chronicity (NS).	M	NS (omitted from final model)	
	OSPRO Yellow Flag Tool → 10-item version (OSPRO-YF-10) (baseline-to-4w change score)	Receiving injections after PT treatment → yes/no	Regression investigating the influence of baseline-to-4w change in OSPRO-YF-10 score on the likelihood of receiving injections (reference: no injections) after PT treatment while also accounting for age, sex, comorbidity, anatomical region of pain, OSPRO Review of Systems score (10-item + 13 items), baseline 10-item OSPRO-YF score, OSPRO-YF remaining 7 items score, insurance, baseline pain intensity, change in disability, surgery for current condition, change in pain intensity (all above: NS – omitted from final model), baseline disability (S), race (NS) and chronicity (NS).	M	NS (omitted from final model)	
	OSPRO Yellow Flag Tool – 10-	Receiving surgery after PT treatment	Regression investigating the influence of baseline OSPRO-YF-	M	NS (omitted from final model)	

	item version (OSPRO-YF-10) (baseline)	→ yes/no	10 score on the likelihood of receiving surgery (reference: no surgery) after PT treatment while also accounting for age, sex, race, chronicity, anatomical region of pain, OSPRO Review of Systems score (10-item + 13 items), baseline OSPRO-YF remaining 7 items, insurance, baseline pain intensity, surgery for current condition, change in pain intensity (all above: NS – omitted from final model), baseline disability (S), change in disability (S), OSPRO-YF-10 change score (S) and comorbidity (NS).			
	OSPRO Yellow Flag Tool (OSPRO-YF) – remaining 7 items (baseline)	Receiving surgery after PT treatment → yes/no	Regression investigating the influence of baseline OSPRO-YF remaining 7 items score on the likelihood of receiving surgery (reference: no surgery) after PT treatment while also accounting for age, sex, race, chronicity, anatomical region of pain, OSPRO Review of Systems score (10-item + 13 items), baseline 10-item OSPRO-YF score, insurance, baseline pain intensity, surgery for current condition, change in pain intensity (all above: NS – omitted from final model), baseline disability (S), change in disability (S), OSPRO-YF-10 change score (S) and comorbidity (NS).	M	NS (omitted from final model)	
	OSPRO Yellow Flag Tool - 10-item version (OSPRO-YF-10)	Receiving surgery after PT treatment → yes/no	Regression investigating the influence of baseline-to-4w change in OSPRO-YF-10 score on the likelihood of receiving surgery (reference: no surgery)	M	OR: 1.14<br>**p=.02**	

		(baseline-to-4w change score)		after PT treatment while also accounting for baseline disability (S), change in disability (S) and comorbidity (NS).			
Navabi (2018)[5] [57]	283<br>Patients w/ endoscopic evaluation of irritable bowel disease	Hospital Anxiety and Depression Scale<br>→ presence of depressive and/or anxiety symptoms yes/no	History of surgery<br>→ yes/no	Regression investigating the influence of having a history of surgery (reference: no previous surgery) on the likelihood of showing depressive and/or anxiety symptoms (reference: no symptoms), while controlling for significant inflammation (NS), age (NS), disease duration (S), female gender (S), mesalamine use (NS), immunomodulator use (NS), Anti-TNF use (NS), corticosteroid use (NS), history of extra-intestinal manifestations (S), tobacco use (S) and opiate use (NS).	M	OR: 2.10; 95%CI: 1.16-3.79<br>**p=.01**	
Psychological distress x CAM use							
Trask (2001) [71]	292<br>Headache	Brief Symptom Inventory<br>→ low/medium/ high distress	Biofeedback use<br>→ yes/no	Chi²	U	% using biofeedback:<br>Low distress: 9.6%<br>Medium distress: 5.8%<br>High distress: 5.6%<br>NS	<u>Univariate</u><br>00<br>0/4 – 0%<br><br><u>Multivariate</u><br>?<br><4
			Relaxation use<br>→ yes/no	Chi²	U	% using relaxation:<br>Low distress: 30.8%<br>Medium distress: 28.9%<br>High distress: 34.7%<br>NS	
			Chiropractor use<br>→ yes/no	Chi²	U	% using chiropractor:<br>Low distress: 48.1%<br>Medium distress: 47.1%<br>High distress: 51.4%<br>NS	
			Acupuncture use<br>→ yes/no	Chi²	U	% using acupuncture:<br>Low distress: 18.3%<br>Medium distress: 12.4%<br>High distress: 19.4%<br>NS	

Thorstensson (2009) [69]	1,119 Chronic hip/knee pain	Euroqol EQ5D → Anxiety/ depression subscale → Anxiety/ depressive symptoms yes/no	Alternative therapist visits for knee/hip pain → yes/no	Regression investigating the influence of presence of anxiety/depressive symptoms (reference: no symptoms) on the likelihood of consulting an alternative therapist (reference: no consultation) while also accounting for age (NS) and sex (S).	M	OR: .84; 95%CI: .43-1.65 NS	
Symptom vigilance x primary care consultations							
Macfarlane (1999) [46]	252 Chronic widespread pain	Illness Attitude Scale – Bodily preoccupations subscale	GP consultation for pain → yes/no	Comparison of level of bodily preoccupations between consulters and non-consulters.	U	NS	Univariate ? <4
Tanatophobia x primary care consultations							
Macfarlane (1999) [46]	252 Chronic widespread pain	Illness Attitude Scale – Thanatophobia subscale	GP consultation for pain yes/no	Comparison of level of thanatophobia between consulters and non-consulters.	U	NS	Univariate ? <4
**POSITIVE CEF CLUSTERS**							
Illness coherence x pain medication use							
Hill (2007) [29]	2,113 Musculoskeletal hand problems	Illness Perception Questionnaire - Revised (IPQ-R) – Illness coherence subscale	Pain medication use → yes/no	Regression investigating the influence of IPQ-R illness coherence score on the likelihood of using pain medication (reference: no use).	U	OR: .98; 95%CI: .96-1.00 **significant**	Univariate ? <4
				Regression investigating the influence of IPQ-R illness coherence score on the likelihood of using pain medication (reference: no use) while also accounting sex (NS – fixed factor), age (NS – fixed factor), diagnosis (S – fixed factor), IPQ-R items (timeline cyclical, identity, consequences, emotional representations and treatment control; all S) and frustration (S).	M	OR: .95; 95%CI: .91-.99 **significant**	Multivariate ? <4
Illness coherence x primary care consultations							

Hill (2007) [29]	2,113 Musculoskeletal hand problems	Illness Perception Questionnaire – Revised (IPQ-R) – Illness coherence subscale	GP consultation → yes/no	Regression investigating the influence of IPQ-R coherence score on the likelihood of pain medication use (reference: no use) while also accounting for IPQ-R items (timeline cyclical, personal control, illness coherence and psychological attributions), frustration (all above: NS – omitted from final model), sex (NS – fixed factor), age (S – fixed factor), diagnosis (NS – fixed factor) and remaining IPQ-R items (timeline acute/chronic, consequences, treatment control, emotional representations and identity; all S).	M	NS (omitted from final model)	Multivariate<br>?<br><4
Illness coherence x secondary care consultations							
Vervoort (2019) [77]	199 Fibromyalgia	IPQR-FM – Illness coherence subscale (Baseline)	Recurrent secondary care user at 18m follow-up → yes/no	Regression to investigate the influence of level of IPQR illness coherence subscale on the likelihood of recurrent secondary care use (reference: no secondary care use).	U	NS	Univariate<br>?<br><4
Pain acceptance x prescription pain medication use							
Kratz (2018) [39]	120 Spinal cord injury w/ chronic pain	Chronic Pain Acceptance Questionnaire – Total score	Gabapentin use → yes/no	Regression investigating the influence of level of pain acceptance on the likelihood of using gabapentin (reference: no use) while also accounting for pain intensity (NS), number of painful body areas (S) and depressive symptoms (NS).	M	OR: .98; 95%CI: .95-1.00<br>p=.08	Multivariate<br>?<br><4
		Chronic Pain Acceptance Questionnaire (CPAQ) – Pain willingness subscale	Gabapentin use → yes/no	Regression investigating the influence of level of CPAQ pain willingness score on the likelihood of using gabapentin (reference: no use) while also accounting for pain intensity (NS), number of painful body	M	OR: .94; 95%CI: .89-1.00<br>**p=.04**	

				areas (S), CPAQ activities engagement (NS) and depressive symptoms (NS).			
		Chronic Pain Acceptance Questionnaire (CPAQ) – Activities engagement subscale	Gabapentin use → yes/no	Regression investigating the influence of level of CPAQ activities engagement score on the likelihood of using gabapentin (reference: no use) while also accounting for pain intensity (NS), number of painful body areas (S), CPAQ pain willingness (S) and depressive symptoms (NS).	M	OR: .53; 95%CI: .95-1.05<br>p=.53	
Pain acceptance x opioid use							
Kratz (2018) [39]	120<br>Spinal cord injury w/ chronic pain	Chronic Pain Acceptance Questionnaire	Opioid use → yes/no	Regression investigating the influence of level of pain acceptance on the likelihood of using opioids (reference: no use) while also accounting for pain intensity (NS), number of painful body areas (NS) and depressive symptoms (NS).	M	OR: .97; 95%CI: .94-.99<br>**p=.03**	<u>Multivariate</u><br>?<br><4
		Chronic Pain Acceptance Questionnaire (CPAQ) – Pain willingness subscale	Opioid use → yes/no	Regression investigating the influence of level of CPAQ pain willingness score on the likelihood of using opioids (reference: no use) while also accounting for pain intensity (NS), number of painful body areas (NS), CPAQ activities engagement (S) and depressive symptoms (NS).	M	OR: .99; 95%CI: .95-1.05<br>p=.90	
		Chronic Pain Acceptance Questionnaire (CPAQ) – Activities engagement subscale	Opioid use → yes/no	Regression investigating the influence of level of CPAQ activities engagement score on the likelihood of using opioids (reference: no use) while also accounting for pain intensity (NS), number of painful body areas (NS), CPAQ pain willingness	M	OR: .95; 95%CI: .90-.99<br>**p=.03**	

				(NS) and depressive symptoms (NS).			
Pain acceptance x secondary care consultations							
Vervoort (2019) [77]	199 Fibromyalgia	Illness Cognition Questionnaire – Acceptance subscale (Baseline)	Recurrent secondary care user at 18m follow-up → yes/no	Regression investigating the influence of baseline level of pain acceptance on the likelihood of recurrent secondary care use (reference: no secondary care use).	U	OR: .96; 95%CI: .89-1.03<br>p=.28	<u>Univariate</u><br>?<br><4
Perceived benefits x secondary care consultations							
Vervoort (2019) [77]	199 Fibromyalgia	Illness Cognition Questionnaire – Perceived benefits subscale	Recurrent secondary care user at 18m follow-up → yes/no	Regression investigating the influence of baseline level of perceived benefits subscale on the likelihood of recurrent secondary care use (reference: no secondary care use).	U	OR: 1.00; 95%CI: .93-1.07<br>p=.96	<u>Univariate</u><br>?<br><4
Perceived symptom control x pain medication use							
Hill (2007) [29]	2,113 Musculoskeletal hand problems	Illness Perception Questionnaire – Revised (IPQ-R) – Treatment control subscale	Pain medication use → yes/no	Regression investigating the influence of IPQ-R treatment control score on the likelihood of using pain medication (reference: no use).	U	OR: .97; 95%CI: .94-1.00<br>**Significant**	<u>Univariate</u><br>?<br><4
				Regression investigating the influence IPQ-R treatment control score on the likelihood of using pain medication (reference: no use) while also accounting for sex (NS – fixed factor), age (NS – fixed factor), diagnosis (S – fixed factor), IPQ-R subscales (emotional representations, timeline cyclical, identity, consequences, and illness coherence; all S) and frustration (S).	M	OR: 1.09; 95%CI: 1.04-1.15<br>**Significant**	<u>Multivariate</u><br>?<br><4

		Illness Perception Questionnaire – Revised (IPQ-R) – Personal control subscale	Pain medication use → yes/no	Regression investigating the influence of IPQ-R personal control score on (reference: low score) on the likelihood of using pain medication (reference: no use) while also accounting for IPQ-R items (timeline acute/chronic and psychological attributions; both NS – omitted from final model), sex (NS – fixed factor), age (NS – fixed factor) and diagnosis (S – fixed factor), remaining IPQ-R items (timeline cyclical, consequences, treatment control, emotional representations, illness coherence and identity; all S) and frustration score (S).	M	NS (omitted from final model)	
Perceived symptom control x consultations							
Macfarlane (2003) [47]	555<br>Orofacial pain	Self-designed question for pain control<br>→ Subdivided into score groups:<br>- 0-2<br>- 3-4<br>- 5-6 (reference)	Consultation for orofacial pain<br>→ yes/no	Regression investigating the influence of level of pain control (reference score: 5-6) on the likelihood of having a healthcare consultation for orofacial pain (reference: no consultation).	U	Score 3-4: RR: 1.47; 95%CI: 1.16-1.87<br>Score 0-2: RR: 1.66; 95%CI: 1.27-2.16<br>**p=.0001**	<u>Univariate</u><br>?<br><4
Perceived symptom control x primary care consultations							
Hill (2007) [29]	2,113<br>Musculoskeletal hand problems	Illness Perception Questionnaire – Revised (IPQ-R) – Treatment control subscale	GP consultation → yes/no	Regression investigating the influence of IPQ-R treatment control score on the likelihood of having a GP consultation (reference: no consultation).	U	OR: 1.00; 95%CI: .97-1.03<br>NS	<u>Univariate</u><br>?<br><4<br><br><u>Multivariate</u><br>?<br><4
				Regression investigating the influence of IPQ-R treatment control score on the likelihood of having a GP consultation (reference: no consultation) while also accounting for sex (NS – fixed factor), age (S – fixed factor), diagnosis (NS – fixed	M	OR: 1.17; 95%CI: 1.10-1.25<br>**Significant**	

				factor) and IPQ-R subscales (timeline acute/chronic, identity, consequences and emotional representations; all S).			
		Illness Perception Questionnaire – Revised (IPQ-R) – Personal control subscale	GP consultation → yes/no	Regression investigating the influence of IPQ-R personal control score on the likelihood of pain medication use (reference: no use) while also accounting for IPQ-R items (timeline cyclical, illness coherence and psychological attributions), frustration (all above: NS – omitted from final model), sex (NS – fixed factor), age (S – fixed factor), diagnosis (NS – fixed factor) and remaining IPQ-R items (timeline acute/chronic, consequences, treatment control, emotional representations and identity; all S).	M	NS (omitted from final model)	
Perceived symptom control x secondary care consultations							
Vervoort (2019) [77]	199 Fibromyalgia	Revised Fibromyalgia Illness Perceptions Questionnaire – Personal control subscale (Baseline)	Recurrent secondary care user at 18m follow-up → yes/no	Regression investigating the influence of baseline level of perceived personal illness control subscale on the likelihood of recurrent secondary care use (reference: no secondary care use).	U	OR: .91; 95%CI: .83-.99 **p=.03**	Univariate<br>?<br><4<br><br>Multivariate<br>?<br><4
				Regression investigating the influence of baseline level of perceived personal illness control on the likelihood of recurrent secondary care use (reference: no secondary care use) while also accounting for severity of fibromyalgia, depressive and anxiety symptoms, helplessness negative consequences beliefs, active pain coping (all above: NS	M	NS (omitted from final model)	

				– omitted from final model) and comorbidity (S).			
		Revised Fibromyalgia Illness Perceptions Questionnaire – Treatment control subscales (Baseline)	Recurrent secondary care user at 18m follow-up → yes/no	Regression investigating the influence of level of perceived treatment control on the likelihood of recurrent secondary care use (reference: no secondary care use).	U	OR: .96; 95%CI: .87-1.06<br>p=.44	
Perceived symptom control x CAM use							
Ndao-Brumblay (2010)[5] [58]	5,079<br>Chronic pain	Likert scale for perceived pain control	CAM use → yes/no	Comparison of level of perceived pain control between CAM users and non-users.	U	Users: 1.60 ± 1.51<br>Non-users: 1.45 ± 1.54<br>**p≤.005**	<u>Univariate</u><br>+<br>3/4 - 75%<br><br><u>Multivariate</u><br>+<br>3/4 - 75%
				Regression investigating the influence of level of perceived pain control on the likelihood of using CAM modalities (reference: no use) while accounting for age (S), gender (NS), race (S), education (S), marital status (NS), pain care perception (S), pain prediction (NS), residence income (NS), comorbidities (S), number of operations (NS), pain duration (S), pain severity (S), depressive symptoms (NS) and functional limitations (S).	M	OR: 1.077<br>**p≤.005**	
			Acupuncture → yes/no	Comparison of level of perceived pain control between acupuncture users and non-users.	U	Users: 1.56 ± 1.52<br>Non-users: 1.49 ± 1.54<br>NS	
				Regression investigating the influence of level of perceived pain control on the likelihood of using acupuncture (reference: no use) while accounting for age (S), gender (NS), race (NS), education (S), marital status (NS), pain care perception (S), pain prediction (NS), residence income (NS),	M	OR: .996<br>NS	

			comorbidities (NS), number of operations (NS), pain duration (S), pain severity (S), depressive symptoms (NS) and functional limitations (NS).		
		Biofeedback/relaxation → yes/no	Comparison of level of perceived pain control between biofeedback/relaxation users and non-users.	U	Users: 1.67 ± 1.49<br>Non-users: 1.47 ± 1.54<br>**p≤.005**
			Regression investigating the influence of level of perceived pain control on the likelihood of using biofeedback/relaxation (reference: no use) while accounting for age (S), gender (NS), race (S), education (S), marital status (NS), pain care perception (NS), pain prediction (NS), residence income (NS), comorbidities (S), number of operations (NS), pain duration (S), pain severity (S), depressive symptoms (NS) and functional limitations (S).	M	OR: 1.114<br>**p≤.005**
		Manipulation → yes/no	Comparison of level of perceived pain control between manipulation users and non-users.	U	Users: 1.59 ± 1.52<br>Non-users: 1.47 ± 1.54<br>**p<.05**
			Regression investigating the influence of level of perceived pain control on the likelihood of using manipulation services (reference: no use) while accounting for age (NS), gender (NS), race (S), education (S), marital status (NS), pain care perception (S), pain prediction (NS), residence income (NS), comorbidities (S), number of operations (NS), pain duration	M	OR: 1.057<br>**p<.05**

				(S), pain severity (S) and functional limitations (NS).			
Self-efficacy beliefs x prescription pain medication use							
Torrance (2013) [70]	215 Chronic pain w/ neuropathic component	Pain Self-Efficacy Scale	Adequate trial of neuropathic pain drug → yes/no	Comparison of self-efficacy score between patients w/ and w/o adequate trial of a neuropathic pain drug.	U	w/ trial: 25.3 ± 15.5<br>w/o trial: 37.8 ± 16.4<br>**p<.001**	Univariate<br>?<br><4
Self-efficacy beliefs x secondary care consultations							
Boyer (2009) [4]	315 Fibromyalgia	Chronic Pain Self-Efficacy Scale – Pain management subscale	Attending rheumatology setting vs primary care	Comparison of self-efficacy for pain management score between users of a rheumatology setting and primary care users.	U	Rheumatology: 38.08 ± 21.66<br>Primary care: 44.02 ± 24.67<br>NS	Univariate<br>0<br>1/4 - 25%
		Chronic Pain Self-Efficacy Scale – Symptoms management subscale	Attending rheumatology setting vs primary care	Comparison of self-efficacy for symptoms management score between users of a rheumatology setting and primary care users.	U	Rheumatology: 60.51 ± 19.14<br>Primary care: 65.28 ± 20.09<br>NS	
		Chronic Pain Self-Efficacy Scale – Physical functioning subscale	Attending rheumatology setting vs primary care	Comparison of self-efficacy for physical functioning score between users of a rheumatology setting and primary care users.	U	Rheumatology: 67.92 ± 22.91<br>Primary care: 74.28 ± 22.22<br>NS	
		Chronic Pain Self-Efficacy Scale – Total	Attending rheumatology setting vs primary care	Comparison of self-efficacy score between users of a rheumatology setting and primary care users.	U	Rheumatology: 56.79 ± 17.27<br>Primary care: 62.64 ± 18.57<br>**p≤.01**	
Self-efficacy beliefs x CAM use							
Rosenberg (2008) [66]	463 Chronic noncancer pain	Pain Self-Efficacy Scale	CAM use → yes/no	Bivariate analysis investigating the influence of level of pain self-efficacy on the likelihood of using CAM services (reference: no use).	U	OR: 1.00; 95%CI: .99-1.01<br>p=.71	Univariate<br>?<br><4
**OTHER CEF CLUSTERS**							
Locus of control x secondary care consultations							
Boyer (2009) [4]	315 Fibromyalgia	Multidimensional Pain Locus of Control Scale – Internal locus of control subscale	Attending rheumatology setting vs primary care	Comparison of internal locus of control score between users of a rheumatology setting and primary care users.	U	Rheumatology: 58.24 ± 19.84<br>Primary care: 59.22 ± 25.25<br>NS	Univariate<br>?<br><4

		Multidimensional Pain Locus of Control Scale – Fate locus of control subscale	Attending rheumatology setting vs primary care	Comparison of fate locus of control score between users of a rheumatology setting and primary care users.	U	Rheumatology: 48.59 ± 23.64<br>Primary care: 48.69 ± 30.07<br>NS	
		Multidimensional Pain Locus of Control Scale – Chance locus of control subscale	Attending rheumatology setting vs primary care	Comparison of chance locus of control score between users of a rheumatology setting and primary care users.	U	Rheumatology: 21.98 ± 24.19<br>Primary care: 18.63 ± 23.85<br>NS	
Perceived cause of symptoms x pain medication use							
Hill<br>(2007) [29]	2,113 Musculoskeletal hand problems	Illness Perception Questionnaire – Revised (IPQ-R) – Psychological attributions subscale	Pain medication use → yes/no	Regression investigating the influence of IPQ-R psychological attributions score on the likelihood of using pain medication (reference: no use) while also accounting for IPQ-R items (timeline acute/chronic and personal control; both NS – omitted from final model), sex (NS – fixed factor), age (NS – fixed factor) and diagnosis (S – fixed factor), remaining IPQ-R items (timeline cyclical, consequences, treatment control, emotional representations, illness coherence and identity; all S) and frustration score (S).	M	NS (omitted from final model)	<u>Multivariate</u><br>?<br><4
Perceived cause of symptoms x primary care consultations							
Hill<br>(2007) [29]	2,113 Musculoskeletal hand problems	Illness Perception Questionnaire – Revised (IPQ-R) – Psychological attributions subscale	GP consultation → yes/no	Regression investigating the influence of IPQ-R psychological attributions score on the likelihood of pain medication use (reference: no use) while also accounting for IPQ-R items (personal control, illness coherence and timeline cyclical), frustration (all above: NS – omitted from final model), sex (NS – fixed factor), age (S – fixed	M	NS (omitted from final model)	<u>Multivariate</u><br>?<br><4

				factor), diagnosis (NS – fixed factor) and remaining IPQ-R items (timeline acute/chronic, consequences, treatment control, emotional representations and identity; all S).			

[1]If outcomes for CEF and HCU were measured at the same moment, the moment of assessment was not mentioned. If there was a difference in moment of assessment, than this was mentioned between brackets under the respective outcome.
[2]Multivariate analyses: If the independent variable of interest (CEF/HCU outcome) was part of the final model, then the remaining independent variables in the final model were mentioned (for information on potential other considered independent variables, see Table A2 with study characteristics characteristics), including their significance in the model. If the independent variable of interest (CEF/HCU outcome) was omitted from the final model, then all independent variables considered for the multivariate model were reported including information on whether they were retained in the model, and if so, their significance in the model.
[3]Effect sizes were reported if available, otherwise only the p-value and, if available, the direction of the relationship was reported (+/-).
[4]Strength of association was rated as follows:
+/-: ≥ 60% of the analyses reported a +/- association
?: 34-59% of the analyses reported a +/- association, or fewer than 4 studies investigated the association (<4)
0: ≤ 33% of the analyses reported an association
++/--/00: If after exclusion of high risk of bias studies the association (+/-) or absence of association (0) was still supported by ≥ 60% of the analyses the summary score was up/downgraded to ++/--/00.
[5]Study rated as 'high risk of bias'
Abbreviations: n: sample size; CEF: cognitive and emotional factors; HCU: healthcare utilization; U: univariate; M: multivariate; NS: non-significant; OTC: over-the-counter; r: Pearson's r correlation coefficient; w/: with; w/o: without; S: significant; OR: odd's ratio; p: p-value; 95%CI: 95% confidence interval; mg: milligram(s); d: day(s); MED: morphine equivalent dose; BMI: body mass index; m: month(s); ER: emergency room; CAM: complementary and alternative medicine; vs: versus; β: regression coefficient; GP: general practitioner; NSAID: non-steroidal anti-inflammatory drug; WOMAC: Western Ontario and McMaster Universities Osteoarthritis Index; PR: prevalence ratio; PT: physiotherapist/physical therapist; RRR: relative risk ratio; TENS: transcutaneous electrical nerve stimulation; y: year(s); ADL: activities of daily living; w: week(s)

1. Alschuler, K.N.; Jensen, M.P.; Ehde, D.M. The association of depression with pain-related treatment utilization in patients with multiple sclerosis. *Pain Med* **2012**, *13*, 1648-1657, doi:10.1111/j.1526-4637.2012.01513.x.
2. Asmundson, G.J.; Wright, K.D.; Norton, P.J.; Veloso, F. Anxiety sensitivity and other emotionality traits in predicting headache medication use in patients with recurring headaches: implications for abuse and dependency. *Addict Behav* **2001**, *26*, 827-840, doi:10.1016/s0306-4603(01)00245-3.
3. Biggs, A.M.; Aziz, Q.; Tomenson, B.; Creed, F. Do childhood adversity and recent social stress predict health care use in patients presenting with upper abdominal or chest pain? *Psychosomatic medicine* **2003**, *65*, 1020-1028, doi:10.1097/01.psy.0000097333.02618.8d.
4. Boyer, A.L.; Mira Pastor, M.A.; Calatayud, N.P.; Lopez-Roig, S.; Cantero Terol, M.C. Comparing fibromyalgia patients from primary care and rheumatology settings: clinical and psychosocial features. *Rheumatol Int* **2009**, *29*, 1151-1160, doi:10.1007/s00296-008-0818-y.
5. Buse, D.C.; Pearlman, S.H.; Reed, M.L.; Serrano, D.; Ng-Mak, D.S.; Lipton, R.B. Opioid use and dependence among persons with migraine: results of the AMPP study. *Headache* **2012**, *52*, 18-36, doi:10.1111/j.1526-4610.2011.02050.x.
6. Carroll, C.P.; Lanzkron, S.; Haywood, C., Jr.; Kiley, K.; Pejsa, M.; Moscou-Jackson, G.; Haythornthwaite, J.A.; Campbell, C.M. Chronic Opioid Therapy and Central Sensitization in Sickle Cell Disease. *Am J Prev Med* **2016**, *51*, S69-77, doi:10.1016/j.amepre.2016.02.012.
7. Carroll, C.P.; Cichowitz, C.; Yu, T.; Olagbaju, Y.O.; Nelson, J.A.; Campbell, T.; Lanzkron, S. Predictors of acute care utilization and acute pain treatment outcomes in adults with sickle cell disease: The role of non-hematologic characteristics and baseline chronic opioid dose. *Am J Hematol* **2018**, *93*, 1127-1135, doi:10.1002/ajh.25168.
8. Ciechanowski, P.; Sullivan, M.; Jensen, M.; Romano, J.; Summers, H. The relationship of attachment style to depression, catastrophizing and health care utilization in patients with chronic pain. *Pain* **2003**, *104*, 627-637, doi:10.1016/s0304-3959(03)00120-9.
9. Citero Vde, A.; Levenson, J.L.; McClish, D.K.; Bovbjerg, V.E.; Cole, P.L.; Dahman, B.A.; Penberthy, L.T.; Aisiku, I.P.; Roseff, S.D.; Smith, W.R. The role of catastrophizing in sickle cell disease--the PiSCES project. *Pain* **2007**, *133*, 39-46, doi:10.1016/j.pain.2007.02.008.
10. Cronan, T.A.; Serber, E.R.; Walen, H.R. Psychosocial Predictors of Health Status and Health Care Costs among People with Fibromyalgia. *Anxiety, Stress & Coping* **2002**, *15*, 261-274, doi:10.1080/1061580021000020725.
11. Cronin, R.M.; Dorner, T.L.; Utrankar, A.; Allen, W.; Rodeghier, M.; Kassim, A.A.; Jackson, G.P.; DeBaun, M.R. Increased Patient Activation Is Associated with Fewer Emergency Room Visits and Hospitalizations for Pain in Adults with Sickle Cell Disease. *Pain Med* **2018**, 10.1093/pm/pny194, doi:10.1093/pm/pny194.
12. Cronin, R.M.; Hankins, J.S.; Byrd, J.; Pernell, B.M.; Kassim, A.; Adams-Graves, P.; Thompson, A.; Kalinyak, K.; DeBaun, M.; Treadwell, M. Risk factors for hospitalizations and readmissions among individuals with sickle cell disease: results of a U.S. survey study. *Hematology* **2019**, *24*, 189-198, doi:10.1080/16078454.2018.1549801.
13. Daltroy, L.H.; Morlino, C.I.; Eaton, H.M.; Poss, R.; Liang, M.H. Preoperative education for total hip and knee replacement patients. *Arthritis care and research : the official journal of the Arthritis Health Professions Association* **1998**, *11*, 469-478.
14. de Boer, M.J.; Struys, M.M.; Versteegen, G.J. Pain-related catastrophizing in pain patients and people with pain in the general population. *Eur J Pain* **2012**, *16*, 1044-1052, doi:10.1002/j.1532-2149.2012.00136.x.

15. Demmelmaier, I.; Asenlof, P.; Lindberg, P.; Denison, E. Biopsychosocial predictors of pain, disability, health care consumption, and sick leave in first-episode and long-term back pain: a longitudinal study in the general population. *Int J Behav Med* **2010**, *17*, 79-89, doi:10.1007/s12529-009-9055-3.
16. Dobkin, P.L.; Sita, A.; Sewitch, M.J. Predictors of adherence to treatment in women with fibromyalgia. *Clin J Pain* **2006**, *22*, 286-294, doi:10.1097/01.ajp.0000173016.87612.4b.
17. Dura-Ferrandis, E.; Ferrando-Garcia, M.; Galdon-Garrido, M.J.; Andreu-Vaillo, Y. Confirming the mechanisms behind cognitive-behavioural therapy effectiveness in chronic pain using structural equation modeling in a sample of patients with temporomandibular disorders. *Clin Psychol Psychother* **2017**, *24*, 1377-1383, doi:10.1002/cpp.2114.
18. Elander, J.; Barry, T. Analgesic use and pain coping among patients with haemophilia. *Haemophilia : the official journal of the World Federation of Hemophilia* **2003**, *9*, 202-213.
19. Elander, J.; Duarte, J.; Maratos, F.A.; Gilbert, P. Predictors of painkiller dependence among people with pain in the general population. *Pain Med* **2014**, *15*, 613-624, doi:10.1111/pme.12263.
20. Engel, C.C.; von Korff, M.; Katon, W.J. Back pain in primary care: predictors of high health-care costs. *Pain* **1996**, *65*, 197-204, doi:10.1016/0304-3959(95)00164-6.
21. Fink-Miller, E.L.; Long, D.M.; Gross, R.T. Comparing chronic pain treatment seekers in primary care versus tertiary care settings. *J Am Board Fam Med* **2014**, *27*, 594-601, doi:10.3122/jabfm.2014.05.130311.
22. Gebauer, S.; Salas, J.; Scherrer, J.F.; Burge, S.; Schneider, F.D. Disability Benefits and Change in Prescription Opioid Dose. *Population health management* **2019**, 10.1089/pop.2018.0210, doi:10.1089/pop.2018.0210.
23. Gil, K.M.; Carson, J.W.; Porter, L.S.; Scipio, C.; Bediako, S.M.; Orringer, E. Daily mood and stress predict pain, health care use, and work activity in African American adults with sickle-cell disease. *Health psychology : official journal of the Division of Health Psychology, American Psychological Association* **2004**, *23*, 267-274, doi:10.1037/0278-6133.23.3.267.
24. Gorge, M.; Ziehm, J.; Farin, E. Health-care utilization of patients with chronic back pain before and after rehabilitation. *BMC health services research* **2017**, *17*, 812, doi:10.1186/s12913-017-2757-3.
25. Grant, M.M.; Gil, K.M.; Floyd, M.Y.; Abrams, M. Depression and functioning in relation to health care use in sickle cell disease. *Annals of behavioral medicine : a publication of the Society of Behavioral Medicine* **2000**, *22*, 149-157, doi:10.1007/bf02895779.
26. Hadlandsmyth, K.; Rosenbaum, D.L.; Craft, J.M.; Gervino, E.V.; White, K.S. Health care utilisation in patients with non-cardiac chest pain: a longitudinal analysis of chest pain, anxiety and interoceptive fear. *Psychol Health* **2013**, *28*, 849-861, doi:10.1080/08870446.2012.762100.
27. Harden, R.N.; Bruehl, S.; Siegler, J.; Cole, P.A. Pain, psychological status, and functional recovery in chronic pain patients on daily opioids: a case comparison. *J Back Musculoskelet Rehabil* **1997**, *9*, 101-108, doi:10.3233/bmr-1997-9203.
28. Harding, K.; Day, M.A.; Ehde, D.M.; Wood, A.E.; McCall, A.; Williams, R. Mental and Physical Health Correlates of Pain Treatment Utilization Among Veterans With Chronic Pain: A Cross-sectional Study. *Mil Med* **2019**, *184*, e127-e134, doi:10.1093/milmed/usy235.
29. Hill, S.; Dziedzic, K.; Thomas, E.; Baker, S.R.; Croft, P. The illness perceptions associated with health and behavioural outcomes in people with musculoskeletal hand problems: findings from the North Staffordshire Osteoarthritis Project (NorStOP). *Rheumatology (Oxford, England)* **2007**, *46*, 944-951, doi:10.1093/rheumatology/kem015.

30. Howell, S.; Talley, N.J. Does fear of serious disease predict consulting behaviour amongst patients with dyspepsia in general practice? *European journal of gastroenterology & hepatology* **1999**, *11*, 881-886, doi:10.1097/00042737-199908000-00012.
31. Huffman, K.L.; Rush, T.E.; Fan, Y.; Sweis, G.W.; Vij, B.; Covington, E.C.; Scheman, J.; Mathews, M. Sustained improvements in pain, mood, function and opioid use post interdisciplinary pain rehabilitation in patients weaned from high and low dose chronic opioid therapy. *Pain* **2017**, *158*, 1380-1394, doi:10.1097/j.pain.0000000000000907.
32. Jensen, M.P.; Turner, J.A.; Romano, J.M. Correlates of improvement in multidisciplinary treatment of chronic pain. *Journal of consulting and clinical psychology* **1994**, *62*, 172-179, doi:10.1037/0022-006x.62.1.172.
33. Jensen, M.K.; Thomsen, A.B.; Hojsted, J. 10-year follow-up of chronic non-malignant pain patients: opioid use, health related quality of life and health care utilization. *Eur J Pain* **2006**, *10*, 423-433, doi:10.1016/j.ejpain.2005.06.001.
34. Jordan, K.; Jinks, C.; Croft, P. A prospective study of the consulting behaviour of older people with knee pain. *Br J Gen Pract* **2006**, *56*, 269-276.
35. Joud, A.; Bjork, J.; Gerdle, B.; Grimby-Ekman, A.; Larsson, B. The association between pain characteristics, pain catastrophizing and health care use - Baseline results from the SWEPAIN cohort. *Scandinavian journal of pain* **2017**, *16*, 122-128, doi:10.1016/j.sjpain.2017.04.071.
36. Kapoor, S.; Thorn, B.E.; Allen, R.S.; Kilgo, G.R. Psychosocial predictors of health care utilization in chronic pain patients living in rural Alabama. Graduate School of The University of Alabama, 2012.
37. Kapoor, S.; Thorn, B.E. Healthcare use and prescription of opioids in rural residents with pain. *Rural and remote health* **2014**, *14*, 2879.
38. Keeley, P.; Creed, F.; Tomenson, B.; Todd, C.; Borglin, G.; Dickens, C. Psychosocial predictors of health-related quality of life and health service utilisation in people with chronic low back pain. *Pain* **2008**, *135*, 142-150, doi:10.1016/j.pain.2007.05.015.
39. Kratz, A.L.; F. Murphy J, r.; Kalpakjian, C.Z.; Chen, P. Medicate or Meditate? Greater Pain Acceptance is Related to Lower Pain Medication Use in Persons With Chronic Pain and Spinal Cord Injury. *Clin J Pain* **2018**, *34*, 357-365, doi:10.1097/AJP.0000000000000550.
40. Kuijper, T.M.; Luime, J.J.; Alves, C.; Barendregt, P.J.; van Zeben, J.; Bindels, P.J.; Hazes, J.M. Quality of life and health care use in patients with arthralgias without synovitis compared with patients diagnosed with early rheumatoid arthritis: data from an early arthritis cohort. *Arthritis Care Res (Hoboken)* **2014**, *66*, 379-386, doi:10.1002/acr.22126.
41. Lee, V.; Guthrie, E.; Robinson, A.; Kennedy, A.; Tomenson, B.; Rogers, A.; Thompson, D. Functional bowel disorders in primary care: factors associated with health-related quality of life and doctor consultation. *Journal of psychosomatic research* **2008**, *64*, 129-138, doi:10.1016/j.jpsychores.2007.09.004.
42. Lentz, T.A.; Beneciuk, J.M.; George, S.Z. Prediction of healthcare utilization following an episode of physical therapy for musculoskeletal pain. *BMC health services research* **2018**, *18*, 648, doi:10.1186/s12913-018-3470-6.
43. Levenson, J.L.; McClish, D.K.; Dahman, B.A.; Bovbjerg, V.E.; de, A.C.V.; Penberthy, L.T.; Aisiku, I.P.; Roberts, J.D.; Roseff, S.D.; Smith, W.R. Depression and anxiety in adults with sickle cell disease: the PiSCES project. *Psychosomatic medicine* **2008**, *70*, 192-196, doi:10.1097/PSY.0b013e31815ff5c5.
44. Lozano-Calderon, S.A.; Souer, J.S.; Jupiter, J.B.; Ring, D. Psychological differences between patients that elect operative or nonoperative treatment for trapeziometacarpal joint arthrosis. *Hand (N Y)* **2008**, *3*, 271-275, doi:10.1007/s11552-008-9098-y.

45. Lozier, C.C.; Nugent, S.M.; Smith, N.X.; Yarborough, B.J.; Dobscha, S.K.; Deyo, R.A.; Morasco, B.J. Correlates of Use and Perceived Effectiveness of Non-pharmacologic Strategies for Chronic Pain Among Patients Prescribed Long-term Opioid Therapy. *J Gen Intern Med* **2018**, *33*, 46-53, doi:10.1007/s11606-018-4325-x.
46. Macfarlane, G.J.; Morris, S.; Hunt, I.M.; Benjamin, S.; McBeth, J.; Papageorgiou, A.C.; Silman, A.J. Chronic widespread pain in the community: the influence of psychological symptoms and mental disorder on healthcare seeking behavior. *J Rheumatol* **1999**, *26*, 413-419.
47. Macfarlane, T.V.; Blinkhorn, A.S.; Davies, R.M.; Kincey, J.; Worthington, H.V. Factors associated with health care seeking behaviour for orofacial pain in the general population. *Community dental health* **2003**, *20*, 20-26.
48. Mann, E.G.; Johnson, A.; Gilron, I.; VanDenKerkhof, E.G. Pain Management Strategies and Health Care Use in Community-Dwelling Individuals Living with Chronic Pain. *Pain Med* **2017**, *18*, 2267-2279, doi:10.1093/pm/pnw341.
49. Mannion, A.F.; Wieser, S.; Elfering, A. Association between beliefs and care-seeking behavior for low back pain. *Spine (Phila Pa 1976)* **2013**, *38*, 1016-1025, doi:10.1097/BRS.0b013e31828473b5.
50. McCracken, L.M. ''Attention'' to pain in persons with chronic pain: A behavioral approach. *Behavior Therapy* **1997**, *28*, 271-284, doi:10.1016/s0005-7894(97)80047-0.
51. McCracken, L.M.; Eccleston, C. A prospective study of acceptance of pain and patient functioning with chronic pain. *Pain* **2005**, *118*, 164-169, doi:10.1016/j.pain.2005.08.015.
52. McCracken, L.M.; Vowles, K.E.; Eccleston, C. Acceptance-based treatment for persons with complex, long standing chronic pain: a preliminary analysis of treatment outcome in comparison to a waiting phase. *Behaviour research and therapy* **2005**, *43*, 1335-1346, doi:10.1016/j.brat.2004.10.003.
53. McCracken, L.M.; Vowles, K.E. Psychological flexibility and traditional pain management strategies in relation to patient functioning with chronic pain: an examination of a revised instrument. *J Pain* **2007**, *8*, 700-707, doi:10.1016/j.jpain.2007.04.008.
54. Mourad, G.; Stromberg, A.; Johansson, P.; Jaarsma, T. Depressive Symptoms, Cardiac Anxiety, and Fear of Body Sensations in Patients with Non-Cardiac Chest Pain, and Their Relation to Healthcare-Seeking Behavior: A Cross-Sectional Study. *Patient* **2016**, *9*, 69-77, doi:10.1007/s40271-015-0125-0.
55. Mourad, G.; Jaarsma, T.; Stromberg, A.; Svensson, E.; Johansson, P. The associations between psychological distress and healthcare use in patients with non-cardiac chest pain: does a history of cardiac disease matter? *BMC psychiatry* **2018**, *18*, 172, doi:10.1186/s12888-018-1689-8.
56. Musey, P.I., Jr.; Patel, R.; Fry, C.; Jimenez, G.; Koene, R.; Kline, J.A. Anxiety Associated With Increased Risk for Emergency Department Recidivism in Patients With Low-Risk Chest Pain. *Am J Cardiol* **2018**, *122*, 1133-1141, doi:10.1016/j.amjcard.2018.06.044.
57. Navabi, S.; Gorrepati, V.S.; Yadav, S.; Chintanaboina, J.; Maher, S.; Demuth, P.; Stern, B.; Stuart, A.; Tinsley, A.; Clarke, K., et al. Influences and Impact of Anxiety and Depression in the Setting of Inflammatory Bowel Disease. *Inflammatory bowel diseases* **2018**, *24*, 2303-2308, doi:10.1093/ibd/izy143.
58. Ndao-Brumblay, S.K.; Green, C.R. Predictors of complementary and alternative medicine use in chronic pain patients. *Pain Med* **2010**, *11*, 16-24, doi:10.1111/j.1526-4637.2009.00767.x.
59. Newman, A.K.; Kapoor, S.; Thorn, B.E. Health Care Utilization for Chronic Pain in Low-Income Settings. *Pain Med* **2018**, *19*, 2387-2397, doi:10.1093/pm/pny119.

60. Nielsen, S.; Lintzeris, N.; Bruno, R.; Campbell, G.; Larance, B.; Hall, W.; Hoban, B.; Cohen, M.L.; Degenhardt, L. Benzodiazepine use among chronic pain patients prescribed opioids: associations with pain, physical and mental health, and health service utilization. *Pain Med* **2015**, *16*, 356-366, doi:10.1111/pme.12594.
61. Osborne, R.H.; Wilson, T.; Lorig, K.R.; McColl, G.J. Does self-management lead to sustainable health benefits in people with arthritis? A 2-year transition study of 452 Australians. *J Rheumatol* **2007**, *34*, 1112-1117.
62. Page, M.G.; Boyd, K.; Ware, M.A. Examination of the Course of Low Back Pain Intensity Based on Baseline Predictors and Health Care Utilization Among Patients Treated in Multidisciplinary Pain Clinics: A Quebec Pain Registry Study. *Pain Med* **2019**, *20*, 564-573, doi:10.1093/pm/pny205.
63. Philpot, L.M.; Ramar, P.; Elrashidi, M.Y.; Sinclair, T.A.; Ebbert, J.O. A Before and After Analysis of Health Care Utilization by Patients Enrolled in Opioid Controlled Substance Agreements for Chronic Noncancer Pain. *Mayo Clin Proc* **2018**, *93*, 1431-1439, doi:10.1016/j.mayocp.2018.05.008.
64. Pierce, J.; Moser, S.; Hassett, A.L.; Brummett, C.M.; Christianson, J.A.; Goesling, J. Influence of Abuse History on Concurrent Benzodiazepine and Opioid Use in Chronic Pain Patients. *J Pain* **2019**, *20*, 473-480, doi:10.1016/j.jpain.2018.10.009.
65. Primavera, J.P., 3rd; Kaiser, R.S. The relationship between locus of control, amount of pre-admission analgesic/ergot overuse, and length of stay for patients admitted for inpatient treatment of chronic headache. *Headache* **1994**, *34*, 204-208, doi:10.1111/j.1526-4610.1994.hed3404204.x.
66. Rosenberg, E.I.; Genao, I.; Chen, I.; Mechaber, A.J.; Wood, J.A.; Faselis, C.J.; Kurz, J.; Menon, M.; O'Rorke, J.; Panda, M., et al. Complementary and alternative medicine use by primary care patients with chronic pain. *Pain Med* **2008**, *9*, 1065-1072, doi:10.1111/j.1526-4637.2008.00477.x.
67. Shmagel, A.; Foley, R.; Ibrahim, H. Epidemiology of Chronic Low Back Pain in US Adults: Data From the 2009-2010 National Health and Nutrition Examination Survey. *Arthritis Care Res (Hoboken)* **2016**, *68*, 1688-1694, doi:10.1002/acr.22890.
68. Talley, N.J.; Boyce, P.; Jones, M. Dyspepsia and health care seeking in a community: How important are psychological factors? *Digestive diseases and sciences* **1998**, *43*, 1016-1022, doi:10.1023/a:1018878717715.
69. Thorstensson, C.A.; Gooberman-Hill, R.; Adamson, J.; Williams, S.; Dieppe, P. Help-seeking behaviour among people living with chronic hip or knee pain in the community. *BMC Musculoskelet Disord* **2009**, *10*, 153, doi:10.1186/1471-2474-10-153.
70. Torrance, N.; Ferguson, J.A.; Afolabi, E.; Bennett, M.I.; Serpell, M.G.; Dunn, K.M.; Smith, B.H. Neuropathic pain in the community: more under-treated than refractory? *Pain* **2013**, *154*, 690-699, doi:10.1016/j.pain.2012.12.022.
71. Trask, P.C.; Iezzi, T.; Kreeft, J. Comparison of headache parameters using headache type and emotional status. *Journal of psychosomatic research* **2001**, *51*, 529-536, doi:10.1016/s0022-3999(01)00251-3.
72. Tremblay, M.A.; Denis, I.; Turcotte, S.; Fleet, R.P.; Archambault, P.; Dionne, C.E.; Foldes-Busque, G. Heart-focused anxiety and health care seeking in patients with non-cardiac chest pain: A prospective study. *Gen Hosp Psychiatry* **2018**, *50*, 83-89, doi:10.1016/j.genhosppsych.2017.10.007.
73. Tsuji, T.; Nakata, K.; Vietri, J.; Jaffe, D.H. The added burden of depression in patients with osteoarthritis in Japan. *ClinicoEconomics and outcomes research : CEOR* **2019**, *11*, 411-421, doi:10.2147/CEOR.S189610.
74. Ullrich, P.M.; Lincoln, R.K.; Tackett, M.J.; Miskevics, S.; Smith, B.M.; Weaver, F.M. Pain, depression, and health care utilization over time after spinal cord injury. *Rehabilitation psychology* **2013**, *58*, 158-165, doi:10.1037/a0032047.

75. Valdes, A.M.; Warner, S.C.; Harvey, H.L.; Fernandes, G.S.; Doherty, S.; Jenkins, W.; Wheeler, M.; Doherty, M. Use of prescription analgesic medication and pain catastrophizing after total joint replacement surgery. *Semin Arthritis Rheum* **2015**, *45*, 150-155, doi:10.1016/j.semarthrit.2015.05.004.
76. van Tilburg, M.A.; Palsson, O.S.; Levy, R.L.; Feld, A.D.; Turner, M.J.; Drossman, D.A.; Whitehead, W.E. Complementary and alternative medicine use and cost in functional bowel disorders: a six month prospective study in a large HMO. *BMC Complement Altern Med* **2008**, *8*, 46, doi:10.1186/1472-6882-8-46.
77. Vervoort, V.M.; Vriezekolk, J.E.; Olde Hartman, T.C.; van Helmond, T.; van der Laan, W.H.; Geenen, R.; van den Ende, C.H. Cognitive-behavioural and social factors do not predict recurrent secondary healthcare use in patients with fibromyalgia: a longitudinal study. *Clinical and experimental rheumatology* **2019**, *37 Suppl 116*, 44-50.
78. Villani, V.; Di Stani, F.; Vanacore, N.; Scattoni, L.; Cerbo, R.; Bruti, G. The "repeater" phenomenon in migraine patients: a clinical and psychometric study. *Headache* **2010**, *50*, 348-356, doi:10.1111/j.1526-4610.2009.01585.x.
79. Vina, E.R.; Hausmann, L.R.M.; Obrosky, D.S.; Youk, A.; Ibrahim, S.A.; Weiner, D.K.; Gallagher, R.M.; Kwoh, C.K. Social & psychological factors associated with oral analgesic use in knee osteoarthritis management. *Osteoarthritis and cartilage* **2019**, *27*, 1018-1025, doi:10.1016/j.joca.2019.01.010.
80. Von Korff, M.; Wagner, E.H.; Dworkin, S.F.; Saunders, K.W. Chronic pain and use of ambulatory health care. *Psychosomatic medicine* **1991**, *53*, 61-79, doi:10.1097/00006842-199101000-00006.
81. Von Korff, M.; Lin, E.H.; Fenton, J.J.; Saunders, K. Frequency and priority of pain patients' health care use. *Clin J Pain* **2007**, *23*, 400-408, doi:10.1097/AJP.0b013e31804ac020.
82. Walker, S.; Hopman, W.M.; Carley, M.E.; Mann, E.G.; VanDenKerkhof, E.G. Healthcare Use for Pain in Women Waiting for Gynaecological Surgery. *Pain Res Manag* **2016**, *2016*, 1343568, doi:10.1155/2016/1343568.
83. Wideman, T.H.; Sullivan, M.J. Differential predictors of the long-term levels of pain intensity, work disability, healthcare use, and medication use in a sample of workers' compensation claimants. *Pain* **2011**, *152*, 376-383, doi:10.1016/j.pain.2010.10.044.
84. Wijnhoven, H.A.; de Vet, H.C.; Picavet, H.S. Sex differences in consequences of musculoskeletal pain. *Spine (Phila Pa 1976)* **2007**, *32*, 1360-1367, doi:10.1097/BRS.0b013e31805931fd.
85. Williams, R.E.; Black, C.L.; Kim, H.Y.; Andrews, E.B.; Mangel, A.W.; Buda, J.J.; Cook, S.F. Determinants of healthcare-seeking behaviour among subjects with irritable bowel syndrome. *Aliment Pharmacol Ther* **2006**, *23*, 1667-1675, doi:10.1111/j.1365-2036.2006.02928.x.
86. Williams, H.; Silva, R.N.S.; Cline, D.; Freiermuth, C.; Tanabe, P. Social and Behavioral Factors in Sickle Cell Disease: Employment Predicts Decreased Health Care Utilization. *J Health Care Poor Underserved* **2018**, *29*, 814-829, doi:10.1353/hpu.2018.0060.
87. Wong, M.; Vogell, A.; Wright, K.; Isaacson, K.; Loring, M.; Morris, S. Opioid use after laparoscopic hysterectomy: prescriptions, patient use, and a predictive calculator. *American journal of obstetrics and gynecology* **2019**, *220*, 259 e251-259 e211, doi:10.1016/j.ajog.2018.10.022.
88. Woodhouse, A.; Pape, K.; Romundstad, P.R.; Vasseljen, O. Health care contact following a new incident neck or low back pain episode in the general population; the HUNT study. *BMC health services research* **2016**, *16*, 81, doi:10.1186/s12913-016-1326-5.

89. Zebenholzer, K.; Lechner, A.; Broessner, G.; Lampl, C.; Luthringshausen, G.; Wuschitz, A.; Obmann, S.M.; Berek, K.; Wober, C. Impact of depression and anxiety on burden and management of episodic and chronic headaches - a cross-sectional multicentre study in eight Austrian headache centres. *J Headache Pain* **2016**, *17*, 15, doi:10.1186/s10194-016-0603-3.
90. Zondervan, K.T.; Yudkin, P.L.; Vessey, M.P.; Jenkinson, C.P.; Dawes, M.G.; Barlow, D.H.; Kennedy, S.H. The community prevalence of chronic pelvic pain in women and associated illness behaviour. *Br J Gen Pract* **2001**, *51*, 541-547.
